# Bench-Stable 2-Halopyridinium Ketene Hemiaminals as Reagents for the Synthesis of 2-Aminopyridine Derivatives

**DOI:** 10.1021/acs.orglett.4c02915

**Published:** 2024-09-20

**Authors:** Isabelle C. Bote, Zoe A. Krevlin, Maria Christina F. Crespo, Sudchananya Udomphan, Carolyn T. Levin, Christie C. Lam, Amy M. Glanzer, Holly L. Hutchinson, Alisha M. Blades, Danielle L. McConnell, Crystal Lin, John P. Frank, William R. Strutton, Jordan C. Merklin, Beau A. Sinardo, Khady J. Gueye, Karly V. Leiman, Ashley Thayaparan, Joel K. A. Adade, Nestor L. Martinez, Wesley W. Kramer, Max M. Majireck

**Affiliations:** Chemistry Department, Hamilton College, 198 College Hill Rd., Clinton, New York 13323, United States

## Abstract

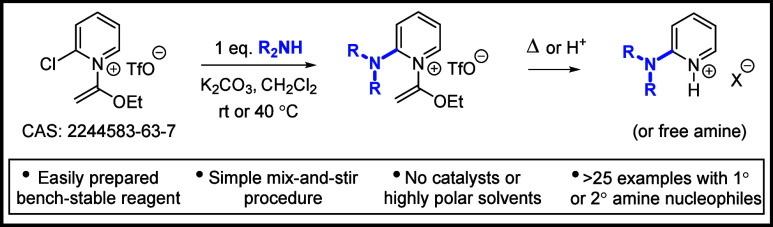

2-Chloro-1-(1-ethoxyvinyl)pyridinium triflate and several other bench-stable *N*-(1-alkoxyvinyl) 2-halopyridinium triflates have been developed as reagents for the synthesis of valuable 2-aminopyridine scaffolds via unusually mild S_N_Ar substitutions with amine nucleophiles. Advantages of this approach include an operationally simple mix-and-stir procedure at room temperature or mild heat and ambient atmosphere and without the need for transition metal catalysts, coupling reagents, or high-boiling solvents. The stable *N*-(1-ethoxyvinyl) moiety serves as a dual S_N_Ar-activating group and pyridine *N*-protecting group that can be cleaved under thermal, acidic, or oxidative conditions. Preliminary results of other nucleophilic substitutions using oxygen-, sulfur-, and carbon-based nucleophiles are also demonstrated.

2-Aminopyridines are regularly targeted by medicinal chemists due to their frequent occurrence within drug-like compounds.^1^ Of the existing approaches to this biologically versatile scaffold, 2-halopyridines are attractive starting materials due to their accessibility and participation in a broad range of transformations such as nucleophilic aromatic substitutions^2^ (S_N_Ar) or metal-catalyzed cross-couplings with amines.^1c,3^ S_N_Ar-based protocols, while direct, typically involve high temperatures, highly polar solvents, or an excess of base/nucleophile which complicates purification of the polar 2-aminopyridine products (Scheme 1A).^2^ Transition metal catalyzed cross-couplings circumvent some of these issues, but often introduce assay-interfering transition metal contaminants (e.g., Ni, Cu, Zn, or Pd),^4^ which is a particular issue for metal-chelating, basic amine products like 2-aminopyridines (Scheme 1B).^5^

**Scheme 1:**
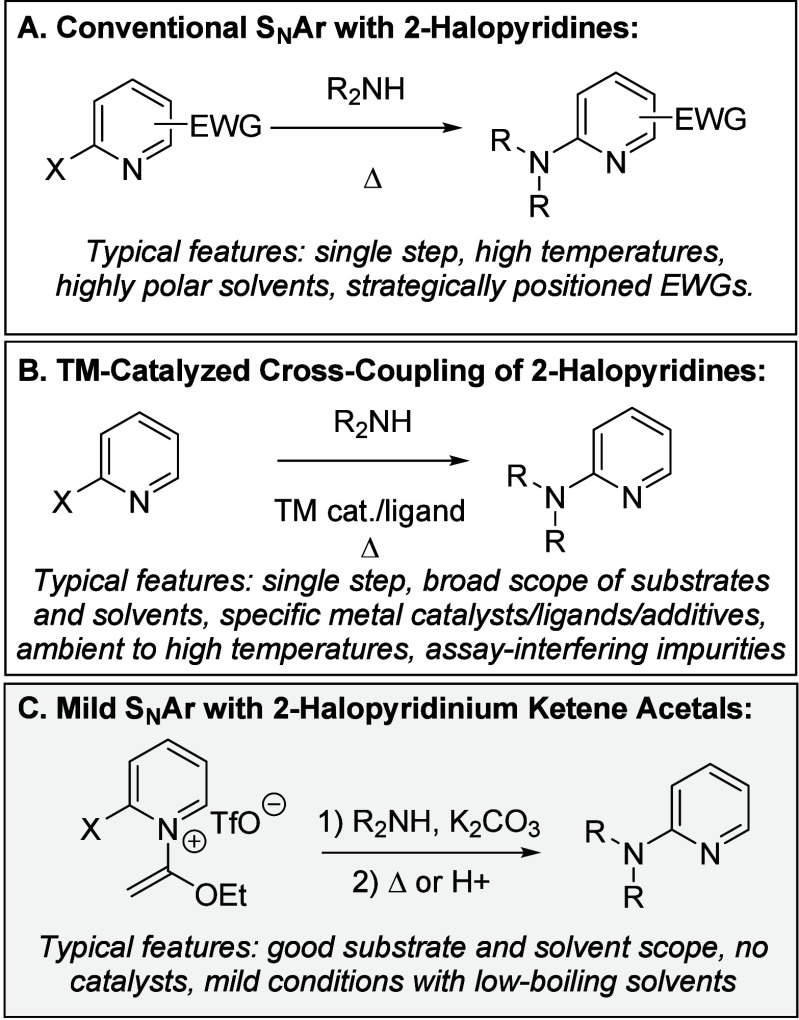
Comparison of 2-Halopyridine Aminations

Previously, our laboratory discovered a broad range of stable pyridinium ketene hemiaminals that can be used as reagents in a variety of useful transformations.^6,7^ Among these products, we found that *N*-(1-ethoxyvinyl)-2-halopyridinium salts undergo unusually mild room-temperature S_N_Ar reactions with amine nucleophiles (Scheme 1C). Our new approach offers a combination of advantages compared to conventional S_N_Ar and metal-catalyzed transformations, most notably (i) a simple procedure under exceptionally mild conditions (e.g., ambient atmosphere, room temperature, or mild warming); (ii) avoidance of high-boiling polar solvents that are difficult to remove and/or complicate extraction of the polar amine products (e.g., DMSO or DMF); and (iii) lack of assay-interfering transition-metal catalysts/ligands. Moreover, the cleavable *N*-(1-ethoxyvinyl) moiety dually serves as an activating group enabling mild S_N_AR reactions^8^ and a protecting group of the basic pyridine *N*-atom, suppressing undesired side reactivity or metal-chelation.

To expand on our preliminary findings,^6c^ we initially focused on developing S_N_Ar protocols in which equimolar ratios of the amine nucleophile and 2-halopyridinium salt reactants were simply mixed together, without additives, in a range of different solvents to produce 2-aminopyridinium salt **2a** directly (Table 1, entry 1). Although we found that this procedure was amenable to a range of substrates on a 0.5 mmol scale, many of the reactions required more than one purification to remove persistent side products (e.g., HCl-salts of the amine nucleophile). Increasing the equivalents of amine nucleophile, or including organic soluble bases like triethylamine yielded complex mixtures with occasional isolation of α-pyridone side product **3**, presumably due to base-promoted conversion of desired product **2a** to an iminopyridine intermediate that is subsequently hydrolyzed. This issue was slightly alleviated by running an additive-free reaction (entry 2), then rapidly purifying the compound on silica gel with 0.5% triethylamine in the eluting chloroform/isopropanol solvents. In this case, clean product was delivered in good yield with minimal formation of side product **3**. We deemed this method suitable for exceptionally mild generation of 2-aminopyridinium salts, especially for medicinal chemistry studies that must avoid contaminating additives. However, the formation of hydrolyzable iminopyridine intermediates on silica compromises the scalability and generality of this approach.

**Table 1:**
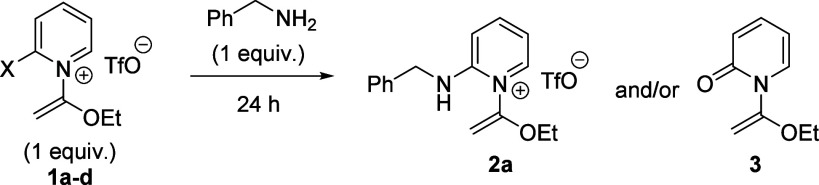
Initial Optimization of S_N_Ar Protocol

Entry	X	Additive	Solvent	Temp	Results
1	Cl (1a)	-	CH_2_Cl_2_	rt	**2a**, 74%
2	Cl (1a)	Et_3_N-SiO_2_^a^	CH_2_Cl_2_	rt	**2a**, 89% + **3**, 10%
3	Cl (1a)	Li_2_CO_3_	CH_2_Cl_2_	rt	**2a**, 71%
4	Cl (1a)	Na_2_CO_3_	CH_2_Cl_2_	rt	**2a**, 72%
5	Cl (1a)	K_2_CO_3_	CH_2_Cl_2_	rt	**2a**, 79%
6	Cl (1a)	Cs_2_CO_3_	CH_2_Cl_2_	rt	**2a**, 75%
7	Cl (1a)	CaCO_3_	CH_2_Cl_2_	rt	**2a**, 74%
8	Cl (1a)	AgCO_3_	CH_2_Cl_2_	rt	**2a**, 68%
9	F (1b)	-	CH_2_Cl_2_	0 °C-rt	**2a**, ∼83%^b^^,^^c^
10	Br (1c)	K_2_CO_3_	CH_2_Cl_2_	rt	**2a**, ∼40%^b^
11	I (1d)	K_2_CO_3_	CH_2_Cl_2_	rt	**2a**, 25%
12	Cl (1a)	K_2_CO_3_	THF	rt	**2a**, 45%
13	Cl (1a)	K_2_CO_3_	MeCN	rt	**2a**, 44%
14	Cl (1a)	K_2_CO_3_	DMF	rt	**2a**, ∼75%^b^
15	Cl (1a)	K_2_CO_3_	DMSO	rt	complex mix
16	Cl (1a)	K_2_CO_3_	CH_2_Cl_2_	40 °C	**2a**, 98%
17	Cl (1a)	K_2_CO_3_	CHCl_3_	40 °C	**2a**, 64%
18	Cl (1a)	K_2_CO_3_	1,2-DCE	40 °C	**2a**, 49%
19	Cl (1a)	K_2_CO_3_	PhH	40 °C	**2a**, 62%
20	Cl (1a)	K_2_CO_3_	PhMe	40 °C	**2a**, 64%
21	Cl (1a)	K_2_CO_3_	Et_2_O	40 °C	**2a**, 79%
22	Cl (1a)	K_2_CO_3_	1,4-dioxane	40 °C	**2a**, 40%
23	Cl (1a)	K_2_CO_3_	glyme	40 °C	**2a**, 40%
24	Cl (1a)	K_2_CO_3_	hexanes	40 °C	**2a**, 53%

a No additive during the reaction; the crude product was chromatographed on Et_3_N-treated silica gel using an isopropanol/chloroform gradient.

b Approximate yield due to presence of a coeluting unknown impurity.

c **1b** is formed in situ; 1.5 equiv of benzylamine; no base was added to avoid competitive hydrolysis of **1b** to **3**.

To circumvent iminopyridine formation, we screened sparingly soluble acid-scavenging bases (Table 1, entries 3–8). Alkali metal carbonates performed generally well, delivering clean product **2a** without any observable hydrolysis product **3**. In further optimization experiments, we used potassium carbonate given its superior performance and low cost.

To gauge the broader reactivity of related 2-halopyridinium salts, we examined different halogen leaving groups (entries 9–11). Remarkably, even the less electrophilic 2-bromo and 2-iodopyridinium salt analogues **1c**–**d** were reactive toward amine nucleophiles at room temperature. The highly reactive 2-fluoropyridinium salt **1b** required an alternative protocol since it is generated at higher purity in situ and the use of base additives promotes degradation.^6c,6d^ Nevertheless, this analogue reacts rapidly with benzylamine, even at a low temperature, to deliver product **2a** in good yield but moderate purity.

We found that cheap and easily removed dichloromethane outperformed more polar solvents typical for S_N_Ar reactions involving amine nucleophiles (Table 1, entries 12–15),^2a−2c^ giving nearly quantitative yield of **2a** with gentle warming of the reaction mixture to 40 °C (cf. entries 5 and 16). As our work was nearing completion, the Environmental Protection Agency drafted new policies to limit the use of dichloromethane,^9^ prompting us to screen additional solvents commonly used as substitutes (Table 1, entries 17–25). In this supplemental screen, we identified several workable alternatives that, like dichloromethane, promoted an efficient S_N_Ar reaction and were also relatively nonpolar and easy to remove via rotary evaporation.

With an optimized S_N_Ar protocol developed for 2-chloropyridinium salt reagent **1a**, we tested a range of primary and secondary amine nucleophiles (Scheme 2). Generally, benzylamine derivatives performed well, delivering their corresponding products **2a**–**k** in moderate to excellent yield. Methoxy, halo, amino, and even hydroxy substitutions on the benzene ring were compatible in this process. Notably, products **2j**–**k** and **2q**–**s**, derived from sterically hindered primary or secondary amines, could be formed under these conditions. Common bioactive pharmacophores, in addition to benzylamines, were also tolerated, such as phenethylamine **2n**, anilines **2s**–**v**, or various heterocycles including pyridine **2m**, benzothiophene **2l**, morpholine **2q**, and indole **2y**. Double S_N_Ar substitutions (**2w**–**x**) were also possible when diamines are employed.

**Scheme 2:**
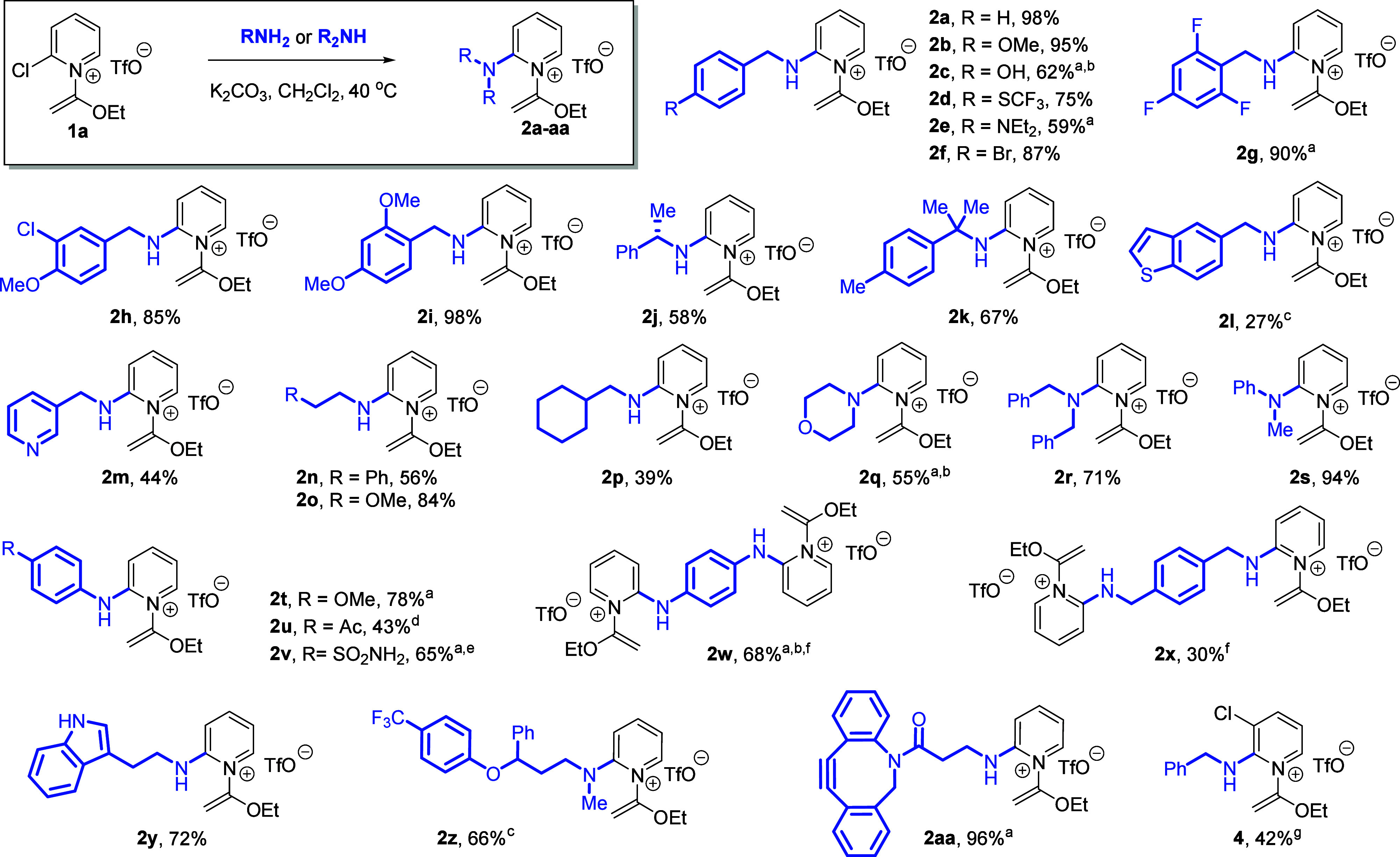
Substrate Scope of Amine Nucleophiles Approximate yield due to unknown minor impurities. Yield estimated by qNMR. With HCl-amine salt and 2 equiv K_2_CO_3_. 4-Ethynylaniline used as nucleophile. Prepared using 2-fluoropyridinium **1b** and no K_2_CO_3_. With 0.5 equiv. diamine. With 2,3-dichloropyridinium salt instead of **1a**.

We also used a 2,3-dichloropyridinium salt derivative en route to the synthesis of **4**, in which the remaining 3-chloro substituent could be leveraged for additional transformations. Bulky secondary amines such as dibenzylamine or diisopropylamine and weaker *N*-nucleophiles bearing strongly electron withdrawing groups (e.g., benzamide or benzenesulfonamide) were incompatible with this procedure, generally yielding α-pyridone hydrolysis product **3** following chromatography.

Several products were generated using known small molecule drugs or probes. When the antibiotic sulfanilamide was used as the nucleophile and 2-fluoropyridinium salt **1b** as the electrophile, compound **2v** was formed as the major product wherein the aniline *N*-atom served as the nucleophile. *p*-Xylylenediamine underwent double S_N_Ar substitution to produce compound **2x**, the core of which is found in CXCR4 antagonists.^10^ Finally, we were pleased that the commonly used bioconjugation probe (DBCO-NH_2_) reacted with **1a** to yield product **2aa** with the strained alkyne moiety intact.

The 2-amino *N*-(1-ethyoxyvinyl)pyridinium salt products are potentially valuable for medicinal chemistry as they are comprised of two common bioactive substructures–2-aminopyridines^1^ and *N*-quaternized pyridinium salts.^11^ However, given the newness of this chemical class, no bioactivity studies have been reported to date. In contrast, 2-aminopyridines in free base or protonated salt form (NR_3_-HX) are frequently produced as drug leads, prompting us to develop multiple conditions for removal of the *N*-(1-ethoxyvinyl) substituent. Using S_N_Ar product **2a** as a test substrate, we identified several conditions for acid promoted hydrolysis, yielding **5a** (Scheme 3). Neutralization with careful NaOH titration, or an aqueous NaHCO_3_ wash, produces the free base **6a** in high yield. We also found that simply dissolving **2a** in isopropanol, then heating the resulting solution in a sealed vial at 125 °C promotes a clean thermolysis of the *N*-(1-ethoxyvinyl) group, providing product **5a** as the corresponding TfOH-salt in excellent yield. Lastly, a tandem oxidative cleavage/*N*-oxidation to yield pyridine *N*-oxide **7a** can be achieved in good yield with peracetic acid, offering a new entry to this valuable pharmacophore.^12^

**Scheme 3:**
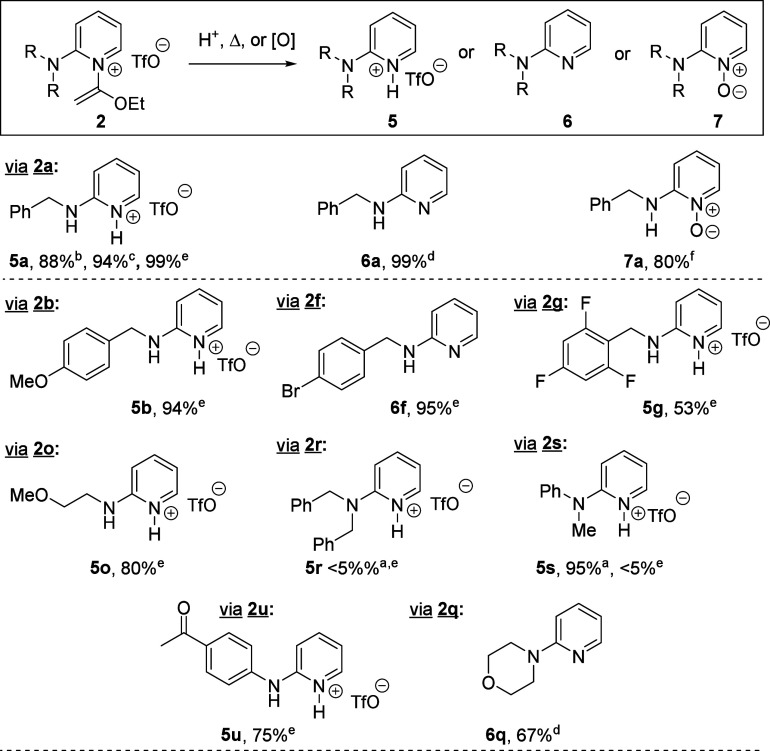
Thermal, Acidic, and Oxidative Cleavage of *N*-(1-Ethoxyvinyl) Group Acidic method: 3 M HCl in MeOH, rt. Acidic method: 3 M HCl in MeOH, 40 °C. Acidic method: 4 M HCl in 1,4-dioxane, 40 °C. Acidic method: conc. HCl; basic workup. Thermal method: iPrOH, 125 °C (sealed vial). Oxidative method: AcOOH, AcOH, rt.

We applied several of the above *N-*(1-ethoxyvinyl) cleavage protocols to a representative subset of S_N_Ar products. In general, thermally promoted alcoholysis in iPrOH worked well across multiple substrates, providing 2-aminopyridines as their corresponding TfOH salts in moderate to good yields (Scheme 3). The more hindered derivatives **2r**–**s** were less reactive, returning mostly the starting material under thermal cleavage conditions. However, **5s** was produced cleanly at room temperature in a 3 M methanolic HCl solution. Thus, while a good assortment of *N-*(1-ethoxyvinyl) cleavage protocols are available, the further development of methods with broader functional group compatibility is warranted.

To expand the scope of participating nucleophiles in this study, we examined several other S_N_Ar reactions and related transformations toward valuable heterocycles (Scheme 4). Using the same conditions developed for amine nucleophiles (condition a), we successfully employed a carbon nucleophile (dimethyl malonate) and 1-octanethiol as alternatives, yielding compounds **8** and **9**, respectively. On the other hand, weakly nucleophilic alcohols yielded mostly hydrolysis products with a range of different solvents and bases. Nevertheless, 2-alkoxypyridine product **10** could be produced in 40% yield at higher temperatures and in benzene (condition b). These same conditions were extended to use indole as a nucleophile, generating **11** in moderate yield.

**Scheme 4:**
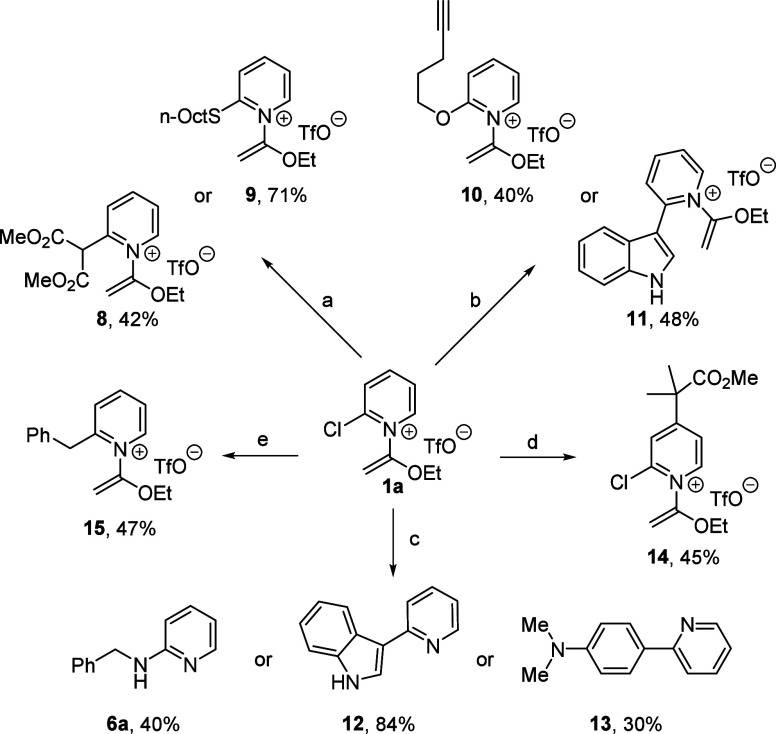
Additional Reactions of **1a** with Sulfur, Oxygen, and Carbon Nucleophiles Reaction conditions (all reactions use 1 equiv. **1a**): 1 equiv. nucleophile (1-octanethiol or dimethylmalonate), K_2_CO_3_, CH_2_Cl_2_, 40 °C. Reaction conditions (all reactions use 1 equiv. **1a**): 1 equiv. nucleophile (indole or 4-pentyn-1-ol), PhH, 80 °C. Reaction conditions (all reactions use 1 equiv. **1a**): 1 equiv. nucleophile (benzylamine, indole, or *N*,*N*-dimethylaniline), MgSO_4_, CF_3_CH_2_OH, 150 °C. 1 equiv. **1a**, 0.33 equiv. (CH_3_)_2_C=C(OCH_3_)OTMS, PhCF_3_, 150 °C. 1 equiv. **1a**, 1.5 equiv. PhCH_2_BF_3_K, 1 mol. % [(Ir[dF(CF3)ppy]_2_(dtbpy)]PF_6_, 2 mol. % Ni(COD)_2_, 2 mol. % dtbpy, blue LED, THF, rt.

We were also eager to explore conditions that enabled a nucleophilic substitution, followed by immediate cleavage of the *N*-(1-ethoxyvinyl) moiety. Toward that end, we discovered that fluorinated solvents (PhCF_3_, HFIP, or CF_3_CH_2_OH) and elevated temperatures enables a S_N_Ar and *N*-(1-ethoxyvinyl) group hydrolysis cascade to produce 2-aminopyridine **6a** in one pot (condition c, Scheme 4). Likewise “deprotected” 3-(pyridin-2-yl)indole **12** and heterobiaryl product **13**, derived from indole or *N*,*N*-dimethylaniline, respectively, could also be generated using this higher temperature protocol. New and unused glassware and stir bars were used to diminish the likelihood of trace-metal catalysis,^13^ and addition of a few common metals (1 mol.% of FeCl_3_, NiCl_2_, CuCl_2_)^14^ were tolerated, but did not improve the yields of **6a**, **12**, and **13**. Though not required, the addition of magnesium sulfate to the reaction mixture slightly improved yields. However, further analysis is necessary to determine whether trace metal contamination plays a significant role in these transformations.

Lastly, we discovered that the trimethylsilyl ketene acetal derived from methyl isobutyrate adds to the C4 position of **1a**, suggesting a blocking group effect by the Cl-atom under these conditions.^15^ Following air oxidation in situ,^16^ C4-alkylation product **14** was isolated. Several attempted Grignard reagent additions (e.g., PhCH_2_MgBr) gave complex mixtures; however, we developed a photochemical coupling approach inspired by Hong’s protocol^17^ to C2-benzylated product **15**, a pyridinium salt that cannot be made efficiently via previous approaches.^6c^

In summary, 2-halopyridinium ketene hemiaminals, particularly reagent **1a**, have been developed as new reagents for the mild synthesis of a variety of 2-aminopyridines and 2-aminopyridinium salts. Preliminary results using **1a** with other nucleophiles reveal multiple promising directions toward the synthesis of other bioactive heterocycles. We plan to study each of these reactions in more detail and report the optimized procedures in due course.

## Data Availability

The data underlying this study are available in the published article and its Supporting Information.

## Supporting Information Available

Experimental procedures and spectral data (PDF)
