# Supplementary material for: Bench-Stable 2-Halopyridinium Ketene Hemiaminals as Reagents for the Synthesis of 2-Aminopyridine Derivatives
Source: Org Lett. 2024 Sep 20;26(46):9805–10. doi: 10.1021/acs.orglett.4c02915 (PMC11590095; doi:10.1021/acs.orglett.4c02915)
Supplement: Supplementary file 1 — ol4c02915_si_001.pdf [file ol4c02915_si_001.pdf]

Supporting Information

Bench-stable 2-halopyridinium ketene hemiaminals as reagents for the synthesis of 2-aminopyridine derivatives

Isabelle C. Bote, Zoe A. Krevlin, Maria Christina F. Crespo, Sudchananya Udomphan, Carolyn T. Levin, Christie C. Lam, Amy M. Glanzer, Holly L. Hutchinson, Alisha M. Blades, Danielle L. McConnell, Crystal Lin, John P. Frank, William R. Strutton, Jordan C. Merklin, Beau A. Sinardo, Khady J. Gueye, Karly V. Leiman, Ashley Thayaparan, Joel K. A. Adade, Nestor L. Martinez, Wesley W. Kramer, Max M. Majireck*

Chemistry Department, Hamilton College, 198 College Hill Road, Clinton, NY, 13323, USA

*Correspondence: mmajirec@hamilton.edu; Tel.: +001-315-859-4742

Contents:

I. General Experimental……………………………………………………………………………………..…..……S2
II. General Synthetic Procedures…………...………………......…………..………………………..…..………......….S2-S5
III. Specific Procedures and Tabulated Spectral Data (in numerical order)..……………………………………….……..S5-S21
IV. Images of ^{1}H and ^{13}C{^{1}H} NMR spectra of new compounds (in numerical order)……......................................S22-S61
V. Representative qNMR spectra……………………………………………………………………………………..S61-S62
VI. Images of ^{1}H NMR spectra of known compounds prepared by current methods (in numerical order)….......…....S62-S66
VII. References……....………………………………………………………………………...…………......S67

I. General Experimental:

All commercially available chemicals were used as obtained, without further purification. Conventional heating was performed with a silicon oil bath and IKA RCT basic stir plate equipped with a temperature probe. Microwave heating was performed in a fourth generation Biotage Initiator+ microwave reactor using sealed 0.5-2 mL or 2-5 mL Biotage reaction vials. NMR Spectra were obtained on a Bruker Avance 500 MHz spectrometer. Both nominal and high-resolution mass spectra were obtained on a Waters Micromass 70-VSE using electrospray ionization (ESI). For mass spectrometry of all pyridinium salts, only the pyridinium cation was detected and analyzed by mass spectrometry due to weak coordination by the triflate counteranion.

Column chromatography was performed using new RediSep Rf Gold normal phase silica columns (20–40 micron) with a Teledyne Isco CombiFlash Rf200 purification system. We found automated chromatography to be most convenient, but not essential. Standard column chromatography may be employed, but high-grade silica is recommended (e.g., fine spherical silica, 20-40 μM) in order to avoid contamination of broken off silica in the product while using polar alcohol solvents (e.g., methanol or isopropanol). Multiple control experiments involving extensive washing of 4g and 12g RediSep Rf Gold normal phase silica columns with pure methanol or isopropanol showed that little (<1 mg) to no silica was eluted.

For compounds containing impurities or present as known mixtures, ^{1}H NMR integrations were used to calculate more accurate yields of the desired and/or side product(s). In several cases, significant amounts of unknown co-eluting impurities are noted and quantitative NMR analysis with an internal standard was attempted to get a better estimate of purity. For the majority of pyridinium salts, we found acetone-d_6 to be an effective solvent for NMR analysis, with CD_3CN and CD_3OD as alternatives whenever poor solubility and/or cross reactivity was observed. In most cases formation of side product formation resulting from carbonyl/amine side reactions was not detected when using acetone-d_6 to obtain a standard length ^{1}H or ^{13}C NMR (~2-60 min), but long-term storage in acetone generally leads to product degradation. In previous studies, DMSO-d_6 was found to promote slow degradation of 2-halopyridinium salts, and poor solubility with consequent lower quality spectra was observed when using $CDCl_3$.

II. General Synthetic Procedures:

1. Preparation 2-halopyridinium ketene hemiaminal reagents

A) Synthesis of 1-(1-ethoxyvinyl)-2-chloropyridinium triflate (1a):

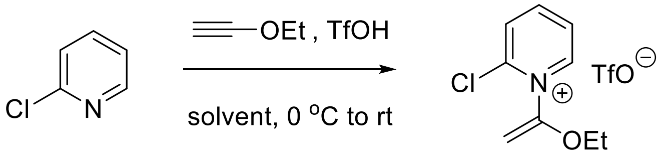

1a

1) Method 1 (1-5 mmol): For smaller quantities of reagent **1a**, the previously reported procedure[1] works well to deliver 1-5 mmol of product as an off-white solid in generally high yields (up to 99%) and high purity (>98%) via purification by column chromatography. Purification of larger quantities (>10 mmol) of reagent **1a** by column chromatography leads to greatly diminished yields due to incomplete elution of the highly polar product.

2) Method 2 (37 mmol, large scale using dichloromethane as solvent): For larger quantities of slightly lower purity than Method 1, a recently published *Organic Syntheses* protocol is available.[2] The product NMR and mass spectra matched those previously reported.[1,2] Nearly all reactions involving **1a** in this manuscript used this method.

3) Method 3 (37 mmol, large scale using toluene in place of dichloromethane): The following unoptimized procedure is provided as a "DCM-free" alternative due to the impending restriction of dichloromethane use: A flame-dried,

3-necked 250 mL round-bottom flask equipped with an addition funnel was charged with 2-chloropyridine (3.5 mL, 36.6 mmol), ethoxyacetylene solution (8.8 mL, 40 wt% in hexanes, 36.6 mmol), and anhydrous toluene (40 mL) and the resulting solution was cooled to 0 °C. Fresh triflic acid from a sealed glass ampule (5.0 g, 33.3 mmol) was added to the addition funnel, then delivered dropwise to the reaction mixture over approximately 10 minutes. The resulting dark brown solution was stirred overnight while gradually warming to room temperature, then the solvent was removed by rotary evaporation to provide a reddish brown solid. The crude product was dissolved in a minimum of boiling 1,2-dimethoxyethane, gradually cooled to room temperature, then placed in a -20 °C freezer overnight to form reddish-brown crystals. The mother liquor was filtered off and the crystalline solid was washed with cold diethyl ether to provide reagent **1a** as a reddish-brown solid (8.06g, ~84% purity with the major side product being the TfOH salt of 2-chloropyridine). Additional recrystallizations in 1,2-dimethoxyethane as in our previously published protocol[2] are recommended if greater purity is desired.

B) Synthesis of 1b-d and 1-(1-ethoxyvinyl)-2,3-dichloropyridinium triflate: Approximately 1 mmol quantities of each reagent were prepared according a previously reported procedure.[1] Note that 1-(1-ethoxyvinyl)-2-fluoropyridinium triflate (**1b**) is best prepared in situ (see section 2B below). All spectral data matched those previously reported.

2. General S_NAr Procedures

A) Method 1: Standard Amine/Malonate/Thiol S_NAr Procedure using Reagent 1a

- for the synthesis of products **2a-u**, **2w-aa**, **8**, and **9**

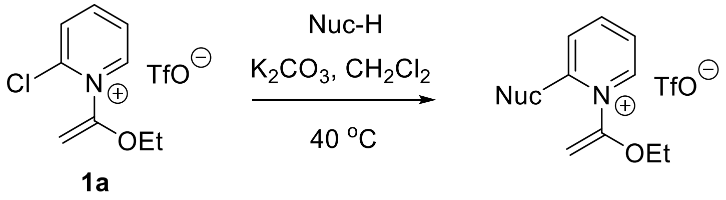

(Nuc-H = primary or secondary amine, thiol, or malonate)

A 0.5-2.0 mL Biotage microwave vial was charged with 1-(1-ethoxyvinyl)-2-chloropyridinium triflate **1a** (167 mg, 0.50 mmol), 1 mL of dichloromethane, nucleophile (0.50 mmol), and then anhydrous potassium carbonate (69 mg, 0.50 mmol). A mild exotherm was generally observed following addition of the amine or potassium carbonate, so the resulting suspension was stirred for one minute before sealing with a crimped septum cap and placing into a pre-heated 40 °C oil bath for 24h. Before uncapping the vial, the reaction was cooled to room temperature and then the septum was punctured with a needle to release pressure. The reaction mixture was then transferred by pipette to a round-bottom flask using acetone or methanol to transfer over any remaining organic soluble residue. The resulting solution was concentrated in vacuo to yield a residue that was purified by automated column chromatography with a Teledyne ISCO CombiFlash system (between 0-100% chloroform/isopropanol gradient; 4g RediSep Gold® pre-packed silica gel cartridge) to yield the corresponding *N*-(1-ethoxyvinyl) 2-aminopyridinium triflate product.

B) Method 2: Alternative Amine S_NAr Procedure via in situ Generation of Reagent 1b

- for synthesis of products **2a** and **2v**

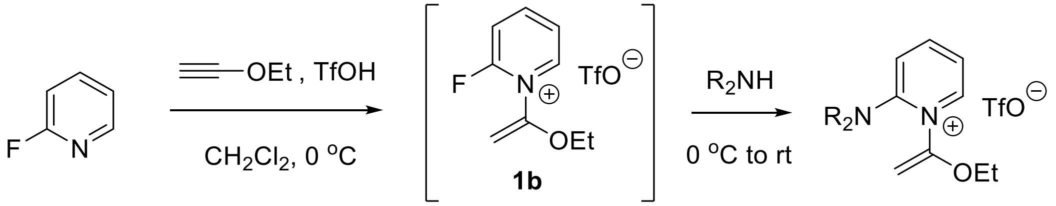

A flame-dried 5 mL round-bottomed flask under an argon atmosphere was charged with dichloromethane (1.5 mL), 2-fluoropyridine (90 μL, 1.0 mmol), and ethoxyacetylene (240 μL, 1.0 mmol, ~40 wt% in hexanes). The resulting solution was cooled to 0 °C in an ice-water bath and then fresh triflic acid (92 μL, 1.0 mmol) was added dropwise. After stirring for 1 hour to form 1-(1-ethoxyvinyl)-2-fluoropyridinium triflate **1b** in situ, amine nucleophile (1.0 mmol) was added, and the resulting mixture was stirred for 24h while gradually warming to room temperature. The resulting mixture was concentrated in vacuo to yield a residue that was purified by automated column chromatography with a Teledyne ISCO CombiFlash

system (between 0-100% chloroform/isopropanol gradient; 4g RediSep Gold® pre-packed silica gel cartridge) to yield the *N*-(1-ethoxyvinyl) 2-aminopyridinium triflate product.

C) Method 3: Tandem S_NAr and *N*-(1-Ethoxyvinyl) Cleavage Procedure using Amine/Indole/Aniline Nucleophiles and Microwave Heating

- for the synthesis of products **6a**, **12**, and **13**

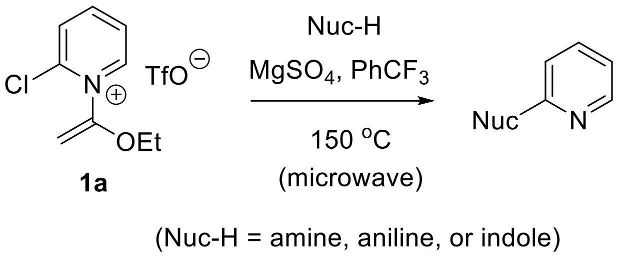

A flame-dried 0.5-2.0 mL Biotage microwave vial was charged with 1-(1-ethoxyvinyl)-2-chloropyridinium triflate **1a** (100 mg, 0.30 mmol), nucleophile (0.30 mmol), anhydrous magnesium sulfate powder (36 mg, 0.30 mmol) and 2,2,2-trifluoroethanol (1.0 mL). The vial was sealed with a crimped septum cap, then heated in a Biotage Initiator+ microwave reactor for 3h for at 150 ℃ using the “very high” absorption level setting. (**Caution!** Generally 10 bars of pressure are generated under these conditions. Although this is significantly below the temperature and pressure capacity of the Biotage Initiator+ system (~300 ℃, 30 bars), extra caution should be taken when heating a sealed reaction beyond the solvent’s boiling point.) Before uncapping the vial, the reaction was cooled to room temperature and then the septum was punctured with a needle to release pressure. The reaction mixture was then transferred to a round-bottom flask using acetone and concentrated in vacuo to produce a residue that was dissolved in 50 mL of EtOAc and transferred to a separatory funnel. The ethyl acetate solution was washed three times with an aqueous solution of sodium bicarbonate (saturated), dried with sodium sulfate, and concentrated in vacuo to produce a residue that was purified by automated column chromatography with a Teledyne ISCO CombiFlash system **(**between 0-100% hexanes/ethyl acetate gradient; 4g RediSep Gold® pre-packed silica gel cartridge) to yield the product.

3. General *N*-(1-Ethoxyvinyl) Cleavage Procedures

A) Method 1 (Thermolytic):

- For synthesis of **5a**, **5b**, **6f**, **5g**, **5o**, and **5u**.

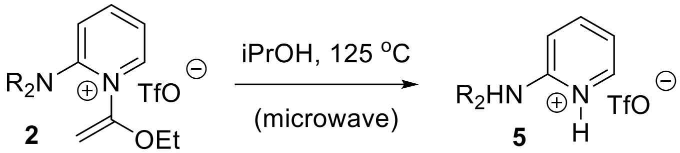

The corresponding *N*-(1-ethoxyvinyl) 2-aminopyridinium triflate **2** (generally between 0.1-0.3 mmol) was dissolved in isopropanol (1 mL) in a 0.5-2.0 mL Biotage microwave vial. The vial was sealed with a crimped septum cap, then placed into a pre-heated 125 ℃ oil bath for 17 h. Before uncapping the vial, the reaction was cooled to room temperature and then the septum was punctured with a needle to release pressure. The reaction mixture was then transferred to a round-bottom flask using additional isopropanol, then concentrated in vacuo to remove the solvent and volatile byproducts and yield pyridinium salt **5**.

B) Method 2 (Acidic):

- For synthesis of **5a** and **5s**

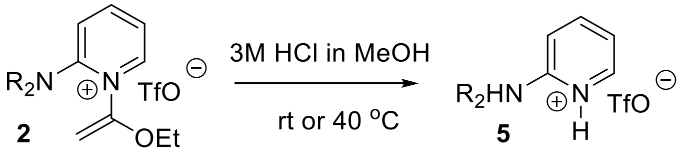

The corresponding *N*-(1-ethoxyvinyl) 2-aminopyridinium triflate **2** was dissolved in 3M HCl in methanol (1 mL) in a 0.5-2.0 mL Biotage microwave vial. The vial was sealed with a crimped septum cap, then stirred at the indicated temperature (room temperature or in a pre-heated 40 °C oil bath) for 17 h. Before uncapping the vial, the septum was punctured with a needle to release pressure. The reaction mixture was then transferred to a round-bottom flask using additional methanol, then concentrated in vacuo to remove the solvent and volatile byproducts and yield pyridinium salt **5**.

4. Modifications / Other Procedures: For products synthesized by significant modification to the above general procedures, or by unique protocols, clarifying notes and/or full experimental procedures are provided in Section III.

III. Specific Procedures and Tabulated Spectral Data

2-(benzylamino)-1-(1-ethoxyvinyl)pyridin-1-ium trifluoromethanesulfonate (**2a**)

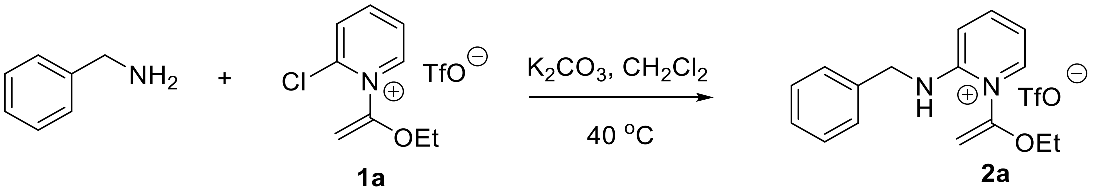

1. General Amine S_NAr Procedure 2A (Method 1) was followed using benzylamine (55 µL, 0.50 mmol) as the amine nucleophile. Purification by automated column chromatography with a Teledyne ISCO CombiFlash system (0-70% chloroform/isopropanol gradient) yielded product **2a** as a pale yellow amorphous solid (198 mg, 98%). Spectral data of product **2a** matched those previously reported.[1] Following the same procedure at double the scale (1.0 mmol of all reactants in 2 mL of CH_2Cl_2) led to similar a yield of **2a** (380 mg, 94%).

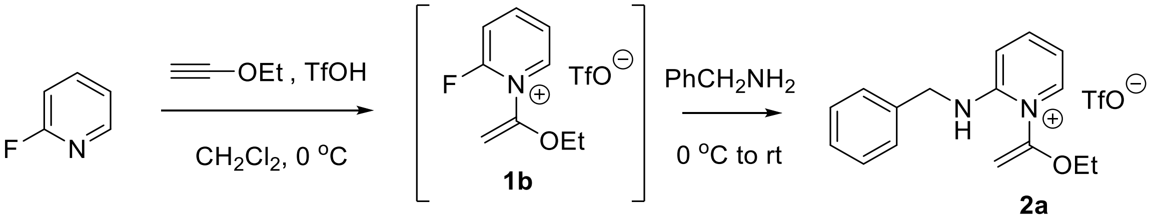

2. Alternative Amine S_NAr Procedure 2B (Method 2) was followed using benzylamine (110 µL, 1.0 mmol) as the amine nucleophile. Purification by automated column chromatography with a Teledyne ISCO CombiFlash system (0-70% chloroform/isopropanol gradient) yielded product **2a** as a pale yellow amorphous solid (168 mg, 83%). Spectral data of product **2a** matched those previously reported.[1]

1-(1-ethoxyvinyl)-2-((4-methoxybenzyl)amino)pyridin-1-ium trifluoromethanesulfonate (**2b**)

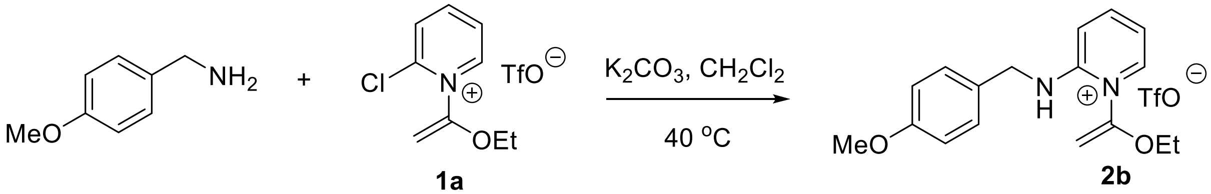

General Amine S_NAr Procedure 2A (Method 1) was followed using 4-methoxybenzylamine (65 µL, 0.50 mmol) as the amine nucleophile. Purification by automated column chromatography with a Teledyne ISCO CombiFlash system (0-70% chloroform/isopropanol gradient) yielded product **2b** pale yellow amorphous solid (206 mg, 95%). Spectral data of product **2b** matched those previously reported.[1]

1-(1-ethoxyvinyl)-2-((4-hydroxybenzyl)amino)pyridin-1-ium trifluoromethanesulfonate (**2c**)

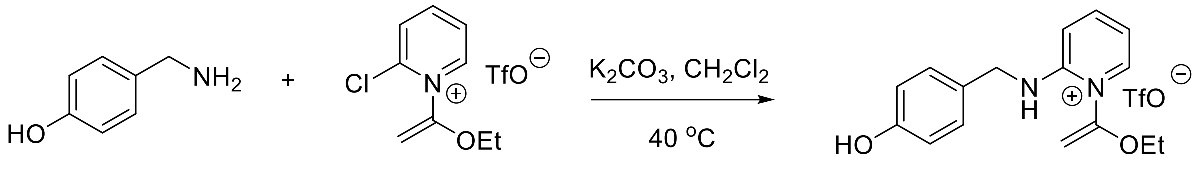

General Amine S_NAr Procedure 2A (Method 1) was followed using 4-hydroxybenzylamine (62 mg, 0.50 mmol) as the amine nucleophile. Purification by automated column chromatography with a Teledyne ISCO CombiFlash system (0-100% chloroform/isopropanol gradient) yielded product **2c** as a tan amorphous solid (149 mg @ ~87% purity, 62%). ^{1}H NMR (500 MHz, Acetone-d_6 δ 8.57 (br s, 2H), 8.15 (dd, *J* = 6.8, 1.6 Hz, 1H), 8.10 (ddd, *J* = 9.0, 7.0, 1.7 Hz, 1H), 7.31 (t, *J* = 9.3 Hz, 3H), 7.07 (td, *J* = 6.9, 1.1 Hz, 1H), 6.88 – 6.81 (m, 2H), 4.92 (d, *J* = 4.4 Hz, 1H), 4.89 (d, *J* = 4.4 Hz, 1H), 4.72 (d, *J* = 5.5 Hz, 2H), 4.22 (q, *J* = 7.0 Hz, 2H), 1.39 (t, *J* = 7.0 Hz, 3H); ^{13}C NMR (126 MHz, Acetone-d_6 δ 157.3, 152.4, 151.2, 145.0, 140.2, 128.6, 126.7, 121.2 (q, $^1J_{CF}$ = 321 Hz, *C*F$_3$), 115.6, 112.9, 112.2, 87.2, 66.4, 45.5, 13.3; LRMS-ES+ *m/z* (relative intensity) 271.1 ($C_{16}H_{19}N_2O_2$ M+, 100); HRMS-ES+ ($C_{16}H_{19}N_2O_2$) calcd 271.1447 (M+), found 271.1450.

1-(1-ethoxyvinyl)-2-((4-((trifluoromethyl)thio)benzyl)amino)pyridin-1-ium trifluoromethanesulfonate (**2d**)

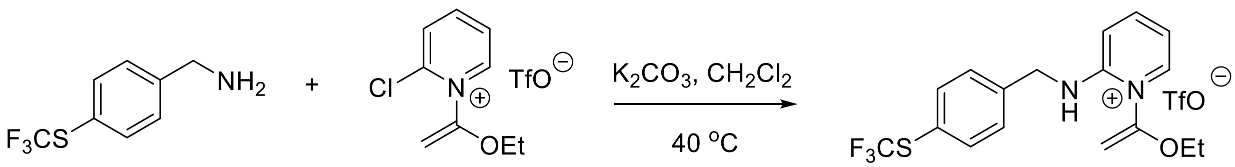

General Amine S_NAr Procedure 2A (Method 1) was followed using 4-(trifluoromethylthio)benzylamine (71 μL, 0.50 mmol) as the amine nucleophile. Purification by automated column chromatography with a Teledyne ISCO CombiFlash system (0-70% chloroform/isopropanol gradient) yielded product **2d** as a pale yellow amorphous solid (189 mg, 75%). ^{1}H NMR (500 MHz, Acetone-d_6) δ 8.72 (s, 1H), 8.21 (dd, *J* = 6.7, 1.7 Hz, 1H), 8.13 (ddd, *J* = 9.0, 7.1, 1.7 Hz, 1H), 7.72 (d, *J* = 8.3 Hz, 2H), 7.65 (d, *J* = 8.3 Hz, 2H), 7.34 (d, *J* = 9.3 Hz, 1H), 7.13 (td, *J* = 7.0, 1.2 Hz, 1H), 4.98 (d, *J* = 4.5 Hz, 1H), 4.96 (s, 2H), 4.91 (d, *J* = 4.5 Hz, 1H), 4.24 (q, *J* = 7.0 Hz, 2H), 1.40 (t, *J* = 7.0 Hz, 3H); ^{13}C NMR (126 MHz, Acetone-d_6) δ 153.3, 152.0, 146.5, 141.3, 137.5, 131.7, 131.2, 130.7 (q, $^1J_{CF}$ = 307 Hz, S*C*F$_3$), 129.5, 122.0 (q, $^1J_{CF}$ = 322 Hz, O*C*F$_3$), 114.3, 112.8, 88.1, 67.4, 46.0, 14.2; LRMS-ES+ *m/z* (relative intensity) 355.1 ($C_{17}H_{18}F_3N_2OS$ M+, 100); HRMS-ES+ ($C_{17}H_{18}F_3N_2OS$) calcd 355.1092 (M+), found 355.1097.

2-((4-(diethylamino)benzyl)amino)-1-(1-ethoxyvinyl)pyridin-1-ium trifluoromethanesulfonate (**2e**)

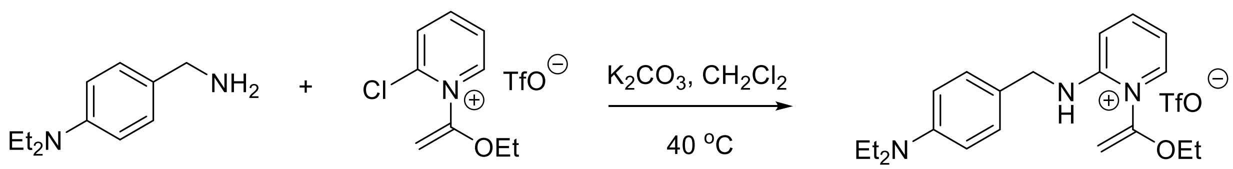

General Amine S_NAr Procedure 2A (Method 1) was followed using 4-(diethylamino)benzylamine (89 mg, 0.50 mmol) as the amine nucleophile. Purification by automated column chromatography with a Teledyne ISCO CombiFlash system (0-100% chloroform/isopropanol gradient) yielded product **2e** as a pale yellow amorphous solid (140 mg, 59%). ^{1}H NMR (500 MHz, Acetone-d_6) δ 8.53 (s, 1H), 8.16 – 8.04 (m, 2H), 7.35 (d, *J* = 9.4 Hz, 1H), 7.28 (d, *J* = 8.3 Hz, 2H), 7.05 (t, *J* = 6.9 Hz, 1H), 6.68 (d, *J* = 8.6 Hz, 2H), 4.89 (q, *J* = 4.5 Hz, 2H), 4.68 (s, 2H), 4.22 (q, *J* = 5.4 Hz, 2H), 3.37 (q, *J* = 7.0 Hz, 4H), 1.40 (t, *J* = 7.0 Hz, 3H), 1.12 (app t, *J* = 6.8 Hz, 6H); ^{13}C NMR (126 MHz, Acetone-d_6) δ 152.3, 151.2, 144.9,

141.7, 140.1, 131.4, 128.6, 121.2 (q, $^1J_{CF}$ = 321 Hz, CF_3), 112.8, 112.2, 112.2, 87.1, 66.4, 49.8, 45.6, 13.4, 11.9; LRMS-ES+ *m/z* (relative intensity) 326.2 ($C_{20}H_{28}N_3O$ M+, 30); HRMS-ES+ ($C_{20}H_{28}N_3O$) calcd 326.2232 (M+), found 326.2231.

2-((4-bromobenzyl)amino)-1-(1-ethoxyvinyl)pyridin-1-ium trifluoromethanesulfonate (**2f**)

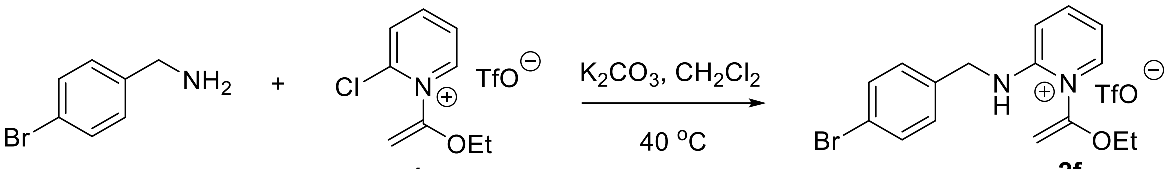

General Amine S_NAr Procedure 2A (Method 1) was followed using 4-bromobenzylamine (93 mg, 0.50 mmol) as the amine nucleophile. Purification by automated column chromatography with a Teledyne ISCO CombiFlash system (0-70% chloroform/isopropanol gradient) yielded product **2f** as a tan amorphous solid (210 mg, 87%). ^{1}H NMR (500 MHz, Acetone-d$_6$) δ 8.66 (br s, 1H), 8.22 (d, *J* = 6.5 Hz, 1H), 8.14 (t, *J* = 8.3 Hz, 1H), 7.56 (d, *J* = 6.5 Hz, 2H), 7.44 (d, *J* = 7.8 Hz, 2H), 7.33 (d, *J* = 9.1 Hz, 1H), 7.14 (t, *J* = 6.8 Hz, 1H), 4.99 (d, *J* = 3.6 Hz, 1H), 4.92 (d, *J* = 3.6 Hz, 1H), 4.85 (s, 2H), 4.23 (q, *J* = 7.3 Hz, 2H), 1.40 (t, *J* = 7.3 Hz, 3H); ^{13}C NMR (126 MHz, Acetone-d$_6$) δ 152.4, 151.1, 145.4, 140.4, 136.0, 131.8, 129.4, 121.3 (q, $^1J_{CF}$ = 323 Hz, CF_3), 121.1, 113.3, 112.0, 87.2, 66.5, 45.2, 13.4; LRMS-ES+ *m/z* (relative intensity) 333.1 ($C_{16}H_{18}BrN_2O$ M+, 100 (Br-79 isotope)), 335.1 ($C_{16}H_{18}BrN_2O$ M+, 98 (Br-81 isotope)); HRMS-ES+ ($C_{16}H_{18}BrN_2O$) calcd 333.0603 (M+), found 333.0603.

1-(1-ethoxyvinyl)-2-((2,4,6-trifluorobenzyl)amino)pyridin-1-ium trifluoromethanesulfonate (**2g**)

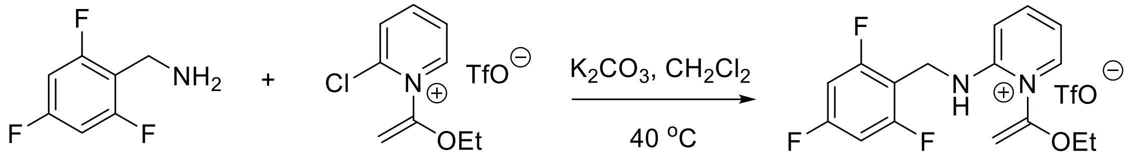

General Amine S_NAr Procedure 2A (Method 1) was followed using 2,4,6-trifluorobenzylamine (81 mg, 0.50 mmol) as the amine nucleophile. Purification by automated column chromatography with a Teledyne ISCO CombiFlash system (0-70% chloroform/isopropanol gradient) yielded product **2g** as a pale yellow amorphous solid (206 mg, 90%). ^{1}H NMR (500 MHz, Acetone-d$_6$) δ 8.39 (s, 1H), 8.28 (t, *J* = 8.3 Hz, 1H), 8.22 (d, *J* = 6.6 Hz, 1H), 7.52 – 7.43 (m, 1H), 7.18 (dt, *J* = 7.2, 4.2 Hz, 1H), 7.03 (dt, *J* = 8.9, 4.7 Hz, 2H), 4.96 – 4.85 (m, 4H), 4.14 (q, *J* = 7.6 Hz, 2H), 1.34 (t, *J* = 5.8 Hz, 3H^{13}C NMR (126 MHz, Acetone-d$_6$) δ 162.8 (dt, *J* = 249, 16.1 Hz, C4-F coupling on benzene ring); 161.9 (ddd, *J* = 249.8, 15.3, 10.6 Hz, C2-F coupling on benzene ring); 152.1, 151.1, 145.7, 140.6, 121.4 (q, $^1J_{CF}$ = 323 Hz, CF_3), 113.5, 111.4, 108.50 (td, *J* = 18.9, 4.9 Hz, C1-F coupling on benzene ring), 100.9-100.4 (m, C3-F coupling on benzene ring), 87.3, 66.4, 35.0 (t, $^3J_{CF}$ = 3.8 Hz, benzyl CH_2), 13.2; LRMS-ES+ *m/z* (relative intensity) 309.1 ($C_{16}H_{16}F_3N_2O$ M+, 100); HRMS-ES+ ($C_{16}H_{16}F_3N_2O$) calcd 309.1215 (M+), found 309.1215.

2-((3-chloro-4-methoxybenzyl)amino)-1-(1-ethoxyvinyl)pyridin-1-ium trifluoromethanesulfonate (**2h**)

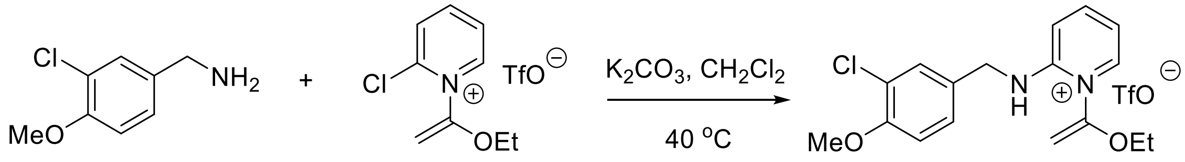

General Amine S_NAr Procedure 2A (Method 1) was followed using 3-chloro-4-methoxybenzylamine (86 mg, 0.50 mmol) as the amine nucleophile. Purification by automated column chromatography with a Teledyne ISCO CombiFlash system (0-70% chloroform/isopropanol gradient) yielded product **2h** as an off-white amorphous solid (199 mg, 85%). ^{1}H NMR (500 MHz, Acetone-d$_6$) δ 8.49 (s, 1H), 8.03 (ddd, *J* = 6.7, 1.7, 0.7 Hz, 1H), 7.98 (ddd, *J* = 9.0, 7.1, 1.6 Hz, 1H), 7.38

(d, J = 2.2 Hz, 1H), 7.29 (dd, J = 8.5, 2.2 Hz, 1H), 7.22 (dt, J = 9.2, 1.0 Hz, 1H), 6.96 (dd, J = 7.6, 6.4 Hz, 2H), 4.80 (d, J = 4.4 Hz, 1H), 4.75 (d, J = 4.4 Hz, 1H), 4.65 (d, J = 3.1 Hz, 2H), 4.10 (q, J = 7.0 Hz, 2H), 3.74 (s, 3H), 1.27 (t, J = 7.0 Hz, 3H); ^{13}C NMR (126 MHz, $CDCl_3$) δ 155.2, 152.6, 151.2, 145.0, 139.9, 129.3, 129.2, 127.4, 123.3, 121.0 (q, $^1J_{CF}$ = 320 Hz, *C*F_3), 113.2, 112.9, 112.4, 88.0, 67.3, 56.6, 45.7, 14.3; LRMS-ES+ *m/z* (relative intensity) 319.1 ($C_{17}H_{20}ClN_2O_2$ M+, 100 (Cl-35 isotope)), 321.1 ($C_{17}H_{20}ClN_2O_2$ M+, 30 (Cl-37 isotope)); HRMS-ES+ ($C_{17}H_{20}ClN_2O_2$) calcd 319.1213 (M+), found 319.1215.

2-((2,4-dimethoxybenzyl)amino)-1-(1-ethoxyvinyl)pyridin-1-ium trifluoromethanesulfonate (**2i**)

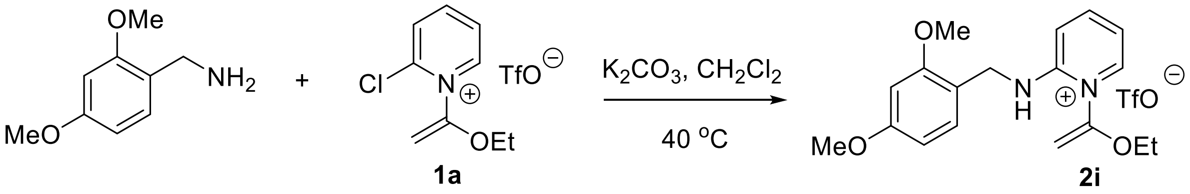

General Amine S_NAr Procedure 2A (Method 1) was followed using 2,4-dimethoxybenzylamine (75 μL, 0.50 mmol) as the amine nucleophile. Purification by automated column chromatography with a Teledyne ISCO CombiFlash system (0-70% chloroform/isopropanol gradient) yielded product **2i** as a pale yellow amorphous solid (228 mg, 98%). ^{1}H NMR (500 MHz, Acetone-d$_6$) δ 8.17 (br t, J = 6.3 Hz, 1H), 8.00 (td, J = 7.0, 1.5 Hz, 2H), 7.23 (d, J = 9.7 Hz, 1H), 7.15 (d, J = 8.4 Hz, 1H), 6.94 (td, J = 6.9, 1.1 Hz, 1H), 6.48 (d, J = 2.4 Hz, 1H), 6.37 (dd, J = 8.4, 2.4 Hz, 1H), 4.76 (q, J = 4.5 Hz, 2H), 4.56 (d, J = 6.2 Hz, 2H), 4.06 (q, J = 7.0 Hz, 2H), 3.76 (s, 3H), 3.66 (s, 3H), 1.24 (t, J = 7.0 Hz, 3H); ^{13}C NMR (126 MHz, Acetone-d$_6$) δ 161.2, 158.5, 152.3, 151.2, 145.0, 140.0, 129.4, 121.5 (q, $^1J_{CF}$ = 323 Hz, *C*F_3),115.7, 112.8, 112.1, 104.8, 98.4, 87.1, 66.4, 55.1, 54.8, 41.5, 13.3; LRMS-ES+ *m/z* (relative intensity) 315.2 ($C_{18}H_{23}N_2O_3$ M+, 100); HRMS-ES+ ($C_{18}H_{23}N_2O_3$) calcd 315.1709 (M+), found 315.1715.

(*S*)-1-(1-ethoxyvinyl)-2-((1-phenylethyl)amino)pyridin-1-ium trifluoromethanesulfonate (**2j**)

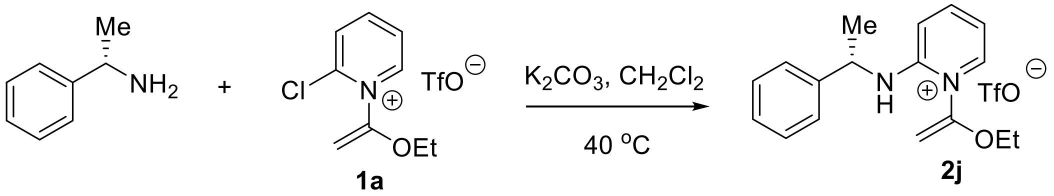

General Amine S_NAr Procedure 2A (Method 1) was followed using (*S*)-(−)-α-Methylbenzylamine (64 μL, 0.50 mmol) as the amine nucleophile. Purification by automated column chromatography with a Teledyne ISCO CombiFlash system (0-70% chloroform/isopropanol gradient) yielded product **2j** as an off-white amorphous solid (121 mg, 58%). ^{1}H NMR (500 MHz, CD_3CN) δ 7.84 – 7.77 (m, 2H), 7.31 (td, J = 8.4, 6.3 Hz, 4H), 7.25 – 7.19 (m, 1H), 7.07 – 6.98 (m, 1H), 6.91 (d, J = 9.2 Hz, 1H), 6.85 (td, J = 6.9, 1.2 Hz, 1H), 4.88 (p, J = 6.8 Hz, 1H), 4.75 – 4.68 (m, 2H), 4.15 – 4.07 (m, 2H), 1.57 (d, J = 6.9 Hz, 3H), 1.30 (t, J = 7.0 Hz, 3H); ^{13}C NMR (126 MHz, CD_3CN) δ 152.0, 151.3, 145.8, 142.2, 140.7, 129.6, 128.6, 126.5, 121.7 (q, $^1J_{CF}$ = 321 Hz, *C*F_3), 114.0, 112.9, 88.2, 67.2, 54.5, 23.0, 13.8; LRMS-ES+ *m/z* (relative intensity) 269.2 ($C_{17}H_{21}N_2O$ M+, 100); HRMS-ES+ ($C_{17}H_{21}N_2O$) calcd 269.1654 (M+), found 269.1655.

1-(1-ethoxyvinyl)-2-((2-(p-tolyl)propan-2-yl)amino)pyridin-1-ium trifluoromethanesulfonate (**2k**)

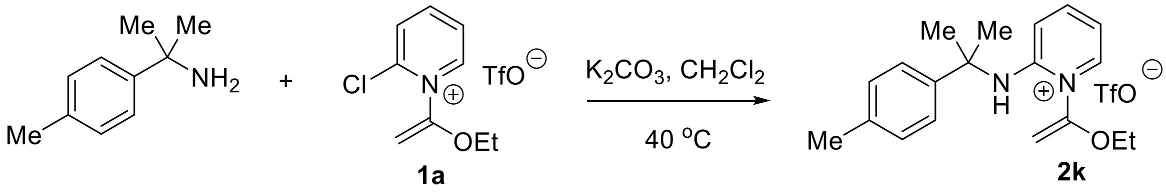

General Amine S_NAr Procedure 2A (Method 1) was followed using 2-(4-methylphenyl)-2-propanamine (75 mg, 0.50 mmol) as the amine nucleophile. Purification by automated column chromatography with a Teledyne ISCO CombiFlash system (0-70% chloroform/isopropanol gradient) yielded product **2k** as a pale yellow amorphous solid (150 mg, 67%). NMR (500 MHz, Acetone-d$_6$) δ 8.11 (dd, J = 6.7, 1.7 Hz, 1H), 7.78 (ddd, J = 9.1, 7.1, 1.7 Hz, 1H), 7.31 (s, 1H),

7.29 – 7.25 (m, 2H), 7.09 (d, J = 8.1 Hz, 2H), 6.96 (td, J = 6.9, 1.2 Hz, 1H), 6.54 (dt, J = 9.3, 1.0 Hz, 1H), 4.94 (d, J = 4.6 Hz, 1H), 4.86 (d, J = 4.7 Hz, 1H), 4.16 (q, J = 7.0 Hz, 2H), 2.18 (s, 3H), 1.72 (s, 6H), 1.33 (t, J = 7.0 Hz, 3H); ^{13}C NMR (126 MHz, Acetone-d$_6$) δ 151.0, 150.9, 144.4, 141.00, 140.95, 137.3, 129.8, 125.0, 118.3 (q, $^1J_{CF}$ = 323 Hz, CF$_3$), 113.9, 113.6, 88.0, 66.6, 59.0, 24.9, 20.1, 13.4; LRMS-ES+ m/z (relative intensity) 297.2 ($C_{19}H_{25}N_2O$ M+, 100); HRMS-ES+ ($C_{19}H_{25}N_2O$) calcd 297.1967 (M+), found 297.1969.

2-((benzo[*b*]thiophen-5-ylmethyl)amino)-1-(1-ethoxyvinyl)pyridin-1-ium trifluoromethanesulfonate (**2l**)

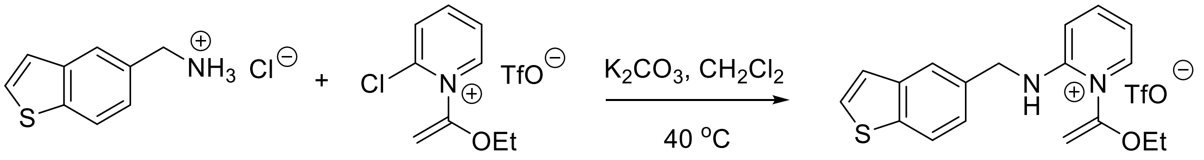

1a **2l**

General Amine S$_N$Ar Procedure 2A (Method 1) was followed using (1-benzothien-5-ylmethyl)amine hydrochloride (100 mg, 0.50 mmol) as the amine nucleophile and an extra equivalent of potassium carbonate (138 mg, 1.0 mmol). Purification by automated column chromatography with a Teledyne ISCO CombiFlash system (0-70% chloroform/isopropanol gradient) yielded product **2l** as a pale yellow amorphous solid (62 mg, 27%). ^{1}H NMR (500 MHz, CD$_3$OD) δ 8.03 (dd, J = 6.7, 1.9 Hz, 1H), 7.97 – 7.82 (m, 4H), 7.59 (dd, J = 5.3, 1.5 Hz, 1H), 7.39 – 7.32 (m, 2H), 7.17 (d, J = 9.3 Hz, 1H), 6.94 (td, J = 6.9, 1.3 Hz, 1H), 4.87 – 4.82 (m, 4H), 4.82 (d, J = 1.4 Hz, 5H), 4.22 (q, J = 7.0 Hz, 2H), 1.45 (t, J = 7.0 Hz, 3H); ^{13}C NMR (126 MHz, CD$_3$OD) δ 152.4, 151.5, 144.7, 140.2, 140.0, 139.3, 132.2, 127.4, 123.4, 122.75, 122.74, 121.4, 120.5 (q, $^1J_{CF}$ = 319 Hz, CF$_3$), 112.7, 111.7, 86.4, 66.3, 45.7, 13.0; LRMS-ES+ m/z (relative intensity) 311.1 ($C_{18}H_{19}N_2OS$ M+, 100); HRMS-ES+ ($C_{18}H_{19}N_2OS$) calcd 311.1218 (M+), found 311.1216.

1-(1-ethoxyvinyl)-2-((pyridin-3-ylmethyl)amino)pyridin-1-ium trifluoromethanesulfonate (**2m**)

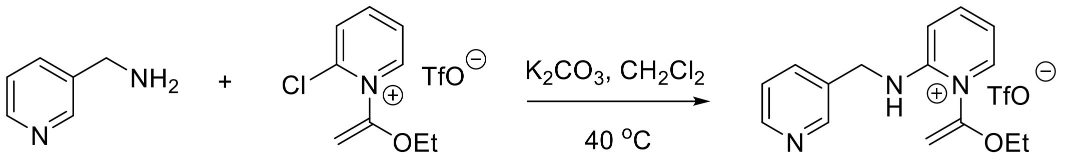

1a **2m**

General Amine S$_N$Ar Procedure 2A (Method 1) was followed using 3-picolylamine (51 µL, 0.50 mmol) as the amine nucleophile. Purification by automated column chromatography with a Teledyne ISCO CombiFlash system (0-100% chloroform/isopropanol gradient) yielded product **2m** as an off-white amorphous solid (89 mg, 44%). ^{1}H NMR (500 MHz, Acetone-d$_6$) δ 8.57 (d, J = 2.4 Hz, 1H), 8.39 (dd, J = 4.7, 1.6 Hz, 1H), 8.08 (ddd, J = 6.6, 1.7, 0.7 Hz, 1H), 8.03 (ddd, J = 9.0, 7.1, 1.7 Hz, 1H), 7.77 – 7.72 (m, 1H), 7.28 (dt, J = 9.3, 0.9 Hz, 1H), 7.24 (ddd, J = 7.9, 4.7, 0.9 Hz, 1H), 7.01 (td, J = 6.9, 1.1 Hz, 1H), 4.86 (d, J = 4.5 Hz, 1H), 4.80 (s, 2H), 4.78 (d, J = 4.5 Hz, 1H), 4.09 (q, J = 7.0 Hz, 2H), 1.26 (t, J = 7.0 Hz, 3H); ^{13}C NMR (126 MHz, Acetone-d$_6$) δ 152.4, 151.2, 149.2, 148.8, 145.6, 140.5, 134.8, 132.1, 123.6, 121.3 (q, $^1J_{CF}$ = 322 Hz, CF$_3$), 113.4, 111.9, 87.3, 66.5, 43.5, 13.3; LRMS-ES+ m/z (relative intensity) 256.1 ($C_{15}H_{18}N_3O$ M+, 100); HRMS-ES+ ($C_{15}H_{18}N_3O$) calcd 256.1450 (M+), found 256.1450.

1-(1-ethoxyvinyl)-2-(phenethylamino)pyridin-1-ium trifluoromethanesulfonate (**2n**)

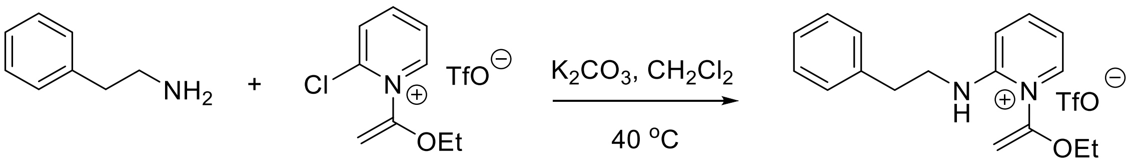

1a **2n**

General Amine S$_N$Ar Procedure 2A (Method 1) was followed using phenethylamine (63 µL, 0.50 mmol) as the amine nucleophile. Purification by automated column chromatography with a Teledyne ISCO CombiFlash system (0-70%

chloroform/isopropanol gradient) yielded product **2n** as a pale yellow residue (117 mg, 56%). ^{1}H NMR (500 MHz, CD_3OD) δ 7.94 – 7.88 (m, 2H), 7.32 – 7.24 (m, 4H), 7.21 (ddd, J = 8.6, 5.6, 2.3 Hz, 1H), 7.11 (d, J = 9.2 Hz, 1H), 6.89 (td, J = 6.9, 1.1 Hz, 1H), 4.75 (d, J = 4.5 Hz, 1H), 4.63 (d, J = 4.4 Hz, 1H), 4.12 (q, J = 7.0 Hz, 2H), 3.78 (t, J = 6.9 Hz, 2H), 2.98 (t, J = 6.9 Hz, 2H), 1.38 (t, J = 7.0 Hz, 3H), the N-H proton was not observed due to rapid hydrogen-deuterium exchange in CD_3OD; ^{13}C NMR (126 MHz, CD_3OD) δ 152.2, 151.2, 144.2, 139.5, 137.8, 128.9, 128.4, 126.5, 120.5 (q, $^{1}J_{CF}$ = 319 Hz, CF_3), 112.1, 111.3, 86.3, 66.2, 43.9, 34.5, 12.9; LRMS-ES+ *m/z* (relative intensity) 269.2 ($C_{17}H_{21}N_2O$ M+, 100); HRMS-ES+ ($C_{17}H_{21}N_2O$) calcd 269.1654 (M+), found 269.1657.

1-(1-ethoxyvinyl)-2-((2-methoxyethyl)amino)pyridin-1-ium trifluoromethanesulfonate (**2o**)

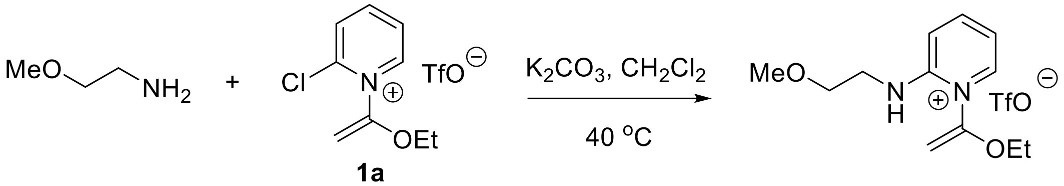

1a

2o

General Amine S_NAr Procedure 2A (Method 1) was followed using 2-methoxyethylamine (43 μL, 0.50 mmol) as the amine nucleophile. Purification by automated column chromatography with a Teledyne ISCO CombiFlash system (0-70% chloroform/isopropanol gradient) yielded product **2o** as an off-white amorphous solid (156 mg, 84%). ^{1}H NMR (500 MHz, Acetone-d_6) δ 8.17 (t, *J* = 8.2 Hz, 1H), 8.13 (d, *J* = 6.9 Hz, 1H), 7.93 (s, 1H), 7.53 (d, *J* = 9.3 Hz, 1H), 7.10 (t, *J* = 6.8 Hz, 1H), 4.88 (app s, 2H), 4.20 (q, *J* = 7.0 Hz, 2H), 3.81 (q, *J* = 5.5 Hz, 2H), 3.66 (t, *J* = 5.2 Hz, 2H), 3.34 (s, 3H), 1.39 (t, *J* = 7.0 Hz, 3H); ^{13}C NMR (126 MHz, Acetone-d_6) δ 152.8, 151.2, 144.6, 139.7, 121.4 (q, $^{1}J_{CF}$ = 322 Hz, CF_3), 112.7, 112.5, 87.1, 70.6, 66.4, 58.2, 42.9,13.2; LRMS-ES+ *m/z* (relative intensity) 223.1 ($C_{12}H_{19}N_2O_2$ M+, 100); HRMS-ES+ ($C_{12}H_{19}N_2O_2$) calcd 223.1447 (M+), found 223.1449.

2-((cyclohexylmethyl)amino)-1-(1-ethoxyvinyl)pyridin-1-ium trifluoromethanesulfonate (**2p**)

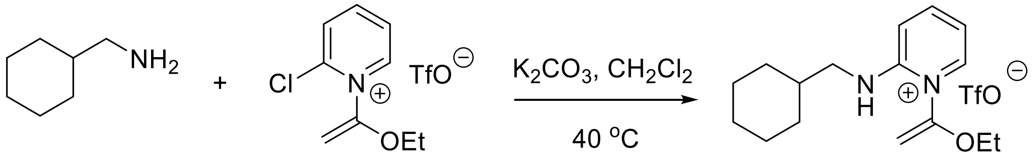

1a

2p

General Amine S_NAr Procedure 2A (Method 1) was followed using cyclohexanemethylamine (65 μL, 0.50 mmol) as the amine nucleophile. Purification by automated column chromatography with a Teledyne ISCO CombiFlash system (0-70% chloroform/isopropanol gradient) yielded product **2p** as a clear oily residue (80 mg, 39%). ^{1}H NMR (500 MHz, Acetone-d_6) δ 8.17 (ddd, *J* = 8.8, 6.9, 1.7 Hz, 1H), 8.13 (dd, *J* = 6.7, 1.7 Hz, 1H), 8.03 (s, 1H), 7.50 (d, *J* = 9.3 Hz, 1H), 7.09 (td, *J* = 6.9, 1.2 Hz, 1H), 4.93 (d, *J* = 4.3 Hz, 1H), 4.88 (d, *J* = 4.4 Hz, 1H), 4.19 (q, *J* = 7.0 Hz, 2H), 3.47 (t, *J* = 6.7 Hz, 2H), 1.88 – 1.71 (m, 5H), 1.39 (t, *J* = 7.0 Hz, 3H), 1.32 – 1.17 (m, 4H), 1.05 (qd, *J* = 12.1, 3.4 Hz, 2H); ^{13}C NMR (126 MHz, Acetone-d_6) δ 152.8, 151.7, 145.2, 140.4, 121.8 (q, $1J_{CF}$ = 323 Hz, CF_3), 112.9, 112.4, 87.5, 66.7, 49.2, 37.4, 30.6, 26.5, 25.9, 13.7; LRMS-ES+ *m/z* (relative intensity) 261.2 ($C_{16}H_{25}N_2O$ M+, 100); HRMS-ES+ ($C_{16}H_{25}N_2O$) calcd 261.1967 (M+), found 261.1974.

1-(1-ethoxyvinyl)-2-morpholinopyridin-1-ium trifluoromethanesulfonate (**2q**)

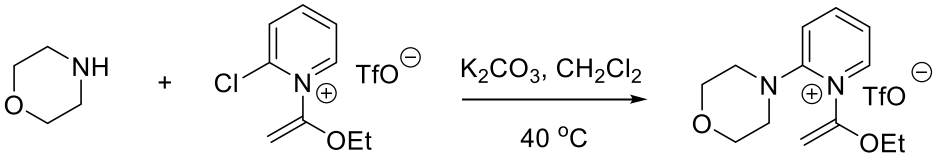

1a

2q

General Amine S_NAr Procedure 2A (Method 1) was followed using morpholine (44 μL, 0.50 mmol) as the amine nucleophile. Purification by automated column chromatography with a Teledyne ISCO CombiFlash system (0-70% chloroform/isopropanol gradient) yielded product **2q** as a white amorphous solid (131 mg @ ~80% purity, 55%). ^{1}H NMR (500 MHz, Acetone-d$_6$) δ 8.21 – 8.06 (m, 2H), 7.65 – 7.55 (m, 1H), 7.17 (td, *J* = 6.8, 1.2 Hz, 1H), 4.80 (d, *J* = 4.7 Hz, 1H), 4.69 (d, *J* = 4.7 Hz, 1H), 4.10 (q, *J* = 7.0 Hz, 2H), 3.79 – 3.60 (m, 8H), 1.31 (t, *J* = 7.0 Hz, 3H); ^{13}C NMR (126 MHz, Acetone-d$_6$) δ 155.7, 153.4, 145.0, 141.6, 121.5 (q, $^1J_{CF}$ = 324 Hz, *C*F$_3$), 118.9, 115.6, 84.9, 66.6, 65.7, 50.2, 13.4; LRMS-ES+ *m/z* (relative intensity) 235.1 ($C_{13}H_{19}N_2O_2$ M+, 100); HRMS-ES+ ($C_{13}H_{19}N_2O_2$) calcd 235.1447 (M+), found 235.1452.

2-(dibenzylamino)-1-(1-ethoxyvinyl)pyridin-1-ium trifluoromethanesulfonate (**2r**)

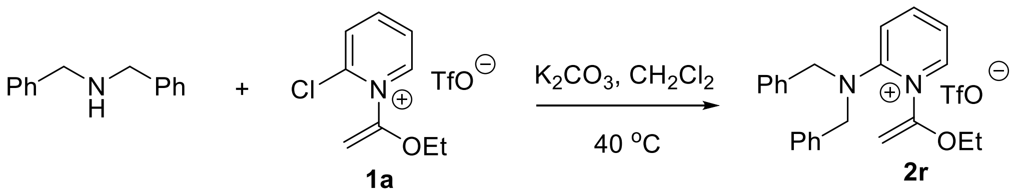

General Amine S_NAr Procedure 2A (Method 1) was followed using dibenzylamine (96 μL, 0.50 mmol) as the amine nucleophile. Purification by automated column chromatography with a Teledyne ISCO CombiFlash system (0-70% chloroform/isopropanol gradient) yielded product **2r** as a white amorphous solid (176 mg, 71%). ^{1}H NMR (500 MHz, Acetone-d$_6$) δ 8.44 (dd, *J* = 6.8, 1.7 Hz, 1H), 8.28 (ddd, *J* = 9.0, 7.1, 1.8 Hz, 1H), 7.80 (dt, *J* = 9.2, 0.8 Hz, 1H), 7.47 – 7.32 (m, 11H), 4.98 – 4.91 (m, 5H), 4.82 (d, *J* = 4.8 Hz, 1H), 4.20 (q, *J* = 7.0 Hz, 2H), 1.44 (t, *J* = 7.0 Hz, 3H); ^{13}C NMR (126 MHz, Acetone-d$_6$) δ 156.1, 154.2, 145.4, 143.2, 135.0, 128.8, 128.2, 128.1, 121.5 (app q, $^1J_{CF}$ = 323 Hz, *C*F$_3$), 119.6, 116.4, 85.7, 66.8, 55.1, 13.4; LRMS-ES+ *m/z* (relative intensity) 345.2 ($C_{23}H_{25}N_2O$ M+, 100); HRMS-ES+ ($C_{23}H_{25}N_2O$) calcd 345.1967 (M+), found 345.1963.

1-(1-ethoxyvinyl)-2-(methyl(phenyl)amino)pyridin-1-ium trifluoromethanesulfonate (**2s**)

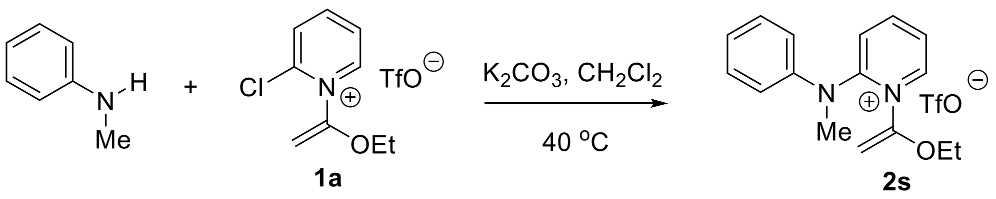

General Amine S_NAr Procedure 2A (Method 1) was followed using *N*-methylaniline (54 μL, 0.50 mmol) as the amine nucleophile. Purification by automated column chromatography with a Teledyne ISCO CombiFlash system (0-70% chloroform/isopropanol gradient) yielded product **2s** as an off-white amorphous solid (190 mg, 94%). ^{1}H NMR (500 MHz, Acetone-d$_6$) δ 8.36 – 8.26 (m, 2H), 7.69 (d, *J* = 9.1 Hz, 1H), 7.52 (t, *J* = 7.7 Hz, 2H), 7.45-7.38 (m, 4H), 4.69 (d, *J* = 4.8 Hz, 1H), 4.36 (d, *J* = 4.8 Hz, 1H), 3.74 (s, 3H), 3.63 (q, *J* = 7.1 Hz, 2H), 1.27 (t, *J* = 7.0 Hz, 3H); ^{13}C NMR (126 MHz, Acetone-d$_6$) δ 154.5, 152.6, 145.5, 144.2, 142.6, 129.8, 127.9, 126.4, 121.5 (q, $^1J_{CF}$ = 323 Hz, *C*F$_3$), 119.5, 116.4, 85.9, 65.8, 43.4, 13.2.; LRMS-ES+ *m/z* (relative intensity) 255.1 ($C_{16}H_{19}N_2O$ M+, 100); HRMS-ES+ ($C_{16}H_{19}N_2O$) calcd 255.1497 (M+), found 255.1497. X-ray crystallography data has been previously reported.[3]

1-(1-ethoxyvinyl)-2-((4-methoxyphenyl)amino)pyridin-1-ium trifluoromethanesulfonate (**2t**)

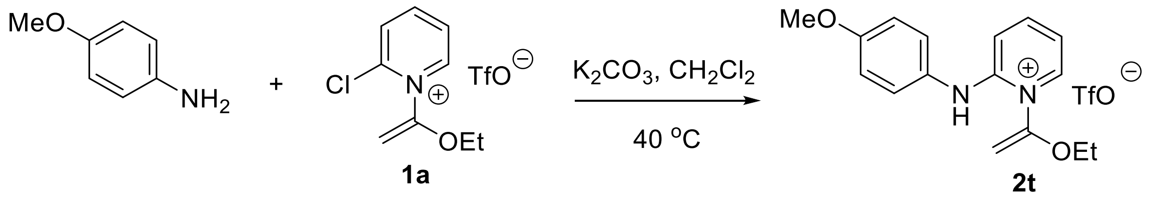

General Amine S_NAr Procedure 2A (Method 1) was followed using *p*-anisidine (62 mg, 0.50 mmol) as the amine nucleophile. Purification by automated column chromatography with a Teledyne ISCO CombiFlash system (0-70%

chloroform/isopropanol gradient) yielded product **2t** as a brown amorphous solid (164 mg, 78%). ^{1}H NMR (500 MHz, CD_3OD) δ 8.09 (dd, *J* = 6.7, 1.6 Hz, 1H), 7.97 (ddd, *J* = 9.0, 7.1, 1.7 Hz, 1H), 7.32 (d, *J* = 8.9 Hz, 2H), 7.10 (d, *J* = 8.9 Hz, 2H), 7.05 (t, *J* = 6.9 Hz, 1H), 6.96 (d, *J* = 9.2 Hz, 1H), 4.89 (d, *J* = 4.4 Hz, 1H), 4.85 (d, *J* = 4.5 Hz, 1H), 4.21 (q, *J* = 7.0 Hz, 2H), 3.87 (s, 3H), 1.45 (t, *J* = 7.0 Hz, 3H), the N-H proton was not observed due to rapid hydrogen-deuterium exchange in CD_3OD; ^{13}C NMR (126 MHz, CD_3OD_SPE) δ 159.9, 152.8, 151.4, 144.6, 139.8, 128.0, 127.8, 120.4 (q, $^{1}J_{CF}$ = 319 Hz, *C*F_3), 115.2, 113.4, 112.7, 86.4, 66.3, 54.77, 12.9; LRMS-ES+ *m/z* (relative intensity) 271.1 ($C_{16}H_{19}N_2O_2$ M+, 5); HRMS-ES+ ($C_{16}H_{19}N_2O_2$) calcd 271.1447 (M+), found 271.1440.

2-((4-acetylphenyl)amino)-1-(1-ethoxyvinyl)pyridin-1-ium trifluoromethanesulfonate (**2u**)

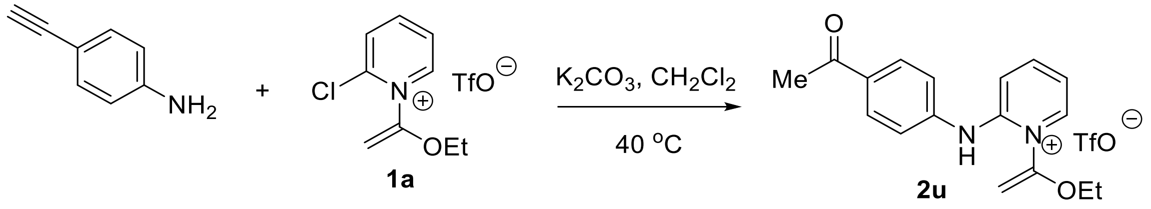

General Amine S_NAr Procedure 2A (Method 1) was followed using 4-ethynylaniline (59 mg, 0.50 mmol) as the amine nucleophile. Purification by automated column chromatography with a Teledyne ISCO CombiFlash system (0-70% chloroform/isopropanol gradient) yielded product **2u** as a yellow amorphous solid (93 mg, 43%). ^{1}H NMR (500 MHz, CD_3OD) δ 8.20 (dd, *J* = 6.8, 1.7 Hz, 1H), 8.15 (dtd, *J* = 7.4, 5.2, 2.6 Hz, 2H), 8.06 (ddd, *J* = 9.0, 7.1, 1.8 Hz, 1H), 7.57 – 7.49 (m, 2H), 7.26 – 7.20 (m, 1H), 7.16 (td, *J* = 7.0, 1.3 Hz, 1H), 4.91 (p, *J* = 4.5 Hz, 1H), 4.86 (d, *J* = 3.9 Hz, 1H), 4.19 (q, *J* = 7.0 Hz, 2H), 2.64 (s, 3H), 1.42 (t, *J* = 7.0 Hz, 3H), the N-H proton was not observed due to rapid hydrogen-deuterium exchange in CD_3OD; ^{13}C NMR (126 MHz, CD_3OD) δ 197.7, 151.8, 151.4, 145.5, 140.6, 140.1, 136.3, 130.1, 125.7, 120.4 (q, $^{1}J_{CF}$ = 319 Hz, *C*F_3), 114.8, 113.3, 86.5, 66.4, 25.4, 12.9; LRMS-ES+ *m/z* (relative intensity) 283.1 ($C_{17}H_{19}N_2O_2$ M+, 100); HRMS-ES+ ($C_{17}H_{19}N_2O_2$) calcd 283.1447 (M+), found 283.1453.

1-(1-ethoxyvinyl)-2-((4-sulfamoylphenyl)amino)pyridin-1-ium trifluoromethanesulfonate (**2v**)

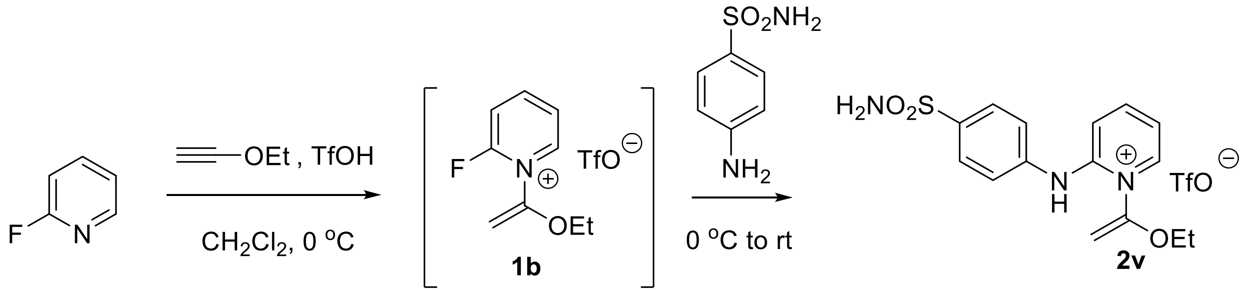

General Amine S_NAr Procedure 2B (Method 2) was followed using sulfanilamide (172 mg, 1.0 mmol) as the amine nucleophile. Purification by automated column chromatography with a Teledyne ISCO CombiFlash system (0-100% chloroform/isopropanol gradient) yielded product **2v** as a yellow amorphous solid (305 mg, 65 %). ^{1}H NMR (500 MHz, Acetone-d_6) δ 9.77 (s, 1H), 8.40 – 8.35 (m, 1H), 8.23 (ddt, *J* = 9.0, 7.1, 1.7 Hz, 1H), 8.03 (dtd, *J* = 6.8, 3.8, 2.2 Hz, 2H), 7.68 (dt, *J* = 9.2, 2.2 Hz, 2H), 7.42 (d, *J* = 9.2 Hz, 1H), 7.33 (tq, *J* = 5.4, 1.9 Hz, 1H), 6.74 (s, 2H), 5.08 (dd, *J* = 4.4, 1.5 Hz, 1H), 4.94 (dd, *J* = 4.6, 1.4 Hz, 1H), 4.24 (qd, *J* = 7.0, 1.5 Hz, 2H), 1.41 (td, *J* = 7.0, 1.5 Hz, 3H); ^{13}C NMR (126 MHz, Acetone-d_6) δ 151.8, 150.9, 146.1, 143.2, 140.9, 139.0, 128.0, 126.2, 121.1 (q, $^{1}J_{CF}$ = 321 Hz, *C*F_3), 115.6, 113.7, 87.6, 66.6, 13.4; LRMS-ES+ *m/z* (relative intensity) 320.1 ($C_{15}H_{18}N_3O_3S$ M+, 100); HRMS-ES+ ($C_{15}H_{18}N_3O_3S$) calcd 320.1069 (M+), found 320.1068.

2,2'-(1,4-phenylenebis(azanediyl))bis(1-(1-ethoxyvinyl)pyridin-1-ium) trifluoromethanesulfonate (**2w**)

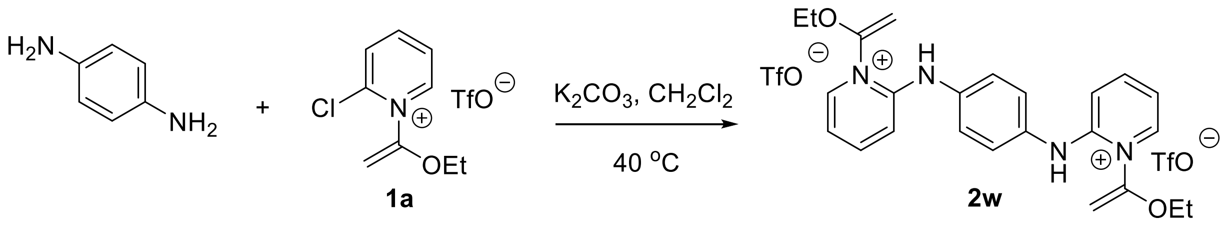

General Amine S_NAr Procedure 2A (Method 1) was followed using *p*-phenylenediamine (27 mg, 0.25 mmol, 0.5 equiv) as the amine nucleophile. Purification by automated column chromatography with a Teledyne ISCO CombiFlash system (0-100% chloroform/isopropanol gradient) yielded product **2w** as a light brown amorphous solid (119 mg @ ~92% purity, 63%). ^{1}H NMR (500 MHz, Acetone-d$_6$) δ 7.62 (dd, *J* = 6.9, 1.8 Hz, 2H), 7.44 (tdd, *J* = 8.8, 7.0, 1.8 Hz, 2H), 6.98 (s, 4H), 6.69 (d, *J* = 9.4 Hz, 2H), 6.43 (t, *J* = 6.8 Hz, 2H), 4.56 (d, *J* = 3.8 Hz, 2H), 4.53 (d, *J* = 3.8 Hz, 2H), 3.97 (q, *J* = 7.0 Hz, 4H), 1.23 (t, *J* = 7.0 Hz, 6H), NH peaks were not observed; ^{13}C NMR (126 MHz, Acetone-d$_6$) δ 153.8, 152.0, 141.0, 139.3, 125.6, 121.1 (q, $^1J_{CF}$ = 321 Hz, *C*F$_3$), 199.1, 113.8, 108.7, 85.4, 65.8, 13.5; LRMS-ES+ *m/z* (relative intensity) 403.21 ($C_{24}H_{27}N_4O_2$ M-H+, 100); HRMS-ES+ ($C_{24}H_{27}N_4O_2$ M-H+) calcd 403.2114 (M-H+), found 403.2134.

2,2'-((1,4-phenylenebis(methylene))bis(azanediyl))bis(1-(1-ethoxyvinyl)pyridin-1-ium) trifluoromethanesulfonate (**2x**)

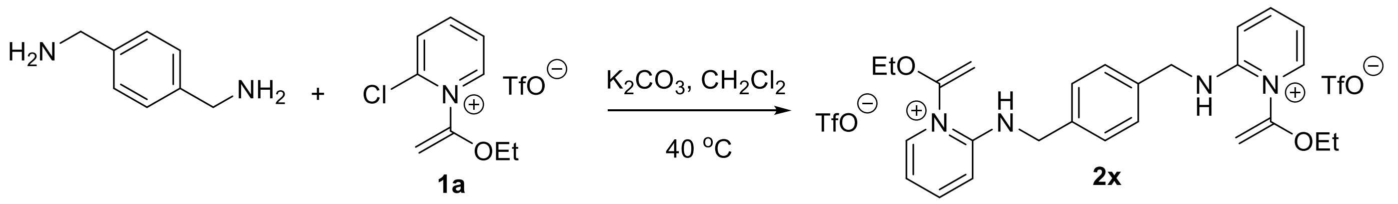

General Amine S_NAr Procedure 2A (Method 1) was followed using *p*-xylylenediamine (34 mg, 0.25 mmol, 0.5 equiv) as the amine nucleophile. Purification by automated column chromatography with a Teledyne ISCO CombiFlash system (0-100% chloroform/isopropanol gradient) yielded product **2x** as a pale yellow amorphous solid (55 mg, 30%). ^{1}H NMR (500 MHz, Acetone-d$_6$) δ 8.53 (t, *J* = 6.3 Hz, 2H), 8.05 (dd, *J* = 6.7, 1.6 Hz, 2H), 7.97 (t, *J* = 8.1 Hz, 2H), 7.35 (s, 4H), 7.18 (d, *J* = 9.2 Hz, 2H), 6.97 (td, *J* = 6.9, 1.1 Hz, 2H), 4.82 (d, *J* = 4.4 Hz, 2H), 4.76 (d, *J* = 4.4 Hz, 2H), 4.72 (d, *J* = 6.4 Hz, 4H), 4.09 (q, *J* = 7.0 Hz, 4H), 1.25 (t, *J* = 7.0 Hz, 6H); ^{13}C NMR (126 MHz, Acetone-d$_6$) δ 152.5, 151.2, 145.3, 140.3, 136.1, 127.6, 121.4 (q, $^1J_{CF}$ = 322 Hz, *C*F$_3$), 113.2, 112.5, 87.2, 66.5, 45.5, 13.3; LRMS-ES+ *m/z* (relative intensity) 431.25 ($C_{26}H_{31}N_4O_2$ M-H+, 100); HRMS-ES+ ($C_{26}H_{31}N_4O_2$ M-H+) calcd 431.2447 (M-H+), found 431.2447.

2-((2-(1*H*-indol-3-yl)ethyl)amino)-1-(1-ethoxyvinyl)pyridin-1-ium trifluoromethanesulfonate (**2y**)

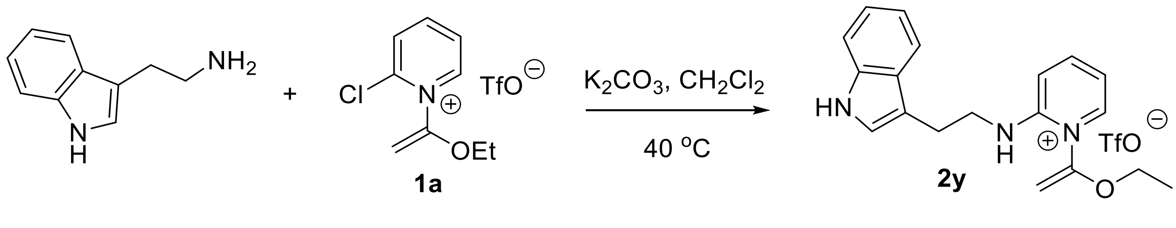

General Amine S_NAr Procedure 2A (Method 1) was followed using tryptamine (80 mg, 0.50 mmol) as the amine nucleophile. Purification by automated column chromatography with a Teledyne ISCO CombiFlash system (0-100% chloroform/isopropanol gradient) yielded product **2y** as a white amorphous solid (165 mg, 72%). ^{1}H NMR (500 MHz, CD$_3$OD) δ 7.85 (dd, *J* = 6.8, 1.6 Hz, 1H), 7.74 (ddd, *J* = 8.9, 7.0, 1.7 Hz, 1H), 7.54 (d, *J* = 7.9 Hz, 1H), 7.34 (d, *J* = 8.1 Hz, 1H), 7.14 – 7.09 (m, 2H), 7.02 (td, *J* = 7.5, 0.9 Hz, 1H), 6.95 (d, *J* = 9.3 Hz, 1H), 6.81 (td, *J* = 6.9, 1.1 Hz, 1H), 4.67 (d, *J* = 4.4 Hz, 1H), 4.51 (d, *J* = 4.4 Hz, 1H), 4.02 (q, *J* = 7.0 Hz, 2H), 3.81 (t, *J* = 6.5 Hz, 2H), 3.16 (t, *J* = 6.5 Hz, 2H), 1.33 (t, *J* = 7.0 Hz, 4H); ^{13}C NMR (126 MHz, Acetone-d$_6$) δ 152.3, 150.9, 144.4, 139.6, 136.8, 127.5, 123.6, 121.4, 121.3 (q, $^1J_{CF}$ = 321 Hz, *C*F$_3$), 118.8, 118.2, 112.4, 111.8, 111.6, 110.6, 87.1, 66.3, 43.7, 24.2, 13.3; LRMS-ES+ *m/z* (relative intensity) 308.2 ($C_{19}H_{22}N_3O$ M+, 100); HRMS-ES+ ($C_{19}H_{22}N_3O$) calcd 308.1763 (M+), found 308.1763.

1-(1-ethoxyvinyl)-2-(methyl(3-phenyl-3-(4-(trifluoromethyl)phenoxy)propyl)amino)pyridin-1-ium trifluoromethanesulfonate (**2z**)

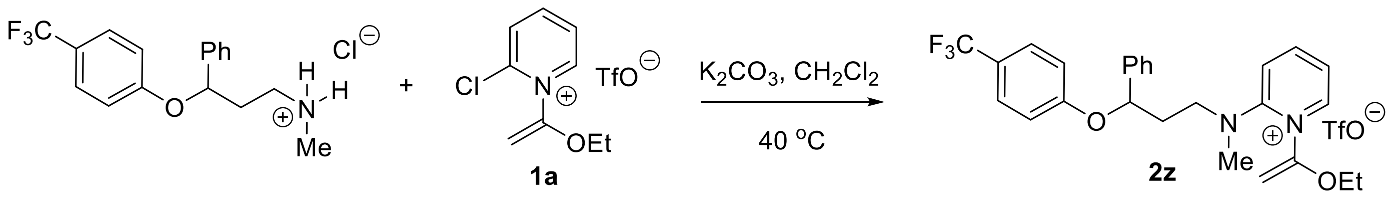

General Amine S_NAr Procedure 2A (Method 1) was followed using racemic fluoxetine hydrochloride (56296-78-7) (104 mg, 0.3 mmol) as the amine nucleophile and an extra equivalent of potassium carbonate (138 mg, 1.0 mmol). Purification by automated column chromatography with a Teledyne ISCO CombiFlash system (0-70% chloroform/isopropanol gradient) yielded product **2z** as a yellow amorphous solid (60 mg, 66%). ^{1}H NMR (500 MHz, CD_3CN) δ 7.87 (ddd, *J* = 9.1, 7.0, 1.8 Hz, 1H), 7.81 (dd, *J* = 6.8, 1.7 Hz, 1H), 7.52 (d, *J* = 8.6 Hz, 2H), 7.44 – 7.37 (m, 4H), 7.33 (dd, *J* = 8.2, 5.6 Hz, 2H), 7.00 (d, *J* = 8.6 Hz, 2H), 6.93 (td, *J* = 6.8, 1.2 Hz, 1H), 5.39 (dd, *J* = 8.6, 4.5 Hz, 1H), 4.60 (d, *J* = 4.7 Hz, 1H), 4.54 (d, *J* = 4.7 Hz, 1H), 4.09 (q, *J* = 7.0 Hz, 2H), 3.84 – 3.76 (m, 2H), 3.25 (s, 3H), 2.33 (qd, *J* = 8.2, 5.8 Hz, 2H), 1.40 (t, *J* = 7.0 Hz, 3H); ^{13}C NMR (126 MHz, CD_3CN) δ 160.2, 155.2, 153.0, 143.4, 140.8, 140.0, 128.9, 128.3, 126.8, 126.8, 126.0, 124.6 (app q, $^1J_{CF}$ = 271 Hz, aryl *C*F$_3$), 121.1 (app q, $^1J_{CF}$ = 309 Hz, triflate *C*F$_3$), 117.2, 116.2, 113.8, 85.0, 77.1, 66.6, 50.7, 39.3, 34.9, 13.3; LRMS-ES+ *m/z* (relative intensity) 457.2 ($C_{26}H_{28}F_3N_2O_2$ M+, 100); HRMS-ES+ ($C_{26}H_{28}F_3N_2O_2$) calcd 457.2103 (M+), found 457.2100.

DBCO-aminopyridinium triflate **2aa**

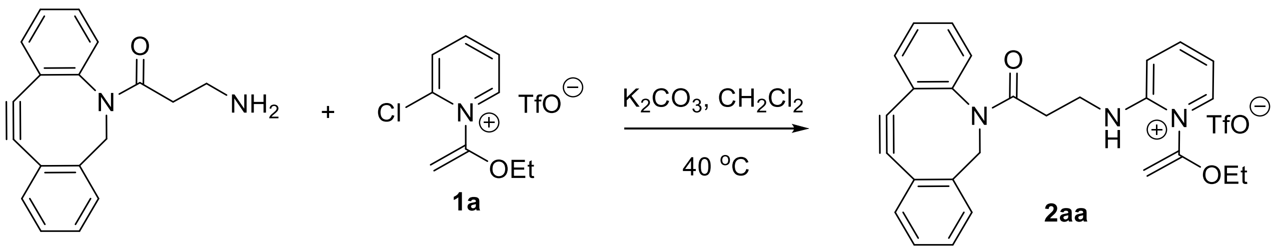

General Amine S_NAr Procedure 2A (Method 1) was followed using "dibenzocyclooctyne-amine" (CAS# 1255942-06-3) (34.8 mg, 0.126 mmol) as the amine nucleophile. Purification by automated column chromatography with a Teledyne ISCO CombiFlash system (0-70% chloroform/isopropanol gradient) yielded product **2aa** as a pale yellow amorphous solid (69 mg, 96%). ^{1}H NMR (500 MHz, CD_3OD) δ 7.87 (dd, *J* = 6.6, 1.7 Hz, 1H), 7.72 – 7.64 (m, 2H), 7.55 – 7.44 (m, 4H), 7.37 (td, *J* = 7.6, 1.5 Hz, 1H), 7.31 (dd, *J* = 8.3, 7.0 Hz, 1H), 7.13 (dd, *J* = 7.6, 1.5 Hz, 1H), 6.98 (d, *J* = 9.4 Hz, 1H), 6.85 (t, *J* = 6.8 Hz, 1H), 5.16 (d, *J* = 14.0 Hz, 1H), 4.65 (d, *J* = 4.5 Hz, 1H), 4.55 (d, *J* = 4.4 Hz, 1H), 4.03 (qd, *J* = 7.0, 1.8 Hz, 2H), 3.72 (dq, *J* = 14.0, 4.8 Hz, 2H), 3.46 (dt, *J* = 14.2, 5.3 Hz, 1H), 2.68 (dt, *J* = 16.1, 5.1 Hz, 1H), 2.28 (ddd, *J* = 15.9, 9.0, 5.0 Hz, 1H), 1.37 (t, *J* = 7.0 Hz, 3H); ^{13}C NMR (126 MHz, CD_3OD) δ 171.3, 151.7, 151.1, 151.0, 147.8, 144.1, 139.1, 132.2, 129.1, 128.8, 128.5, 127.9, 127.7, 126.9, 125.2, 122.8, 122.1, 120.6 (q, $^1J_{CF}$ = 320 Hz, *C*F$_3$), 114.2, 112.3, 111.2, 107.5, 86.2, 66.2, 55.2, 39.3, 33.7, 12.9; LRMS-ES+ *m/z* (relative intensity) 424.2 ($C_{27}H_{26}N_3O_2$ M+, 100); HRMS-ES+ ($C_{27}H_{26}N_3O_2$) calcd 424.2025 (M+), found 424.2029.

2-(benzylamino)-3-chloro-1-(1-ethoxyvinyl)pyridin-1-ium trifluoromethanesulfonate (**4**)

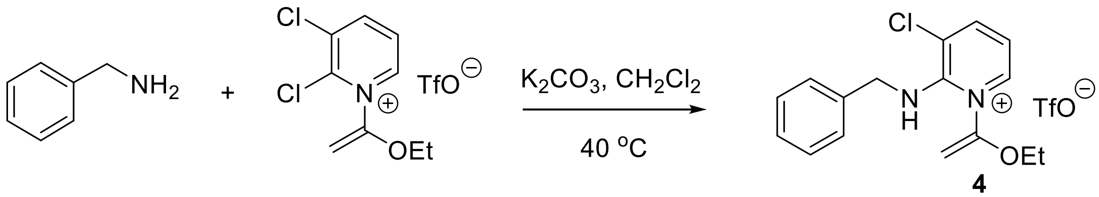

A 0.5-2.0 mL Biotage microwave vial was charged with freshly prepared 1-(1-ethoxyvinyl)-2,3-dichloropyridinium triflate (74 mg, 0.20 mmol),[1] 1 mL of dichloromethane, benzylamine (22 μL, 0.20 mmol), and then anhydrous potassium carbonate (28 mg, 0.20 mmol). The resulting suspension was stirred for one minute before sealing with a crimped septum cap and placing into a pre-heated 40 °C oil bath for 24h. Before uncapping the vial, the reaction was cooled to room

temperature and then the septum was punctured with a needle to release pressure. The reaction mixture was then transferred by pipette to a round-bottom flask using acetone or methanol to transfer over any remaining organic soluble residue. The resulting solution was concentrated in vacuo to yield a residue that was purified by automated column chromatography with a Teledyne ISCO CombiFlash system (0-100% chloroform/isopropanol gradient; 4g RediSep Gold® pre-packed silica gel cartridge) to yield product **4** as a light brown amorphous solid (37 mg, 42%). ^{1}H NMR (500 MHz, Acetone-d$_6$) δ 8.22 (dd, *J* = 7.7, 1.4 Hz, 1H), 8.09 (dd, *J* = 6.7, 1.4 Hz, 1H), 7.45 (d, *J* = 8.4 Hz, 2H), 7.39 (t, *J* = 7.6 Hz, 2H), 7.35 – 7.29 (m, 1H), 7.08 (t, *J* = 7.2 Hz, 1H), 5.06 (s, 2H), 4.89 (d, *J* = 4.5 Hz, 1H), 4.79 (d, *J* = 4.5 Hz, 1H), 4.16 (q, *J* = 7.0 Hz, 2H), 1.35 (t, *J* = 7.0 Hz, 3H); ^{13}C NMR (126 MHz, Acetone-d$_6$) δ 152.4, 150.0, 142.7, 140.0, 137.7, 128.6, 127.6, 127.1, 122.3, 121.3 (q, $^1J_{CF}$ = 322 Hz, *C*F$_3$), 112.6, 86.9, 66.4, 49.0, 13.3; LRMS-ES+ *m/z* (relative intensity) 289.1 ($C_{16}H_{18}ClN_2O$ M+, 100); HRMS-ES+ ($C_{16}H_{18}ClN_2O$) calcd 289.1108 (M+), found 289.1104.

2-(benzylamino)pyridin-1-ium trifluoromethanesulfonate (**5a**)

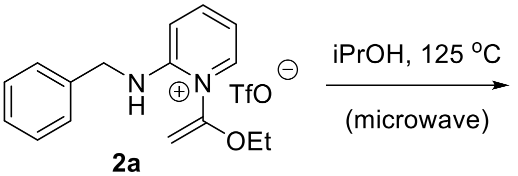

2a **5a**

General *N*-(1-Ethoxyvinyl) Cleavage Procedure 3A (Method 1, Thermolytic) was followed using *N*-(1-ethoxyvinyl) 2-aminopyridinium salt **2a** (150 mg, 0.37 mmol), yielding *N*-H pyridinium salt **5a** as a white amorphous solid (123 mg, 99%). ^{1}H NMR (500 MHz, Acetone-d$_6$) δ 8.01 – 7.87 (m, 2H), 7.79 (ddd, *J* = 8.9, 7.0, 1.7 Hz, 1H), 7.36 – 7.29 (m, 2H), 7.27 – 7.19 (m, 2H), 7.20 – 7.13 (m, 1H), 7.00 (d, *J* = 9.0 Hz, 1H), 6.83 – 6.76 (m, 1H), 5.15 (br s, 1H), 4.59 (d, *J* = 5.4 Hz, 2H); ^{13}C NMR (126 MHz, Acetone-d$_6$) δ 154.3, 142.8, 138.3, 136.9, 128.7, 127.7, 127.60, 121.0 (q, $^1J_{CF}$ = 321 Hz, *C*F$_3$), 112.8, 112.0, 45.5; LRMS-ES+ *m/z* (relative intensity) 185.1 ($C_{12}H_{13}N_2$ M+, 100); HRMS-ES+ ($C_{12}H_{13}N_2$) calcd 185.1079 (M+), found 185.1081.

2-((4-methoxybenzyl)amino)pyridin-1-ium trifluoromethanesulfonate (**5b**)

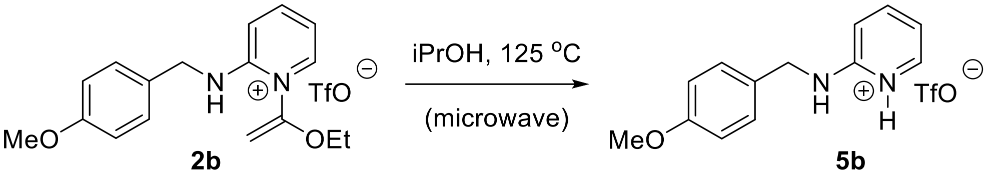

2b **5b**

General *N*-(1-Ethoxyvinyl) Cleavage Procedure 3A (Method 1, Thermolytic) was followed using *N*-(1-ethoxyvinyl) 2-aminopyridinium salt **2b** (51 mg, 0.12 mmol), yielding *N*-H pyridinium salt **5b** as an off-white amorphous solid (40 mg, 94%). ^{1}H NMR (500 MHz, CDCl$_3$) δ 8.22 (s, 1H), 7.81 (dd, *J* = 8.2, 6.4 Hz, 2H), 7.28 (d, *J* = 7.2 Hz, 2H), 6.88 (dd, *J* = 13.1, 9.0 Hz, 3H), 6.82 (t, *J* = 6.7 Hz, 1H), 4.50 (d, *J* = 5.7 Hz, 2H), 3.80 (s, 3H); ^{13}C NMR (126 MHz, Acetone) δ 159.7, 152.9, 144.2, 135.9, 130.5, 129.2, 127.5, 121.0 (q, $^1J_{CF}$ = 320 Hz, *C*F$_3$), 114.2, 112.8, 54.7, 45.2; LRMS-ES+ *m/z* (relative intensity) 215.1 ($C_{13}H_{15}N_2O$ M+, 100); HRMS-ES+ ($C_{13}H_{15}N_2O$) calcd 215.1184 (M+), found 215.1188.

2-((2,4,6-trifluorobenzyl)amino)pyridin-1-ium trifluoromethanesulfonate (**5g**)

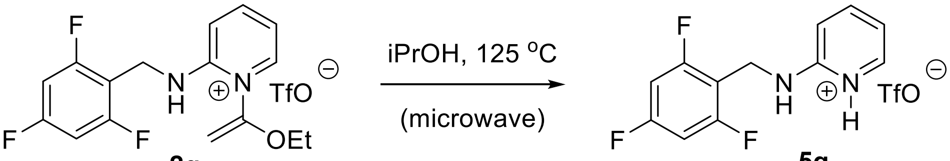

2g **5g**

General *N*-(1-Ethoxyvinyl) Cleavage Procedure 3A (Method 1, Thermolytic) was followed using *N*-(1-ethoxyvinyl) 2-aminopyridinium salt **2g** (103 mg, 0.225 mmol), yielding *N*-H pyridinium salt **5g** as a pale yellow amorphous solid (46 mg, 53%). ^{1}H NMR (500 MHz, CDCl$_3$) δ 13.21 (br s, 1H), 8.28 (s, 1H), 7.96 (t, *J* = 8.3 Hz, 1H), 7.89 – 7.84 (m, 1H), 7.09

(d, J = 9.2 Hz, 1H), 6.91 (t, J = 6.7 Hz, 1H), 6.74 (t, J = 8.1 Hz, 2H), 4.58 (d, J = 5.8 Hz, 2H); ^{13}C NMR (126 MHz, $CDCl_3$) δ 163.0 (dt, $^1J_{CF}$ = 249 Hz, $^3J_{CF}$ = 16 Hz, C4-F coupling on benzene ring), 161.9 (dq, $^1J_{CF}$ = 250 Hz, $^3J_{CF}$ = 15 Hz, C2-F coupling on benzene ring), 152.8 (C2 of pyridinium ring), 144.8 (C4 of pyridinium ring), 136.2 (C6 of pyridinium ring), 121.0 (q, $^1J_{CF}$ = 321 Hz, CF_3), 113.3 (C3 of pyridinium ring), 112.5 (C5 of pyridinium ring), 108.2 (app t, , $^2J_{CF}$ = 20 Hz, C1-F coupling on benzene ring), 100.6 (app t, $^2J_{CF}$ = 29 Hz, C3-F coupling on benzene ring), 33.9 (t, $^3J_{CF}$ = 3.8 Hz, benzyl CH_2); LRMS-ES+ *m/z* (relative intensity) 239.1 ($C_{12}H_{10}F_3N_2$ M+, 100); HRMS-ES+ ($C_{12}H_{10}F_3N_2$) calcd 239.0796 (M+), found 239.0799.

2-((2-methoxyethyl)amino)pyridin-1-ium trifluoromethanesulfonate (**5o**)

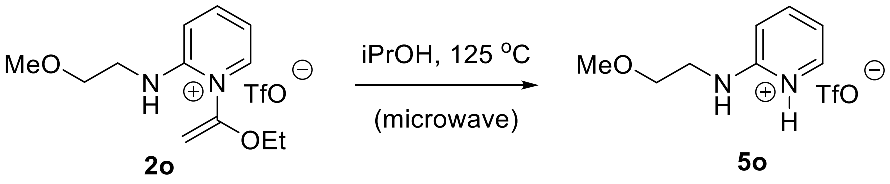

General *N*-(1-Ethoxyvinyl) Cleavage Procedure 3A (Method 1, Thermolytic) was followed using *N*-(1-ethoxyvinyl) 2-aminopyridinium salt **2o** (108 mg, 0.29 mmol), yielding *N*-H pyridinium salt **5o** as a white amorphous solid (70 mg, 80%). ^{1}H NMR (500 MHz, $CDCl_3$) δ 7.89 – 7.74 (m, 3H), 7.07 (d, J = 9.1 Hz, 1H), 6.81 (t, J = 6.7 Hz, 1H), 6.28 (br s, 1H), 3.66 (t, J = 4.9 Hz, 2H), 3.57 (q, J = 5.2 Hz, 2H), 3.40 (s, 3H); ^{13}C NMR (126 MHz, Acetone-d_6) δ 153.8, 143.5, 135.9, 121.1 (q, $^1J_{CF}$ = 321 Hz, CF_3), 113.4, 112.6, 70.5, 58.1, 42.3; LRMS-ES+ *m/z* (relative intensity) 153.1 ($C_8H_{13}N_2O$ M+, 100); HRMS-ES+ ($C_8H_{13}N_2O$) calcd 153.1028 (M+), found 153.1028.

2-(methyl(phenyl)amino)pyridin-1-ium (**5s**)

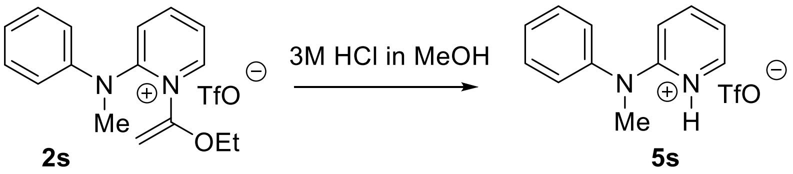

General *N*-(1-Ethoxyvinyl) Cleavage Procedure 3B (Method 2, Acidic) was followed using *N*-(1-ethoxyvinyl) 2-aminopyridinium salt **2s** (50 mg, 0.12 mmol), yielding *N*-H pyridinium salt **5s** as a pale yellow amorphous solid (39 mg, 95%). ^{1}H NMR (500 MHz, $CDCl_3$) δ 13.05 (s, 1H), 8.14 (t, J = 5.3 Hz, 1H), 7.66 (t, J = 8.0 Hz, 1H), 7.51 (t, J = 7.5 Hz, 2H), 7.43 (t, J = 7.4 Hz, 1H), 7.22 (d, J = 7.8 Hz, 2H), 6.86 (t, J = 6.5 Hz, 1H), 6.59 (d, J = 9.1 Hz, 1H), 3.60 (s, 3H); ^{13}C NMR (126 MHz, $CDCl_3$) δ 152.6, 143.3, 141.8, 137.1, 131.3, 129.7, 126.7, 120.4 (q, $^1J_{CF}$ = 319 Hz, CF_3), 113.23, 113.22, 40.6; LRMS-ES+ *m/z* (relative intensity) 185.1 ($C_{12}H_{13}N_2$ M+, 30); HRMS-ES+ ($C_{12}H_{13}N_2$) calcd 185.1079 (M+), found 185.1081.

2-((4-acetylphenyl)amino)pyridin-1-ium trifluoromethanesulfonate (**5u**)

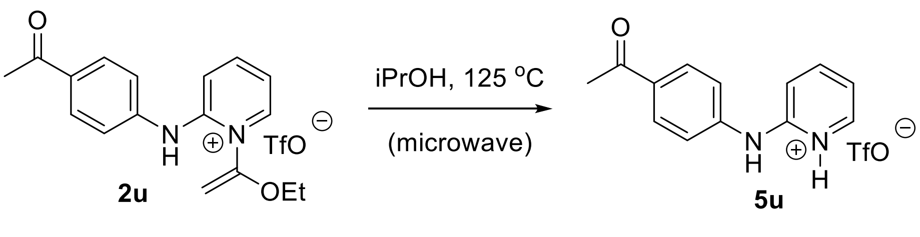

General *N*-(1-Ethoxyvinyl) Cleavage Procedure 3A (Method 1, Thermolytic) was followed using *N*-(1-ethoxyvinyl) 2-aminopyridinium salt **2u** (62 mg, 0.15 mmol), yielding *N*-H pyridinium salt **5u** as a pale yellow amorphous solid (42 mg, 75%). ^{1}H NMR (500 MHz, $CDCl_3$) δ 13.91 (s, 1H), 10.13 (s, 1H), 8.07 (d, J = 8.1 Hz, 2H), 8.00 (d, J = 7.0 Hz, 2H), 7.42 (d, J = 8.2 Hz, 2H), 7.38 (d, J = 9.0 Hz, 1H), 7.09 (t, J = 6.7 Hz, 1H), 2.65 (s, 3H); ^{13}C NMR (126 MHz, Acetone-d_6) δ

195.9, 151.3, 145.9, 140.2, 137.4, 135.3, 130.3, 122.7, 121.1 (q, $^1J_{CF}$ = 321 Hz, CF_3), 115.7, 113.9, 25.8; LRMS-ES+ *m/z* (relative intensity) 213.1 ($C_{13}H_{13}N_2O$ M+, 100); HRMS-ES+ ($C_{13}H_{13}N_2O$) calcd 213.1028 (M+), found 213.1029.

N-benzylpyridin-2-amine (**6a**)

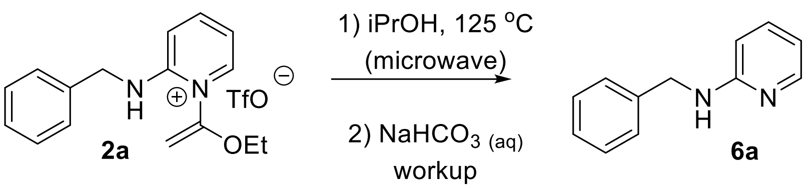

1. The corresponding *N*-(1-ethoxyvinyl) 2-aminopyridinium triflate **2a** (100 mg, 0.25 mmol) was dissolved in isopropanol (1 mL) in a 0.5-2.0 mL Biotage microwave vial. The vial was sealed with a crimped septum cap, then placed into a pre-heated 125 °C oil bath for 17 h. Before uncapping the vial, the reaction was cooled to room temperature and then the septum was punctured with a needle to release pressure. The reaction mixture was then transferred to a round-bottom flask using additional isopropanol, then concentrated in vacuo to produce a residue. The resulting residue was dissolved in 25 mL of EtOAc and washed with saturated $NaHCO_{3\ (aq)}$ 4x10mL. The organic layer was dried over anhydrous sodium sulfate, then concentrated in vacuo to yield **6a** as a clear thin film (46 mg, 99%). Spectral data matches those previously reported.[4]

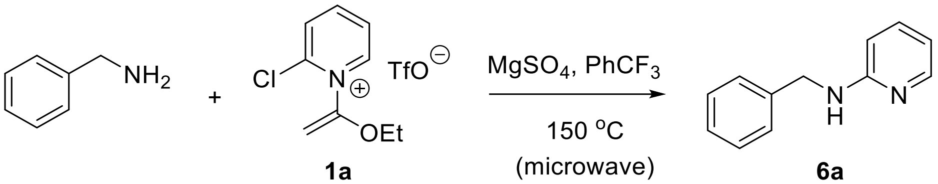

2. Tandem S_NAr and *N*-(1-Ethoxyvinyl) Cleavage Procedure 2C (Method 3) was followed using benzylamine (35 mg, 0.30 mmol) as the nucleophile. Purification by automated column chromatography with a Teledyne ISCO CombiFlash system (0-100% hexanes/ethyl acetate gradient) yielded product **12** clear thin film (22 mg, 40%). Spectral data of product **12** matched those previously reported.[4]

2-((4-bromobenzyl)amino)pyridin-1-ium trifluoromethanesulfonate (**6f**)

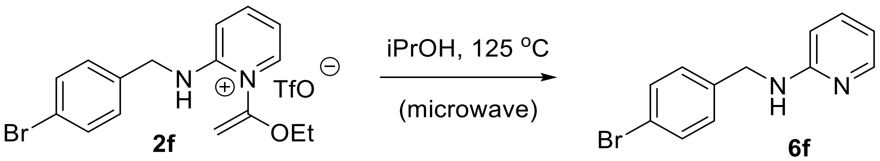

General *N*-(1-Ethoxyvinyl) Cleavage Procedure 3A (Method 1, Thermolytic) was followed using *N*-(1-ethoxyvinyl) 2-aminopyridinium salt **2f** (37 mg, 0.076 mmol), yielding *N*-H pyridinium salt **6f** as a pale yellow amorphous solid (30 mg, 95%) . ^{1}H NMR (500 MHz, Acetone-d_6) δ 7.85 (ddd, *J* = 5.0, 2.0, 0.9 Hz, 1H), 7.38 – 7.26 (m, 2H), 7.23 (ddd, *J* = 8.7, 7.0, 2.0 Hz, 1H), 7.21 – 7.13 (m, 2H), 6.44 – 6.32 (m, 2H), 6.15 (s, 1H), 4.42 (d, *J* = 6.0 Hz, 2H); ^{13}C NMR (126 MHz, Acetone-d_6) δ 158.8, 147.8, 140.5, 136.7, 131.2, 129.4, 119.8, 112.3, 108.0, 44.1; LRMS-ES+ *m/z* (relative intensity) 263.0 ($C_{12}H_{11}BrN_2$ M+H, 100 (Br-79 isotope)), 265.0 ($C_{12}H_{12}BrN_2$ M+H, 98 (Br-81 isotope)); HRMS-ES+ ($C_{12}H_{12}BrN_2$) calcd 263.0184 (M+H), found 263.0185.

2-morpholinopyridin-1-ium trifluoromethanesulfonate (**6q**)

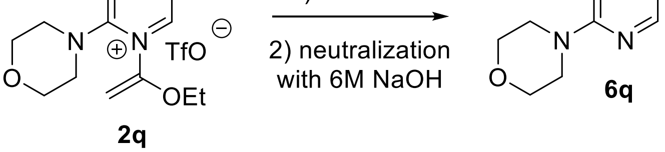

Compound **2q** (154 mg, 0.40 mmol) was dissolved in 1 mL of concentrated hydrochloric acid for 17h. Following this, the reaction mixture was carefully pipetted dropwise into an ice water bath-chilled beaker containing 25 mL of

deionized water. The dilute solution was then carefully neutralized to a pH of 7 (as indicated by pH paper) via dropwise addition of a 6M aqueous solution of sodium hydroxide. The aqueous layer was then extracted 3x with EtOAc (15 mL), dried over anhydrous sodium sulfate, then concentrated in vacuo to yield product **6q** as a clear thin film (44 mg, 66%). Spectral data of **6q** matched those previously reported.[5]

2-(benzylamino)pyridine 1-oxide (**7a**)

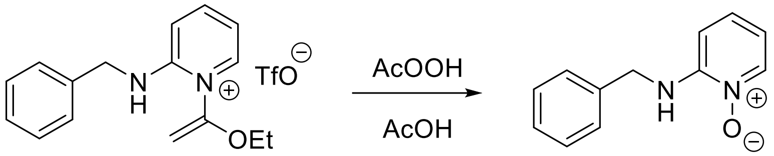

2a **7a**

A solution of compound **2a** (110 mg, 0.27 mmol) in 30% peracetic acid in acetic acid solution (3.2 mL, 15 mmol of AcOOH) was stirred in a 10 mL round-bottom flask overnight for 18 hours. The reaction mixture was slowly poured into a rapidly stirring solution of $NaHCO_{3\,(aq)}$ (50 mL) and $Na_2S_2O_{3\,(aq)}$ (50 mL) in order to neutralize residual AcOH and AcOOH, respectively. The aqueous mixture was extracted with EtOAc (3 x 25 mL), and the combined organic layer washed with water (10 mL) then brine (10 mL). The combined organic layer was dried over sodium sulfate, filtered, and concentrated to produce a residue that was purified by automated column chromatography with a Teledyne ISCO CombiFlash system (0-100% chloroform/isopropanol gradient) to yield the *N*-oxide product **7a** as a yellow amorphous solid (44 mg, 80%). The product NMR and mass spectra matched those previously reported.[6]

2-(1,3-dimethoxy-1,3-dioxopropan-2-yl)-1-(1-ethoxyvinyl)pyridin-1-ium trifluoromethanesulfonate (**8**)

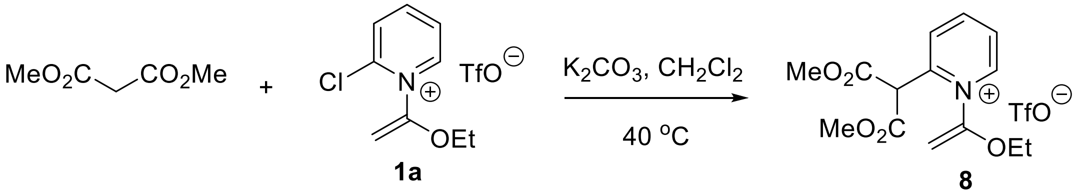

1a **8**

A 0.5-2.0 mL Biotage microwave vial was charged with 1-(1-ethoxyvinyl)-2-chloropyridinium triflate **1a** (100 mg, 0.30 mmol), 1 mL of dichloromethane, dimethylmalonate (35 µL, 0.30 mmol), and then anhydrous potassium carbonate (42 mg, 0.30 mmol). The resulting suspension was stirred for one minute before sealing with a crimped septum cap and placing into a pre-heated 40 °C oil bath for 24h. Before uncapping the vial, the reaction was cooled to room temperature and then the septum was punctured with a needle to release pressure. The reaction mixture was then transferred by pipette to a round-bottom flask using acetone to transfer over any remaining organic soluble residue. The resulting solution was concentrated in vacuo to yield a residue that was purified by automated column chromatography with a Teledyne ISCO CombiFlash system (0-100% chloroform/isopropanol gradient) to yield product **8** as a pale yellow amorphous solid (54 mg, 42%). ^1H NMR (500 MHz, Acetone-d_6) δ 8.13 (dd, *J* = 6.5, 2.2 Hz, 1H), 7.86 (ddd, *J* = 8.9, 1.5, 0.8 Hz, 1H), 7.77 (ddd, *J* = 8.8, 7.0, 1.7 Hz, 1H), 7.08 (td, *J* = 6.9, 1.5 Hz, 1H), 4.35 (d, *J* = 4.2 Hz, 1H), 4.28 (d, *J* = 4.2 Hz, 1H), 3.85 (q, *J* = 7.0 Hz, 2H), 3.38 (s, 6H), 2.78 (s, 1H), 1.19 (t, *J* = 7.0 Hz, 3H); ^{13}C NMR (126 MHz, Acetone) δ 167.5, 157.7, 156.3, 141.2, 139.9, 130.6, 121.5 (q, $^1J_{CF}$ = 322 Hz, *C*F_3) 117.2, 82.6, 66.0, 64.9, 49.4, 13.4; LRMS-ES+ *m/z* (relative intensity) 280.1 ($C_{14}H_{18}NO_5$ M+, 100); HRMS-ES+ ($C_{14}H_{18}NO_5$) calcd 280.1185 (M+), found 280.1185.

1-(1-ethoxyvinyl)-2-(octylthio)pyridin-1-ium trifluoromethanesulfonate (**9**)

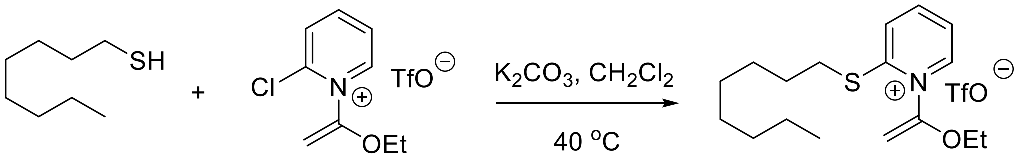

1a **9**

A 0.5-2.0 mL Biotage microwave vial was charged with 1-(1-ethoxyvinyl)-2-chloropyridinium triflate **1a** (167 mg, 0.50 mmol), 1 mL of dichloromethane, 1-octanethiol (87 µL, 0.50 mmol), and then anhydrous potassium carbonate (69 mg,

0.50 mmol). The resulting suspension was stirred for one minute before sealing with a crimped septum cap and placing into a pre-heated 40 °C oil bath for 24h. Before uncapping the vial, the reaction was cooled to room temperature and then the septum was punctured with a needle to release pressure. The reaction mixture was then transferred by pipette to a round-bottom flask using acetone to transfer over any remaining organic soluble residue. The resulting solution was concentrated in vacuo to yield a residue that was purified by automated column chromatography with a Teledyne ISCO CombiFlash system (0-70% chloroform/isopropanol gradient) to yield product **9** as a pale yellow amorphous solid (157 mg, 71%). ^{1}H NMR (500 MHz, $CDCl_3$) δ 8.69 (dd, J = 6.4, 1.6 Hz, 1H), 8.48 (ddd, J = 9.0, 7.5, 1.6 Hz, 1H), 8.06 (d, J = 8.6 Hz, 1H), 7.78 (ddd, J = 7.5, 6.1, 1.2 Hz, 1H), 4.71 (q, J = 5.2 Hz, 2H), 4.12 (q, J = 7.0 Hz, 2H), 3.29 (t, J = 7.3 Hz, 2H), 1.77 (p, J = 7.4 Hz, 2H), 1.46 (tt, J = 8.1, 6.0 Hz, 2H), 1.40 (t, J = 7.0 Hz, 3H), 1.35 – 1.17 (m, 9H), 0.85 (t, J = 7.0 Hz, 3H); ^{13}C NMR (126 MHz, $CDCl_3$) δ 161.4, 152.8, 146.1, 145.5, 125.9, 120.8 (q, $^1J_{CF}$ = 321 Hz, CF_3), 122.5, , 87.4, 67.2, 33.6, 31.6, 28.9, 28.9, 28.5, 27.55, 22.54, 14.0, 13.8; LRMS-ES+ *m/z* (relative intensity) 294.2 ($C_{17}H_{28}NOS$ M+, 100); HRMS-ES+ ($C_{17}H_{28}NOS$) calcd 294.1892 (M+), found 294.1898.

1-(1-ethoxyvinyl)-2-(pent-4-yn-1-yloxy)pyridin-1-ium trifluoromethanesulfonate (**10**)

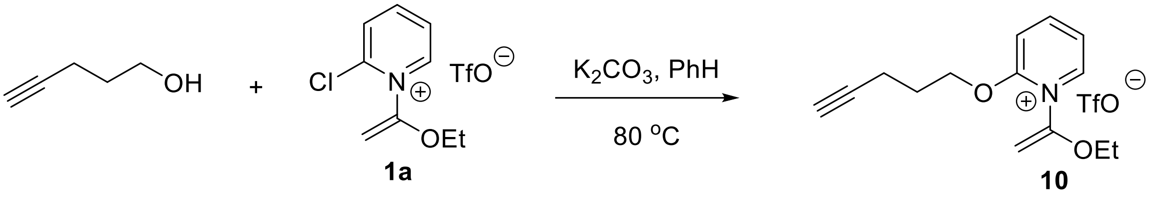

A 0.5-2.0 mL Biotage microwave vial was charged with 1-(1-ethoxyvinyl)-2-chloropyridinium triflate **1a** (167 mg, 0.50 mmol), 1 mL of benzene, 4-pentyn-1-ol (47 μL, 0.50 mmol), and then anhydrous potassium carbonate (69 mg, 0.50 mmol). The resulting suspension was stirred for one minute before sealing with a crimped septum cap and placing into a pre-heated 80 °C oil bath for 24h. Before uncapping the vial, the reaction was cooled to room temperature and then the septum was punctured with a needle to release pressure. The reaction mixture was then transferred by pipette to a round-bottom flask using acetone to transfer over any remaining organic soluble residue. The resulting solution was concentrated in vacuo to yield a residue that was purified by automated column chromatography with a Teledyne ISCO CombiFlash system (0-70% chloroform/isopropanol gradient) to yield product **10** as a clear oil (76 mg, 40%). ^{1}H NMR (500 MHz, $CDCl_3$) δ 8.53 (t, J = 8.0 Hz, 1H), 8.24 (d, J = 6.1 Hz, 1H), 7.75 (d, J = 8.7 Hz, 1H), 7.53 (t, J = 6.8 Hz, 1H), 4.75 – 4.61 (m, 3H), 4.55 (d, J = 4.7 Hz, 1H), 4.02 (q, J = 7.0 Hz, 2H), 2.32 (dt, J = 6.9, 3.5 Hz, 2H), 2.06 (p, J = 6.2 Hz, 2H), 1.95 (t, J = 2.6 Hz, 1H), 1.35 (t, J = 7.0 Hz, 3H); ^{13}C NMR (126 MHz, $CDCl_3$) δ 159.4, 151.4, 151.1, 142.0, 120.6 (q, J = 320 Hz), 119.2, 112.8, 86.5, 81.8, 72.1, 70.2, 66.9, 27.1, 14.7, 14.0; LRMS-ES+ *m/z* (relative intensity) 232.1 ($C_{14}H_{18}NO_2$ M+, 100); HRMS-ES+ ($C_{14}H_{18}NO_2$) calcd 232.1338 (M+), found 232.1334.

1-(1-ethoxyvinyl)-2-(1*H*-indol-3-yl)pyridin-1-ium trifluoromethanesulfonate (**11**)

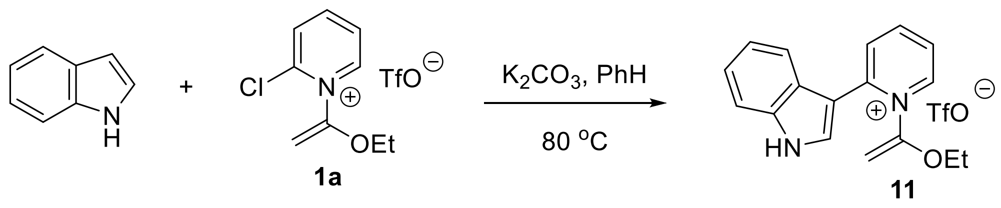

A 0.5-2.0 mL Biotage microwave vial was charged with 1-(1-ethoxyvinyl)-2-chloropyridinium triflate **1a** (167 mg, 0.50 mmol), 1 mL of benzene, indole (59 mg, 0.50 mmol), and then anhydrous potassium carbonate (69 mg, 0.50 mmol). The resulting suspension was stirred for one minute before sealing with a crimped septum cap and placing into a pre-heated 80 °C oil bath for 24h. Before uncapping the vial, the reaction was cooled to room temperature and then the septum was punctured with a needle to release pressure. The reaction mixture was then transferred by pipette to a round-bottom flask using acetone to transfer over any remaining organic soluble residue. The resulting solution was concentrated in vacuo to yield a residue that was purified by automated column chromatography with a Teledyne ISCO CombiFlash system (0-100% chloroform/isopropanol gradient) to yield product **11** as a pale yellow amorphous solid (99 mg, 48%). ^{1}H NMR (500 MHz, Acetone-d_6) δ 11.59 (s, 1H), 9.04 (dd, J = 6.5, 1.5 Hz, 1H), 8.75 (td, J = 7.9, 1.6 Hz, 1H), 8.45 (dd, J = 8.4, 1.4 Hz, 1H),

8.16 (d, J = 3.1 Hz, 1H), 8.05 (ddd, J = 7.6, 6.4, 1.4 Hz, 1H), 7.85 (d, J = 8.0 Hz, 1H), 7.73 – 7.68 (m, 1H), 7.37 – 7.30 (m, 1H), 7.28 (ddd, J = 8.2, 7.1, 1.2 Hz, 1H), 4.92 (d, J = 4.6 Hz, 1H), 4.76 (d, J = 4.7 Hz, 1H), 4.07 (q, J = 7.0 Hz, 2H), 1.18 (t, J = 7.0 Hz, 3H); ^{13}C NMR (126 MHz, Acetone-d$_6$) δ 155.0, 151.0, 146.9, 145.9, 136.6, 129.7, 129.4, 125.2, 123.7, 123.6, 122.0, 121.2 (q, $^1J_{CF}$ = 322 Hz, CF$_3$), 118.6, 113.0, 106.8, 86.3, 66.8, 13.2; LRMS-ES+ *m/z* (relative intensity) 265.1 (($C_{17}H_{17}N_2O$ M+, 100); HRMS-ES+ ($C_{17}H_{17}N_2O$) calcd 265.1341 (M+), found 265.1333.

3-(pyridin-2-yl)-1*H*-indole (**12**)

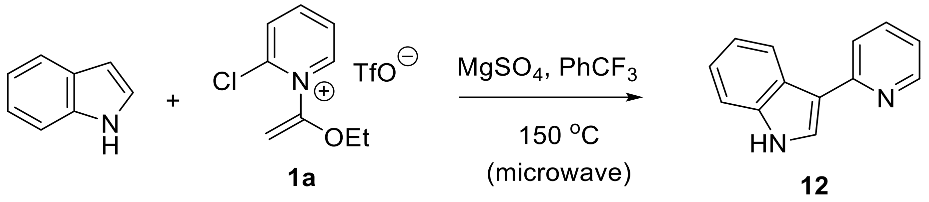

Tandem S$_N$Ar and *N*-(1-Ethoxyvinyl) Cleavage Procedure 2C (Method 3) was followed using indole (35 mg, 0.30 mmol) as the nucleophile. Purification by automated column chromatography with a Teledyne ISCO CombiFlash system (0-70% hexanes/ethyl acetate gradient) yielded product **12** as a white amorphous solid (49 mg, 84%). Spectral data of product **12** matched those previously reported.[7]

N,*N*-dimethyl-4-(pyridin-2-yl)aniline (**13**)

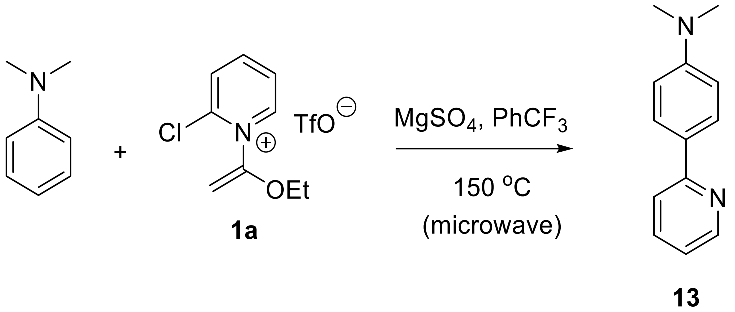

Tandem S$_N$Ar and *N*-(1-Ethoxyvinyl) Cleavage Procedure 2C (Method 3) was followed using aniline (38 μL, 0.30 mmol) as the nucleophile. Purification by automated column chromatography with a Teledyne ISCO CombiFlash system (0-50% hexanes/ethyl acetate gradient) yielded product **13** as a clear oil (18 mg, 30%). Spectral data of product **13** matched those previously reported.[8]

1-(1-ethoxyvinyl)-4-(1-methoxy-2-methyl-1-oxopropan-2-yl)pyridin-1-ium trifluoromethanesulfonate (**14**)

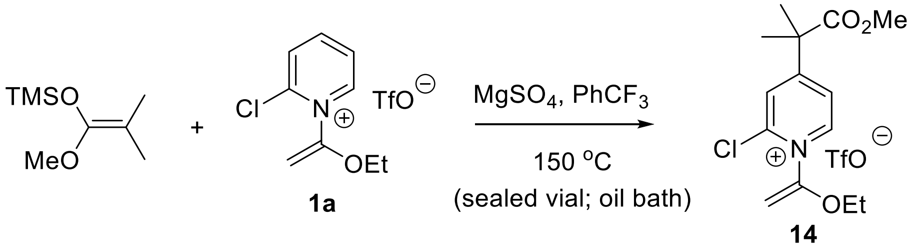

To a flame-dried 2-5 mL Biotage microwave vial containing a stir bar was added 1-(1-ethoxyvinyl)-2-chloropyridinium triflate **1a** (300 mg, 0.90 mmol), α,α,α-trifluorotoluene (2 mL), and 1-methoxy-2-methyl-1-(trimethylsiloxy)propene (64 μL, 0.32 mmol). The vial was sealed with a crimped septum then stirred in a pre-heated oil bath at 150 °C for 1h. (**Caution!** The reaction generates significant pressure since it is being heated beyond the solvent's boiling point. Although the Biotage microwave vails are made to withstand this pressure, additional precautions should be taken such as use of a face shield mask and blast shield). Following this, the reaction is cooled to room temperature and the septum is punctured with a needle to release pressure prior to uncapping the vial. The reaction mixture was then transferred by pipette to a round-bottom flask using acetone to transfer over any remaining organic soluble residue. The resulting

solution was concentrated in vacuo to yield a residue that was purified by automated column chromatography with a Teledyne ISCO CombiFlash system (0-100% chloroform/isopropanol gradient; 4g RediSep Gold® pre-packed silica gel cartridge) to yield product **14** as a pale yellow amorphous solid (62 mg, 45%). ^{1}H NMR (500 MHz, Acetone-d$_6$) δ 9.23 (d, *J* = 6.7 Hz, 1H), 8.49 (d, *J* = 2.1 Hz, 1H), 8.30 (ddd, *J* = 6.6, 2.0, 1.0 Hz, 1H), 5.06 (d, *J* = 4.9 Hz, 1H), 5.01 (d, *J* = 5.0 Hz, 1H), 4.27 (q, *J* = 7.0 Hz, 2H), 3.73 (s, 3H), 1.74 (s, 6H), 1.40 (t, *J* = 7.0 Hz, 3H); ^{13}C NMR (126 MHz, Acetone-d$_6$) δ 174.4, 169.7, 154.2, 148.4, 147.4, 129.24, 126.16, 122.2 (q, $^{1}J_{CF}$ = 322 Hz, *C*F$_3$), 88.3, 68.5, 53.7, 49.5, 30.5, 30.4, 30.2, 30.1, 29.9, 29.7, 29.6, 26.1, 14.3; LRMS-ES+ *m/z* (relative intensity) 284.1 ($C_{14}H_{19}NO_3Cl$ M+, 100); HRMS-ES+ ($C_{14}H_{19}NO_3Cl$) calcd 284.1053 (M+), found 284.1054.

2-benzyl-1-(1-ethoxyvinyl)pyridin-1-ium trifluoromethanesulfonate (**15**)

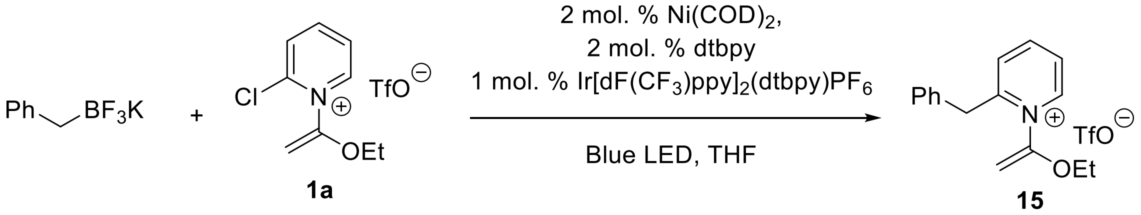

An oven-dried Schlenk flask was charged with (Ir[dF(CF$_3$)ppy]$_2$(dtbpy))PF$_6$ (11.2 mg, 0.01 mmol), potassium benzyltrifluoroborate (297mg, 1.5 mmol), 2-chloro-1-(1-ethoxyvinyl)pyridinium triflate (**1a**) (333 mg, 1.0 mmol), 4,4'-di-*tert*-butyl-2,2'-dipyridyl (dtbpy) (5.4 mg, 0.02 mmol) and an oven-dried stir bar. The flask was then secured and purged with nitrogen. The reaction vessel was then transferred into an argon-filled glove box and bis(1,5-cyclooctadiene) nickel(0) (5.5 mg, 0.02 mmol) was added and dissolved in 20 mL of anhydrous THF. An orange solution was observed with some of the reagents remaining undissolved. The flask was secured with a septum while still in the glovebox and then transferred to a standard Schlenk line in a fume hood and set to stir under an inert atmosphere of nitrogen. The remaining solids were dissolved after stirring. While stirring, the flask was then placed into a cylindrical plastic container lined with blue LED lights and stirred for 72h at ambient temperature. The solution was then transferred from the reaction vessel to a round bottom flask rinsing with dichloromethane, then concentrated *in vacuo* to produce a residue that was purified by automated column chromatography with a Teledyne ISCO CombiFlash system (0-100% chloroform/isopropanol gradient) to yield product **15** as a pale yellow amorphous solid (183 mg, 47%). ^{1}H NMR (500 MHz, Acetone-d$_6$) δ 9.04 (dd, *J* = 6.5, 1.3 Hz, 1H), 8.66 (td, *J* = 8.0, 1.6 Hz, 1H), 8.10 (ddd, *J* = 7.8, 6.2, 1.5 Hz, 1H), 7.88 (dd, *J* = 8.2, 1.4 Hz, 1H), 7.32 – 7.26 (m, 2H), 7.26 – 7.21 (m, 3H), 4.91 (d, *J* = 4.9 Hz, 1H), 4.87 (d, *J* = 4.8 Hz, 1H), 4.54 (s, 2H), 4.12 (q, *J* = 7.0 Hz, 2H), 1.21 (t, *J* = 7.0 Hz, 3H); ^{13}C NMR (126 MHz, Acetone-d$_6$) δ 157.5, 153.5, 148.5, 146.1, 134.9, 129.7, 129.6, 129.1, 127.8, 126.3, 121.6 (app q, $^{1}J_{CF}$ = 329 Hz, *C*F$_3$) 86.65, 67.0, 38.1, 13.2; LRMS-ES+ *m/z* (relative intensity) 240.1 ($C_{16}H_{18}NO$ M+, 100); HRMS-ES+ ($C_{16}H_{18}NO$) calcd 240.1388 (M+), found 240.1389.y

IV. Images of ^{1}H and $^{13}C\{^{1}H\}$ NMR spectra of new compounds

^{1}H NMR Spectrum of **2c** (500 MHz, Acetone-d_6)

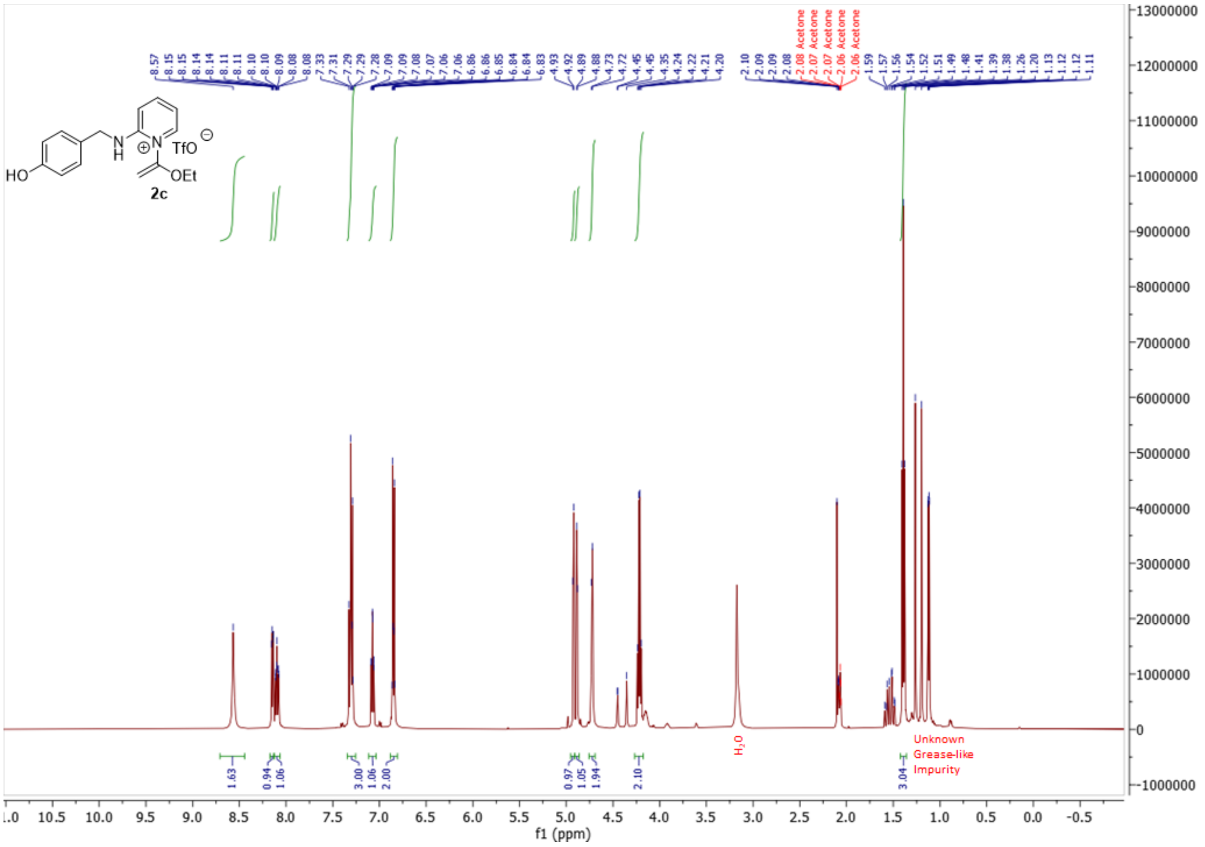

$^{13}C\{^{1}H\}$ NMR Spectrum of **2c** (126 MHz, Acetone-d_6)

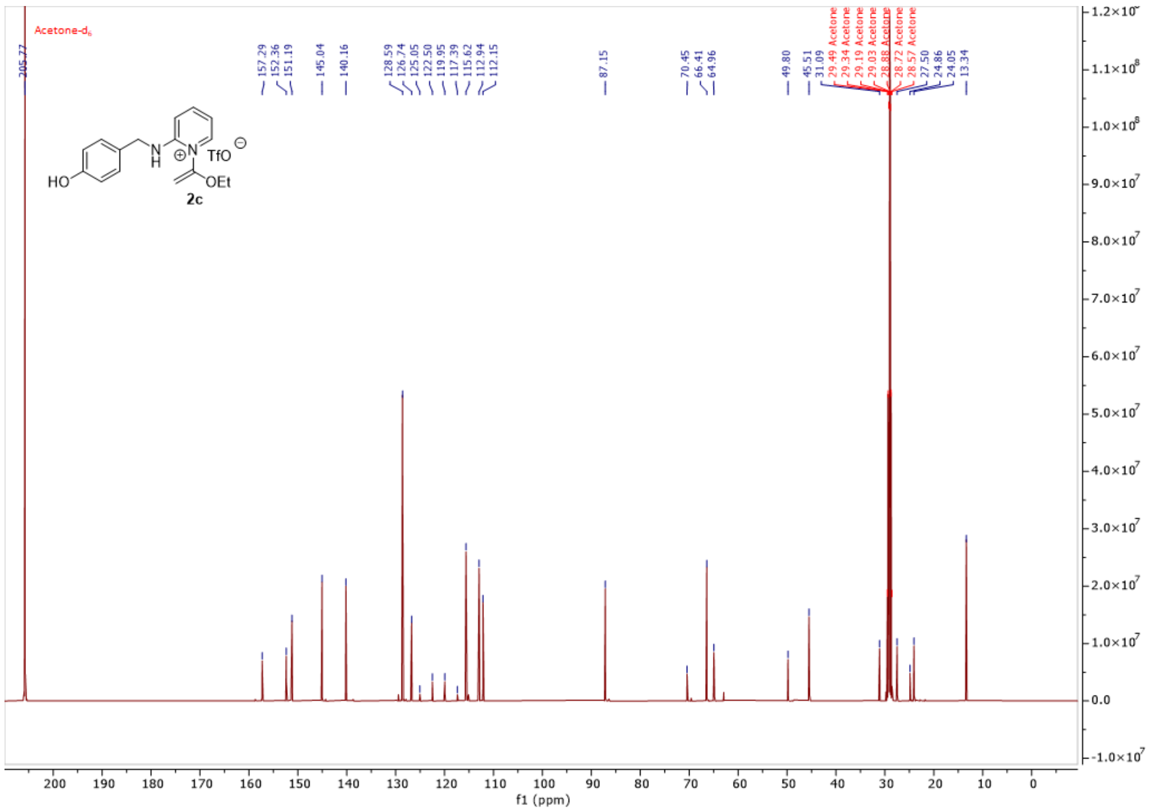

^{1}H NMR Spectrum of **2d** (500 MHz, Acetone-d_6)

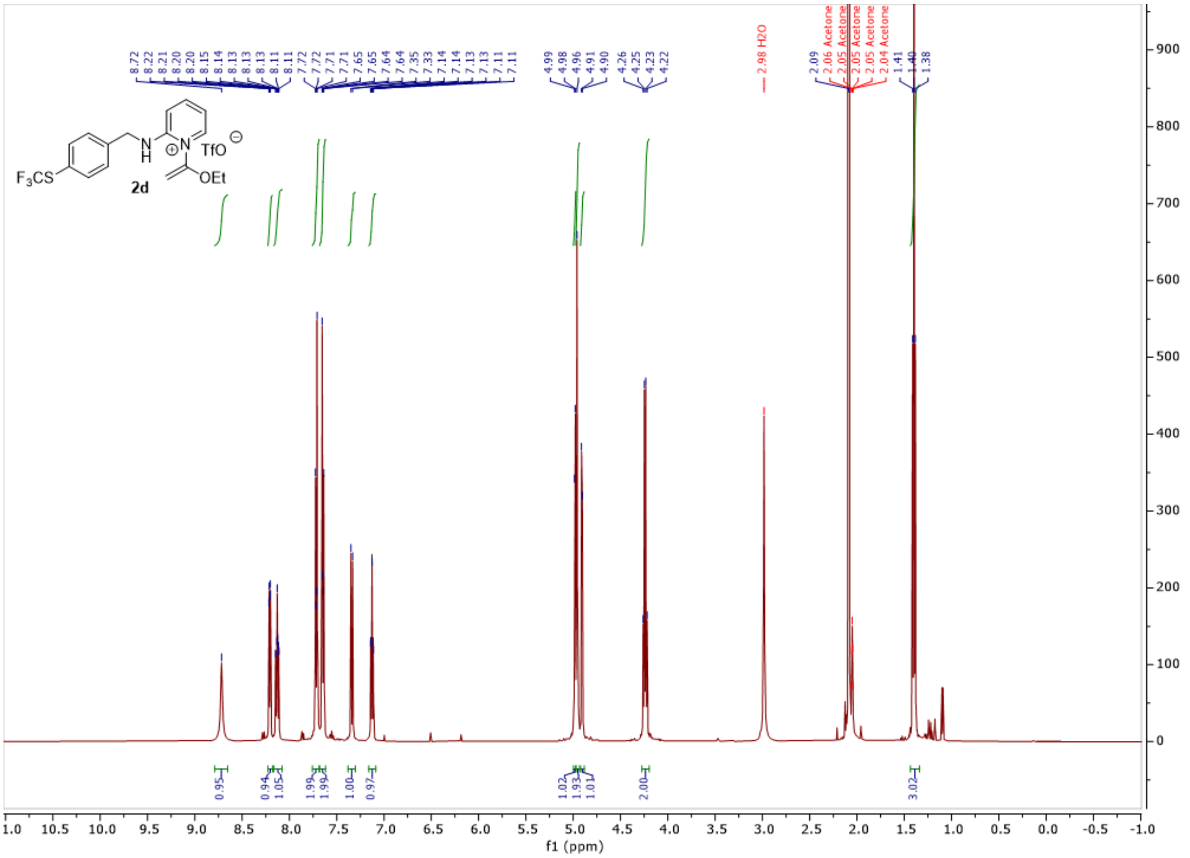

^{13}C{^{1}H} NMR Spectrum of **2d** (126 MHz, Acetone-d_6)

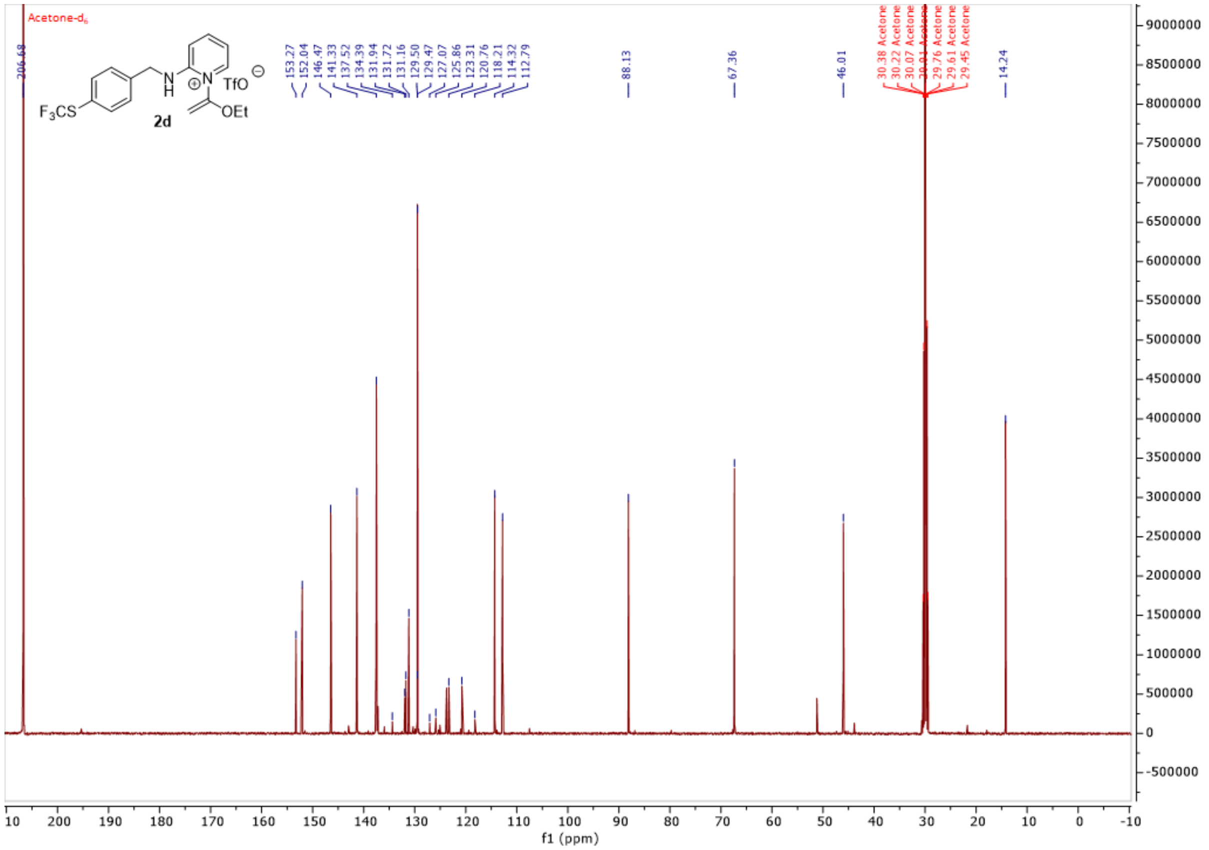

^{1}H NMR Spectrum of **2e** (500 MHz, Acetone-d_6)

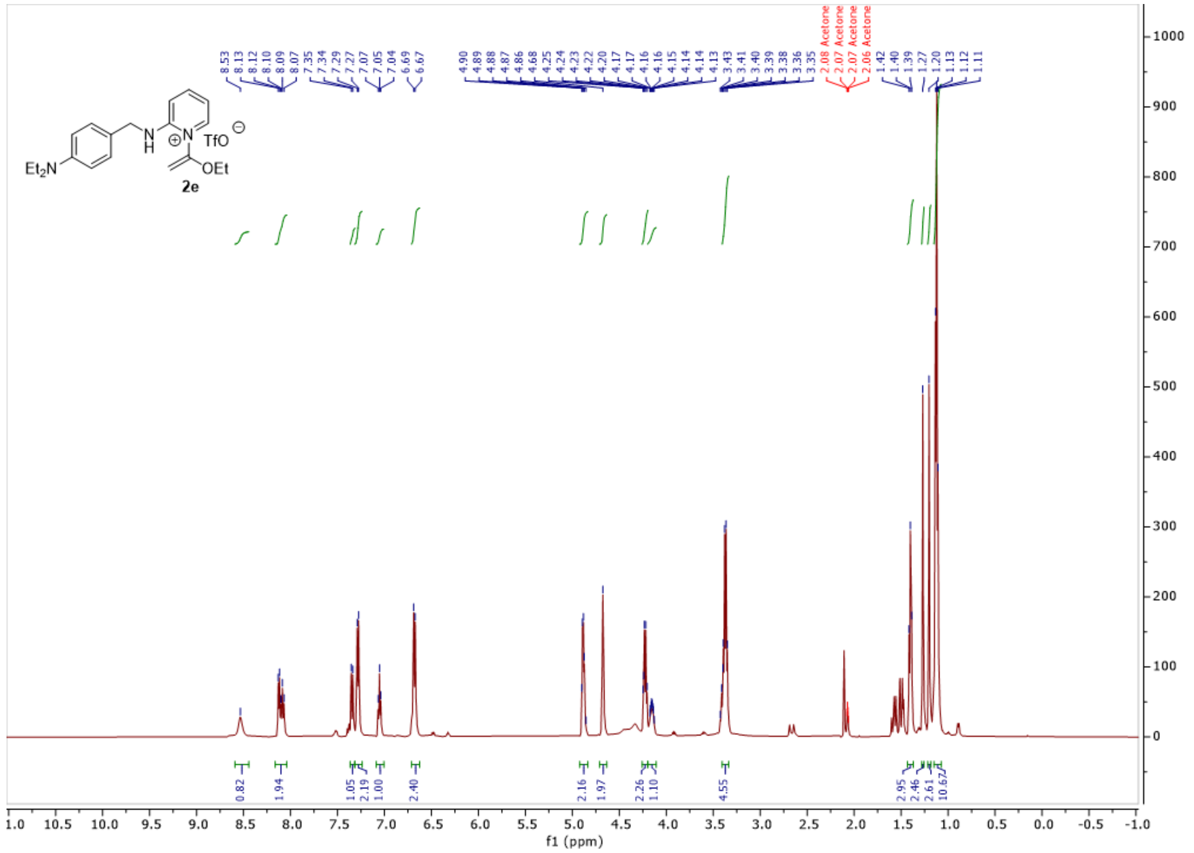

$^{13}C\{^{1}H\}$ NMR Spectrum of **2e** (126 MHz, Acetone-d_6)

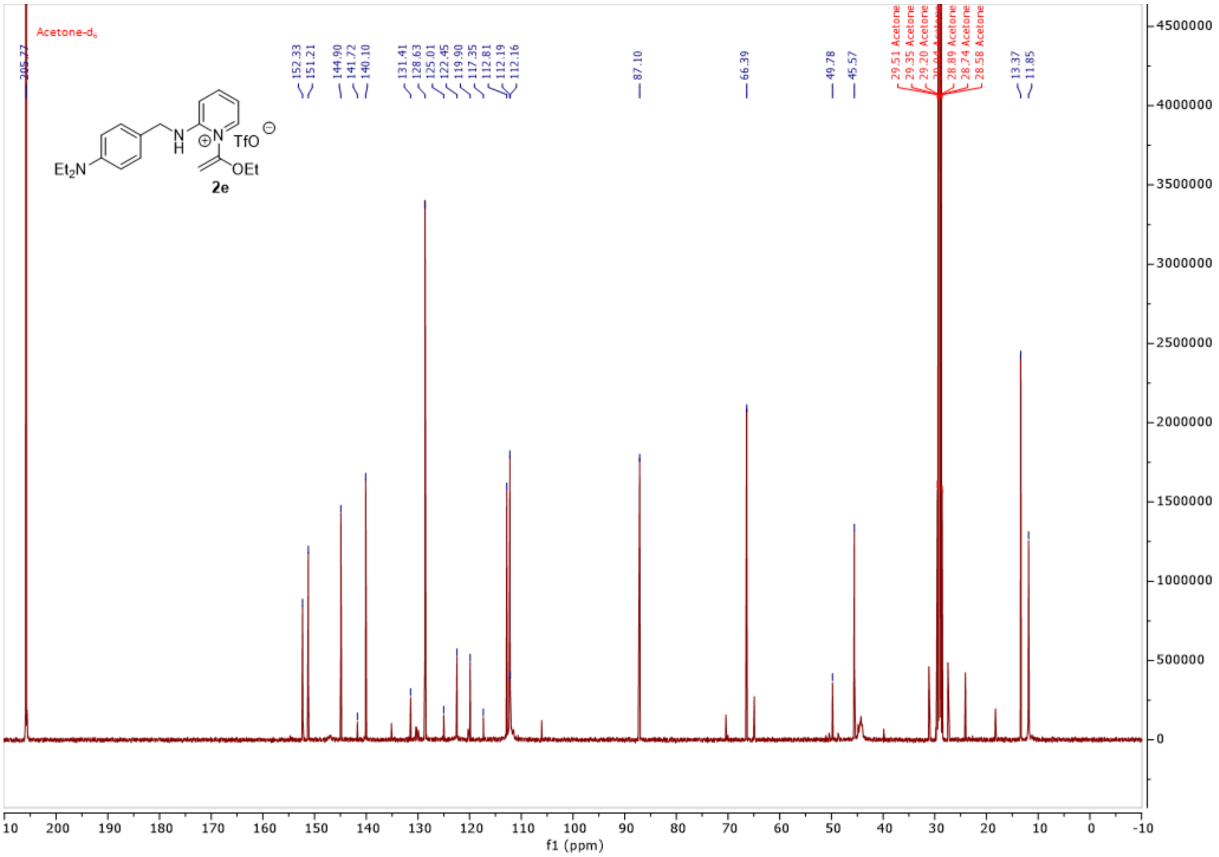

^{1}H NMR Spectrum of **2f** (500 MHz, Acetone-d_6)

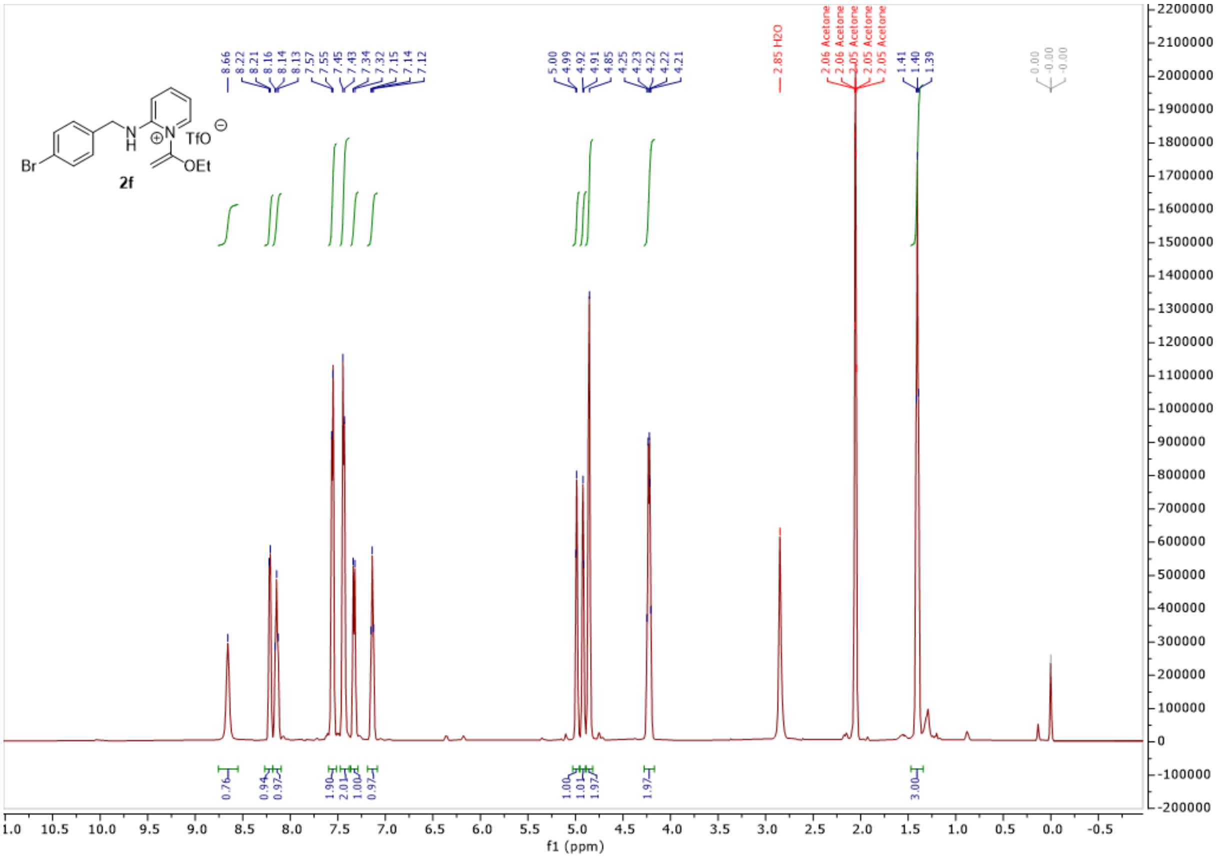

^{13}C{^{1}H} NMR Spectrum of **2f** (126 MHz, Acetone-d_6)

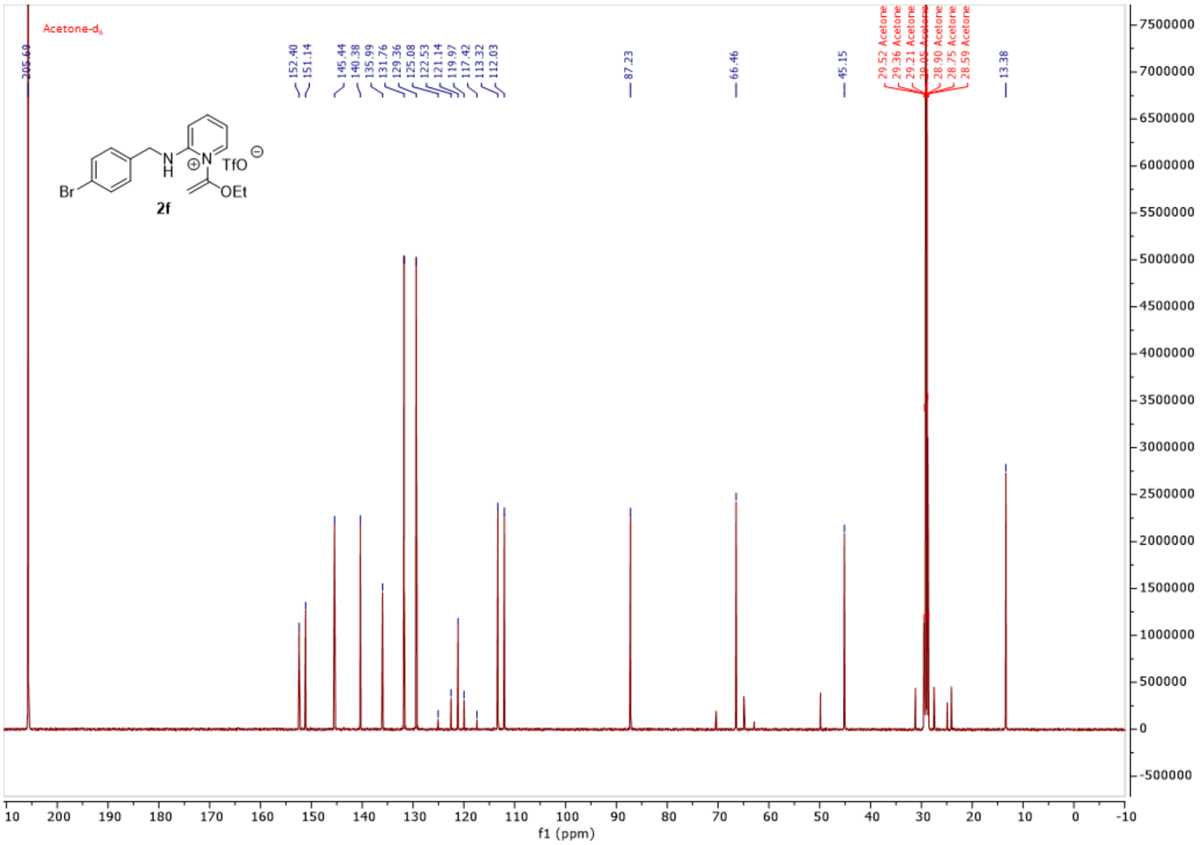

^{1}H NMR Spectrum of **2g** (500 MHz, Acetone-d_6)

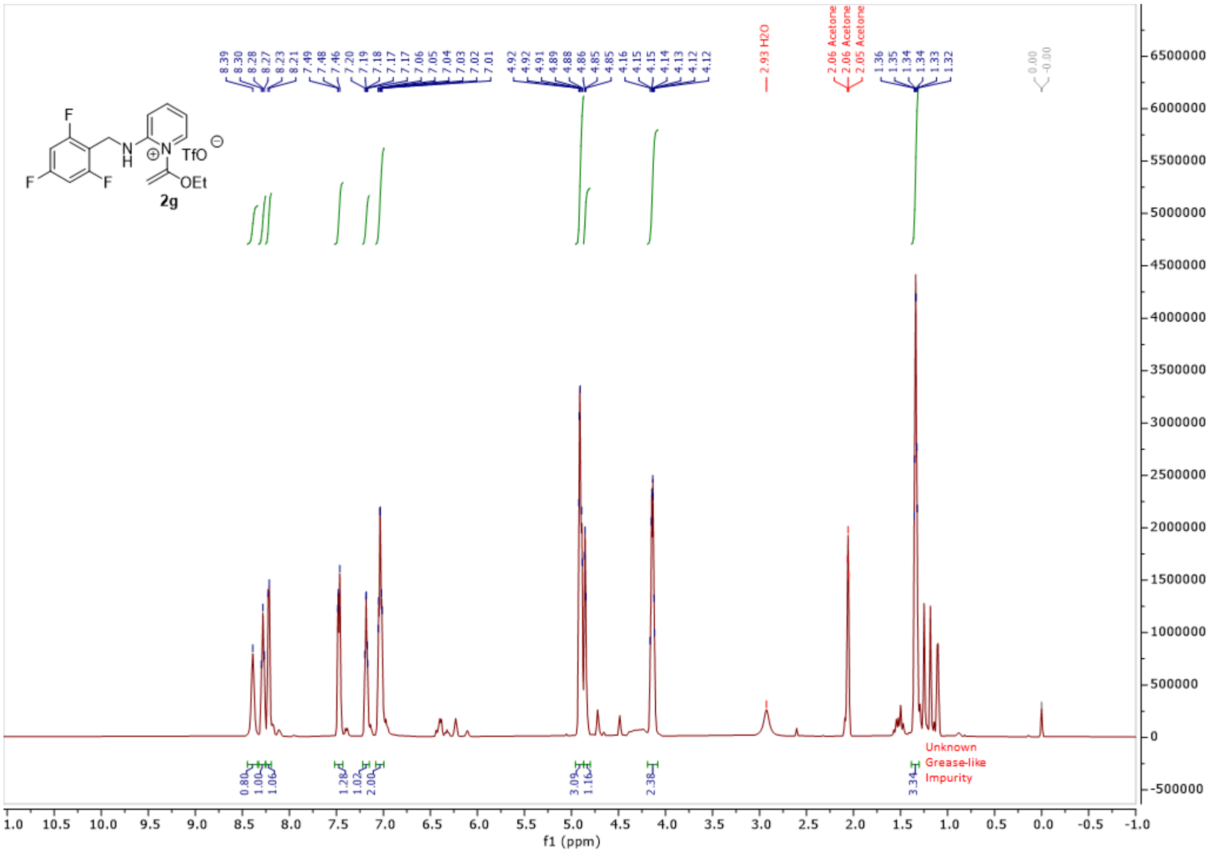

$^{13}C\{^{1}H\}$ NMR Spectrum of **2g** (126 MHz, Acetone-d_6)

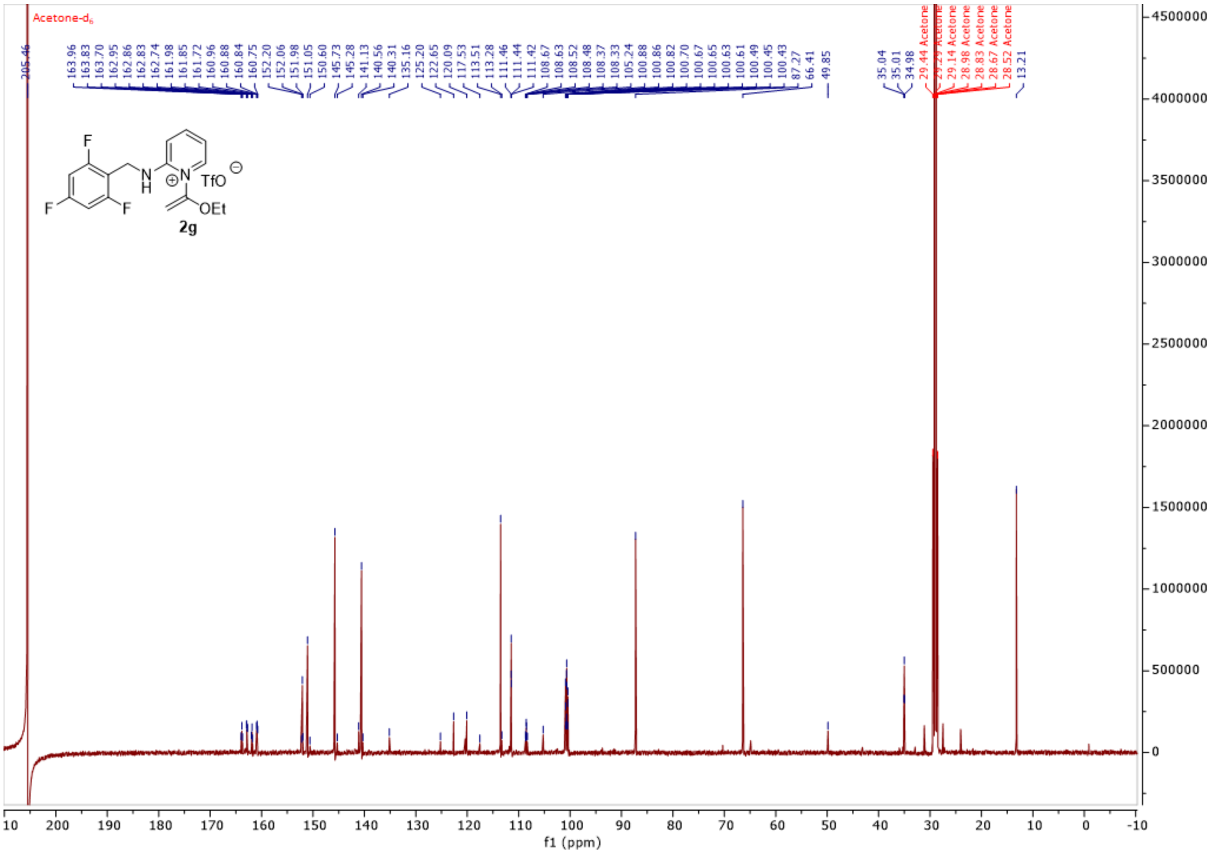

^{1}H NMR Spectrum of **2h** (500 MHz, Acetone-d_6)

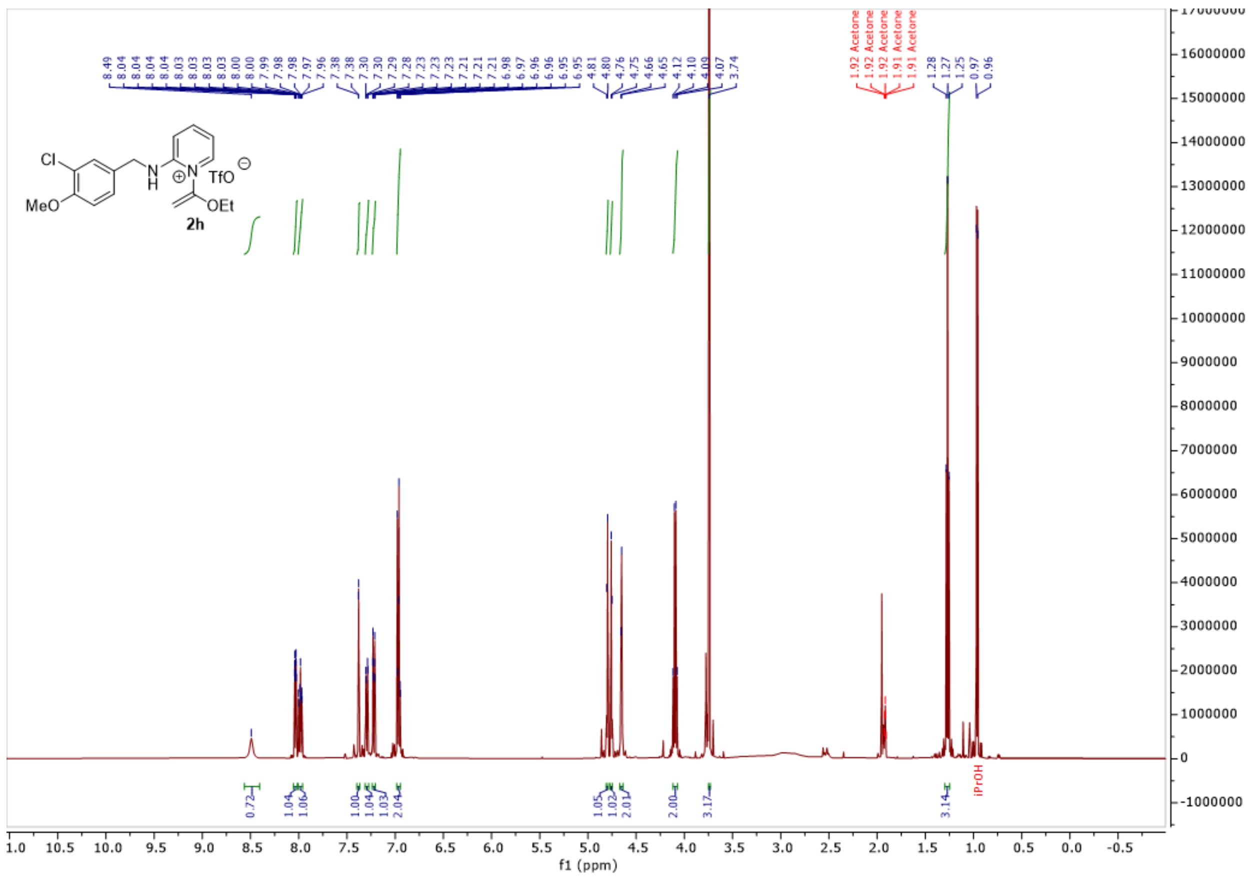

$^{13}C\{^{1}H\}$ NMR Spectrum of **2h** (126 MHz, $CDCl_3$)

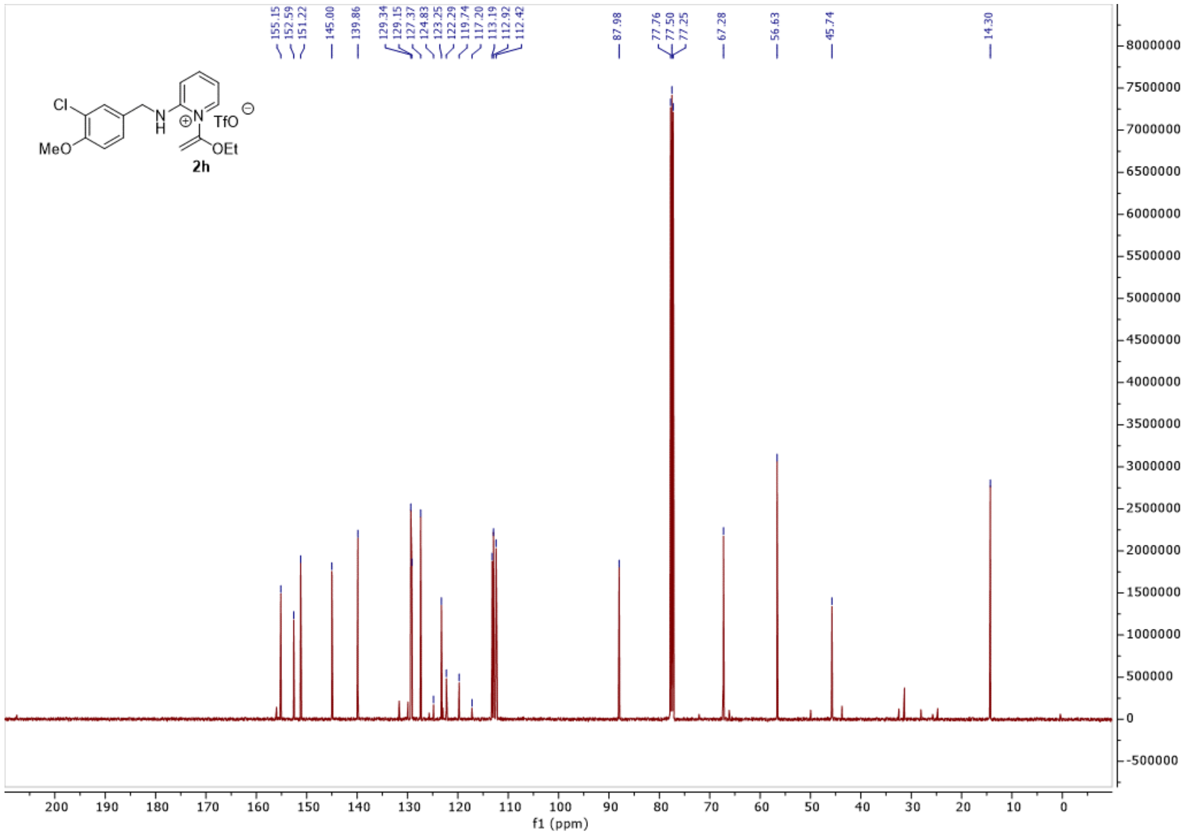

^{1}H NMR Spectrum of **2i** (500 MHz, Acetone-d_6)

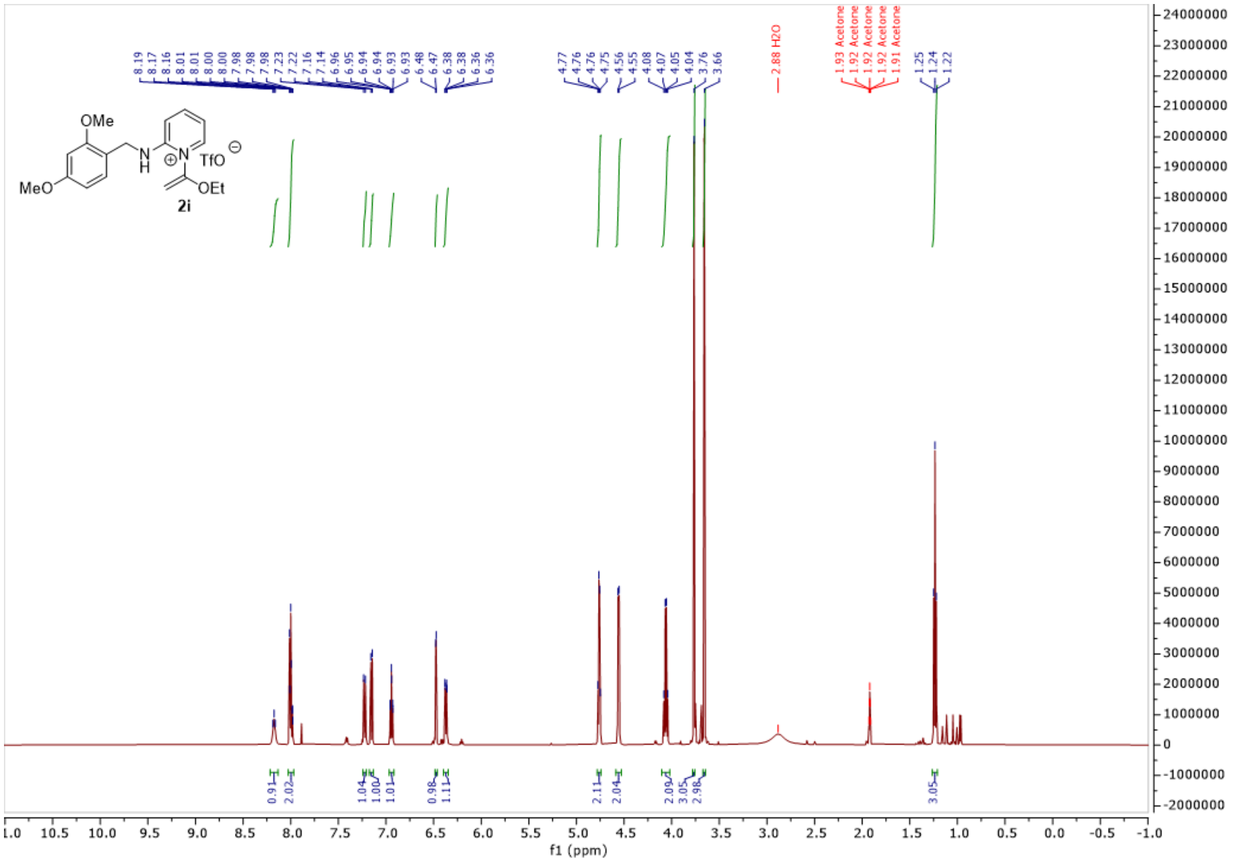

$^{13}C\{^{1}H\}$ NMR Spectrum of **2i** (126 MHz, Acetone-d_6)

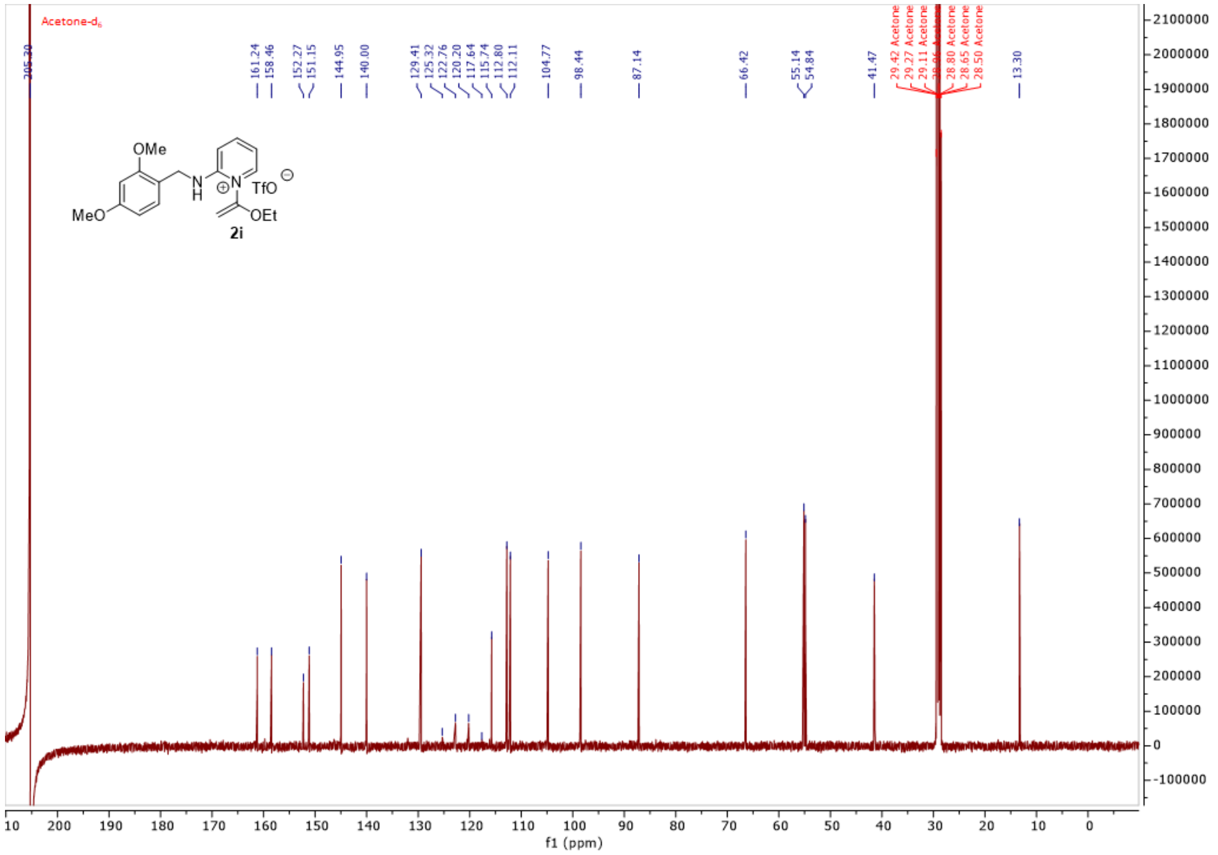

^{1}H NMR Spectrum of **2j** (500 MHz, CD_3CN)

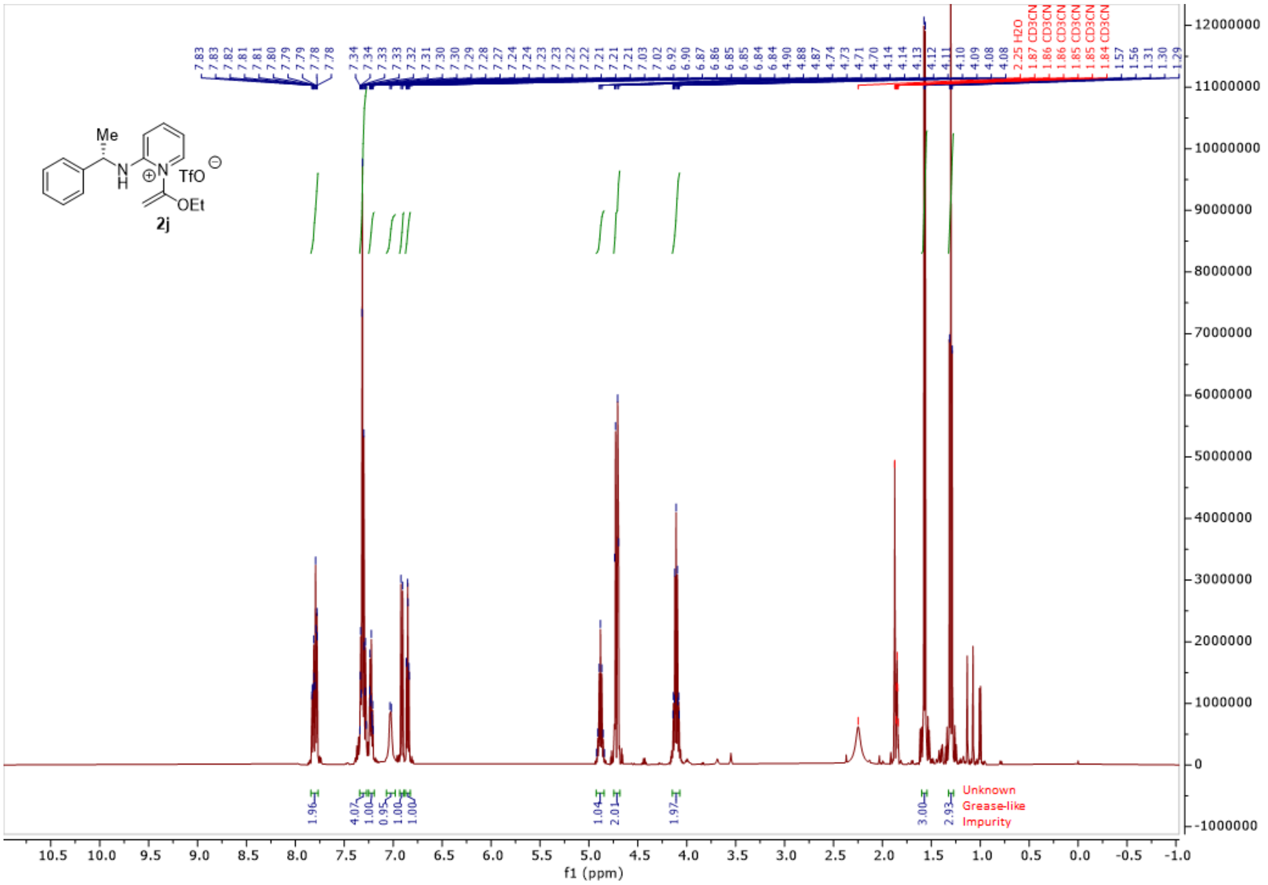

^{13}C{^{1}H} NMR Spectrum of **2j** (126 MHz, CD_3CN)

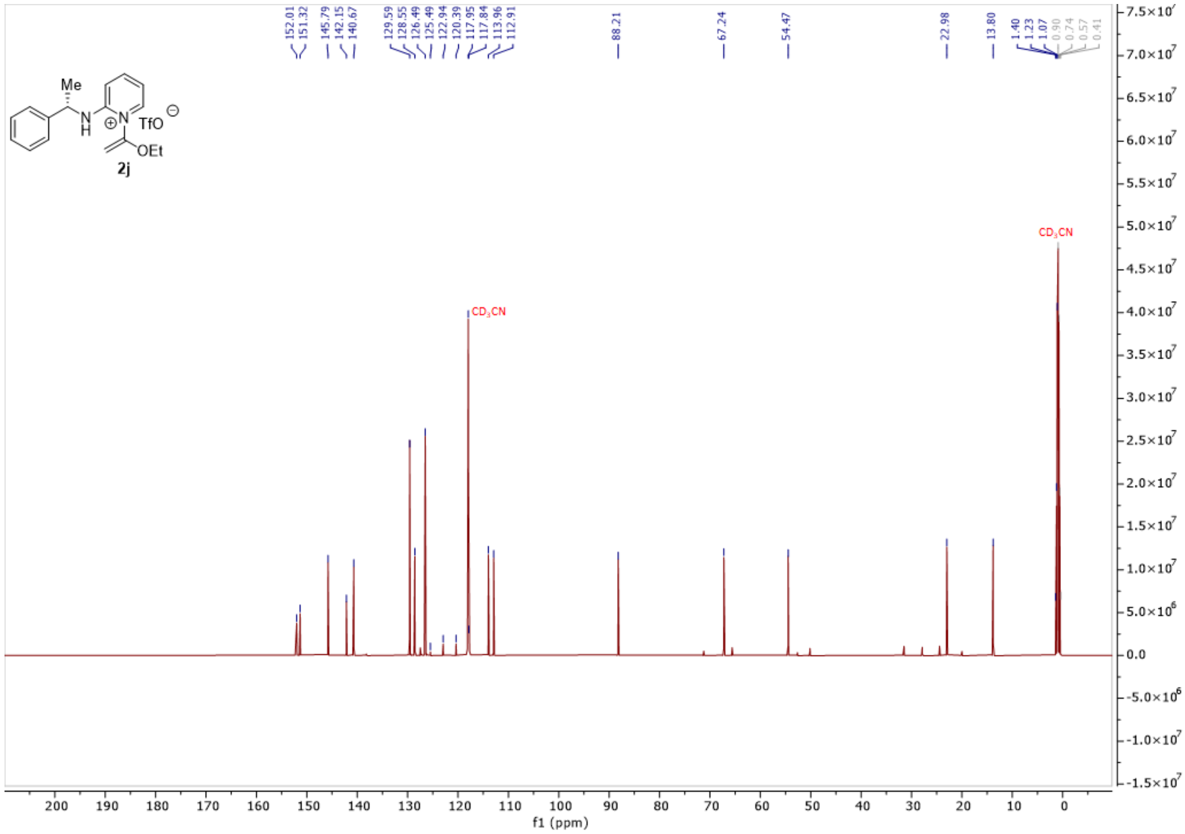

^{1}H NMR Spectrum of **2k** (500 MHz, Acetone-d_6)

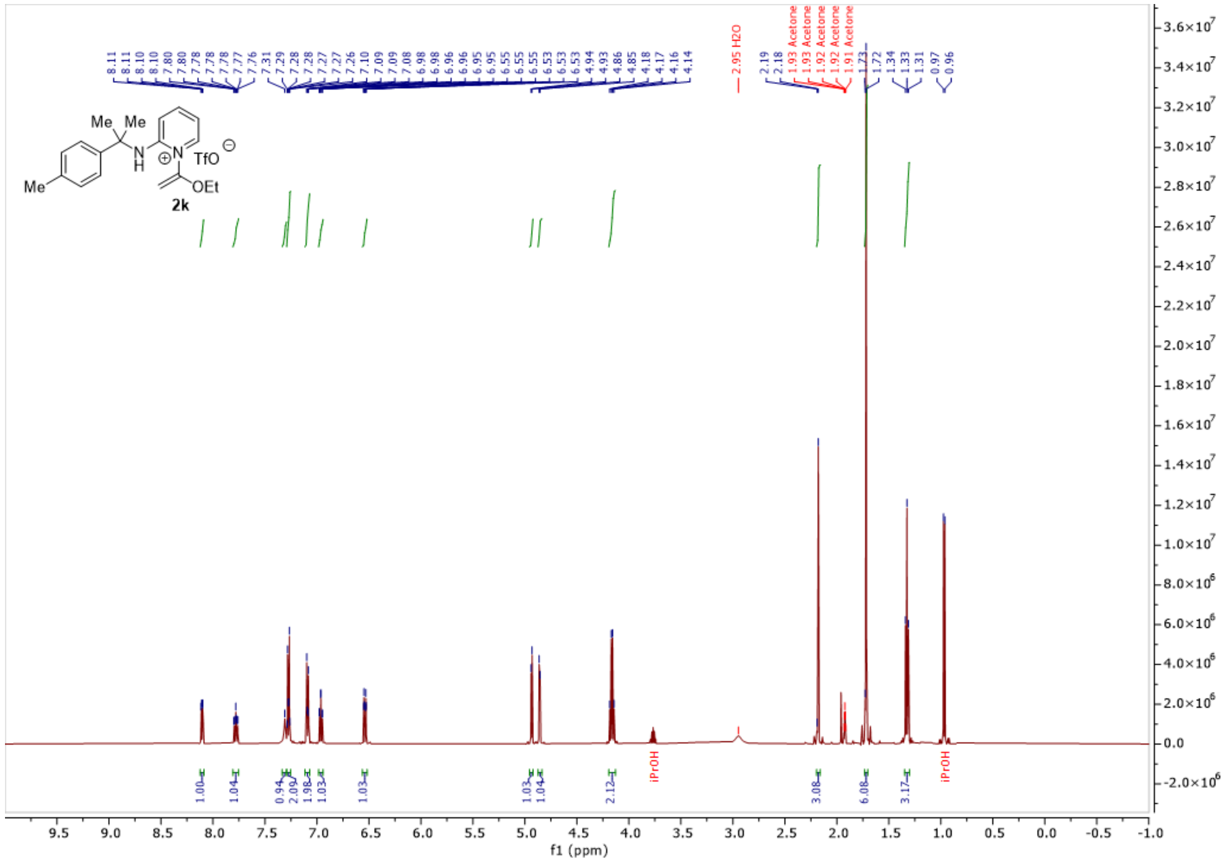

^{13}C{^{1}H} NMR Spectrum of **2k** (126 MHz, Acetone-d_6)

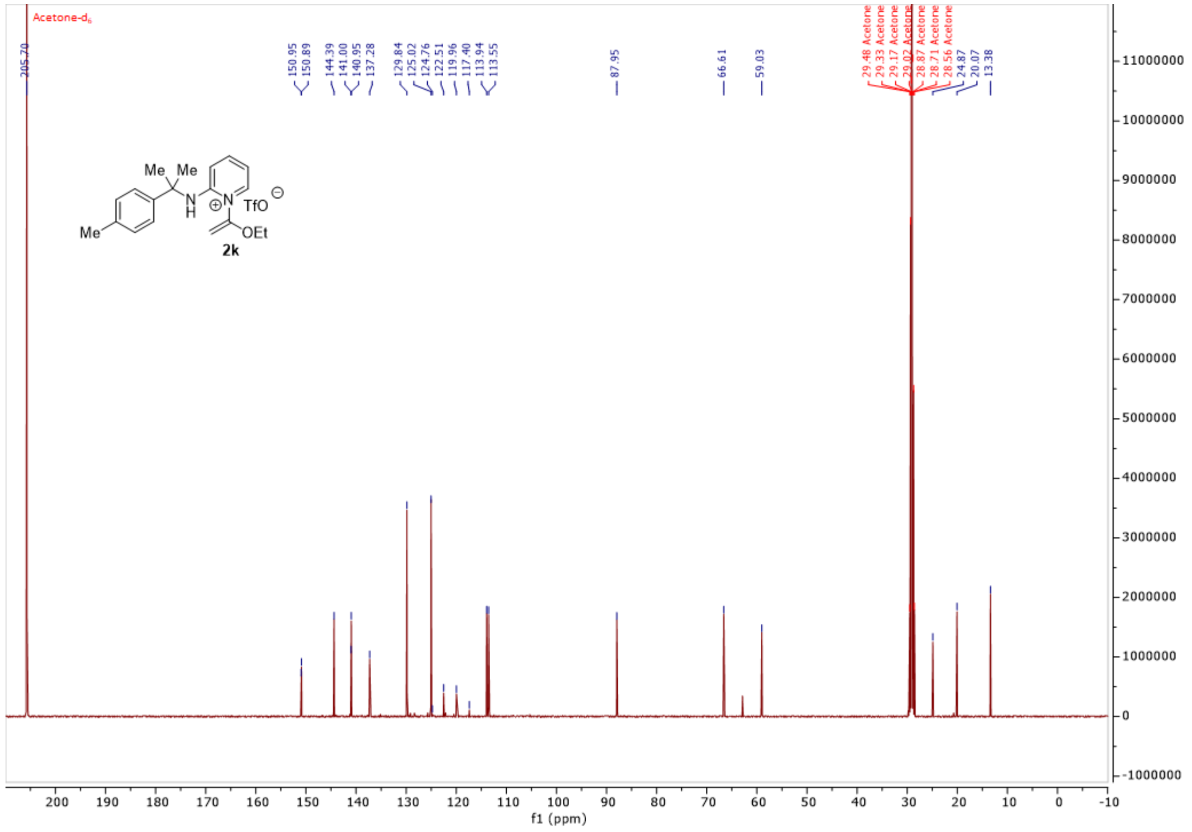

^{1}H NMR Spectrum of **2l** (500 MHz, CD_3OD)

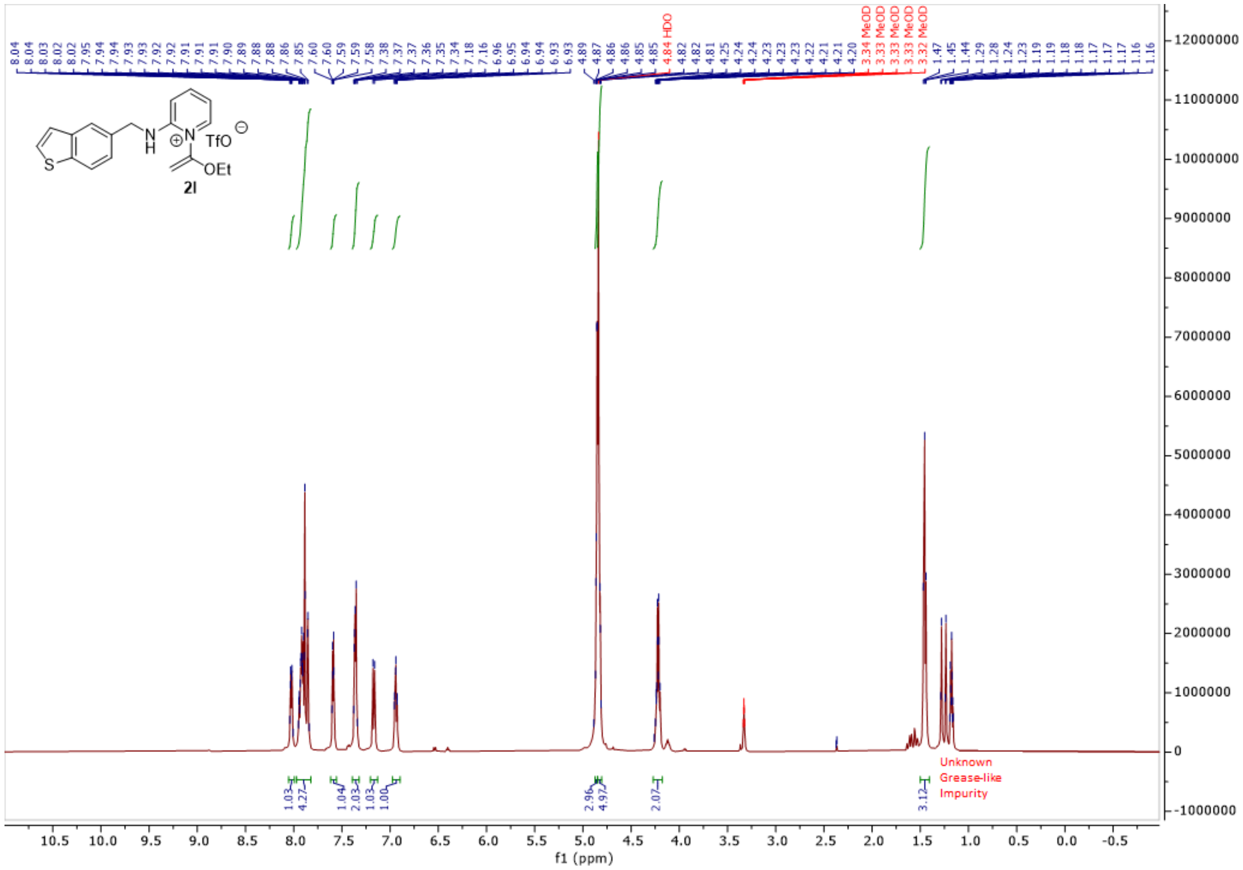

^{13}C{^{1}H} NMR Spectrum of **2l** (126 MHz, CD_3OD)

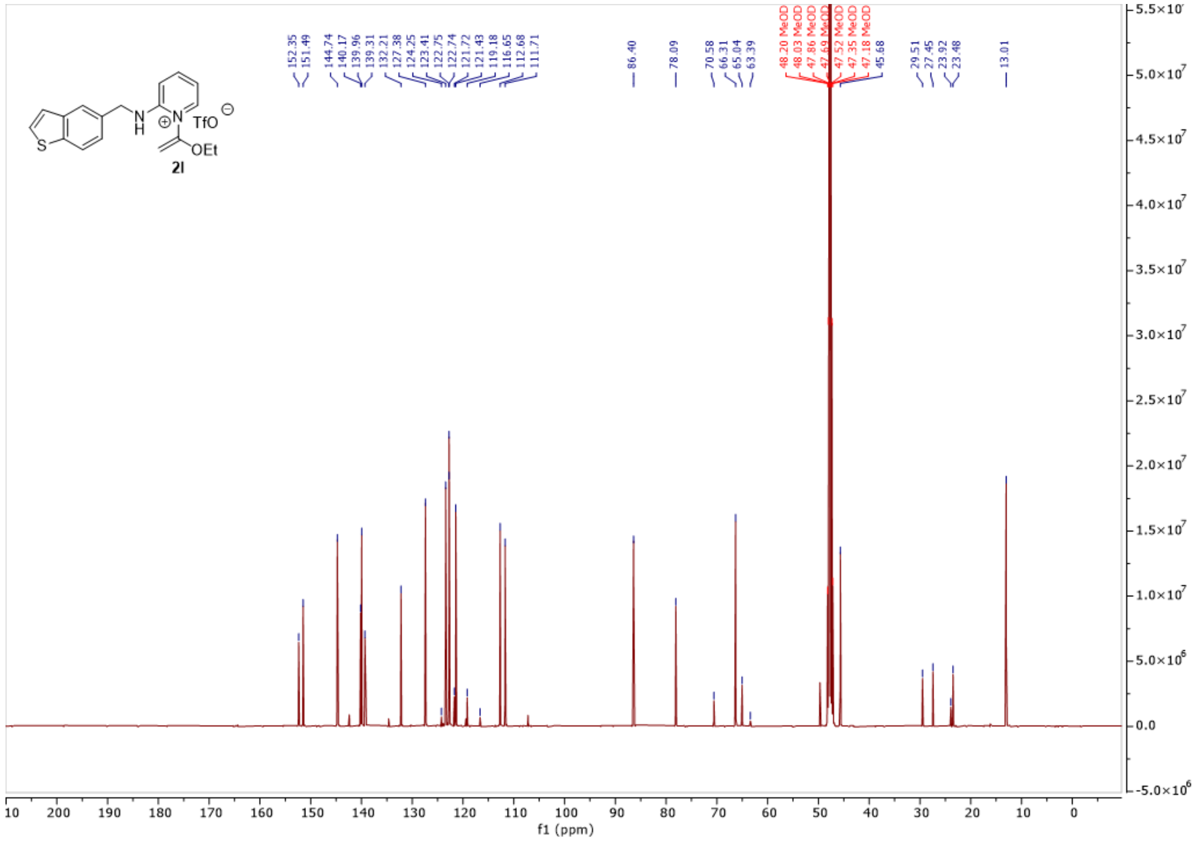

^{1}H NMR Spectrum of **2m** (500 MHz, Acetone-d_6)

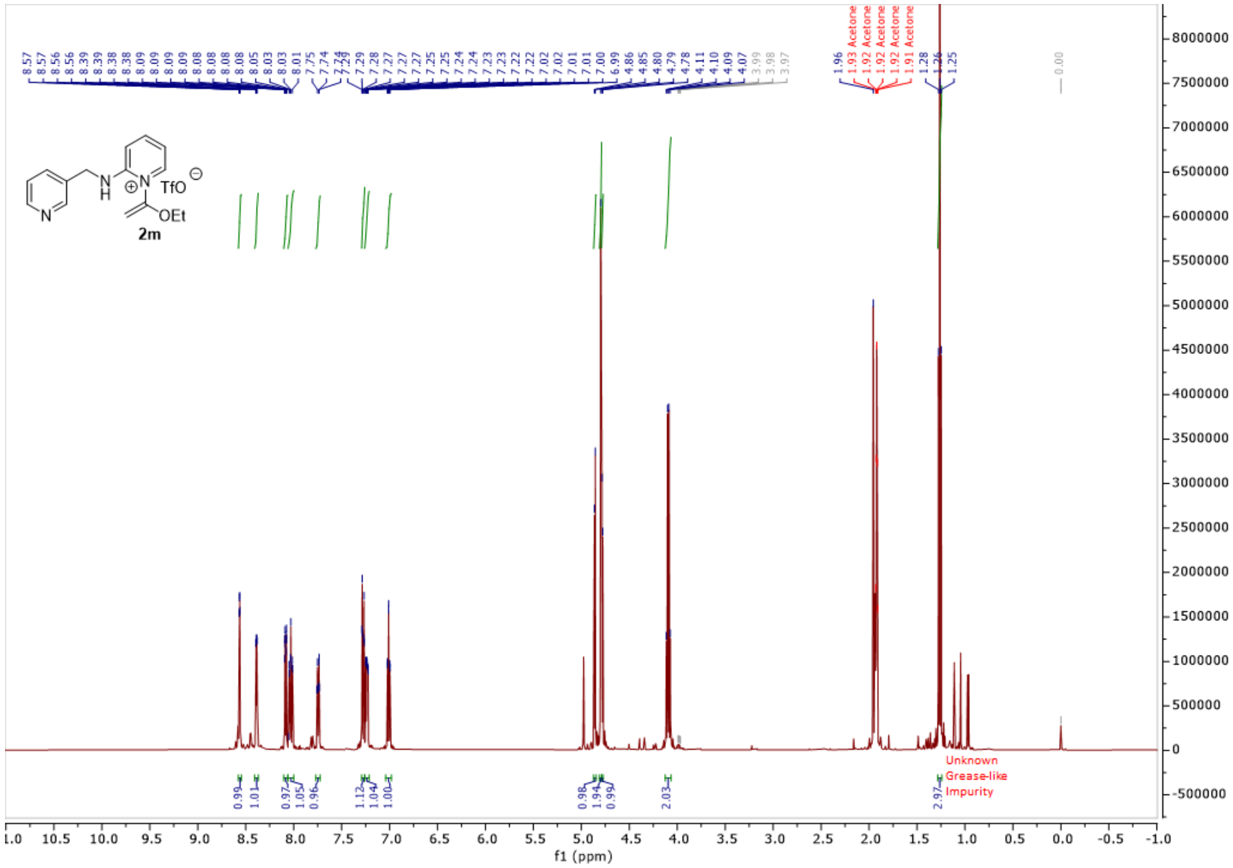

^{13}C{^{1}H} NMR Spectrum of **2m** (126 MHz, Acetone-d_6)

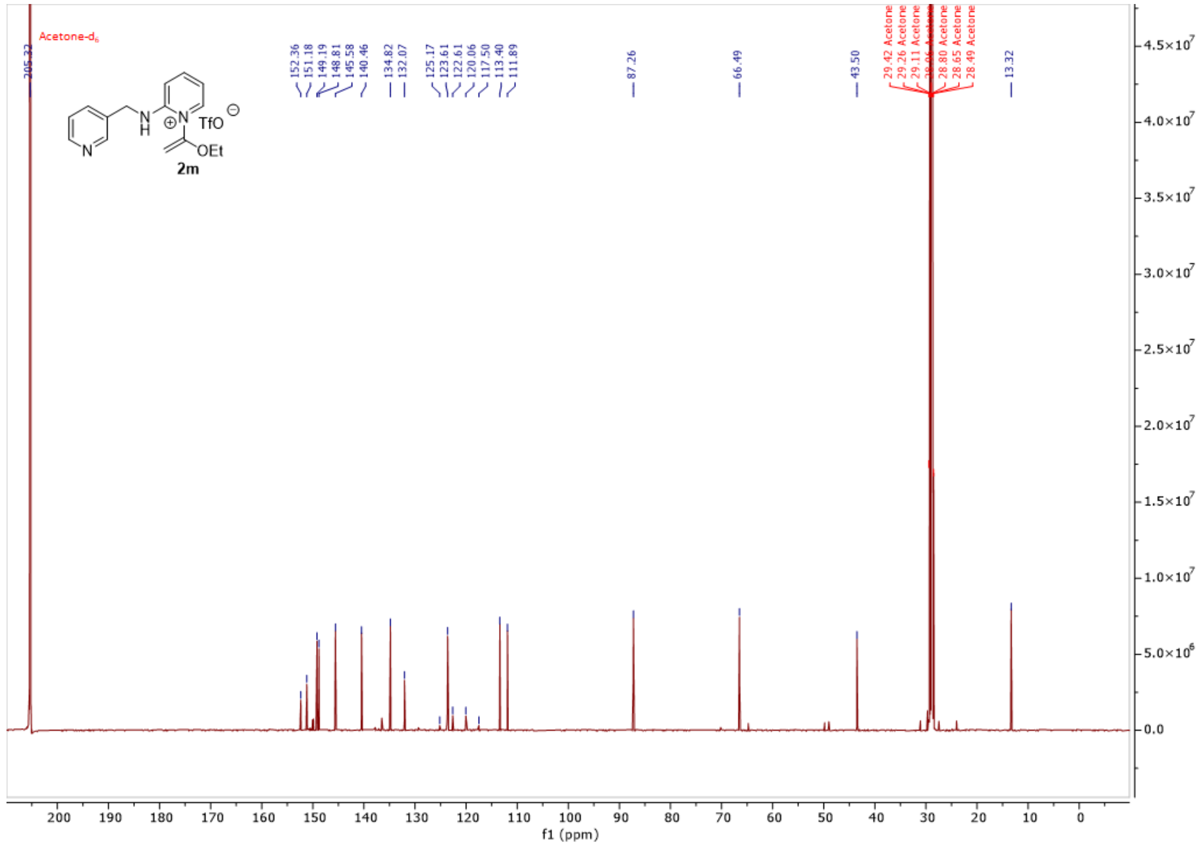

^{1}H NMR Spectrum of **2n** (500 MHz, CD_3OD)

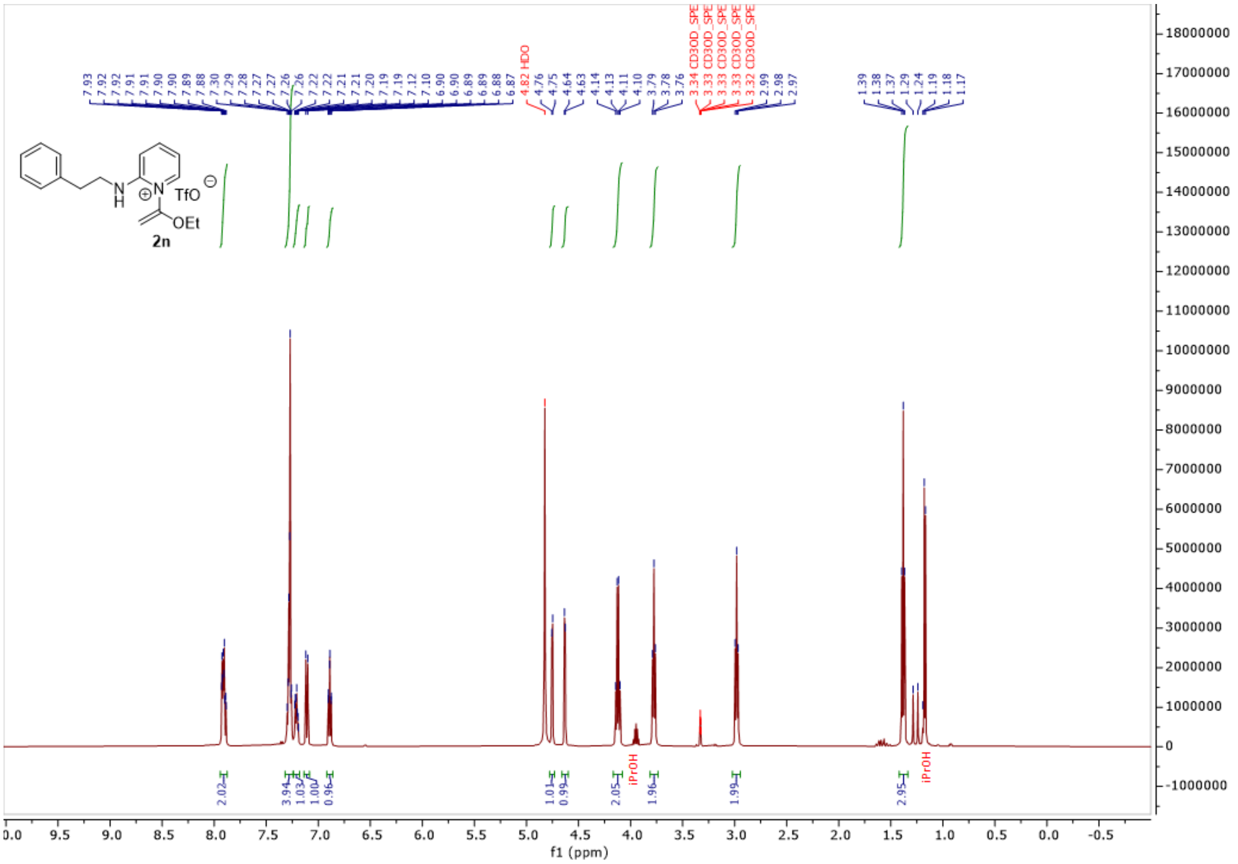

$^{13}C\{^{1}H\}$ NMR Spectrum of **2n** (126 MHz, CD_3OD)

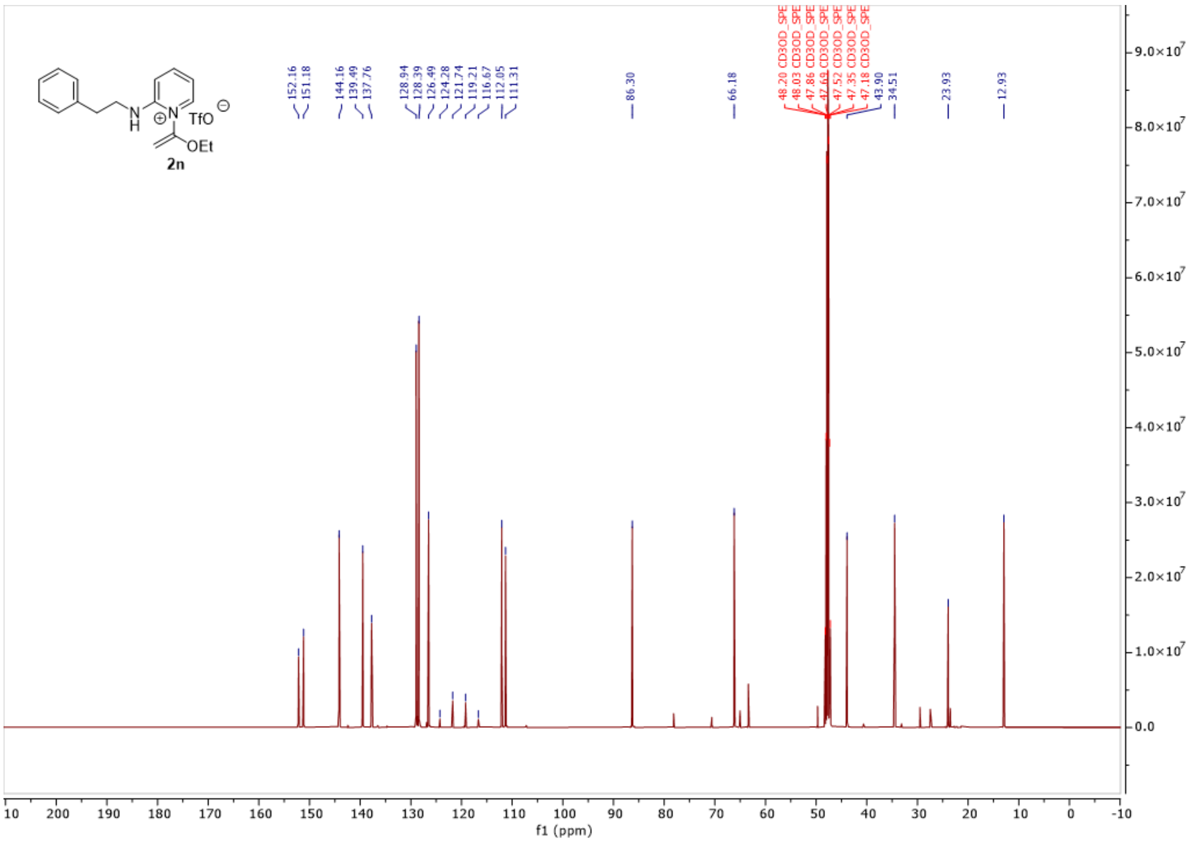

^{1}H NMR Spectrum of **2o** (500 MHz, Acetone-d_6)

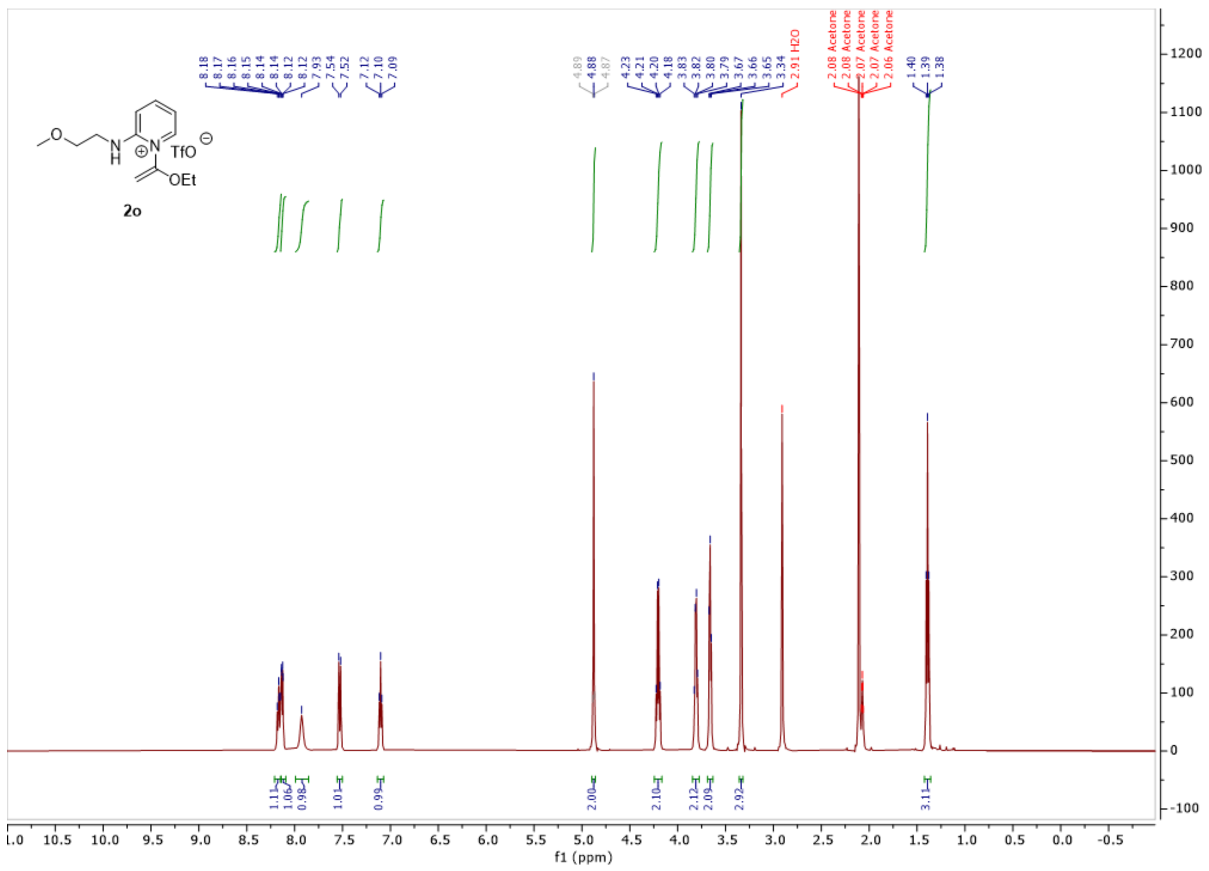

$^{13}C\{^{1}H\}$ NMR Spectrum of **2o** (126 MHz, Acetone-d_6)

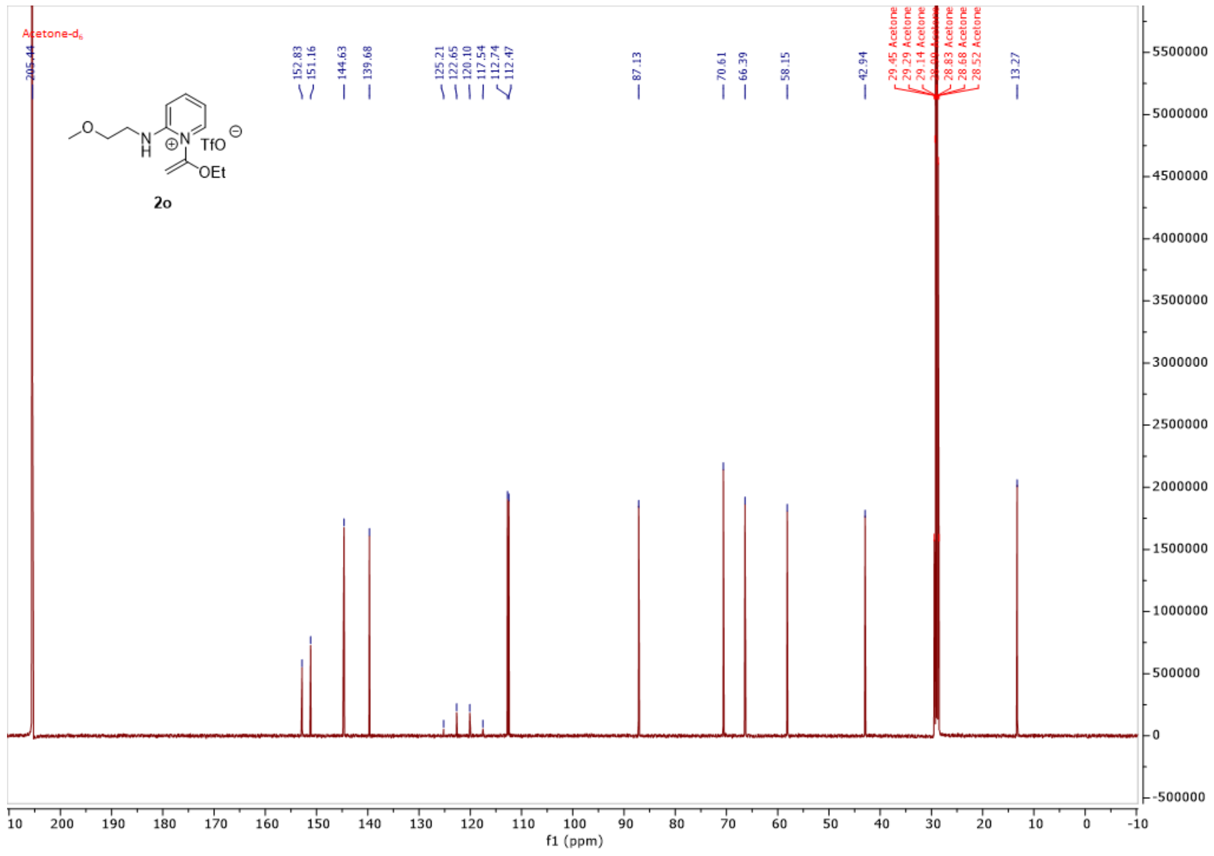

^{1}H NMR Spectrum of **2p** (500 MHz, Acetone-d_6)

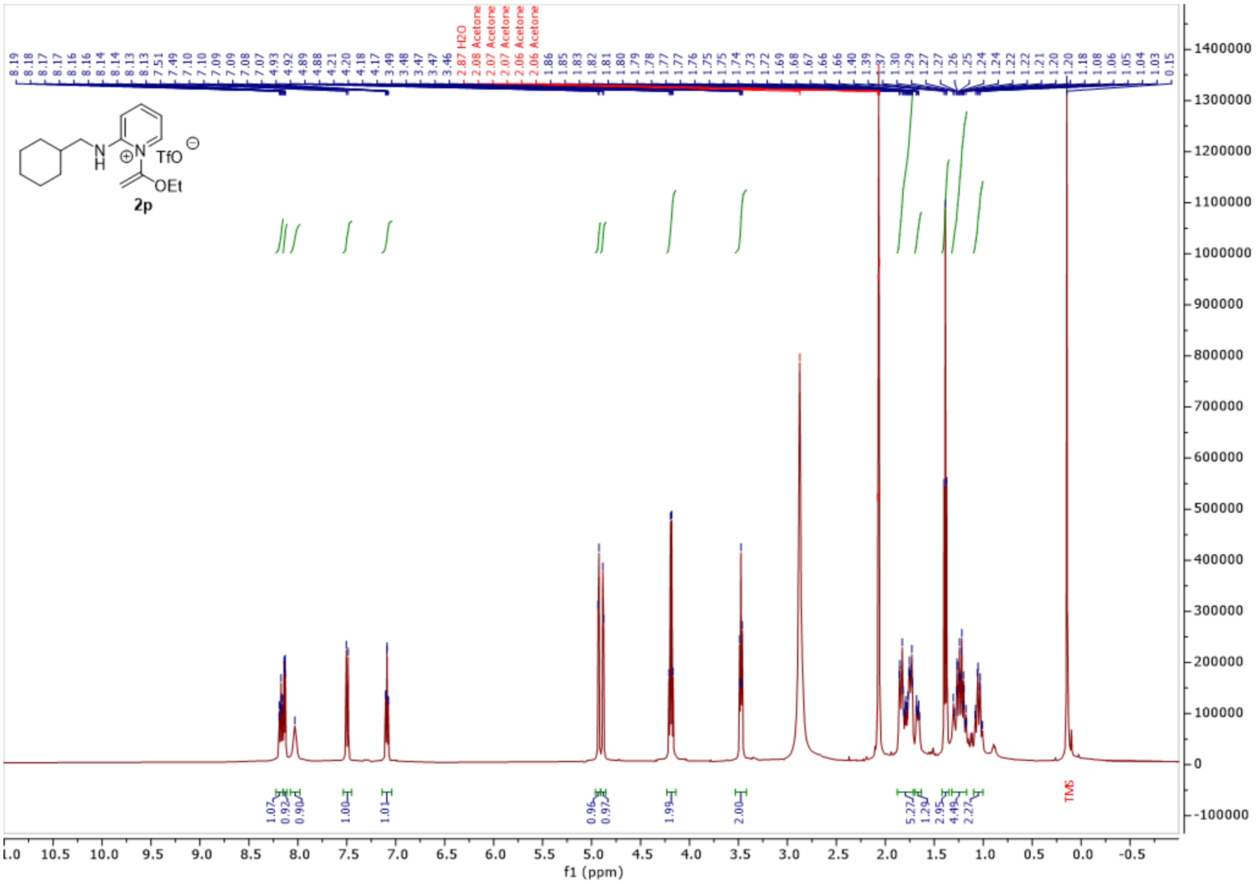

$^{13}C\{^{1}H\}$ NMR Spectrum of **2p** (126 MHz, Acetone-d_6)

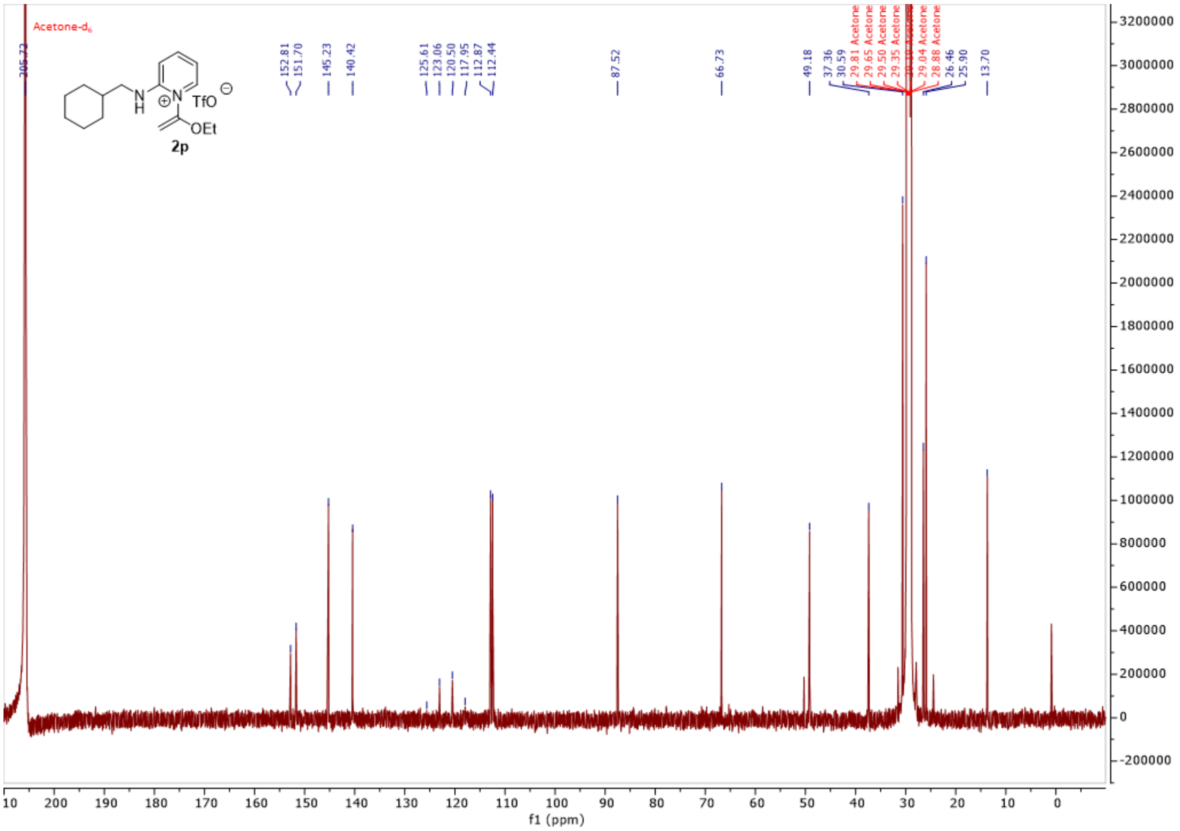

^{1}H NMR Spectrum of **2q** (500 MHz, Acetone-d_6)

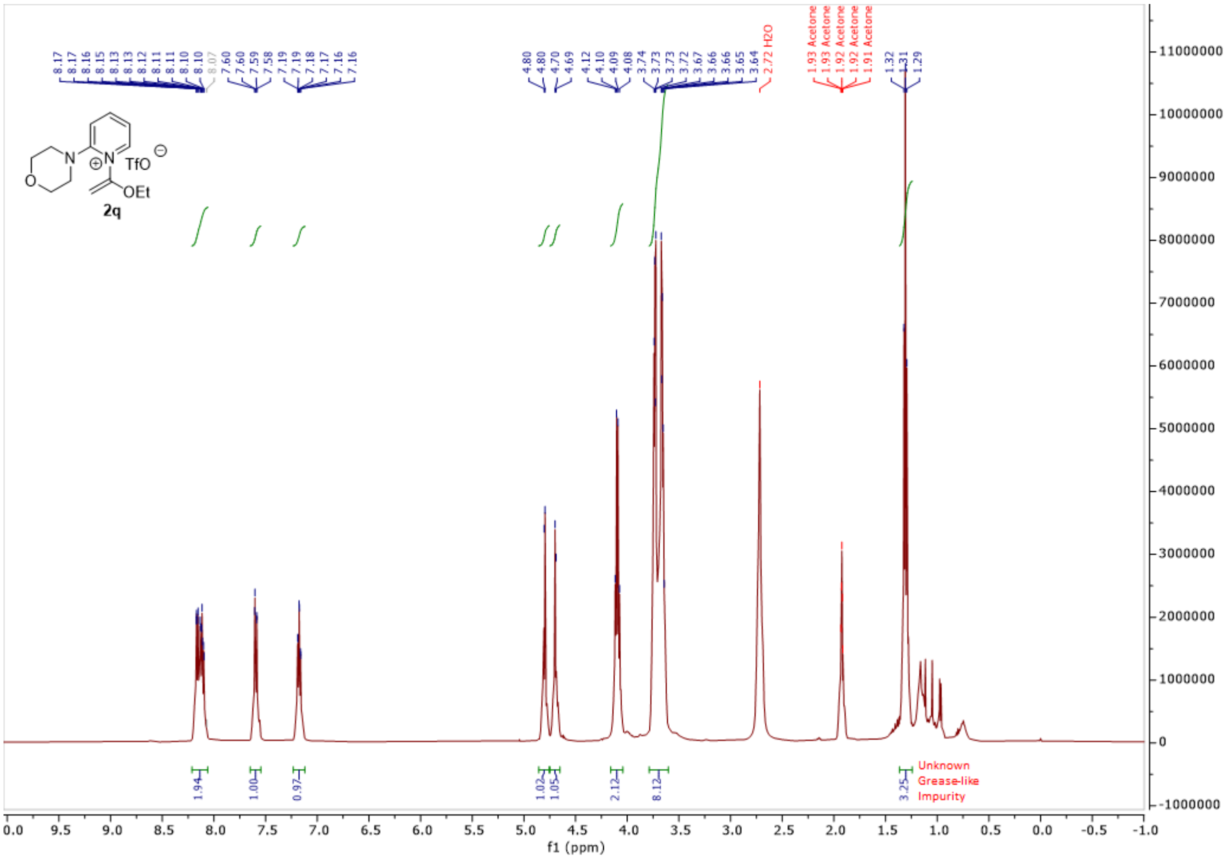

^{13}C{^{1}H} NMR Spectrum of **2q** (126 MHz, Acetone-d_6)

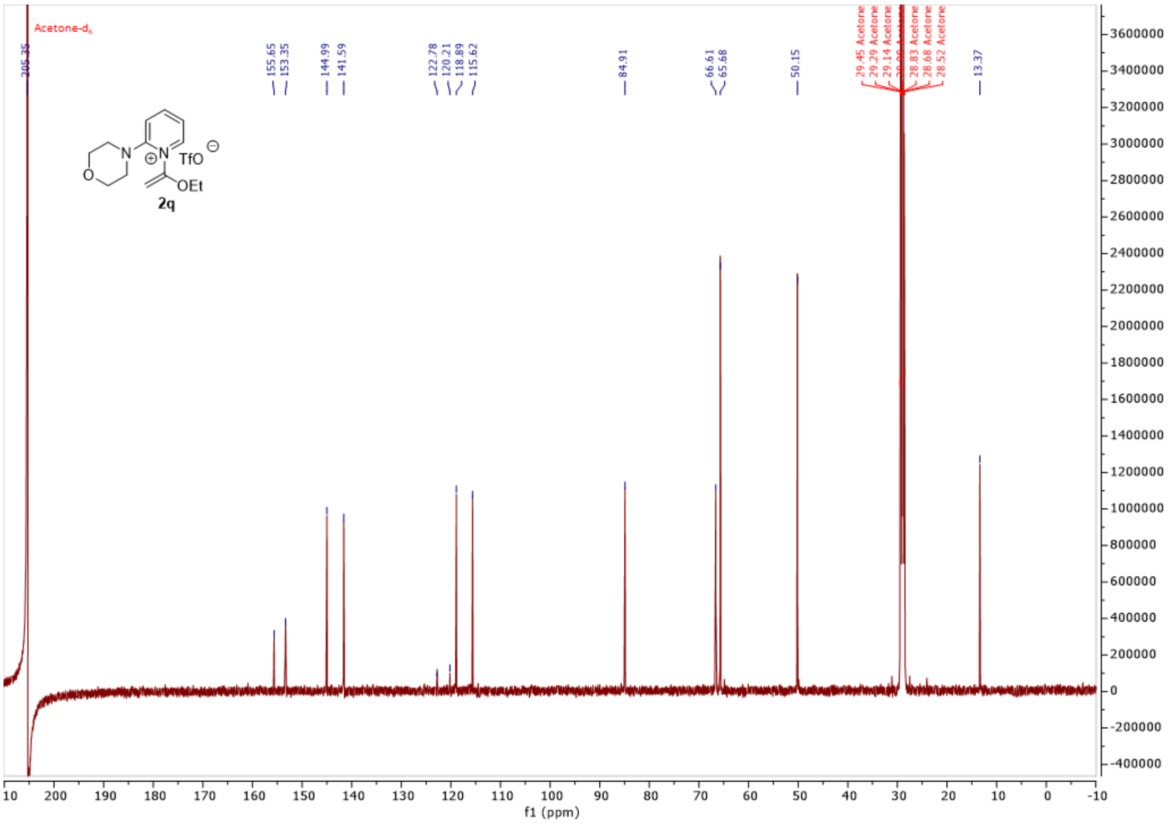

^{1}H NMR Spectrum of **2r** (500 MHz, Acetone-d_6)

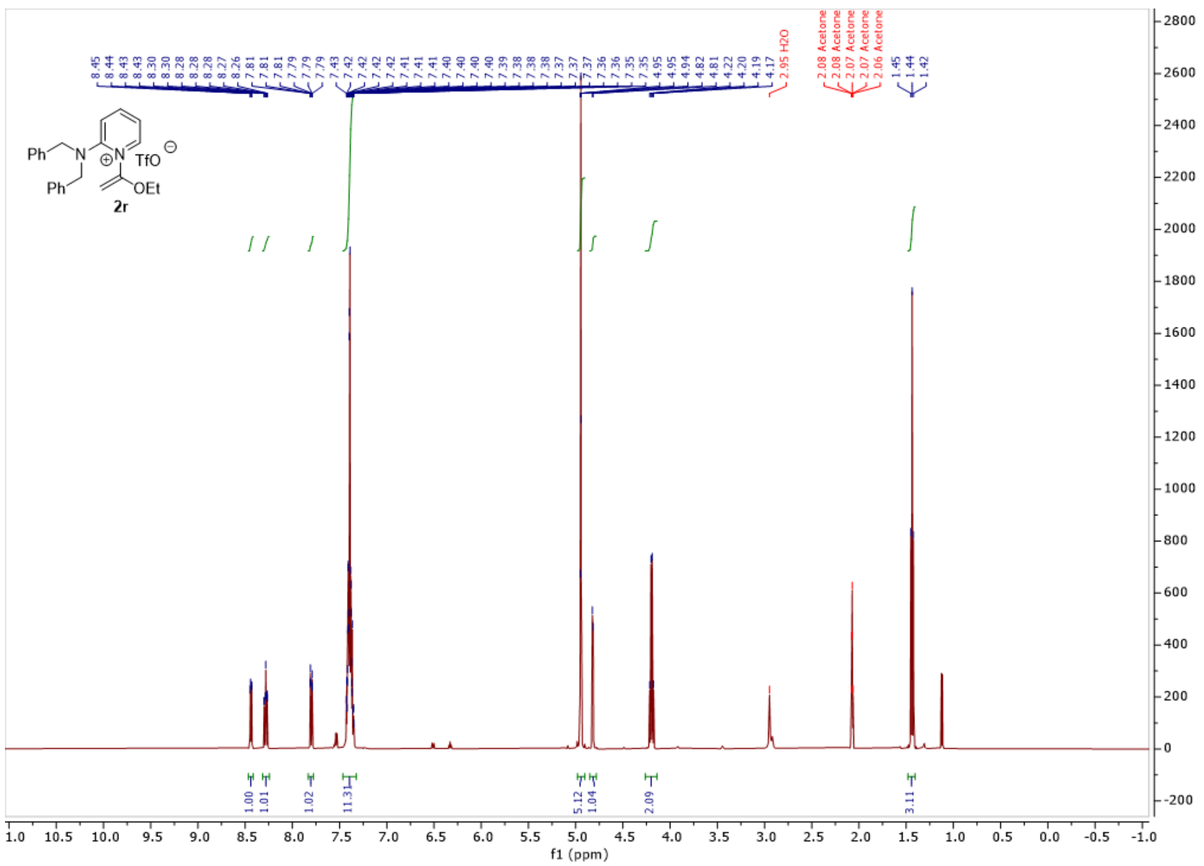

$^{13}C\{^{1}H\}$ NMR Spectrum of **2r** (126 MHz, Acetone-d_6)

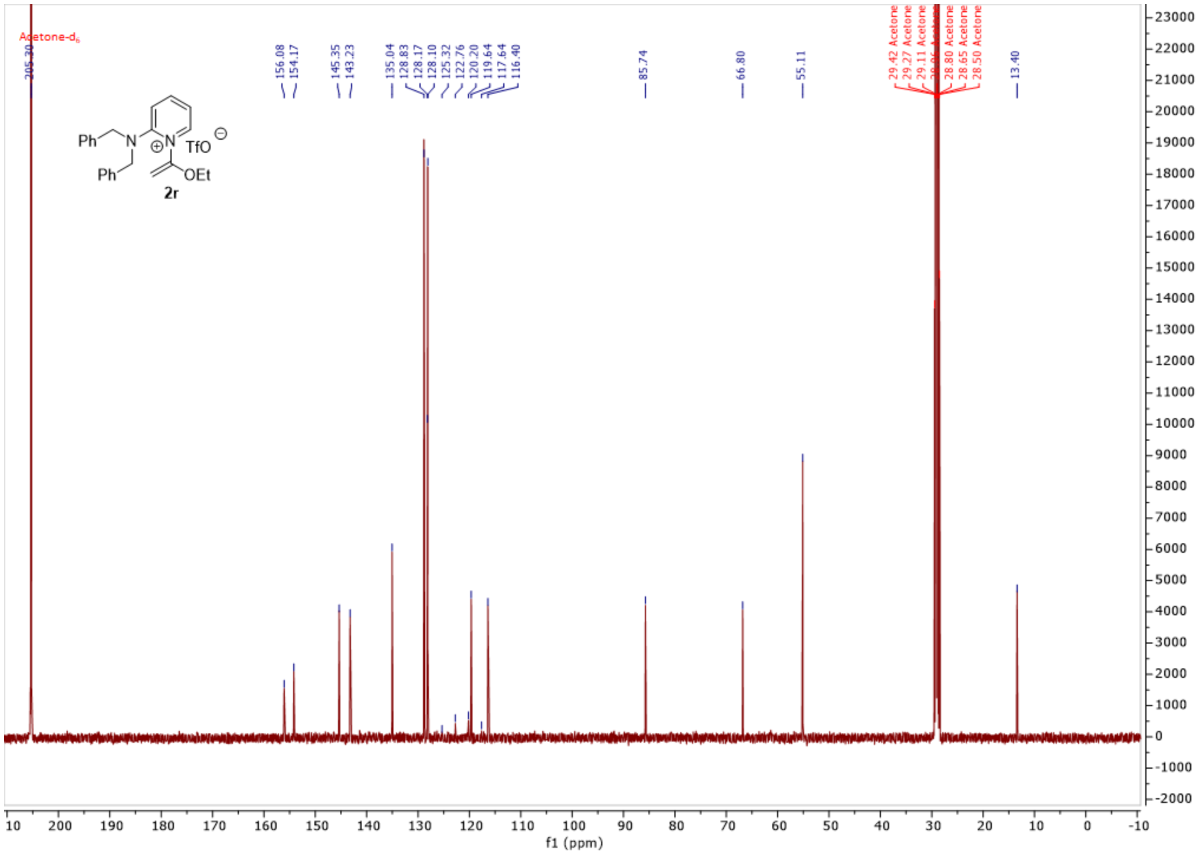

^{1}H NMR Spectrum of **2s** (500 MHz, Acetone-d_6)

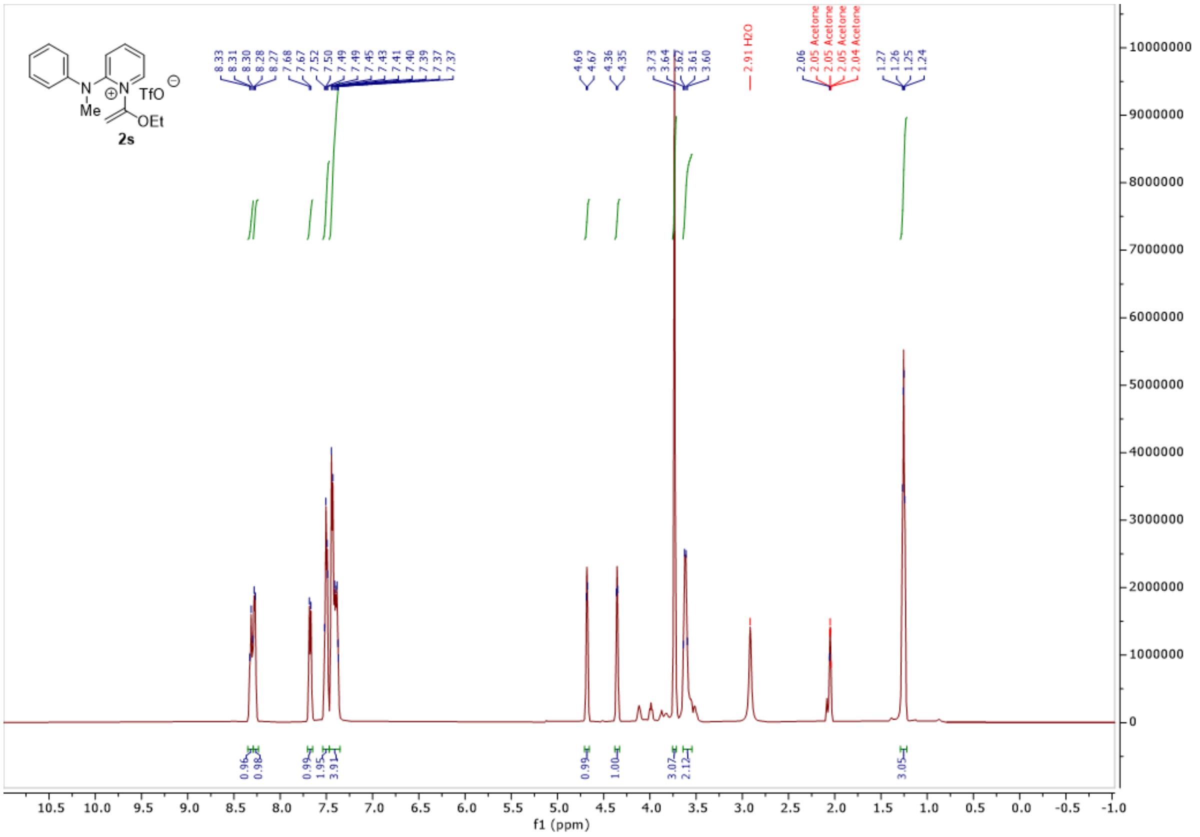

$^{13}C\{^{1}H\}$ NMR Spectrum of **2s** (126 MHz, Acetone-d_6)

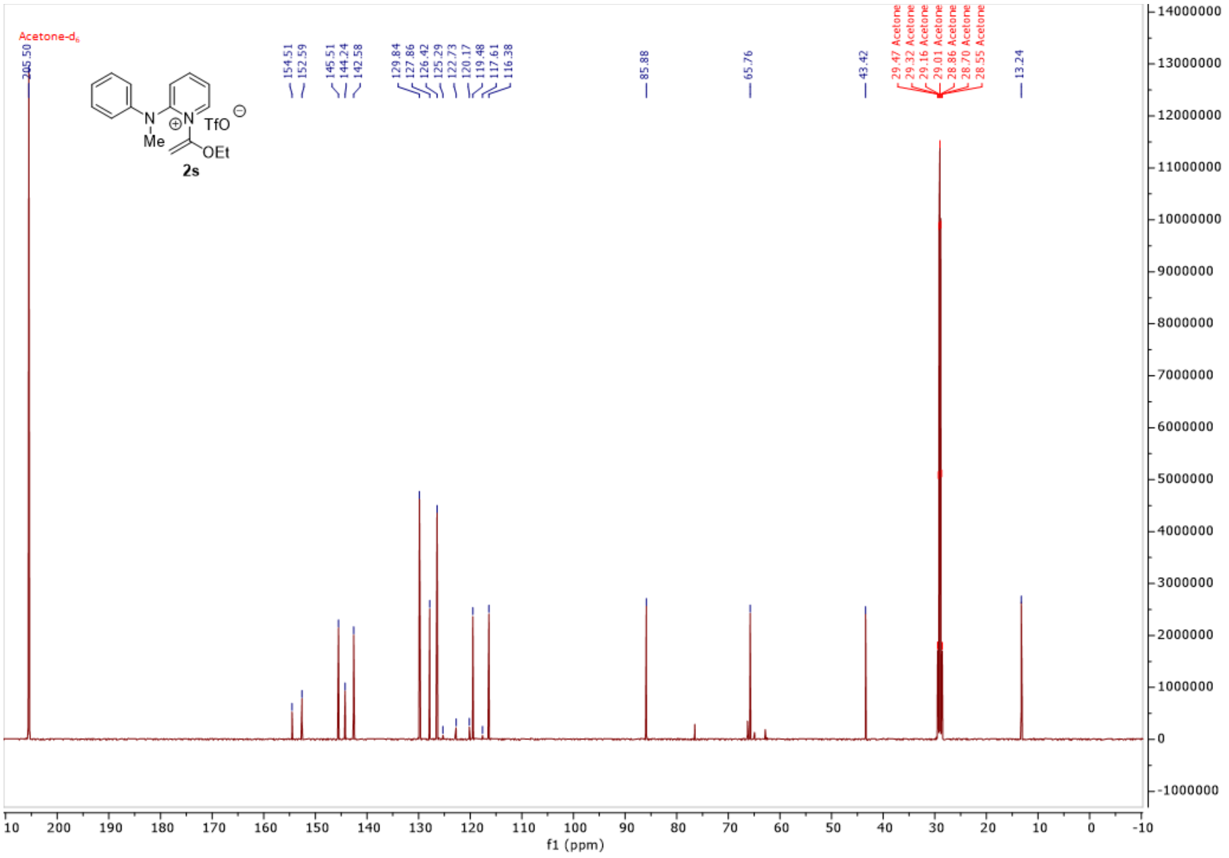

^{1}H NMR Spectrum of **2t** (500 MHz, CD_3OD)

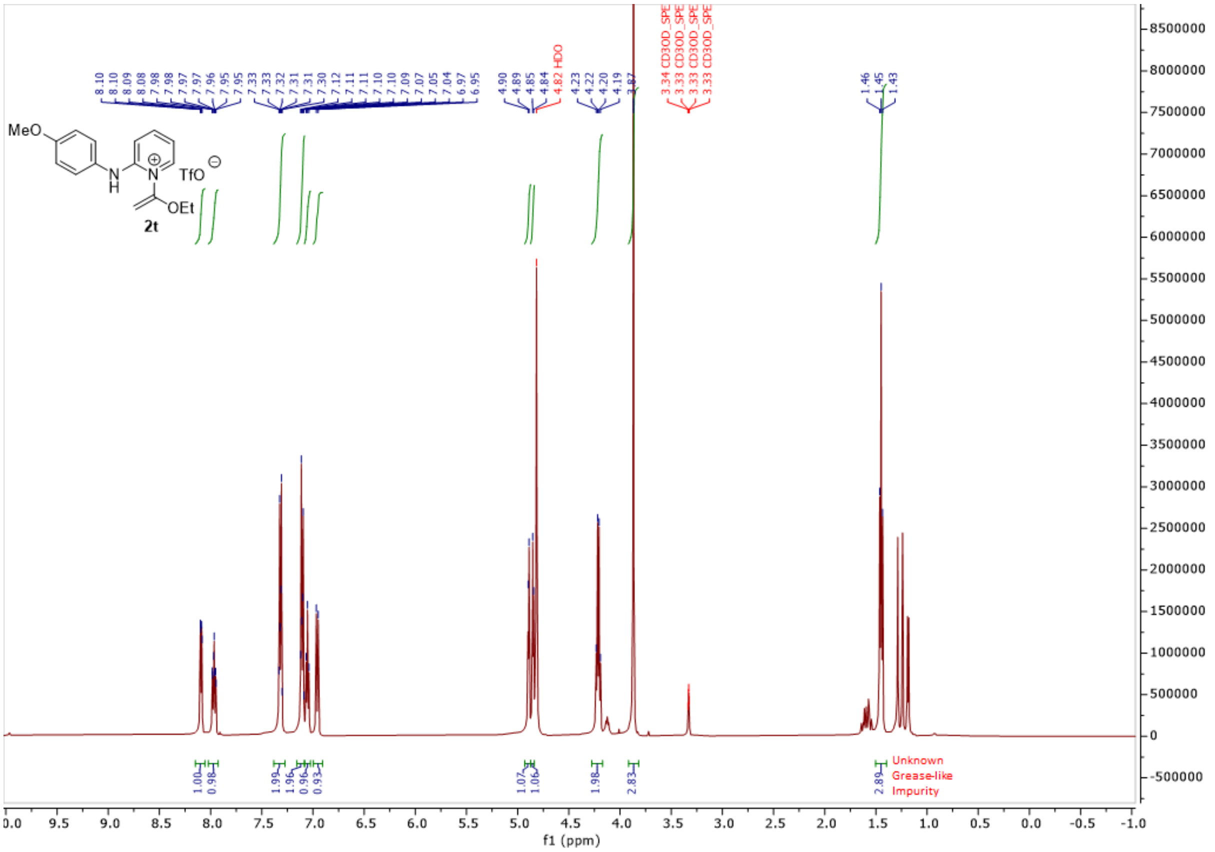

$^{13}C\{^{1}H\}$ NMR Spectrum of **2t** (126 MHz, CD_3OD)

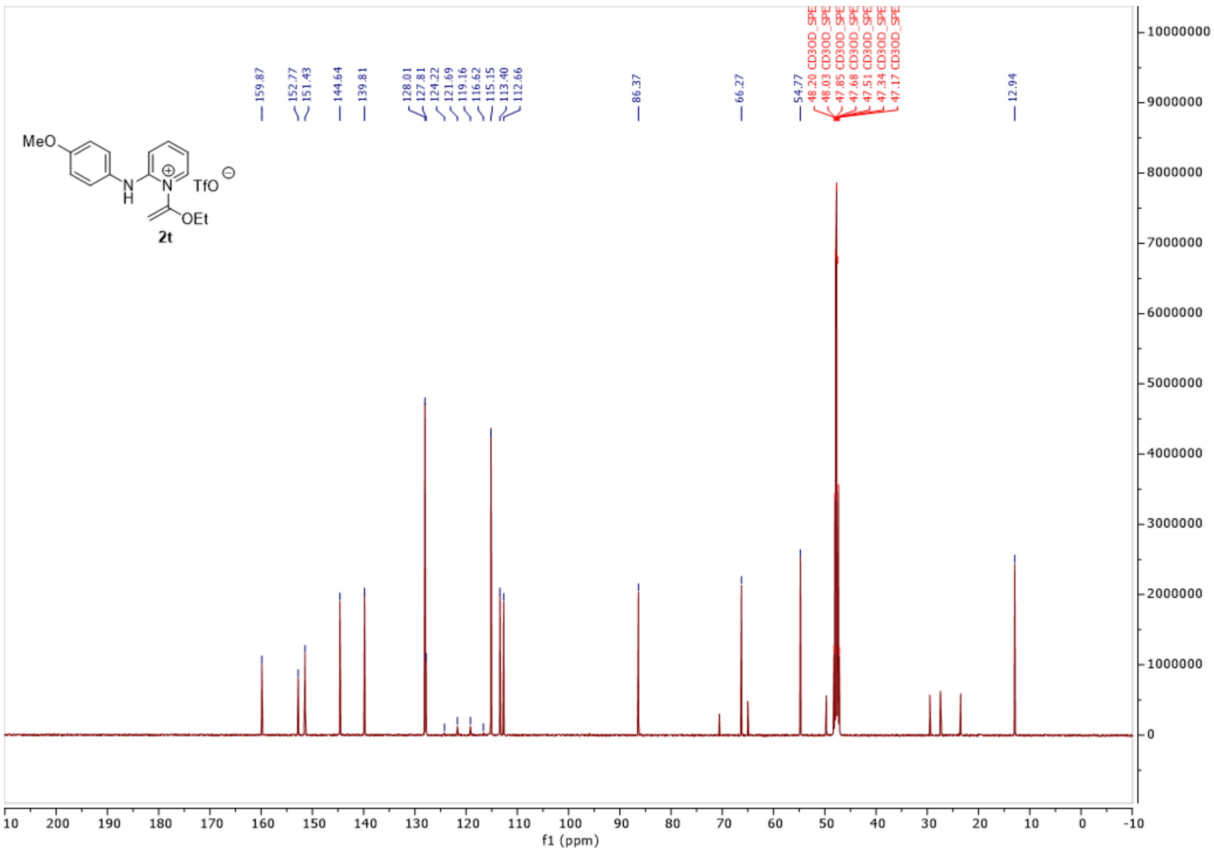

^{1}H NMR Spectrum of **2u** (500 MHz, CD_3OD)

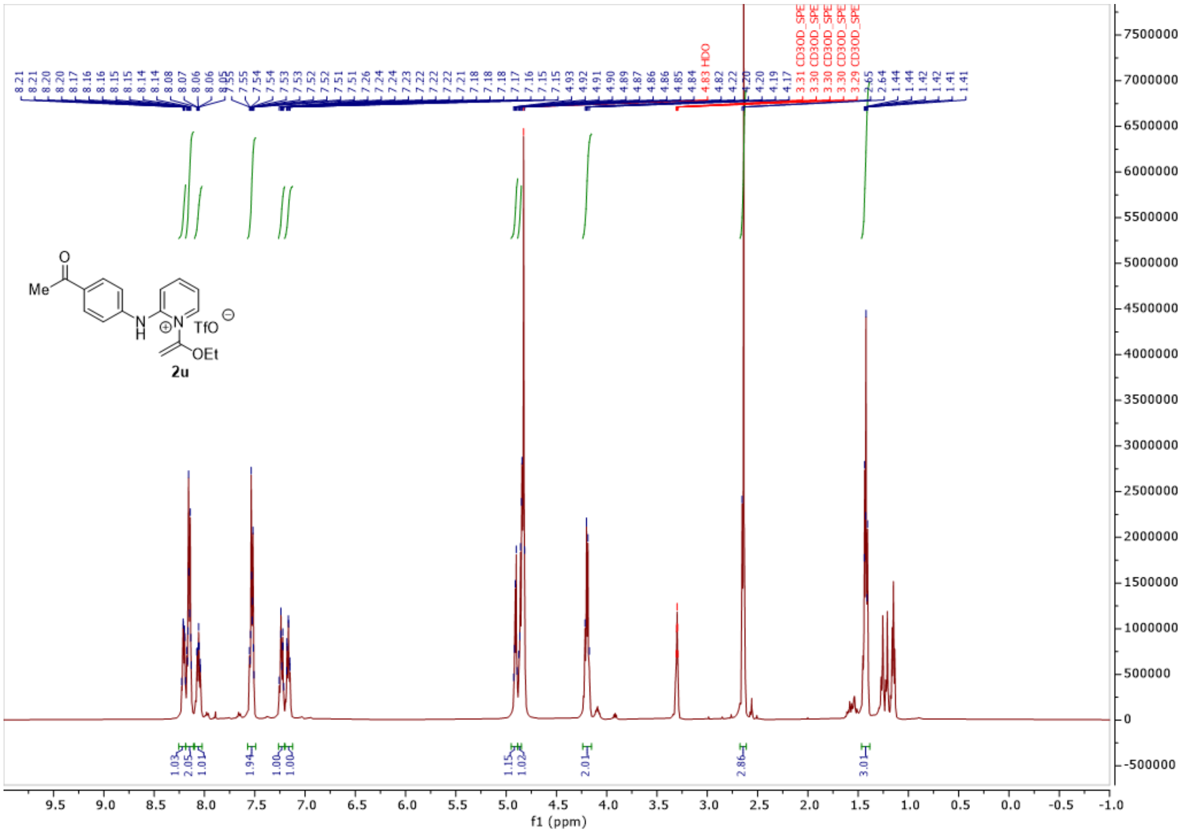

^{13}C{^{1}H} NMR Spectrum of **2u** (126 MHz, CD_3OD)

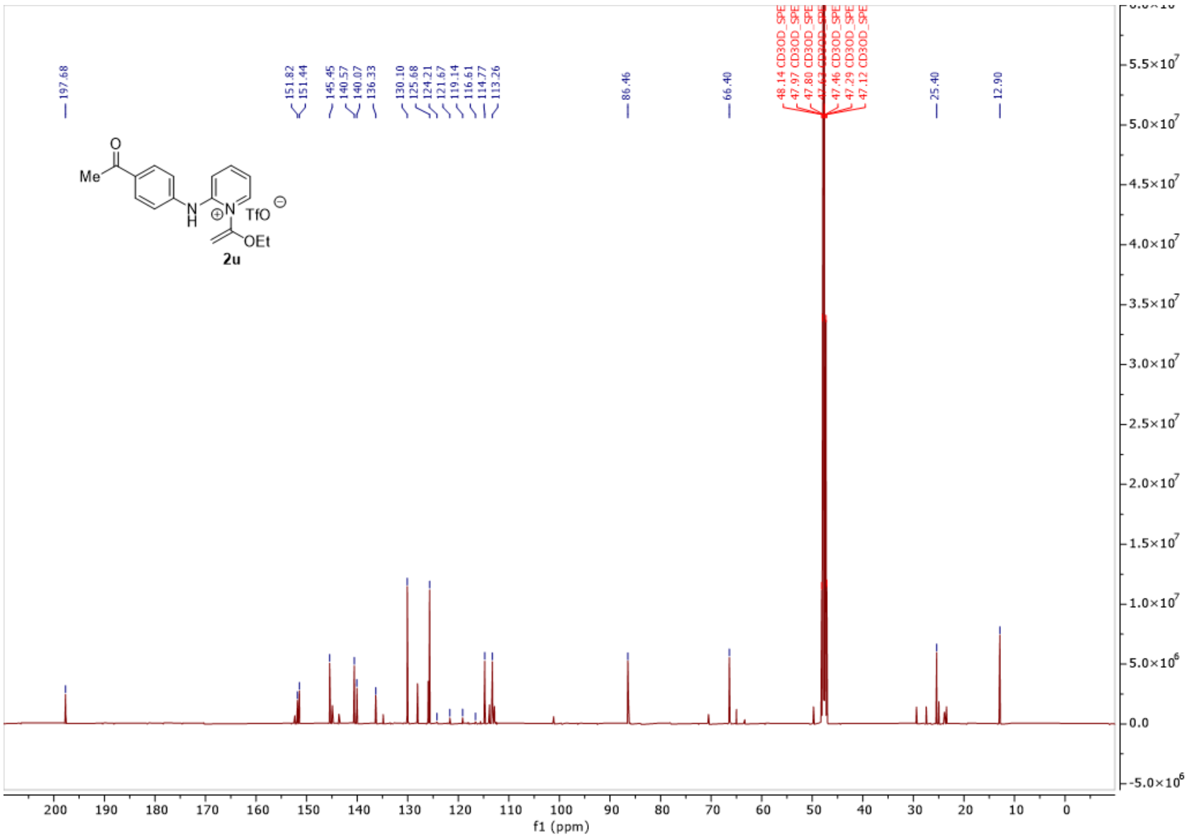

^{1}H NMR Spectrum of **2v** (500 MHz, Acetone-d_6)

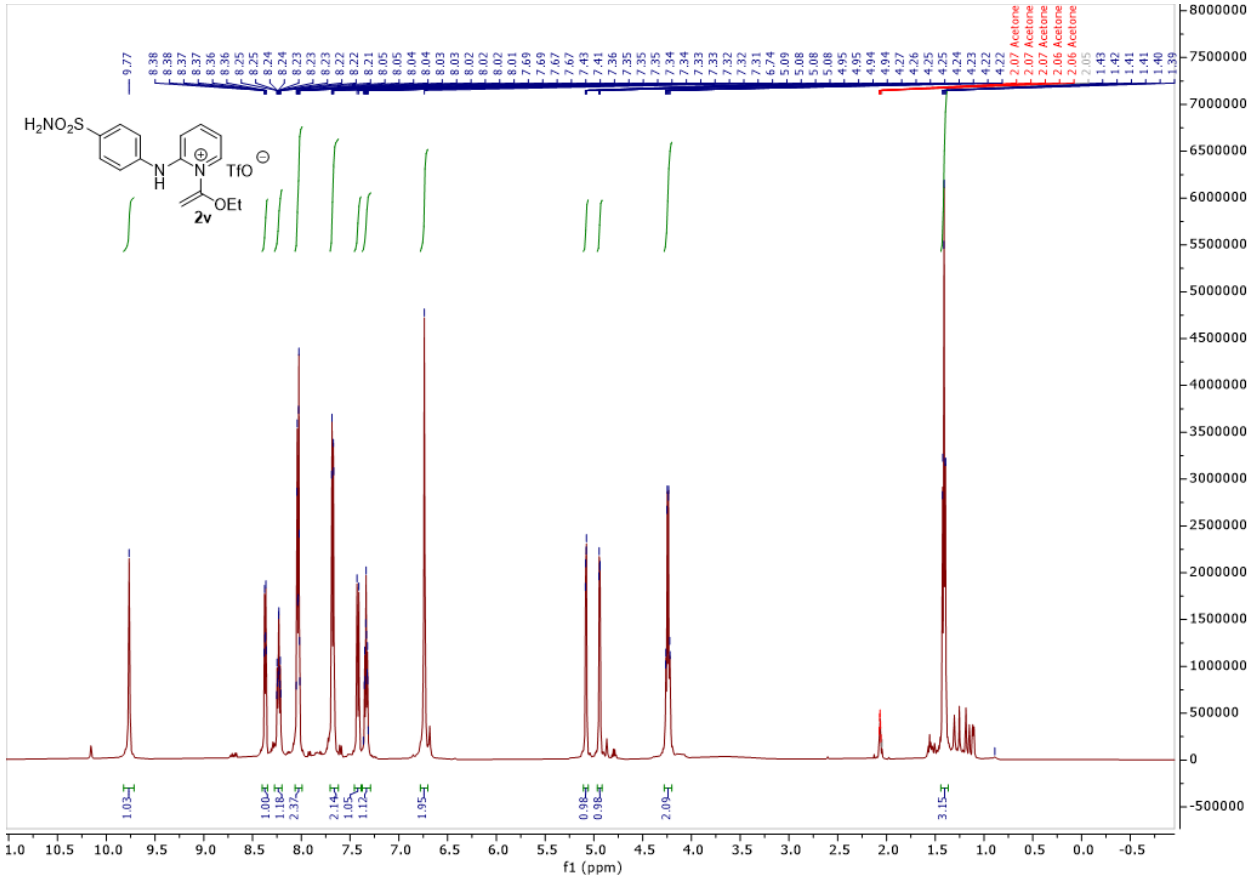

$^{13}C\{^{1}H\}$ NMR Spectrum of **2v** (126 MHz, Acetone-d_6)

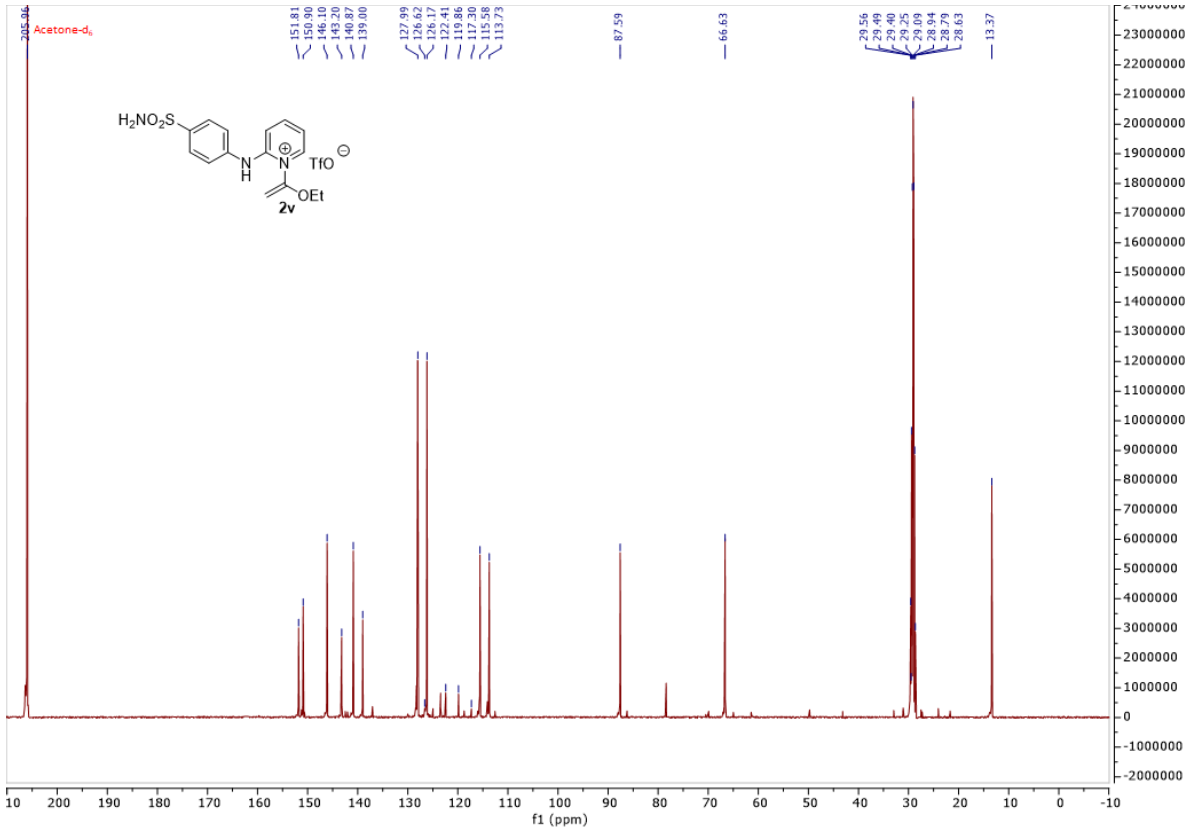

^{1}H NMR Spectrum of **2w** (500 MHz, Acetone-d_6)

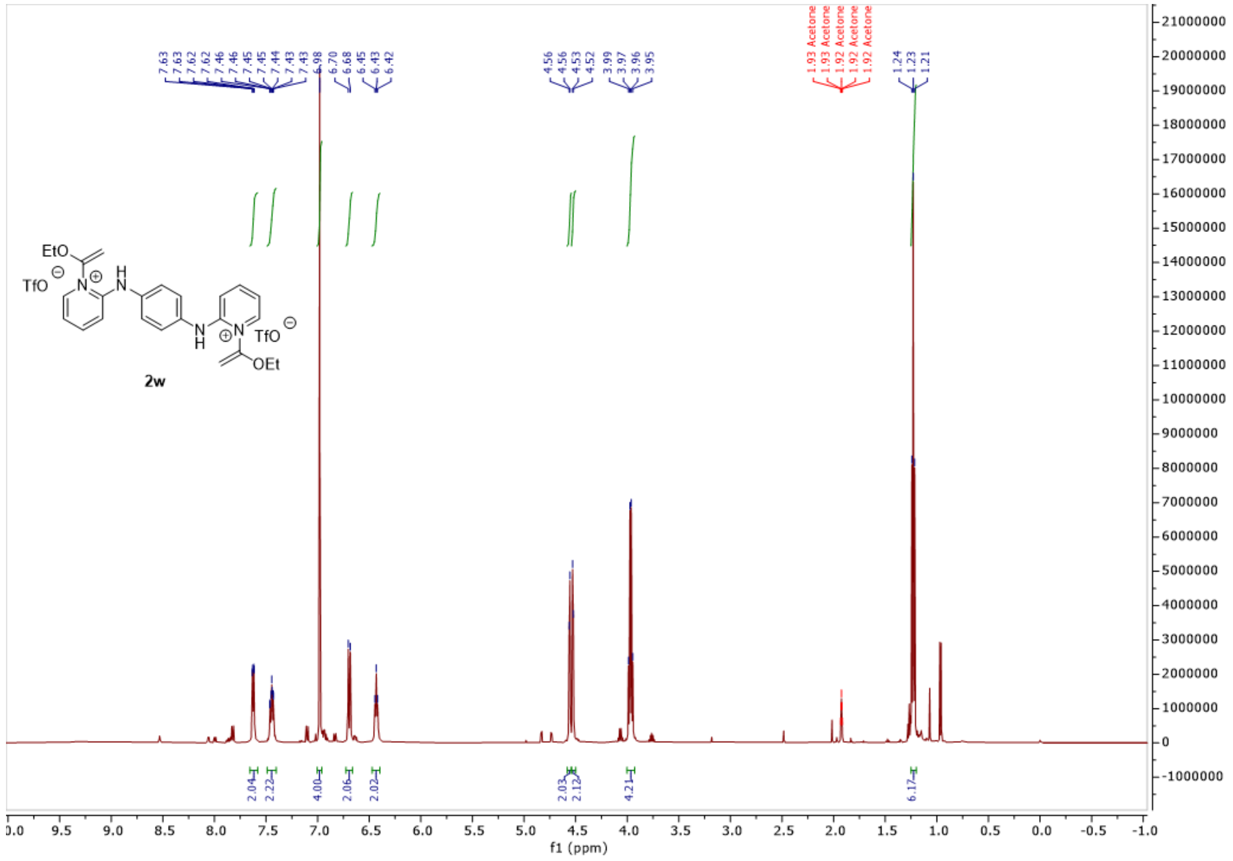

$^{13}C\{^{1}H\}$ NMR Spectrum of **2w** (126 MHz, Acetone-d_6)

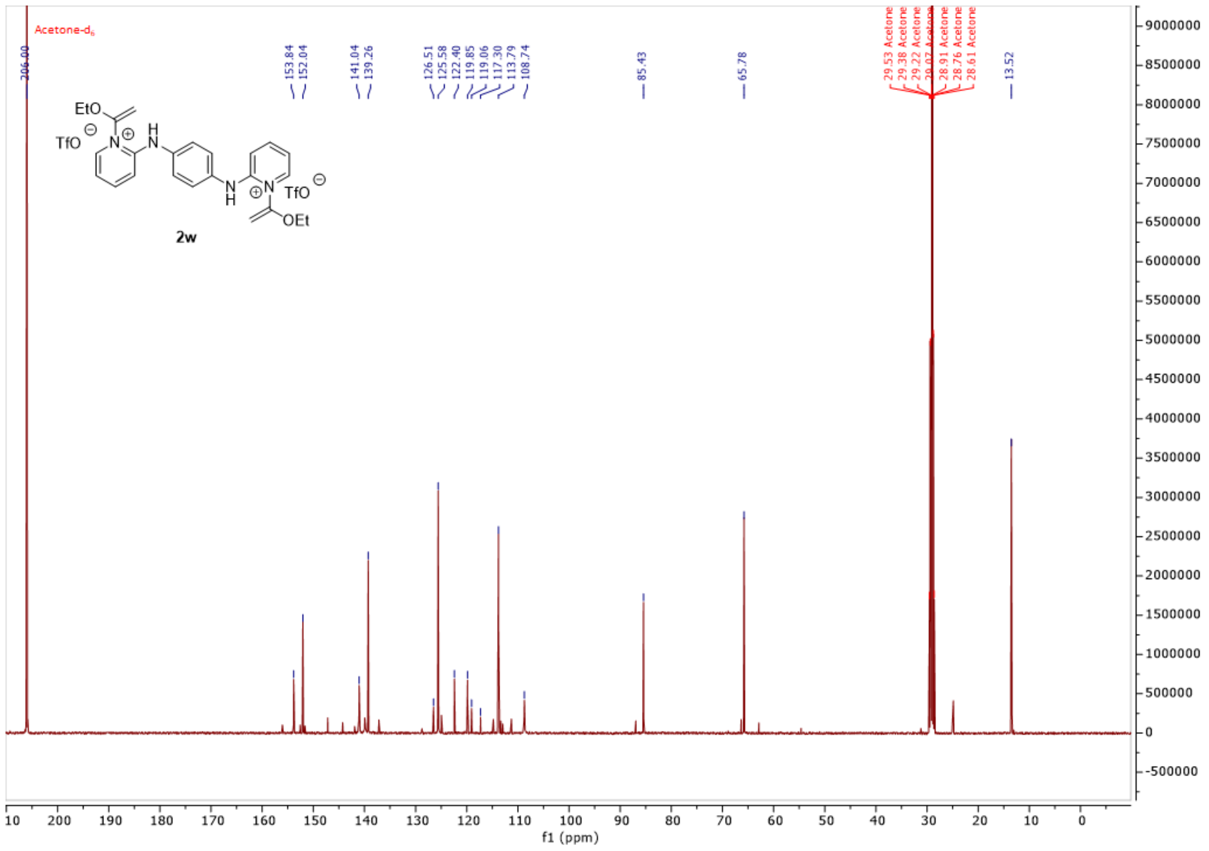

^{1}H NMR Spectrum of **2x** (500 MHz, Acetone-d_6)

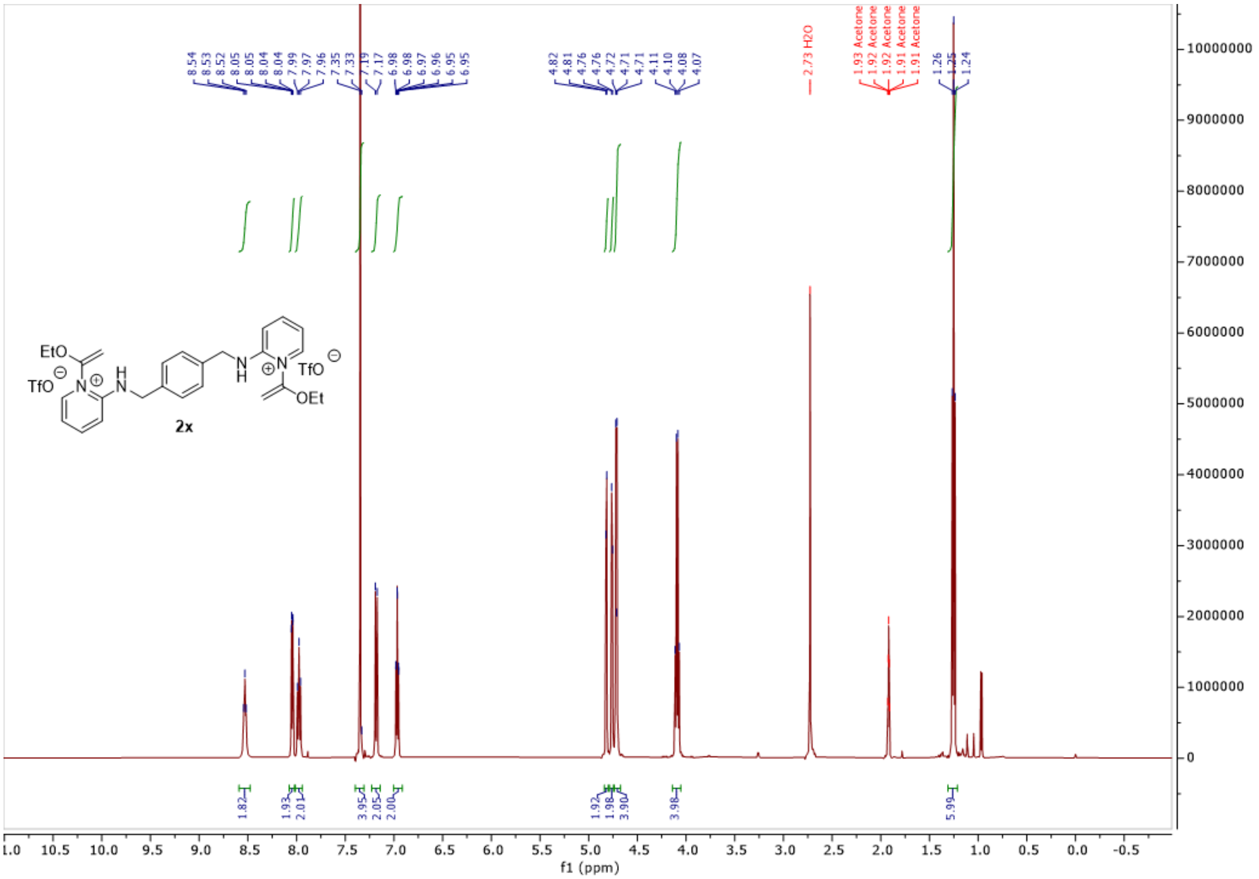

$^{13}C\{^{1}H\}$ NMR Spectrum of **2x** (126 MHz, Acetone-d_6)

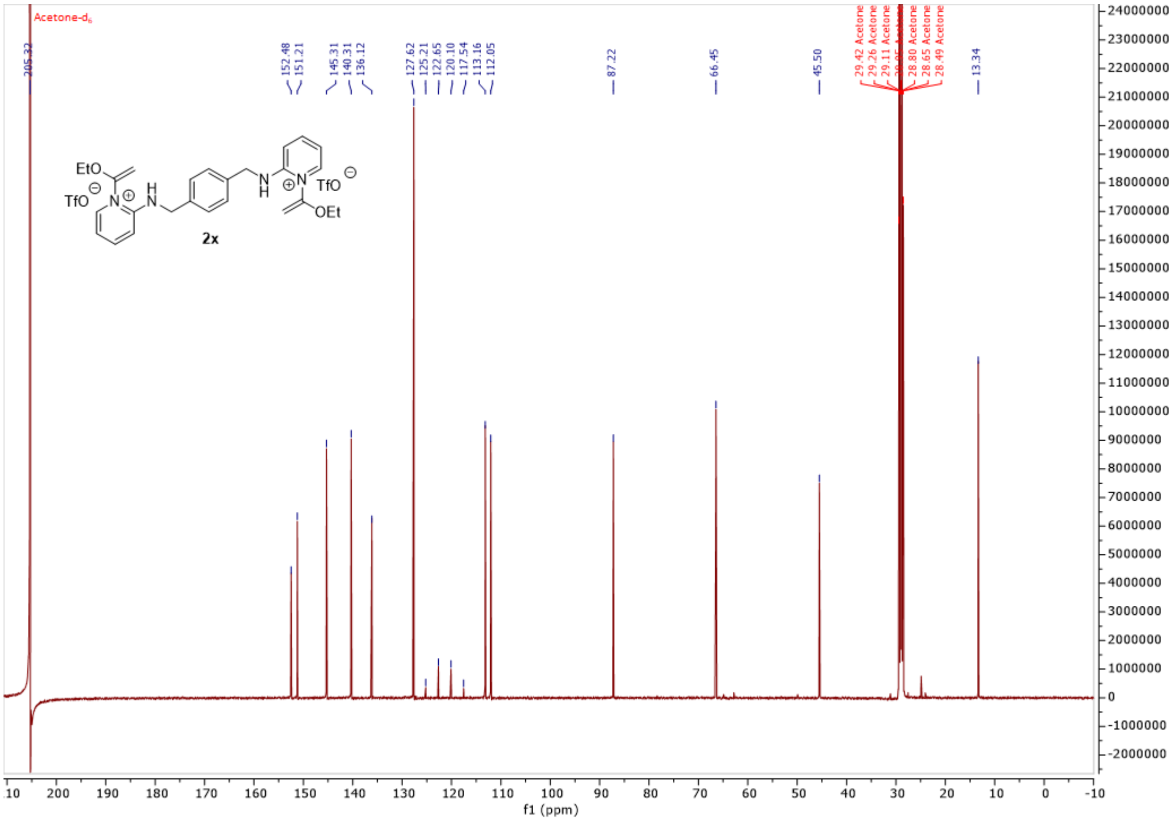

^{1}H NMR Spectrum of **2y** (500 MHz, CD_3OD)

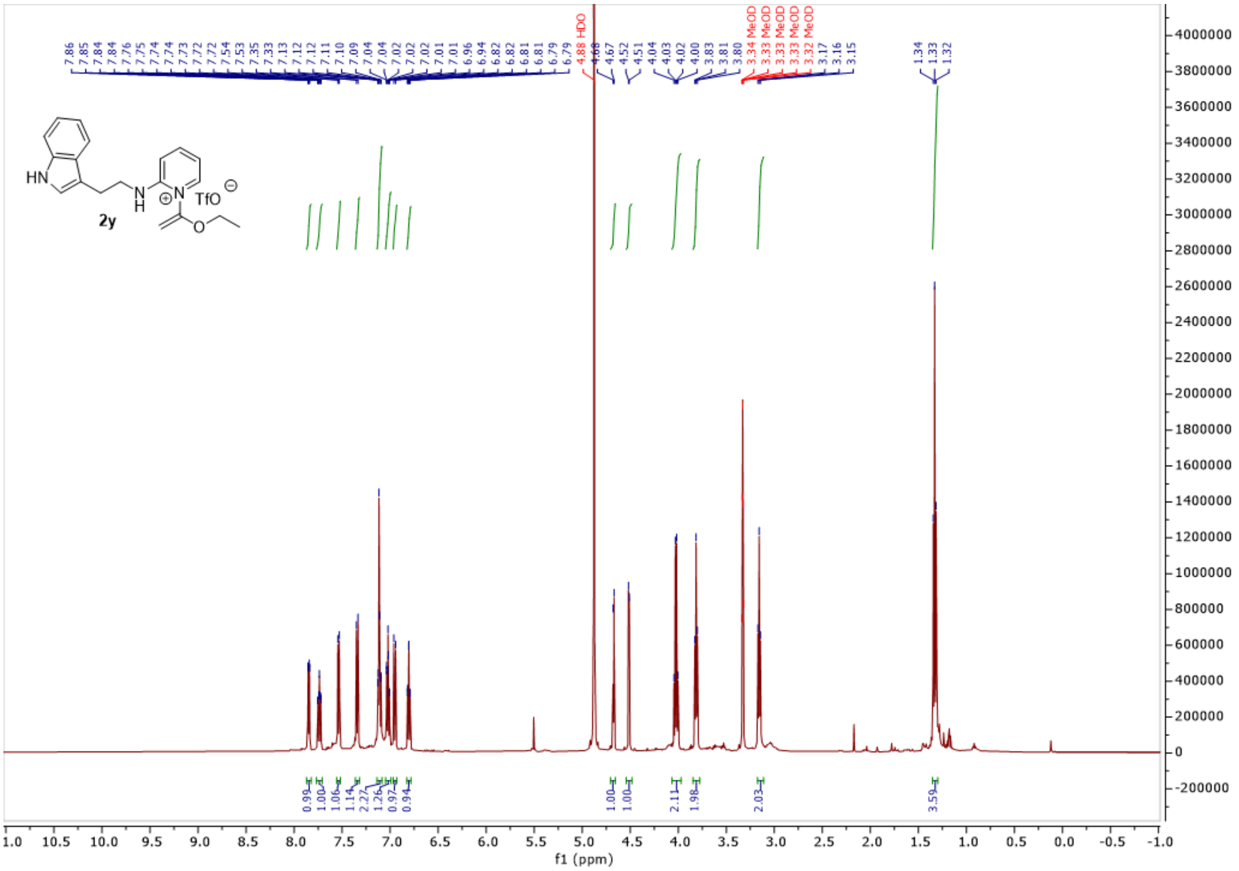

$^{13}C\{^{1}H\}$ NMR Spectrum of **2y** (126 MHz, CD_3OD)

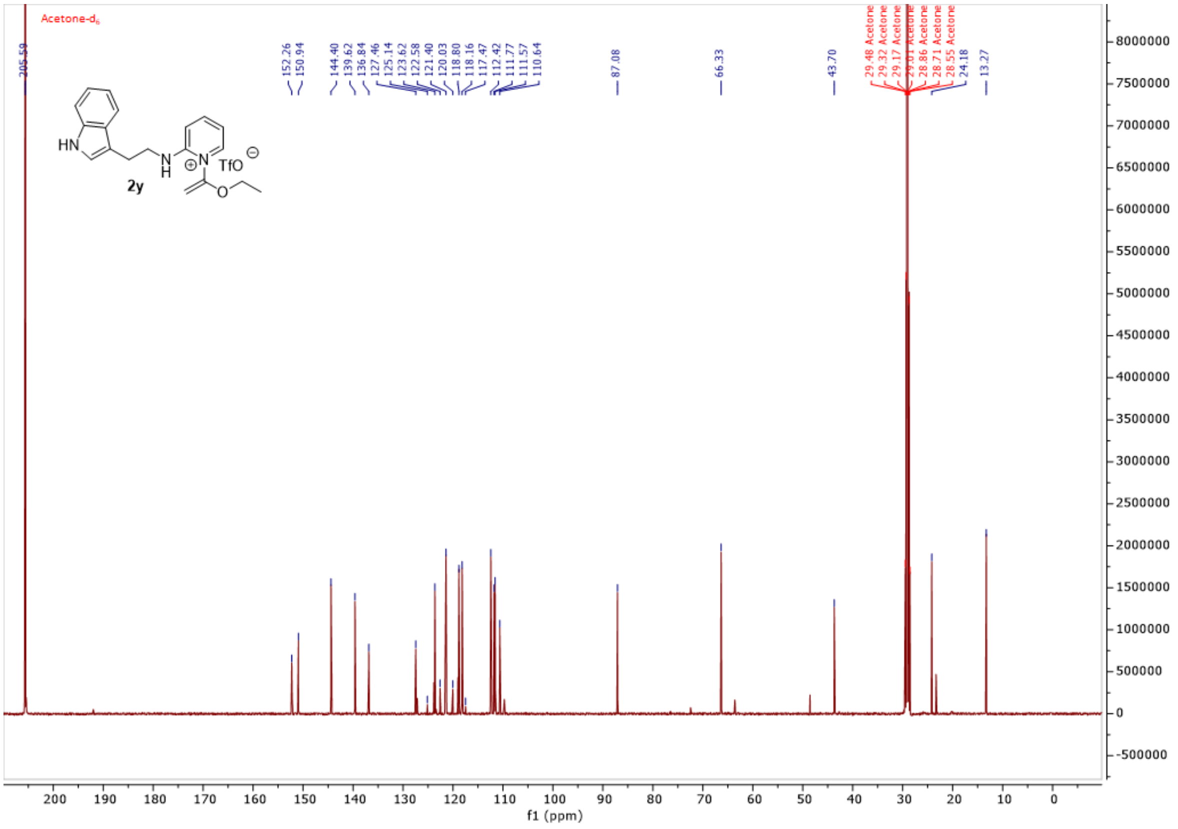

^{1}H NMR Spectrum of **2z** (500 MHz, CD_3CN)

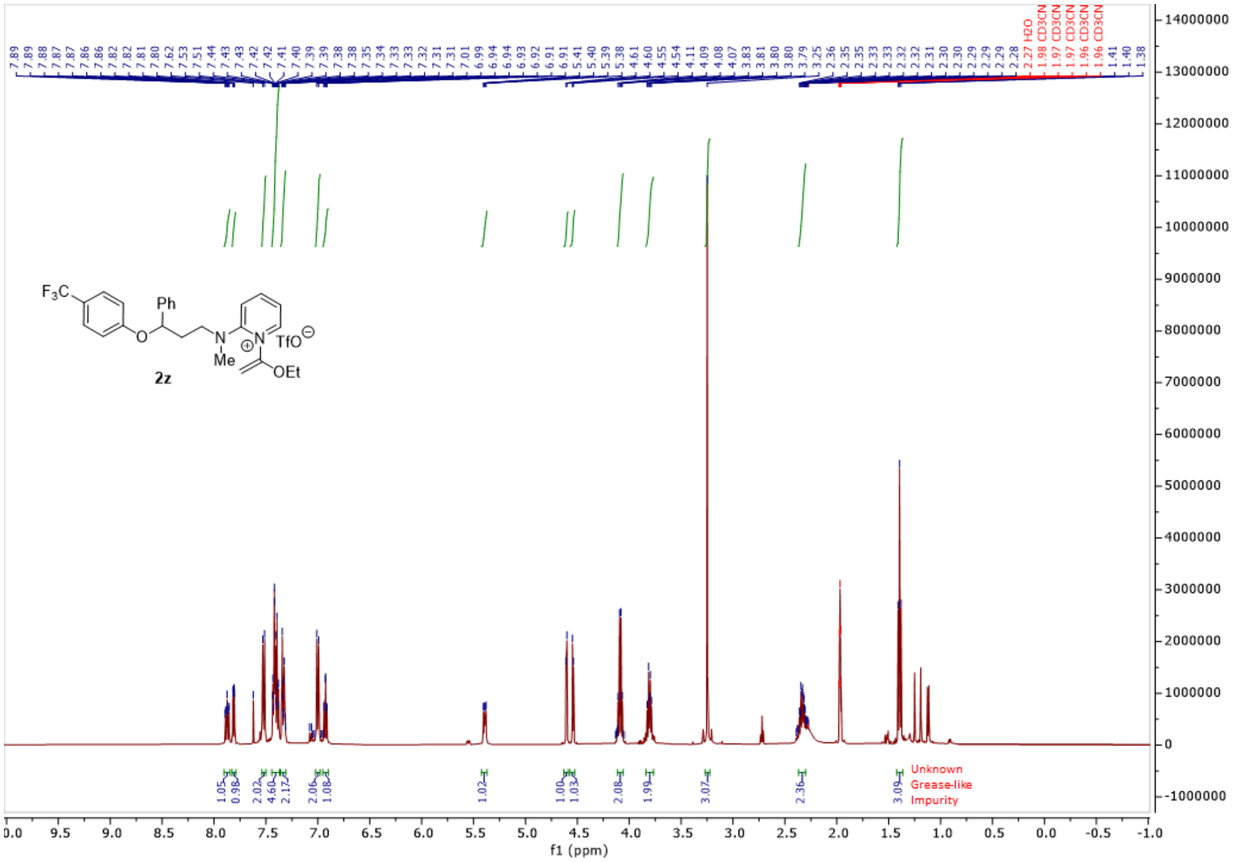

$^{13}C\{^{1}H\}$ NMR Spectrum of **2z** (126 MHz, CD_3CN)

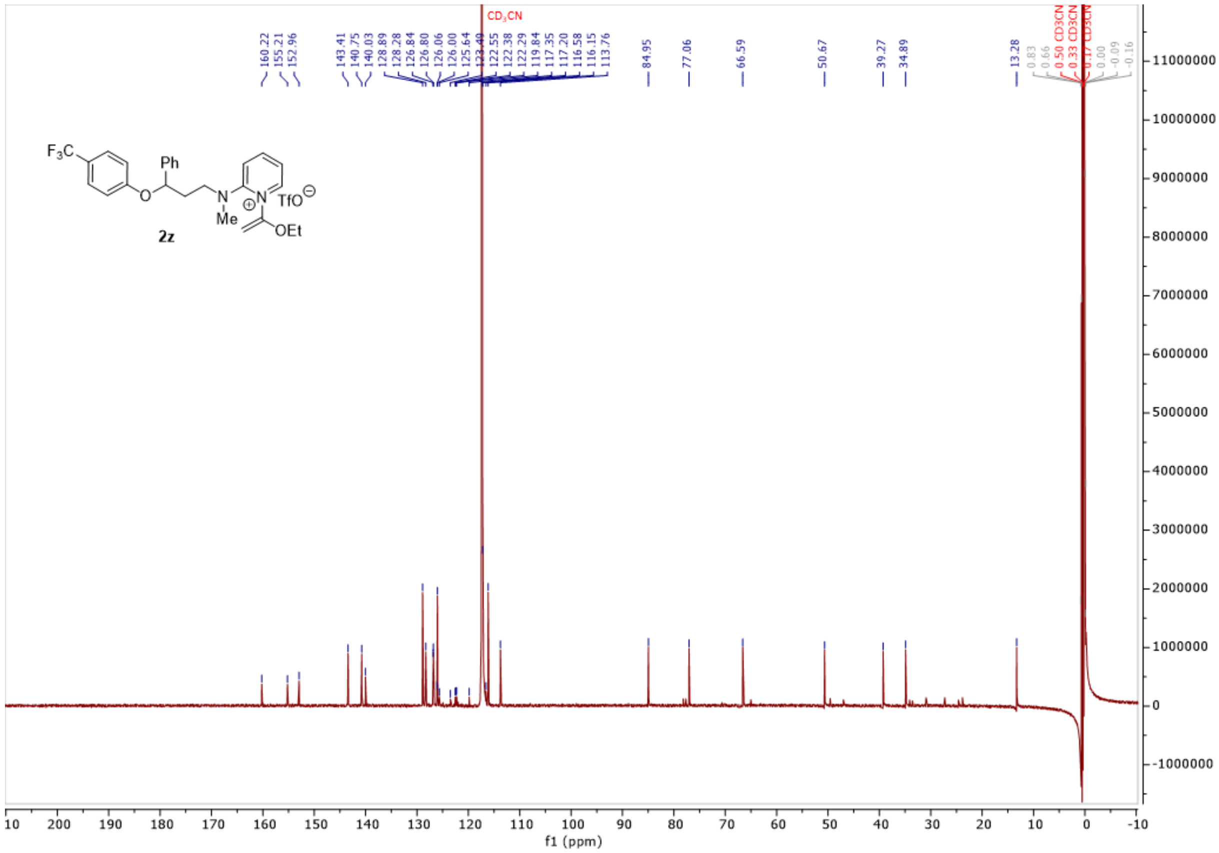

^{1}H NMR Spectrum of **2aa** (500 MHz, CD_3OD)

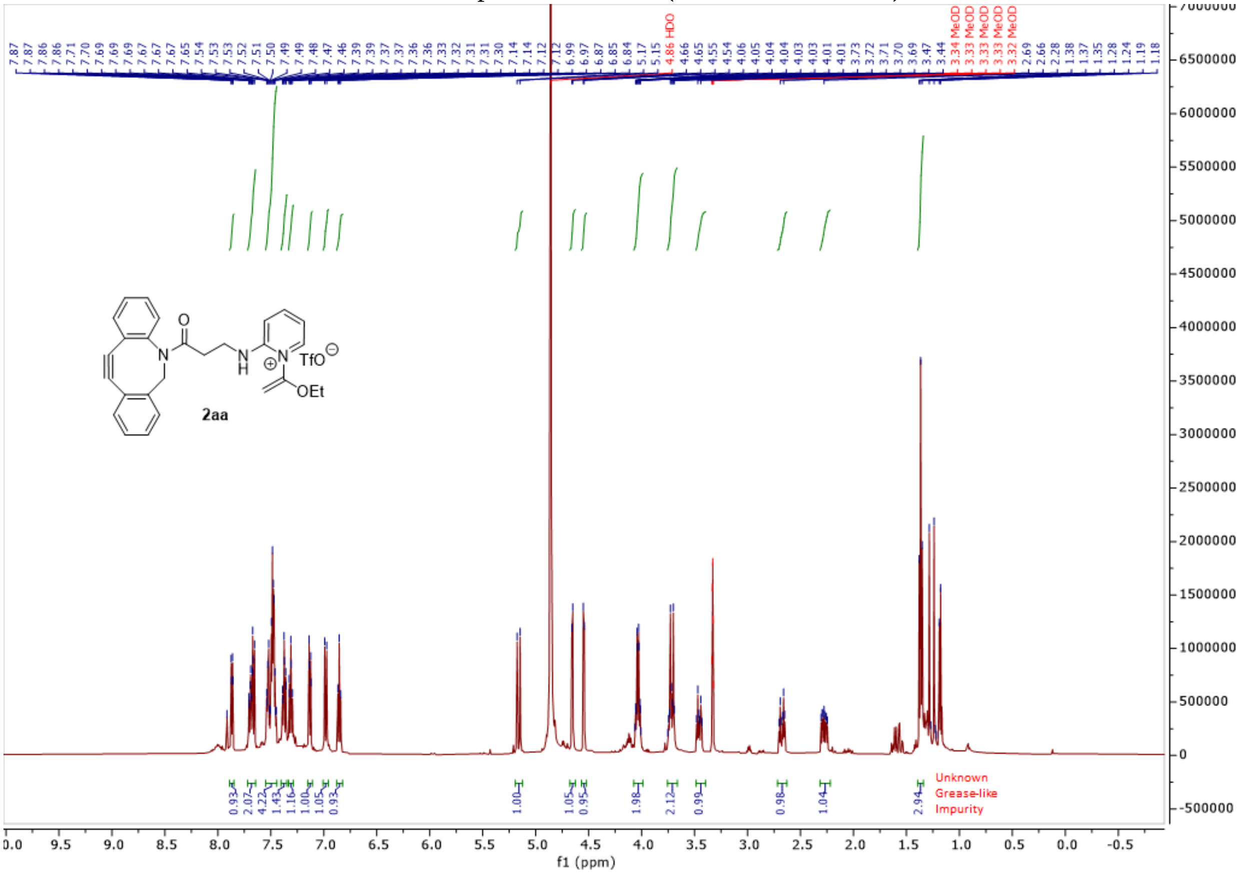

$^{13}C\{^{1}H\}$ NMR Spectrum of **2aa** (126 MHz, CD_3OD)

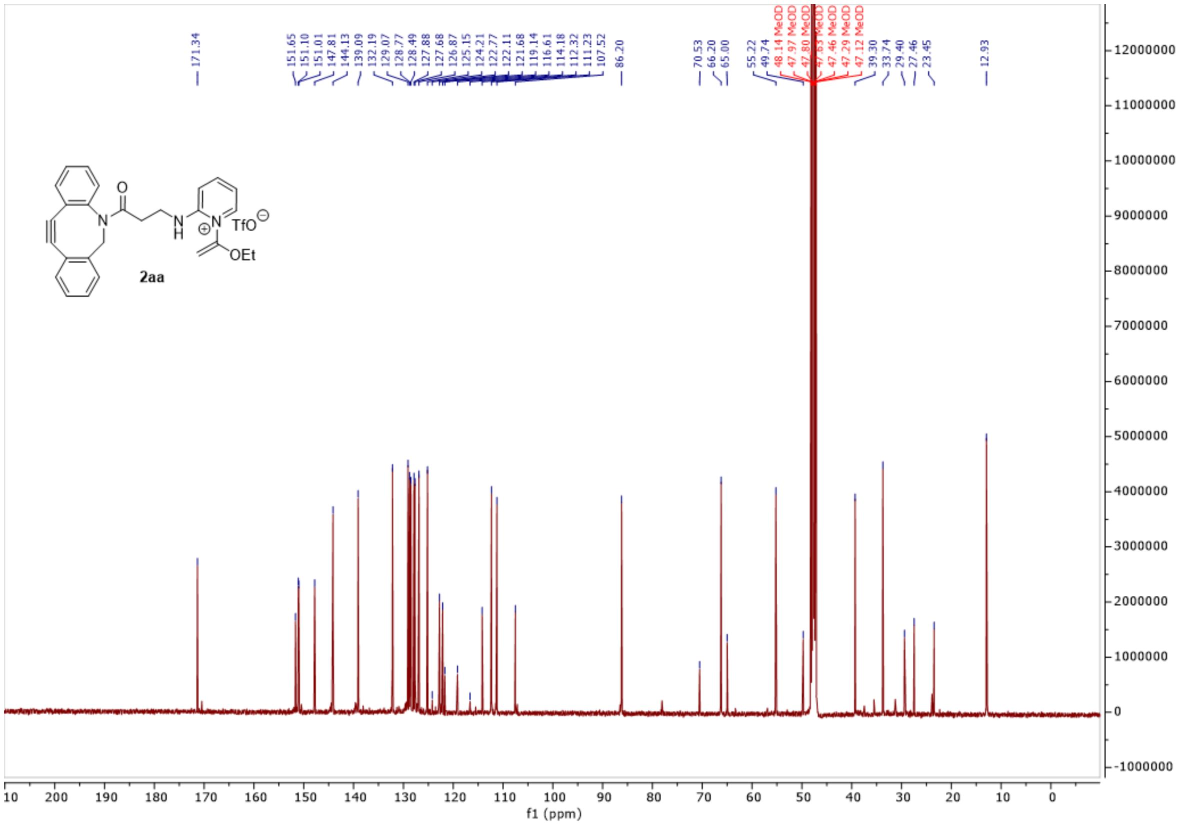

^{1}H NMR Spectrum of **4** (500 MHz, Acetone-d_6)

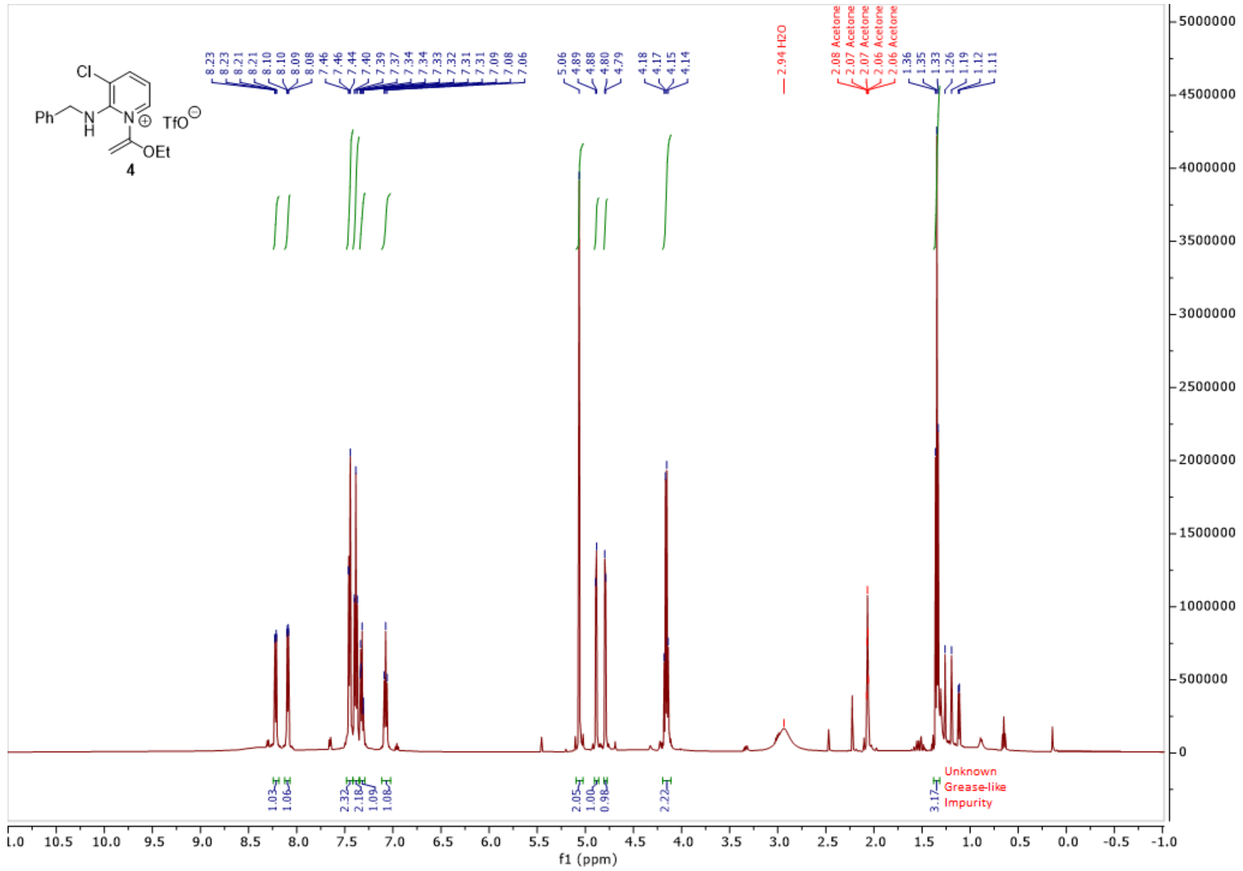

^{13}C{^{1}H} NMR Spectrum of **4** (126 MHz, Acetone-d_6)

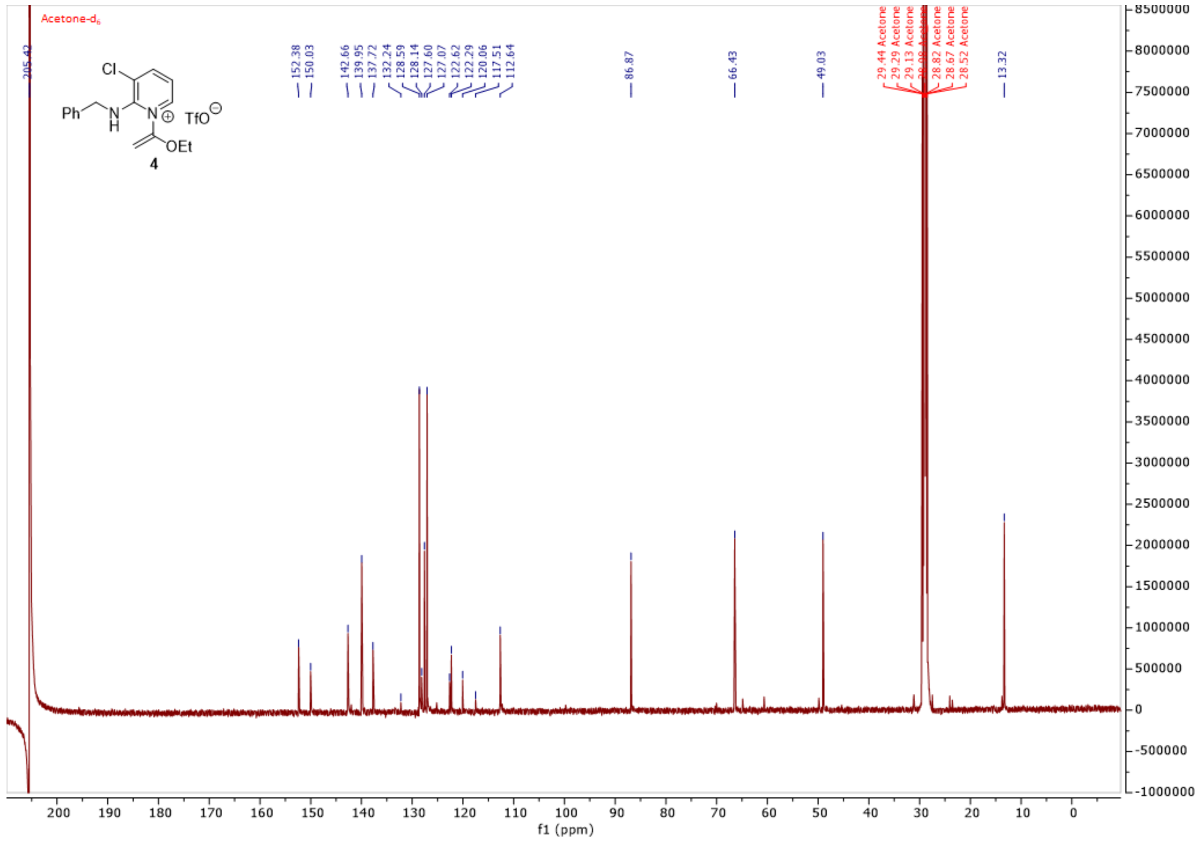

^{1}H NMR Spectrum of **5a** (500 MHz, Acetone-d_6)

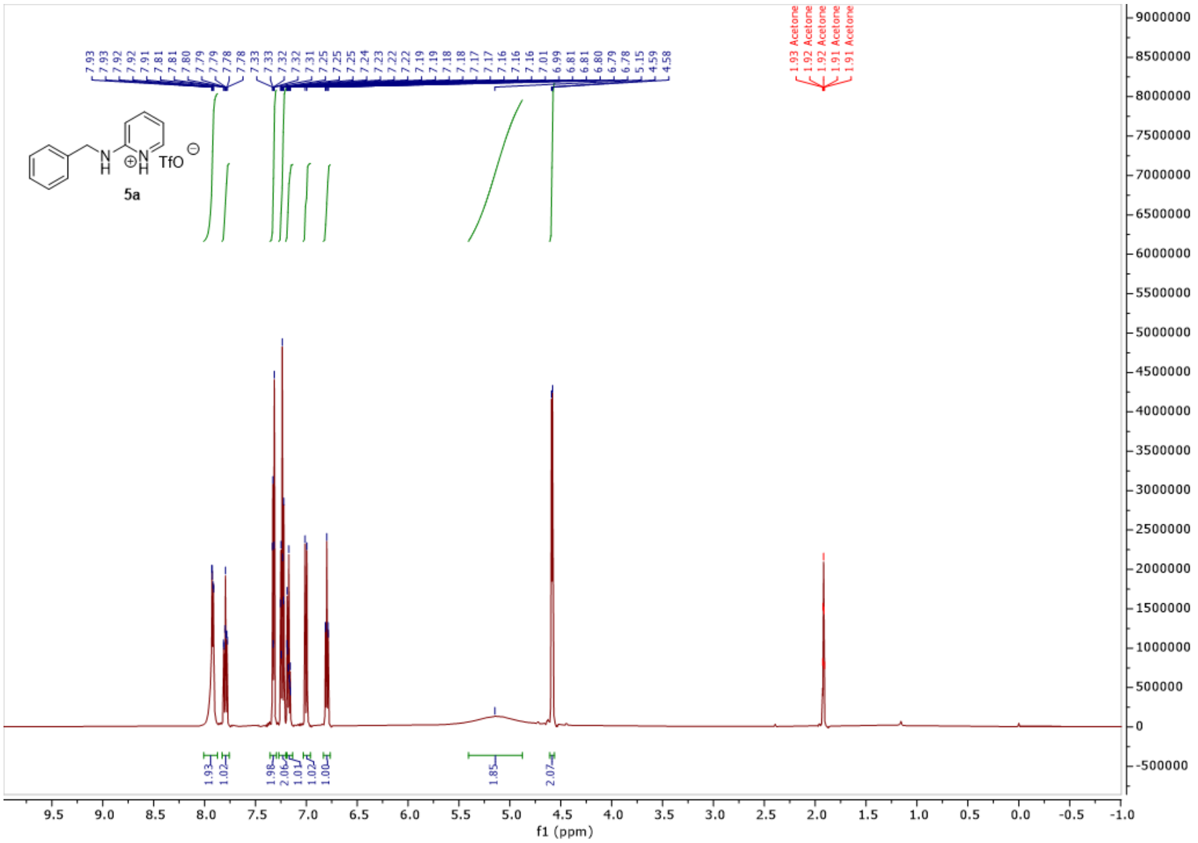

^{13}C{^{1}H} NMR Spectrum of **5a** (126 MHz, Acetone-d_6)

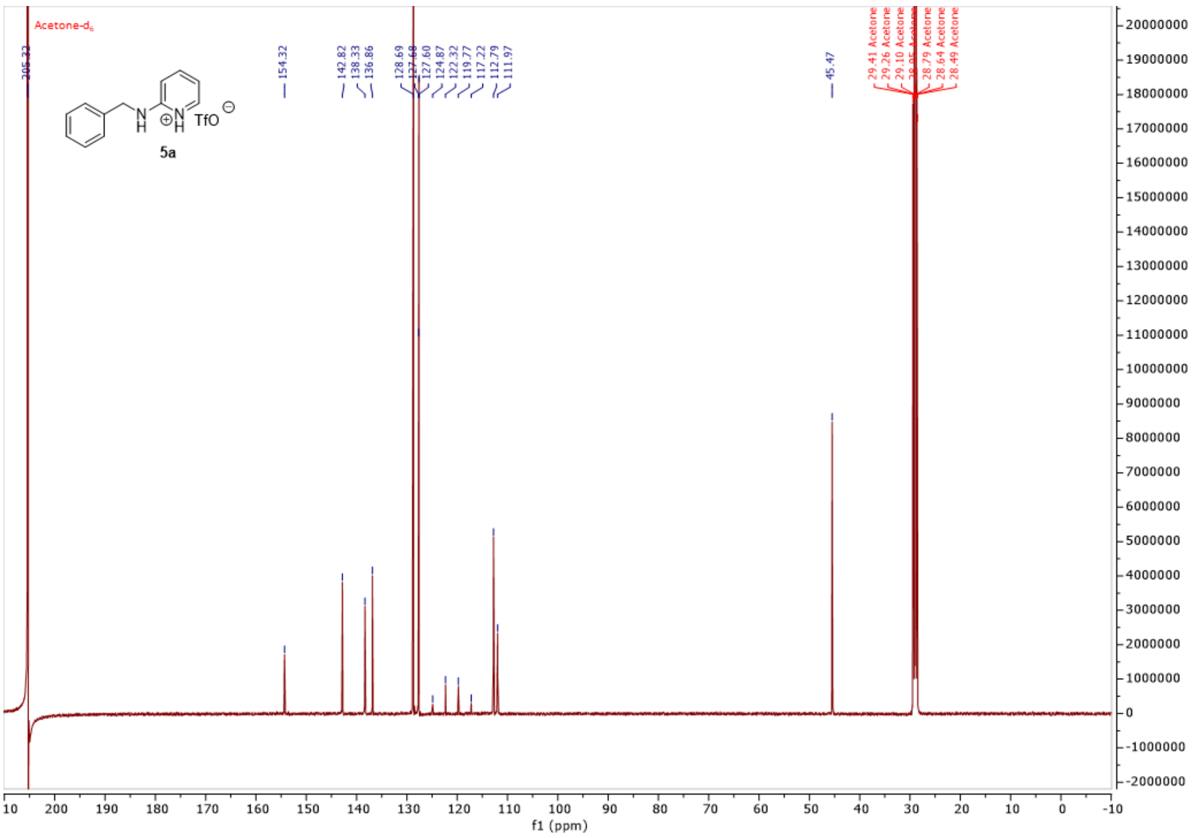

^{1}H NMR Spectrum of **5b** (500 MHz, $CDCl_3$)

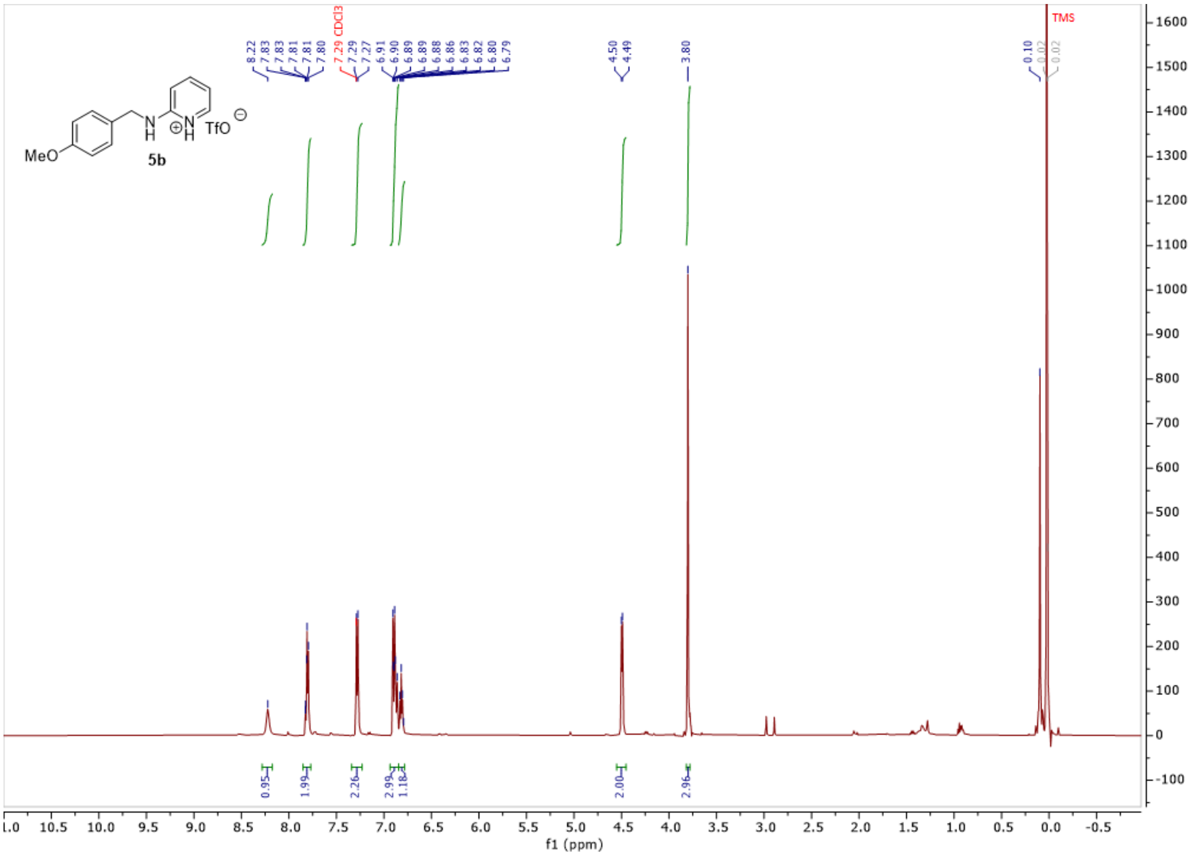

$^{13}C\{^{1}H\}$ NMR Spectrum of **5b** (126 MHz, Acetone-d_6)

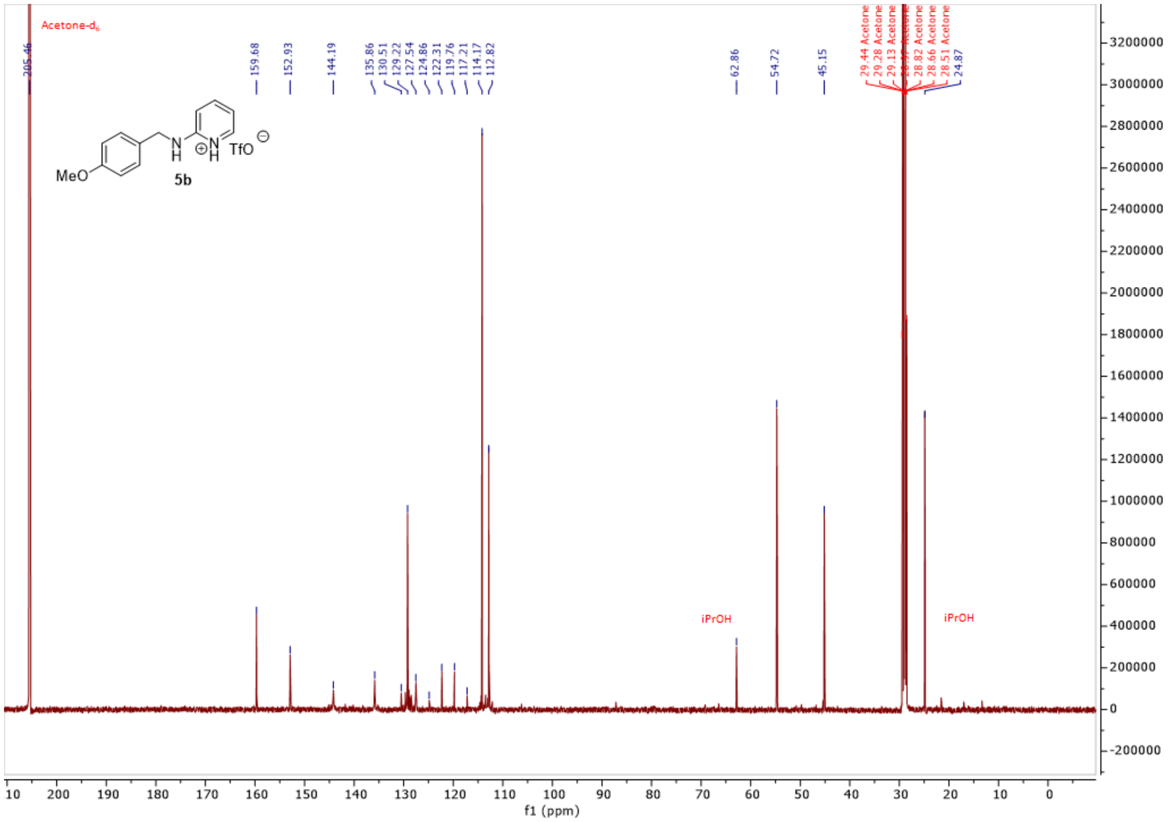

^{1}H NMR Spectrum of **5g** (500 MHz, $CDCl_3$)

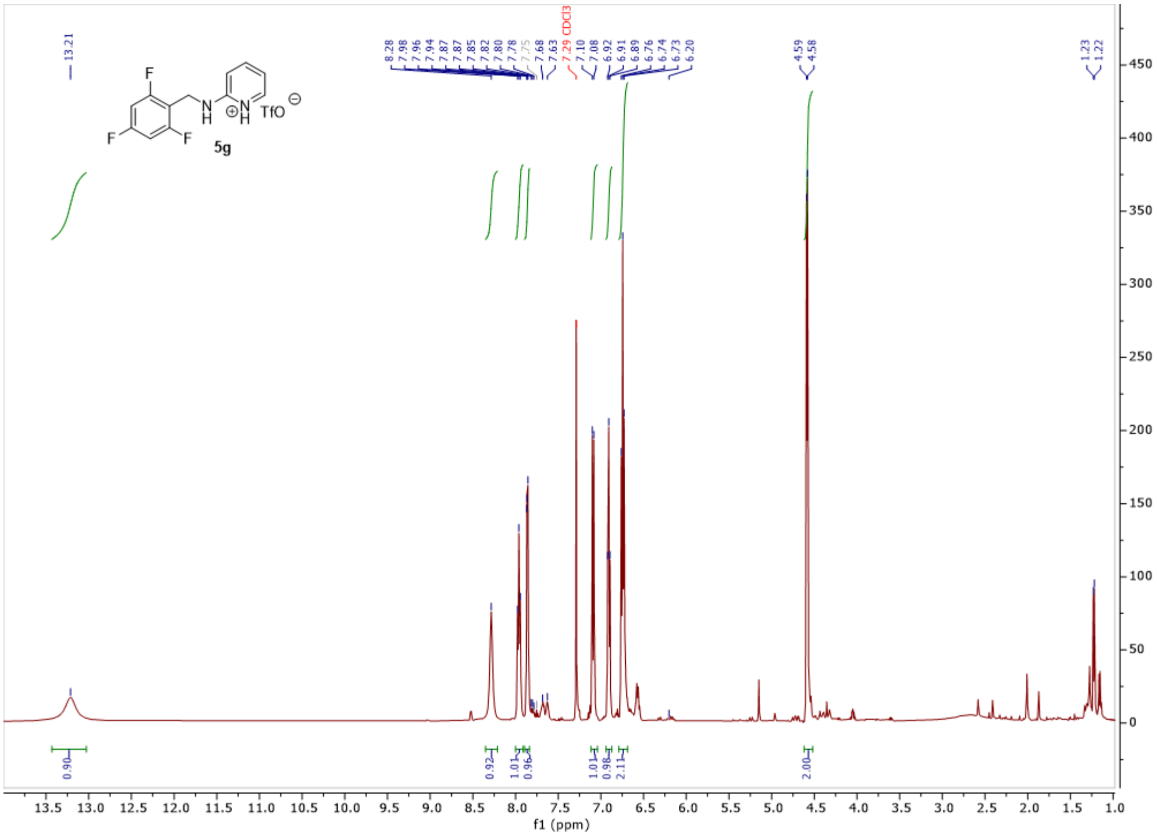

$^{13}C\{1H\}$ NMR Spectrum of **5g** (126 MHz, Acetone-d_6)

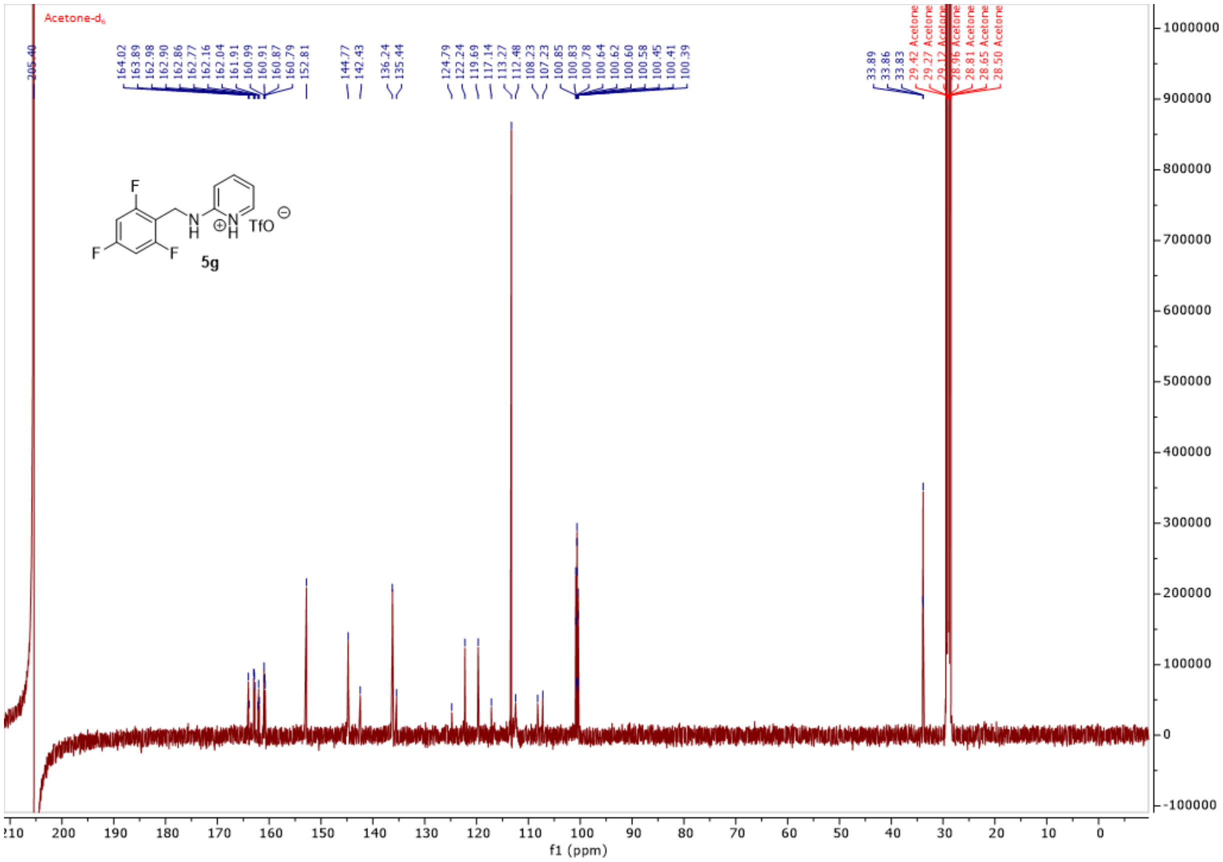

^{1}H NMR Spectrum of **5o** (500 MHz, $CDCl_3$)

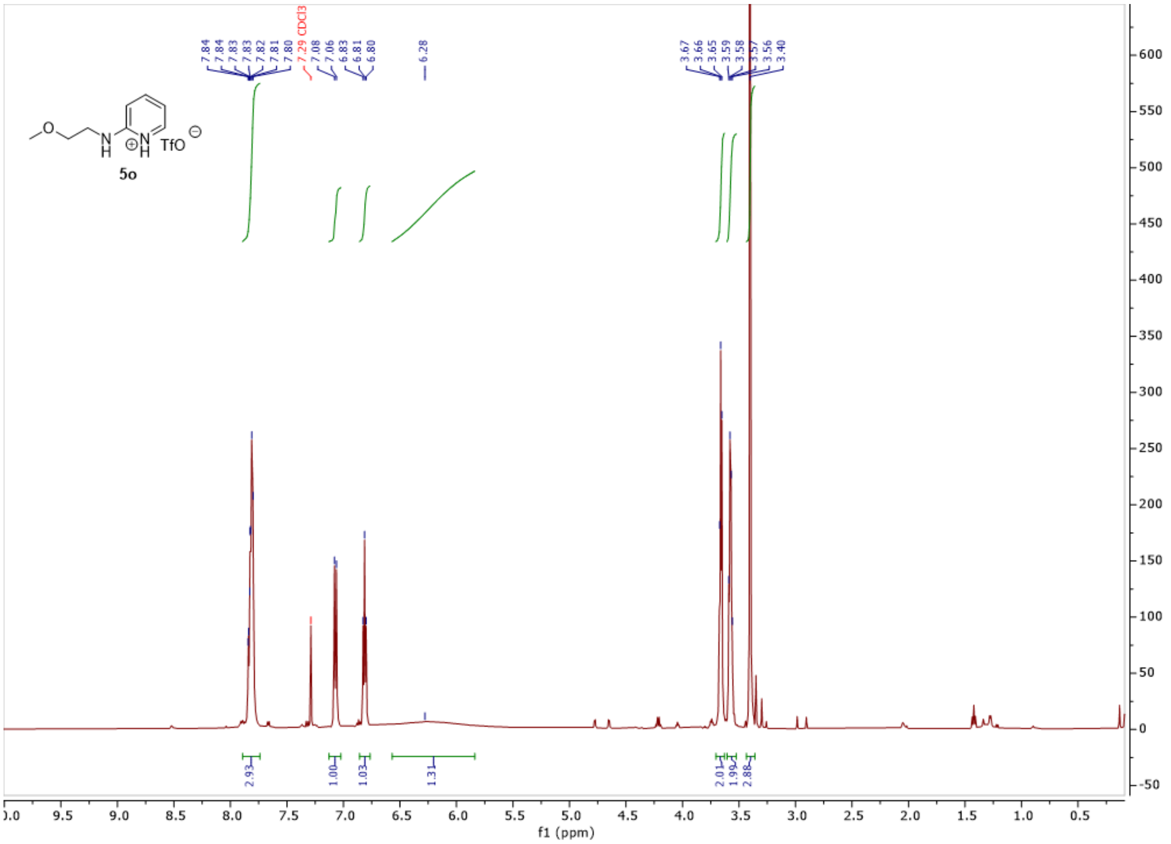

$^{13}C\{1H\}$ NMR Spectrum of **5o** (126 MHz, Acetone-d_6)

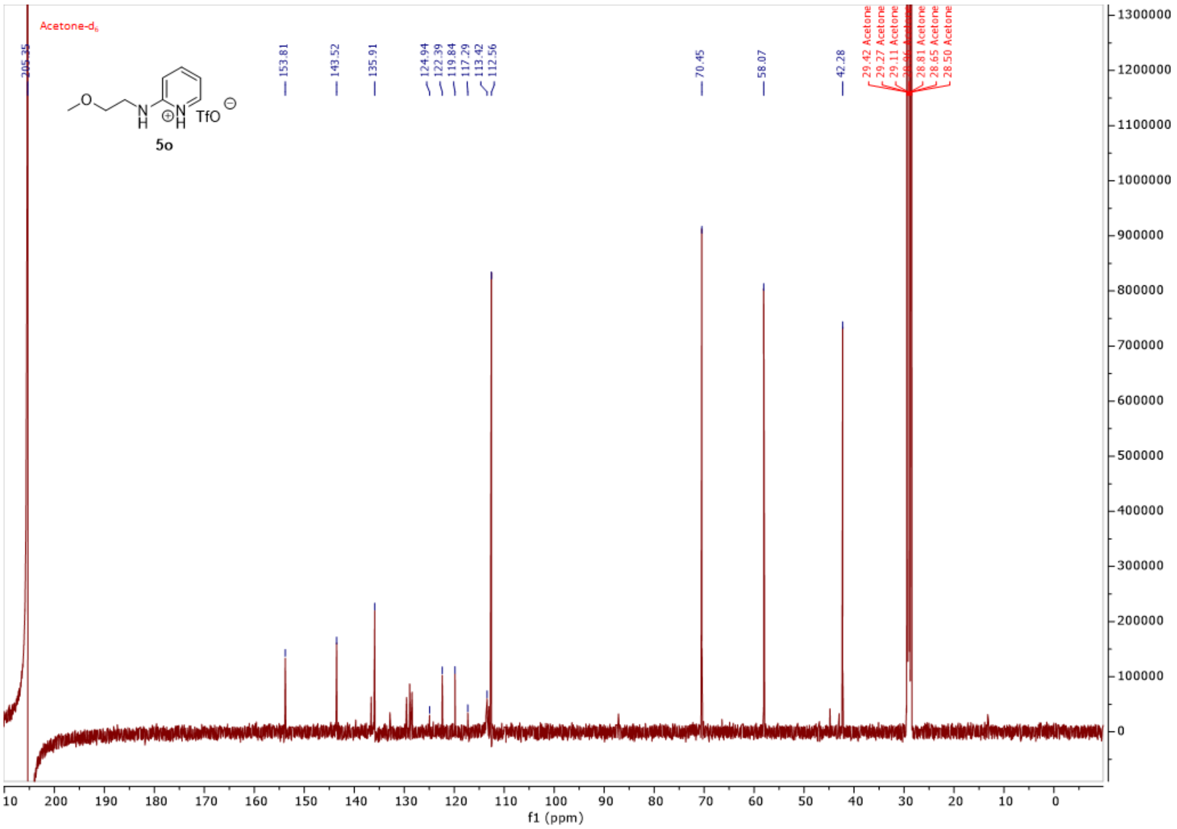

^{1}H NMR Spectrum of **5s** (500 MHz, $CDCl_3$)

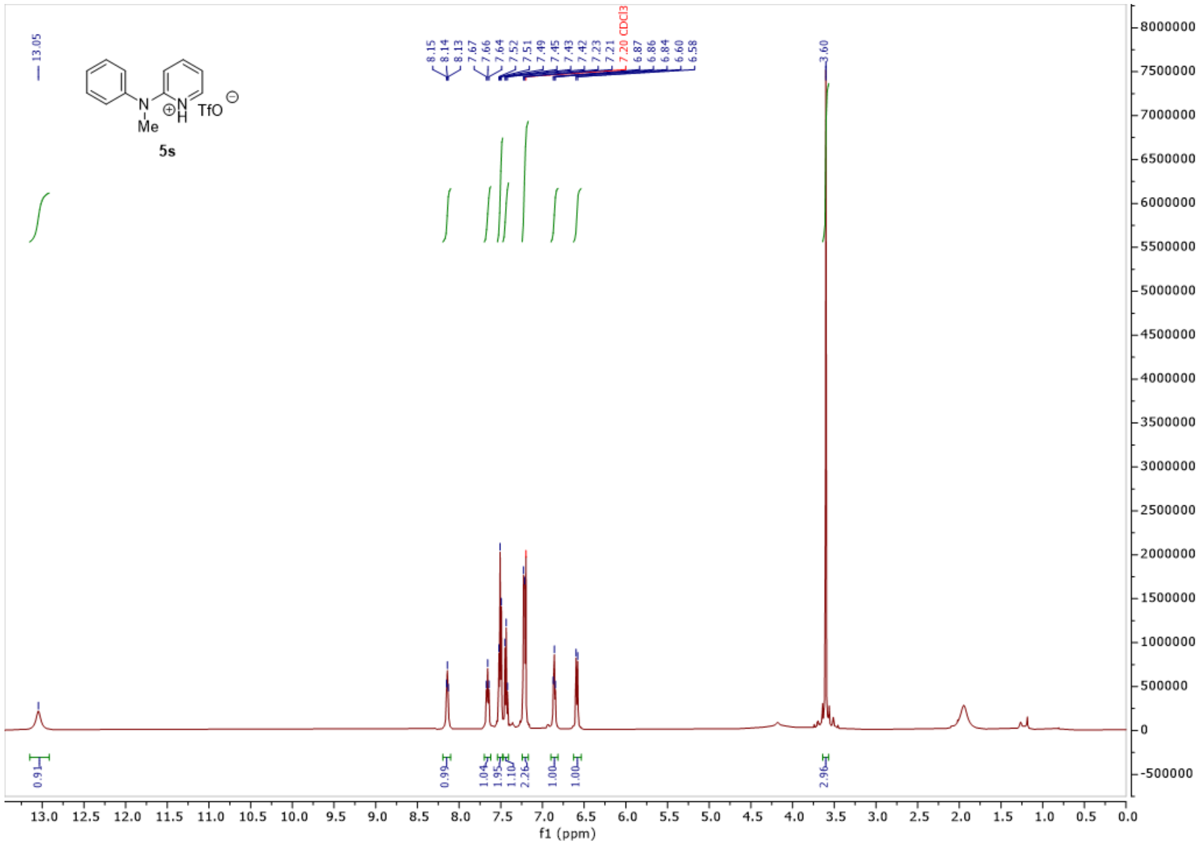

$^{13}C\{1H\}$ NMR Spectrum of **5s** (126 MHz, $CDCl_3$)

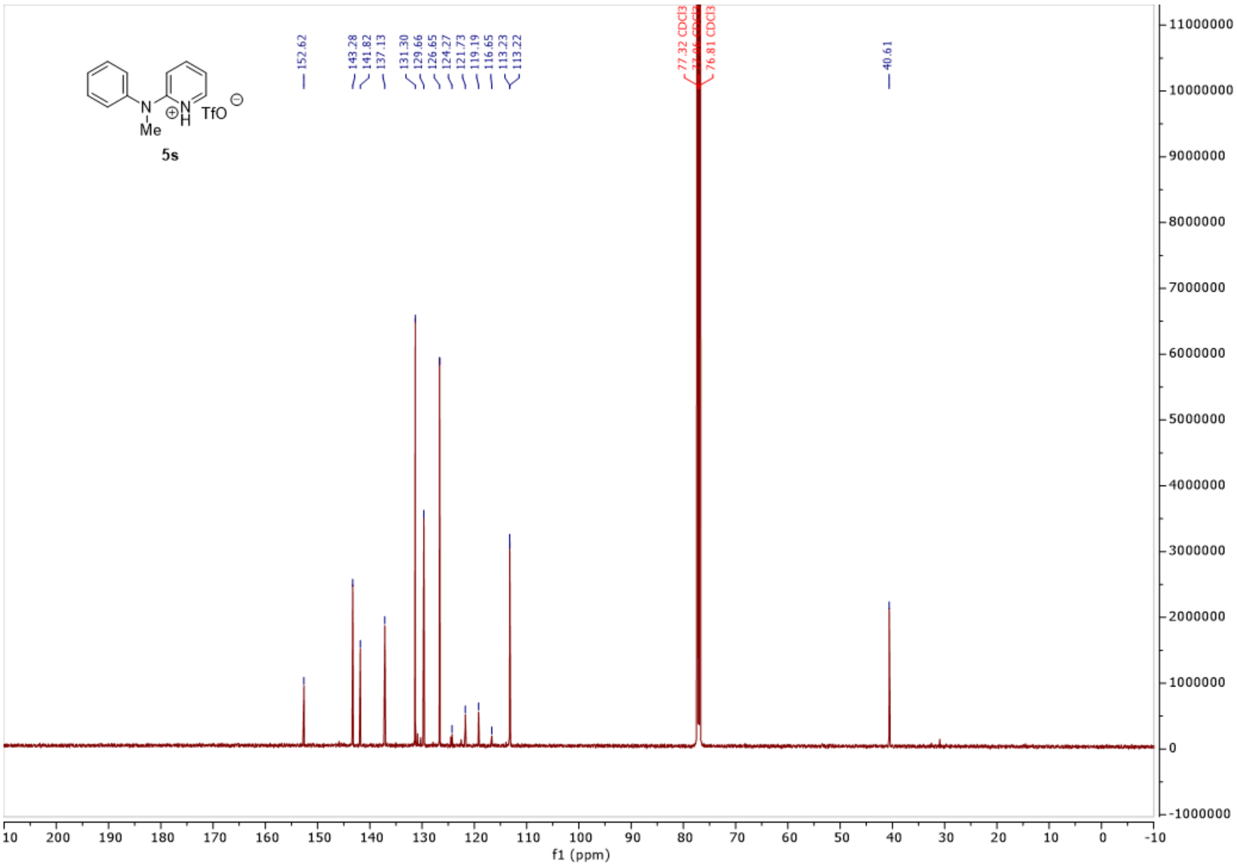

^{1}H NMR Spectrum of **5u** (500 MHz, $CDCl_3$)

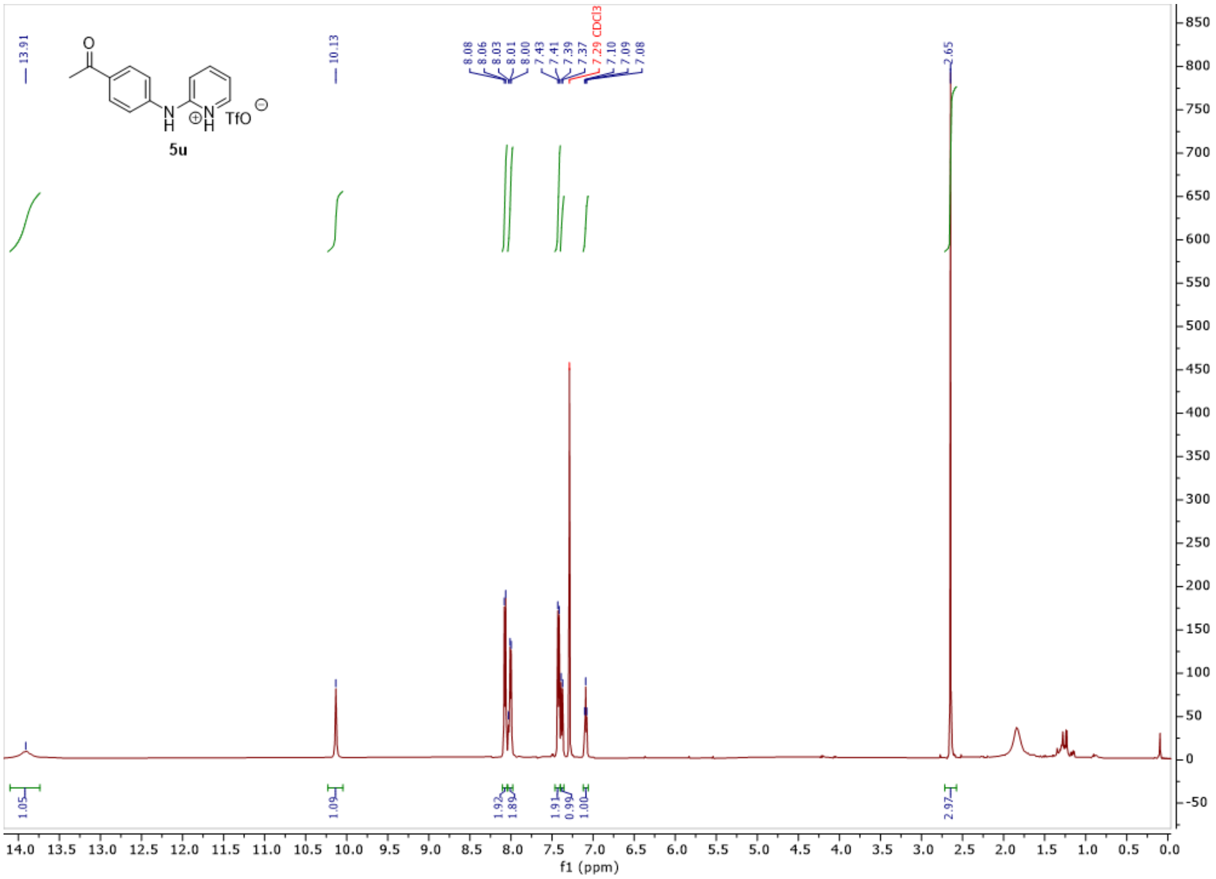

^{13}C{1H} NMR Spectrum of **5u** (126 MHz, Acetone-d_6)

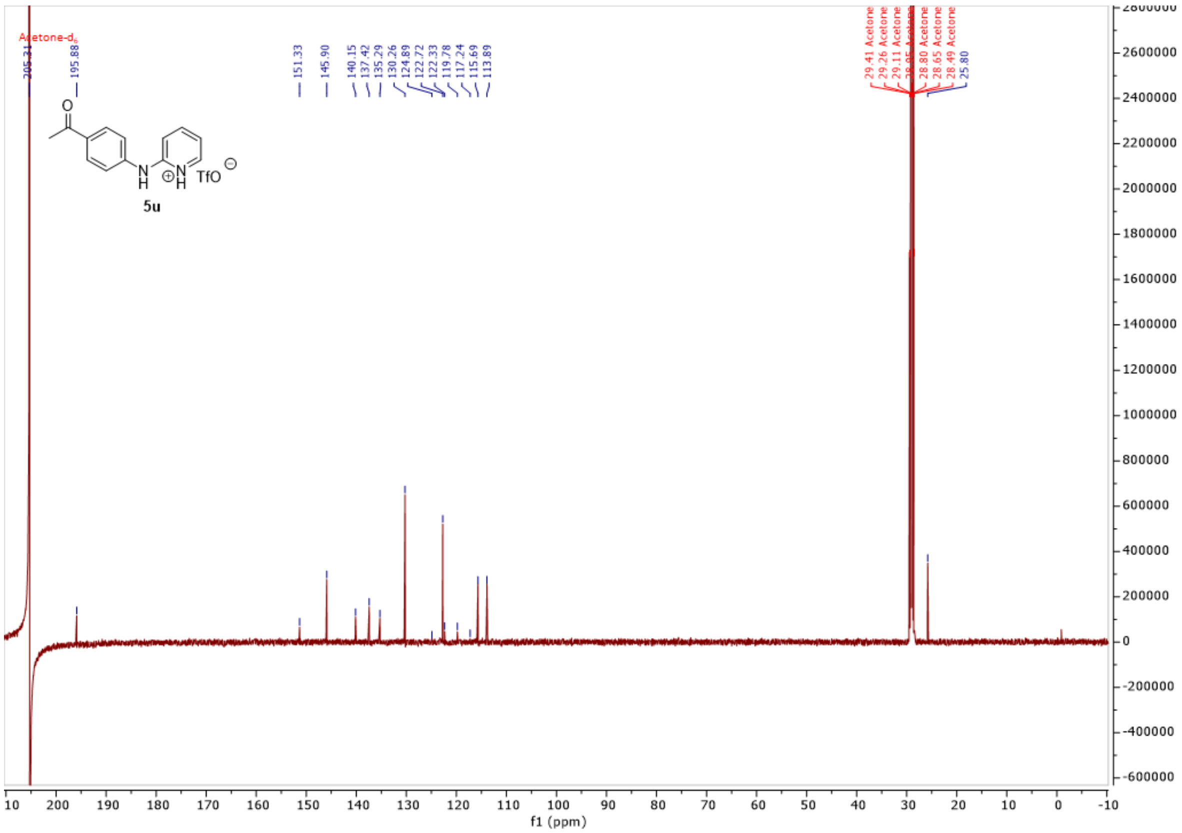

^{1}H NMR Spectrum of **6f** (500 MHz, Acetone-d_6)

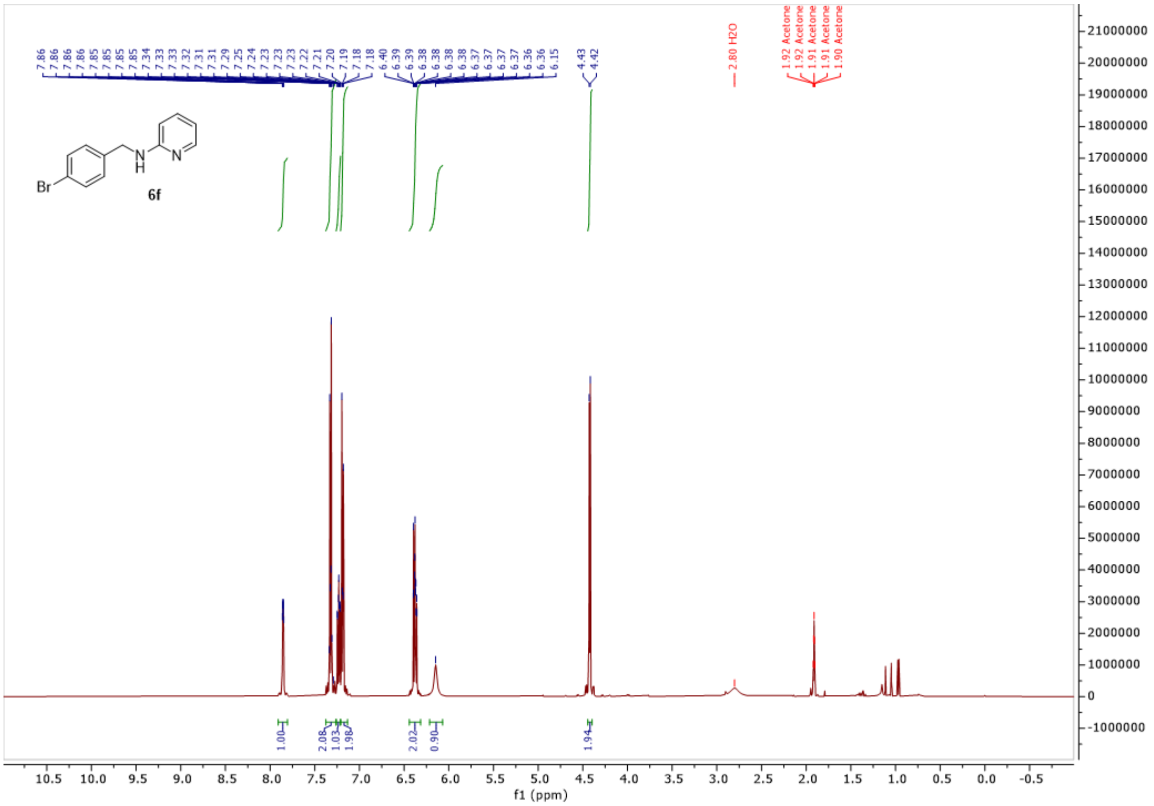

$^{13}C\{1H\}$ NMR Spectrum of **6f** (126 MHz, Acetone-d_6)

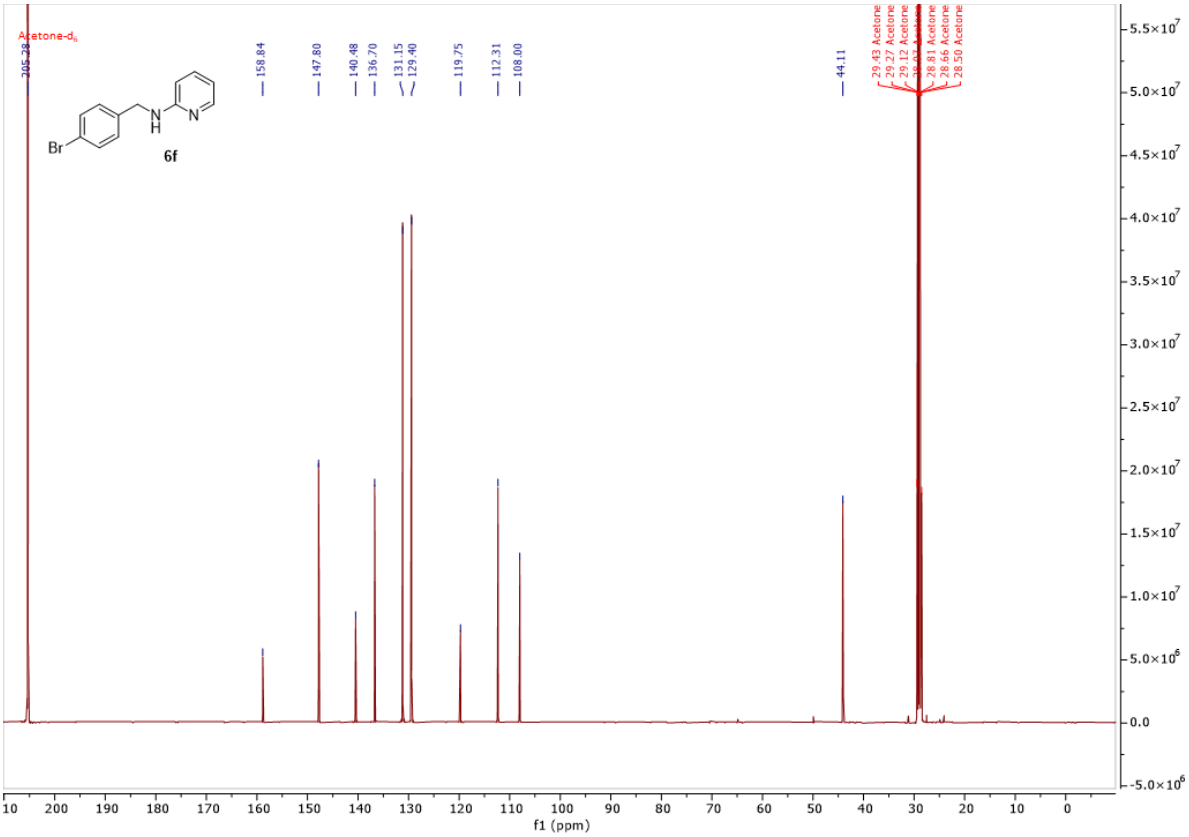

^{1}H NMR Spectrum of **8** (500 MHz, Acetone-d_6)

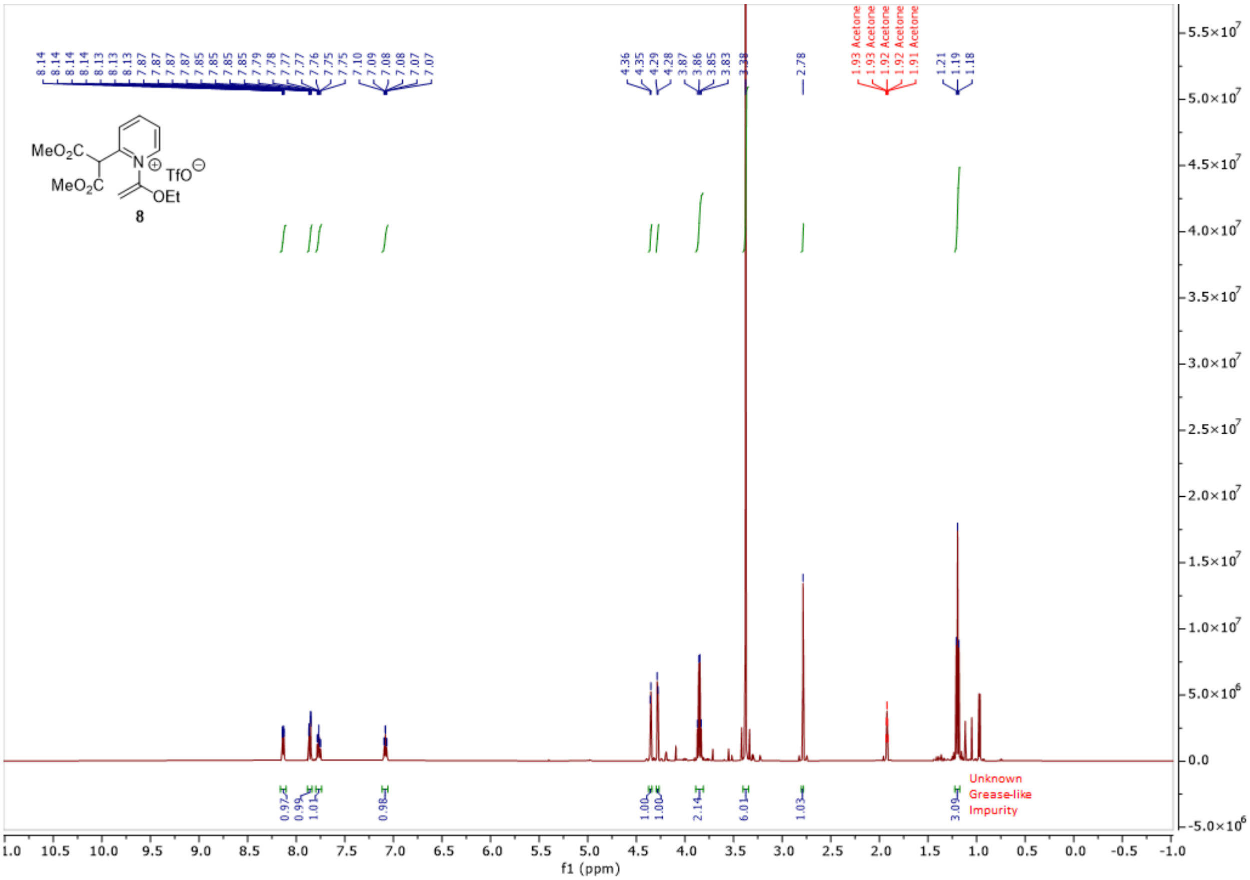

$^{13}C\{1H\}$ NMR Spectrum of **8** (126 MHz, Acetone-d_6)

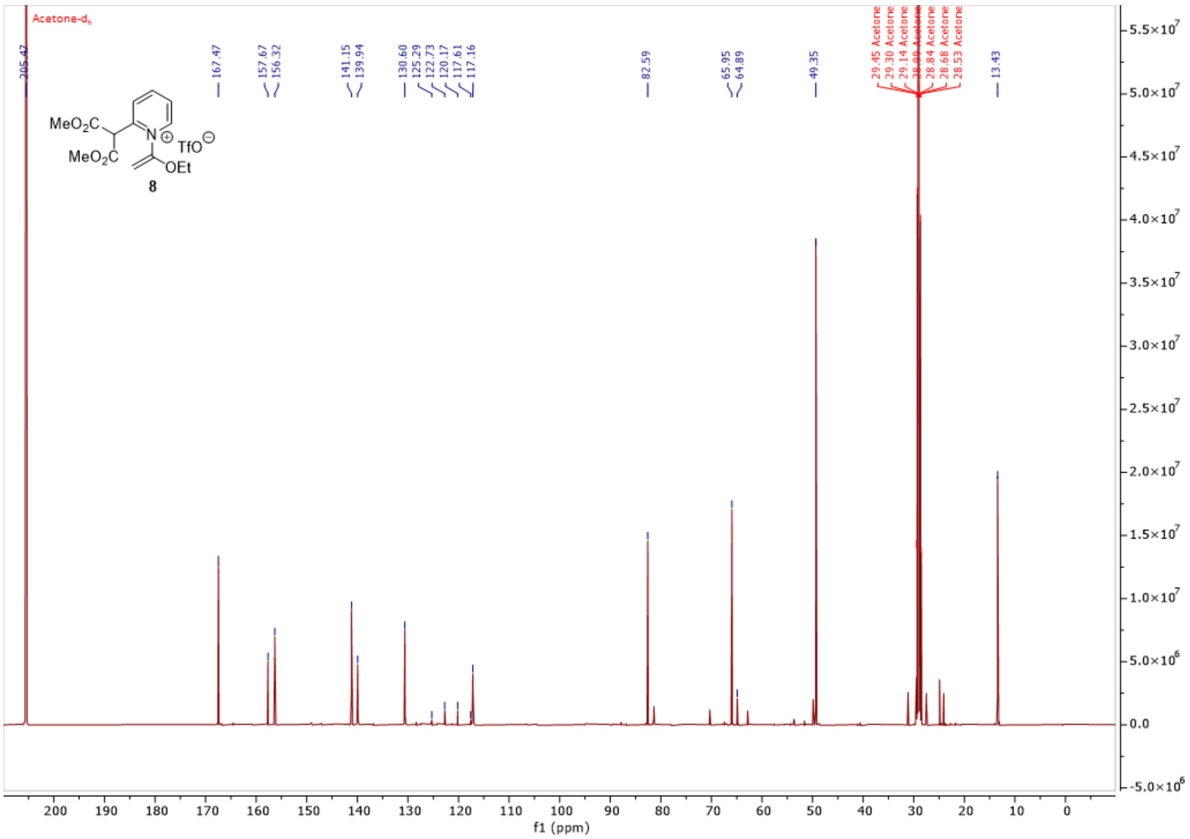

^{1}H NMR Spectrum of **9** (500 MHz, $CDCl_3$)

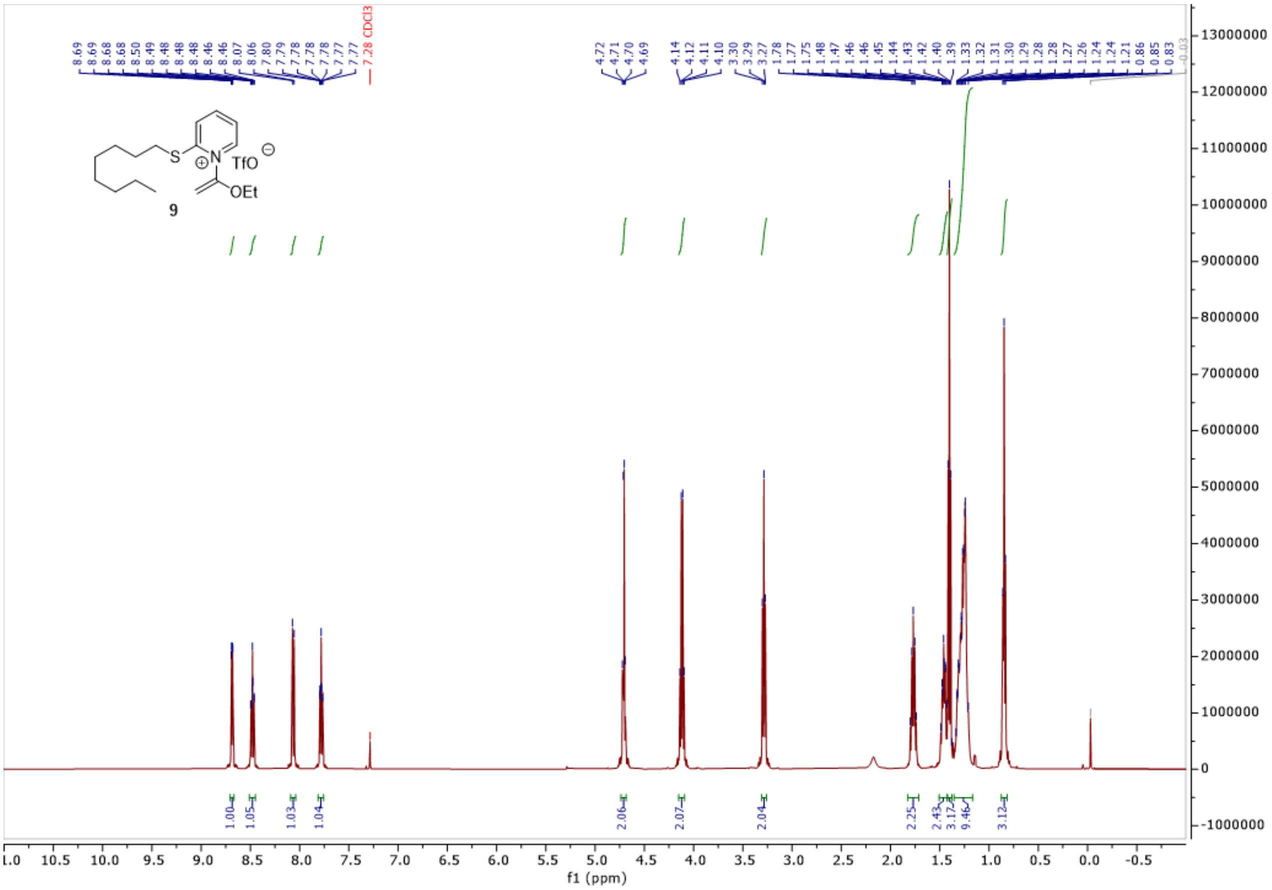

$^{13}C\{1H\}$ NMR Spectrum of **9** (126 MHz, $CDCl_3$)

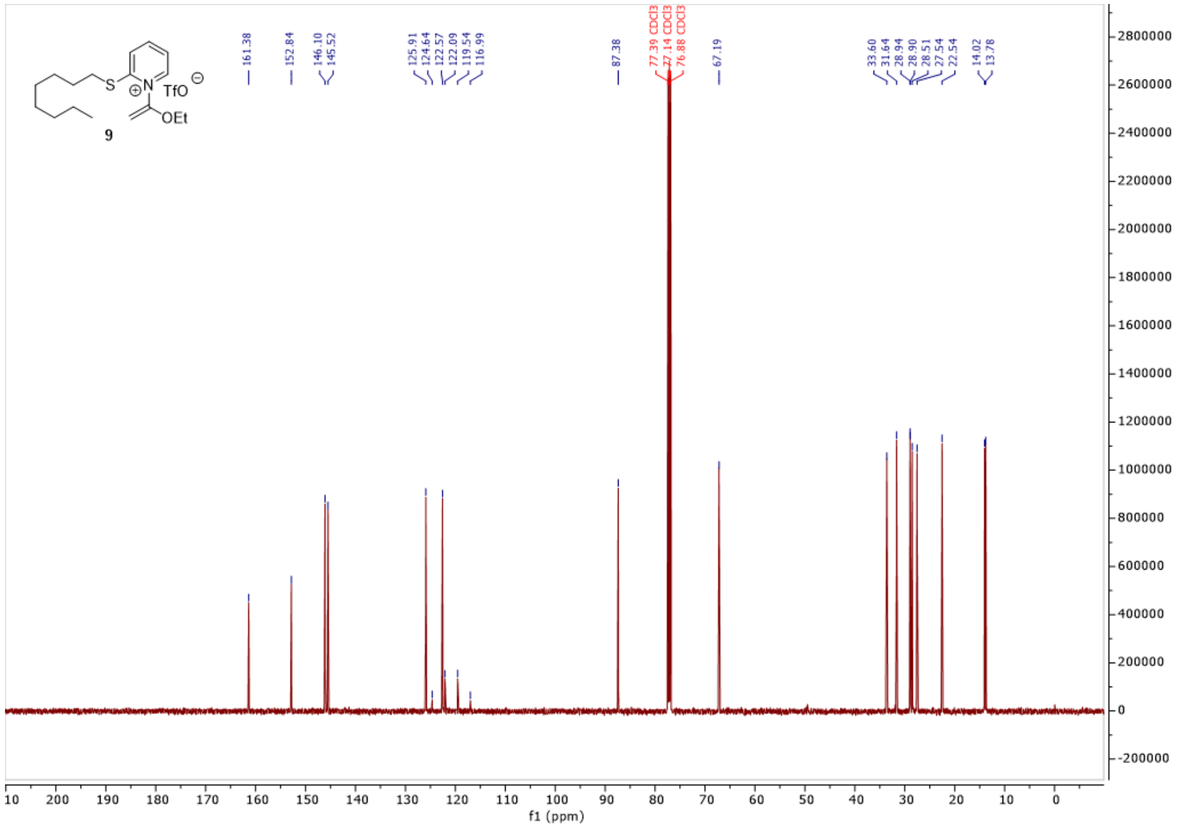

^{1}H NMR Spectrum of **10** (500 MHz, $CDCl_3$)

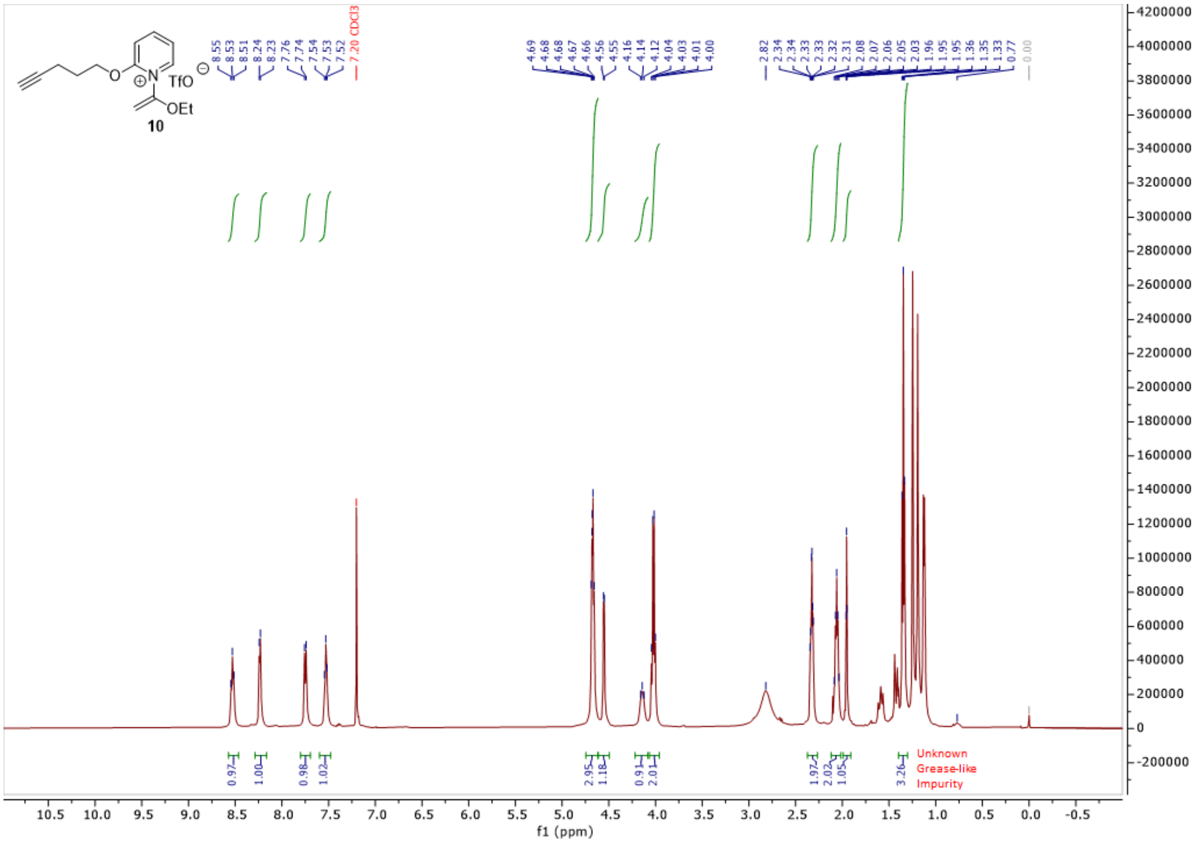

^{13}C{1H} NMR Spectrum of **10** (126 MHz, $CDCl_3$)

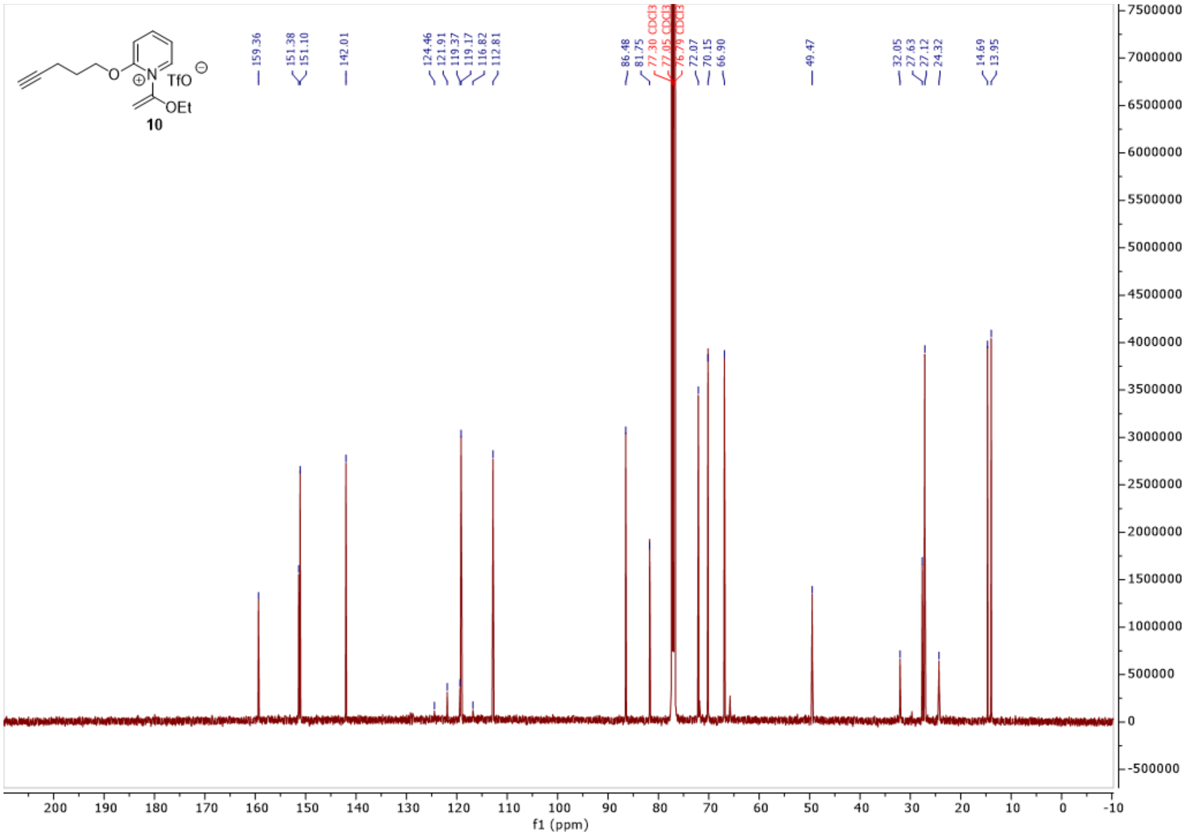

^{1}H NMR Spectrum of **11** (500 MHz, Acetone-d_6)

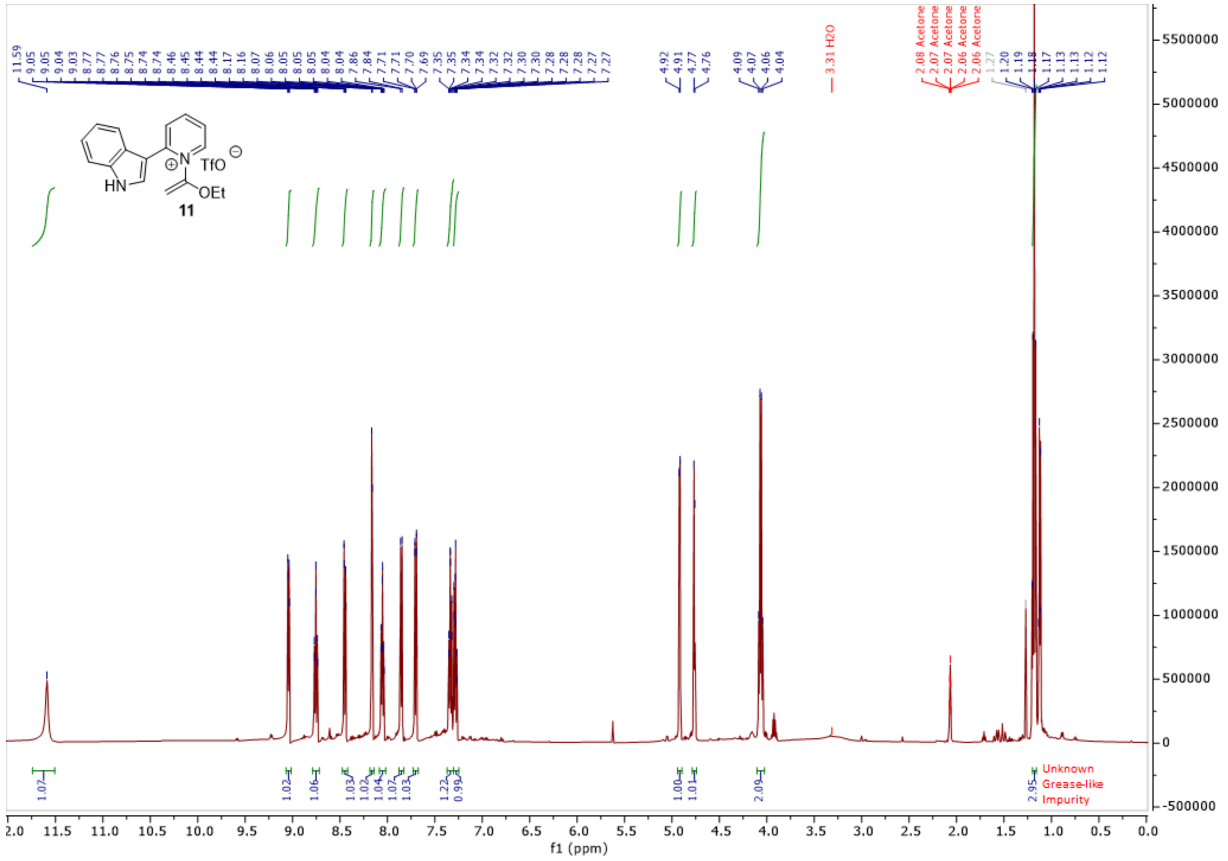

$^{13}C\{1H\}$ NMR Spectrum of **11** (126 MHz, Acetone-d_6)

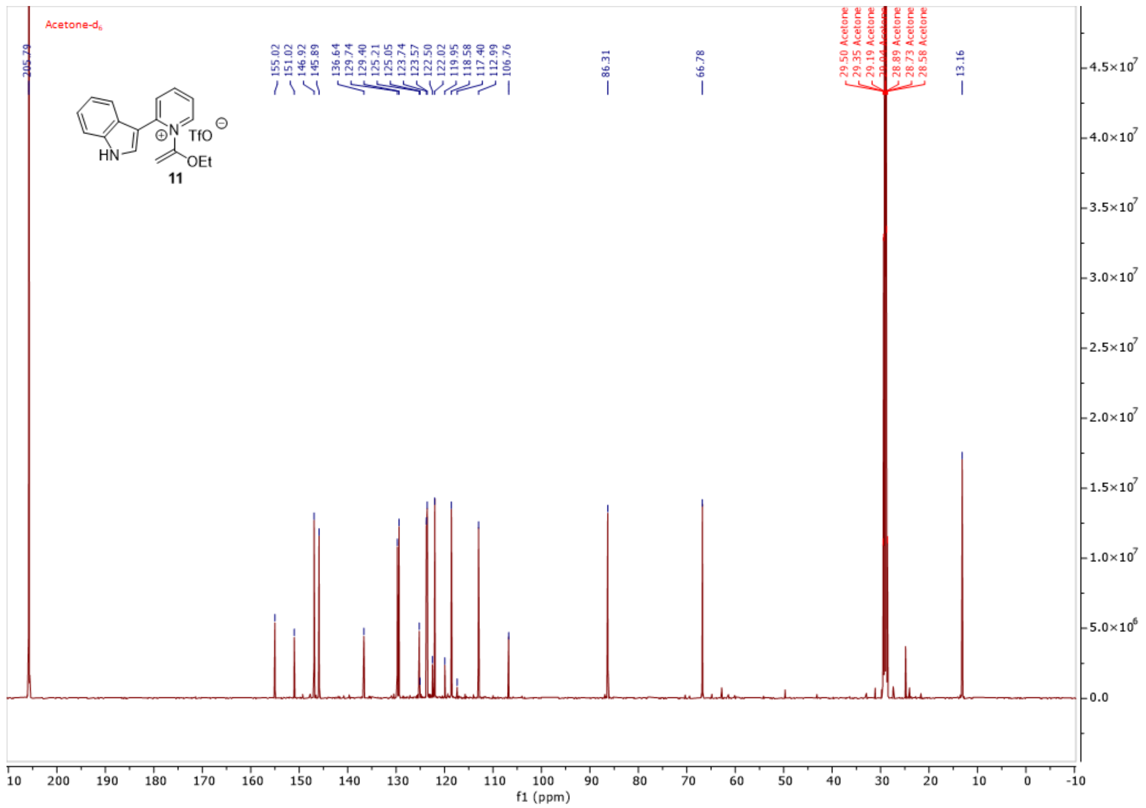

^{1}H NMR Spectrum of **14** (500 MHz, Acetone-d_6)

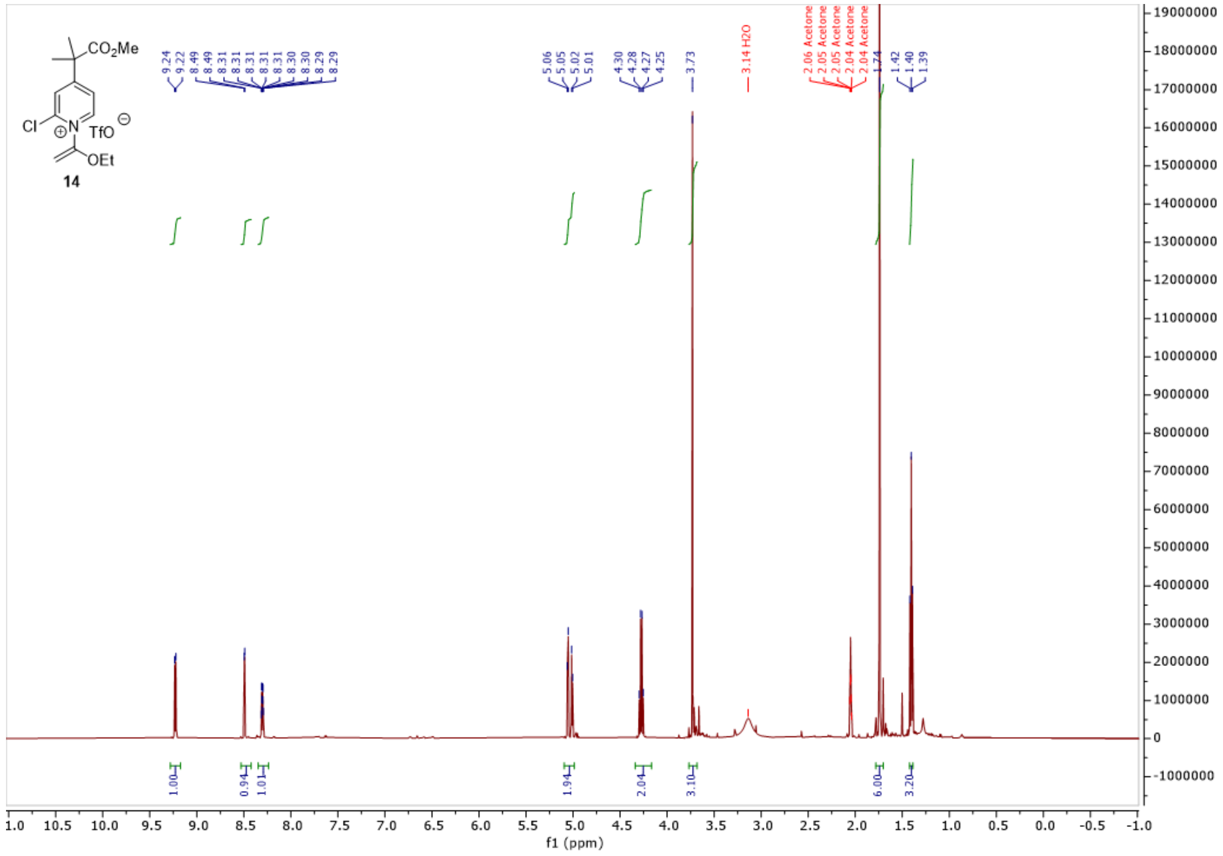

^{13}C{1H} NMR Spectrum of **14** (126 MHz, Acetone-d_6)

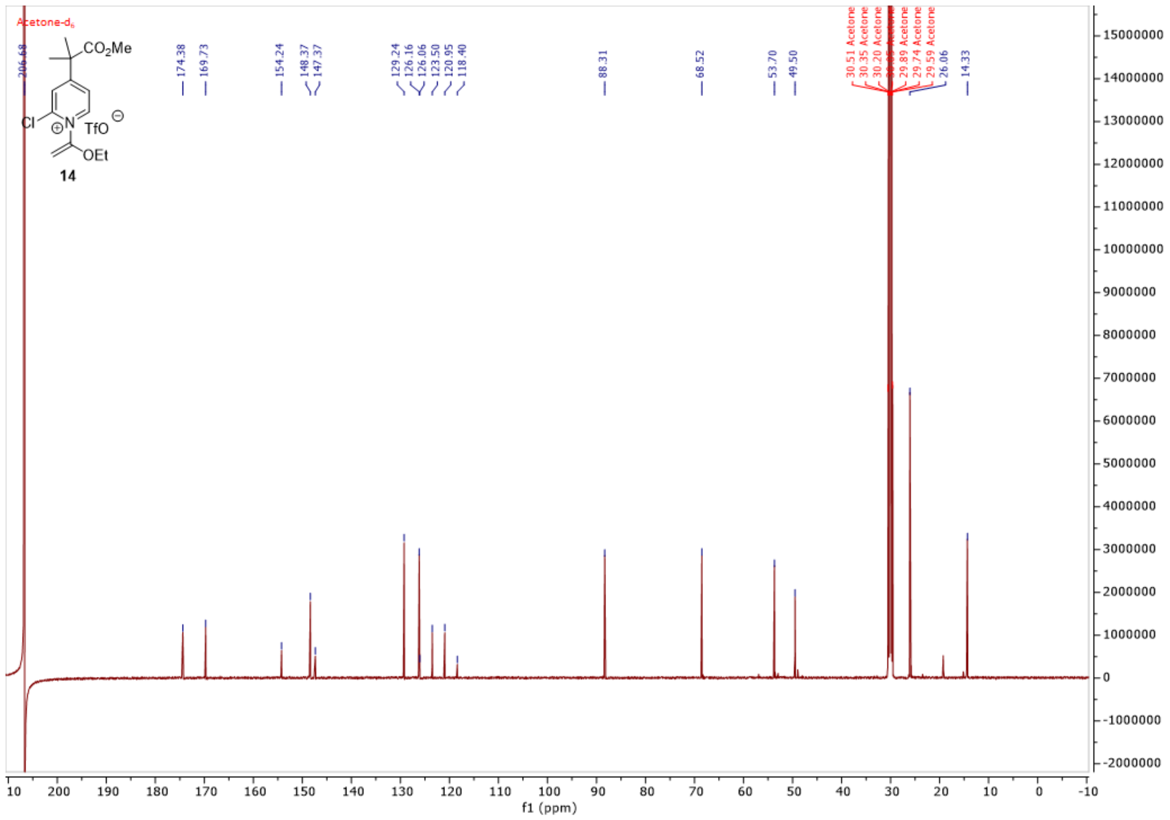

^{1}H NMR Spectrum of **15** (500 MHz, Acetone-d_6)

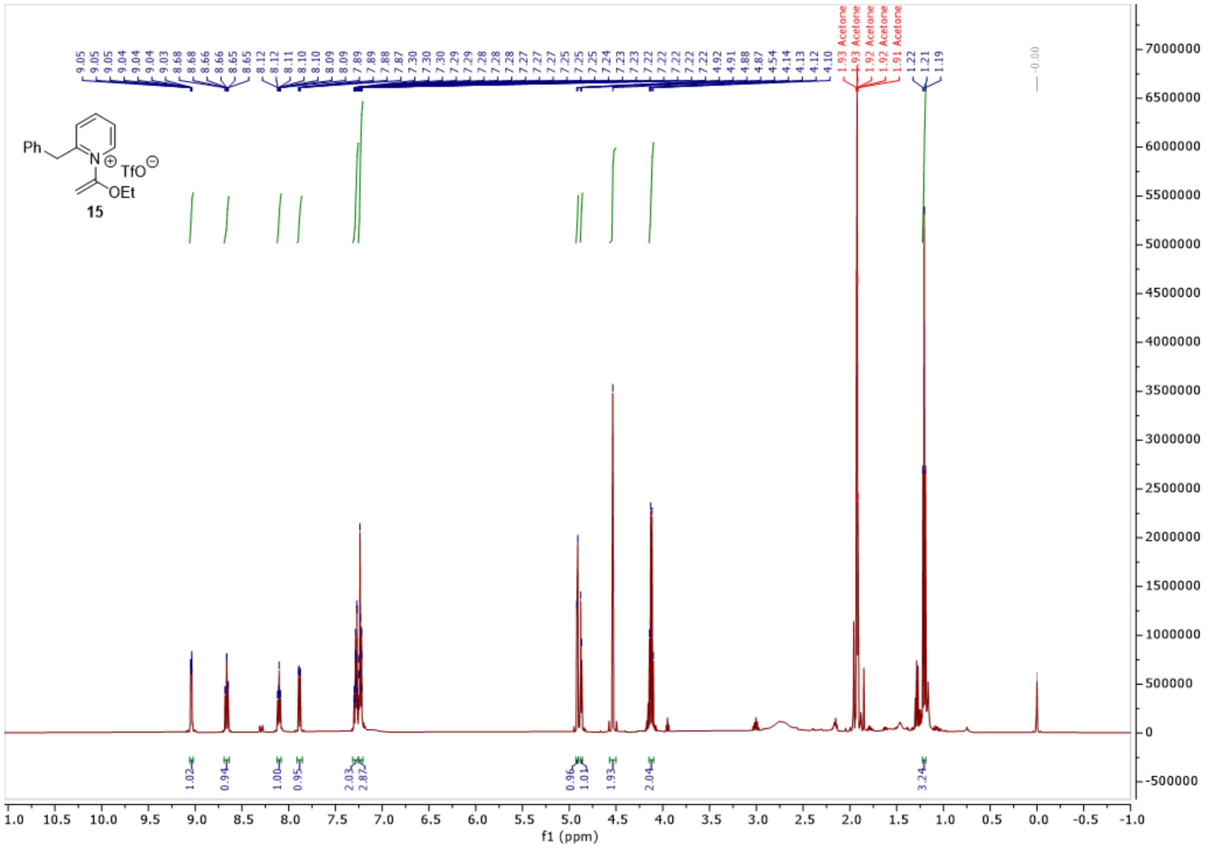

^{13}C{1H} NMR Spectrum of **15** (126 MHz, Acetone-d_6)

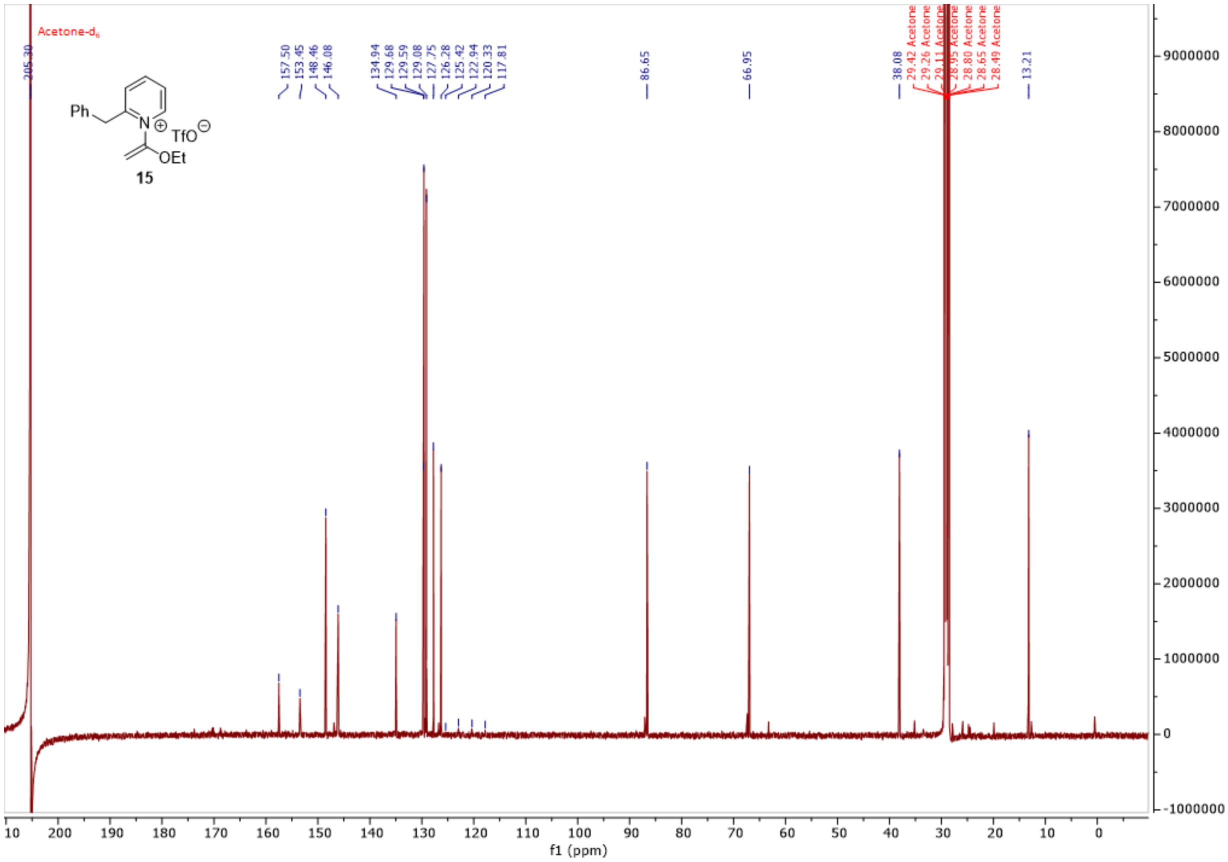

V. Representative qNMR spectra

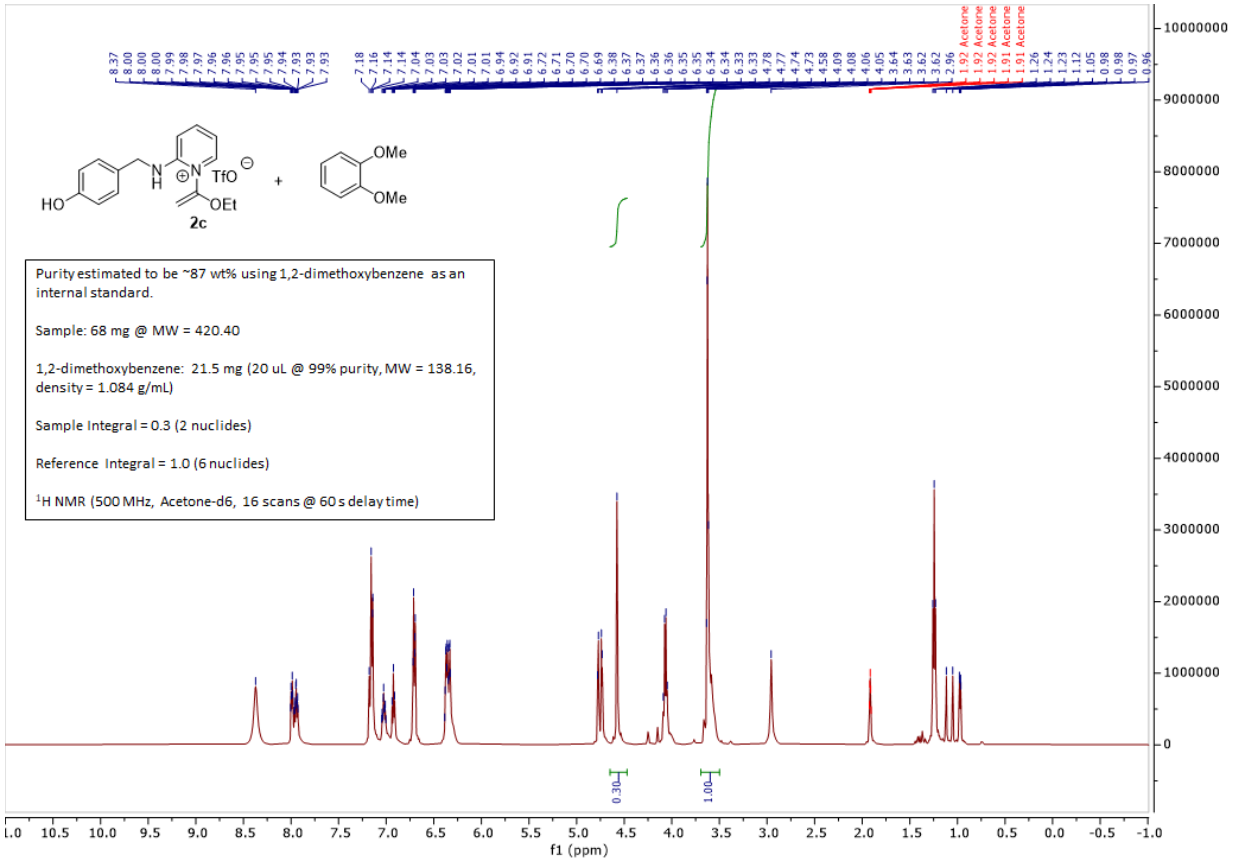

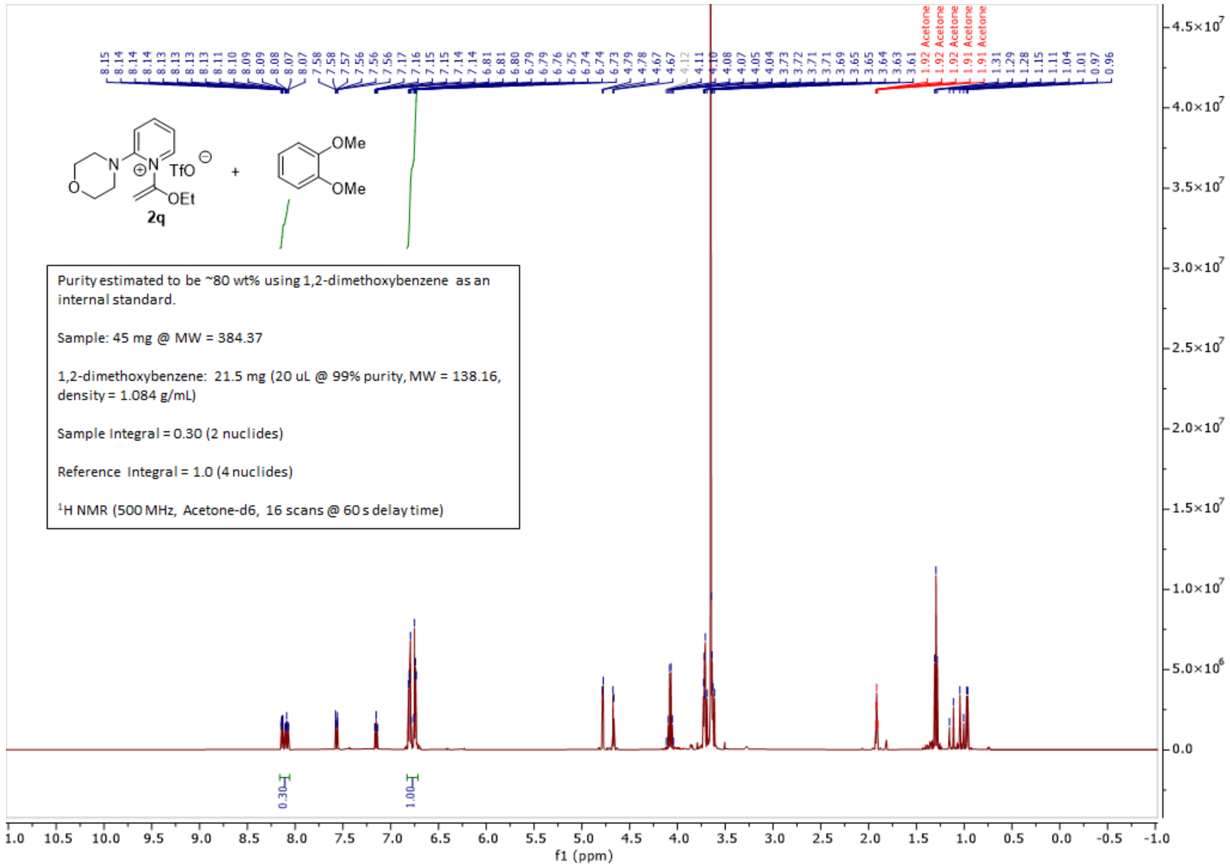

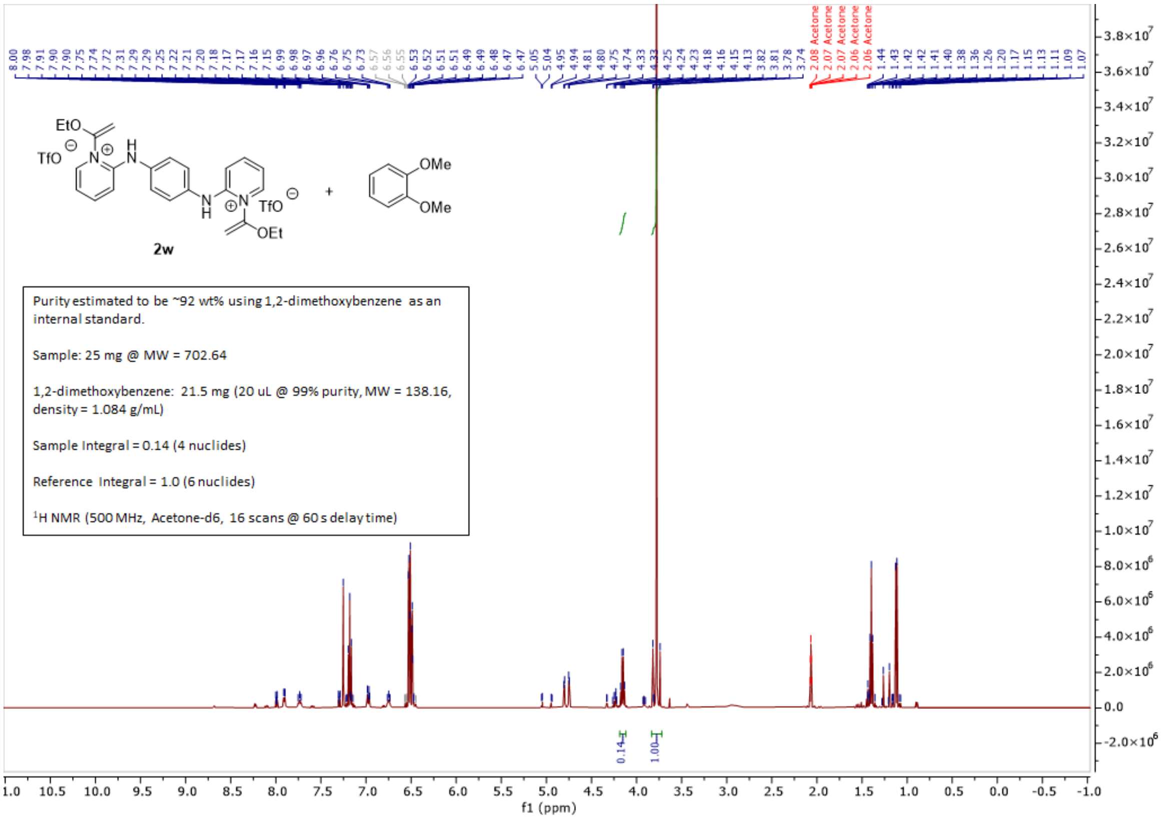

VI. Images of ^{1}H NMR spectra of known compounds prepared by current methods

(Listed in Numerical Order)

^{1}H NMR Spectrum of **1a** (500 MHz, CD_3CN)

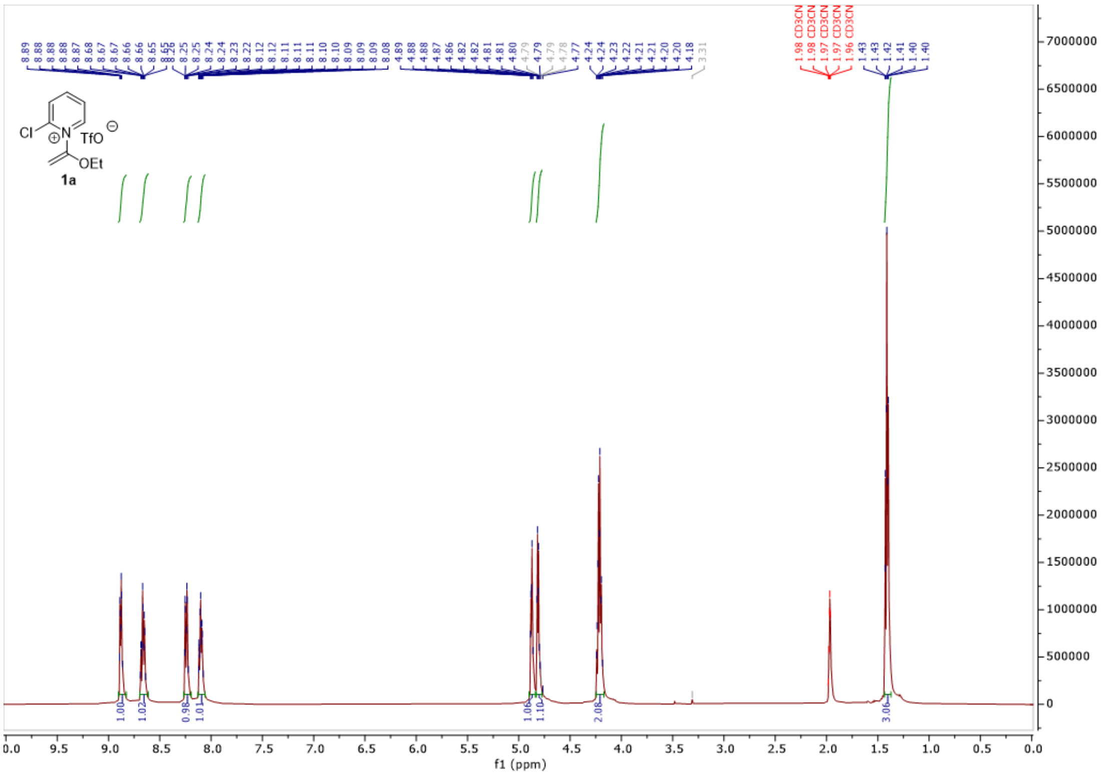

^{1}H NMR Spectrum of **2a** (500 MHz, Acetone-d_6)

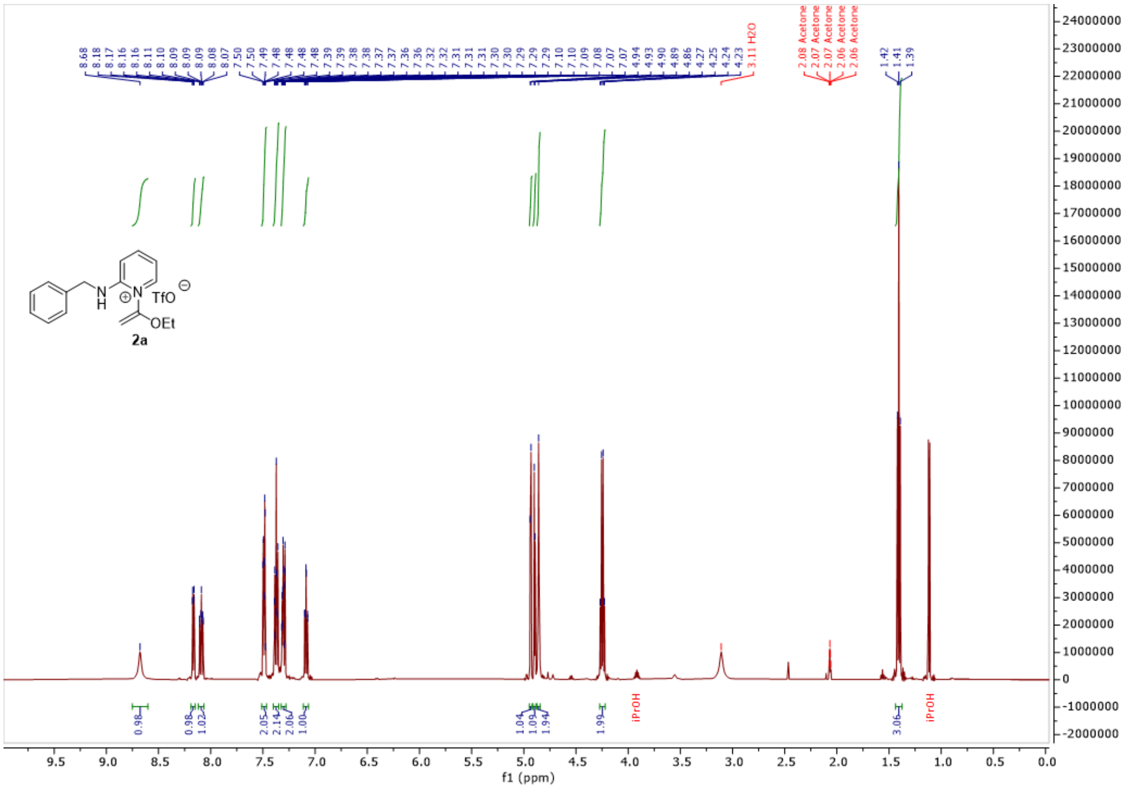

^{1}H NMR Spectrum of **2b** (500 MHz, Acetone-d_6)

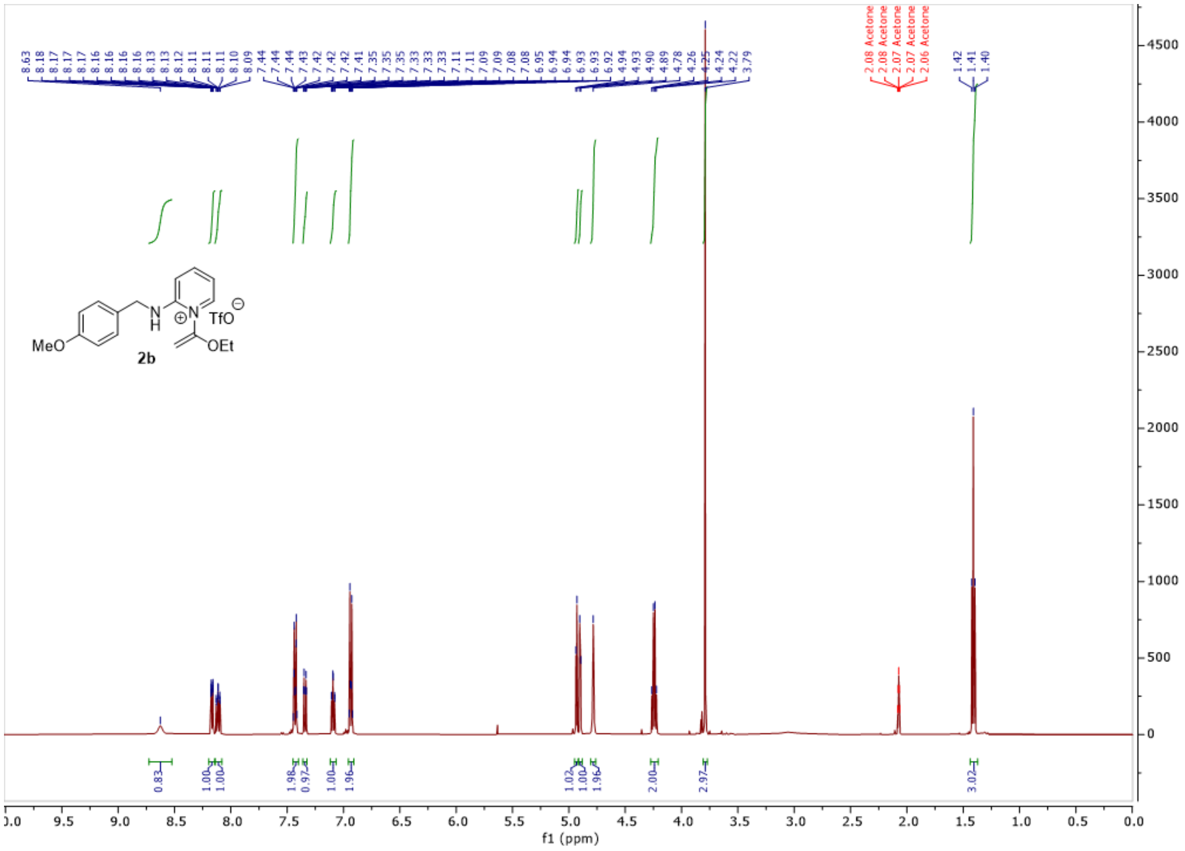

^{1}H NMR Spectrum of **3** (500 MHz, Acetone-d_6)

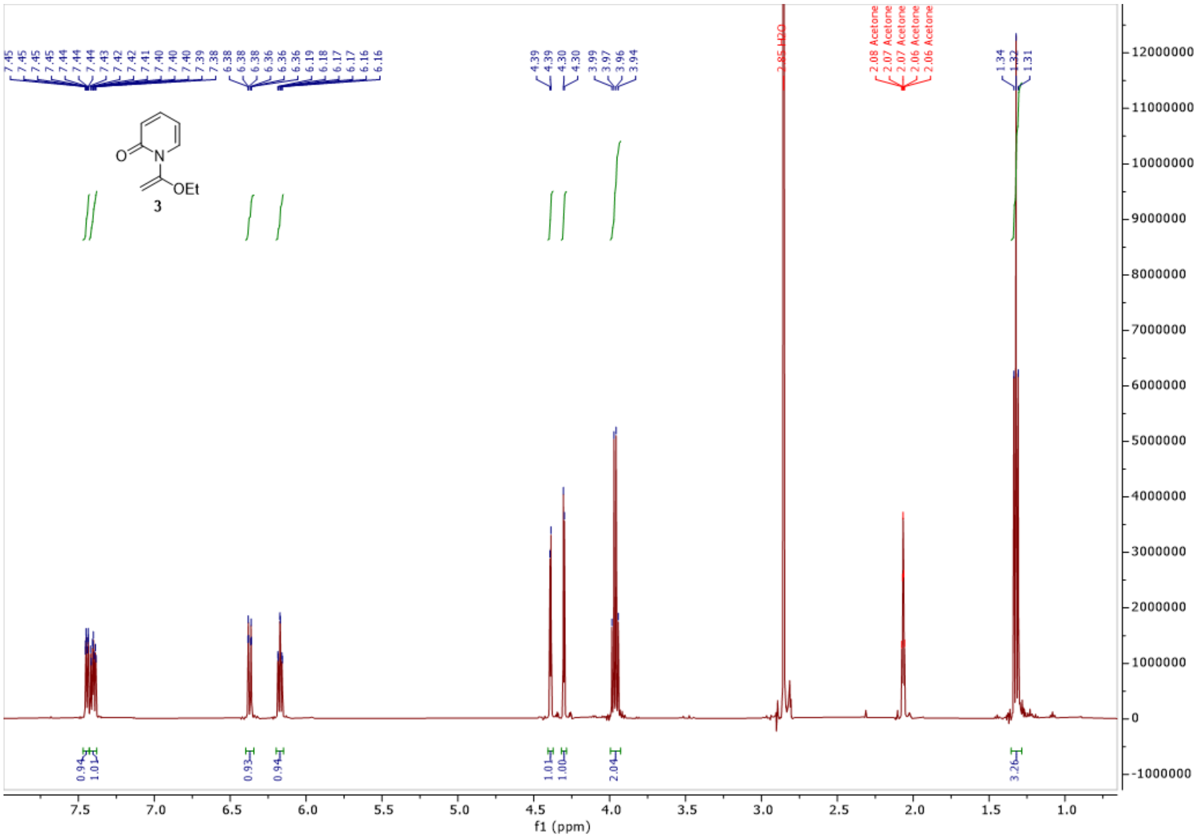

^{1}H NMR Spectrum of **6a** (500 MHz, Acetone-d_6)

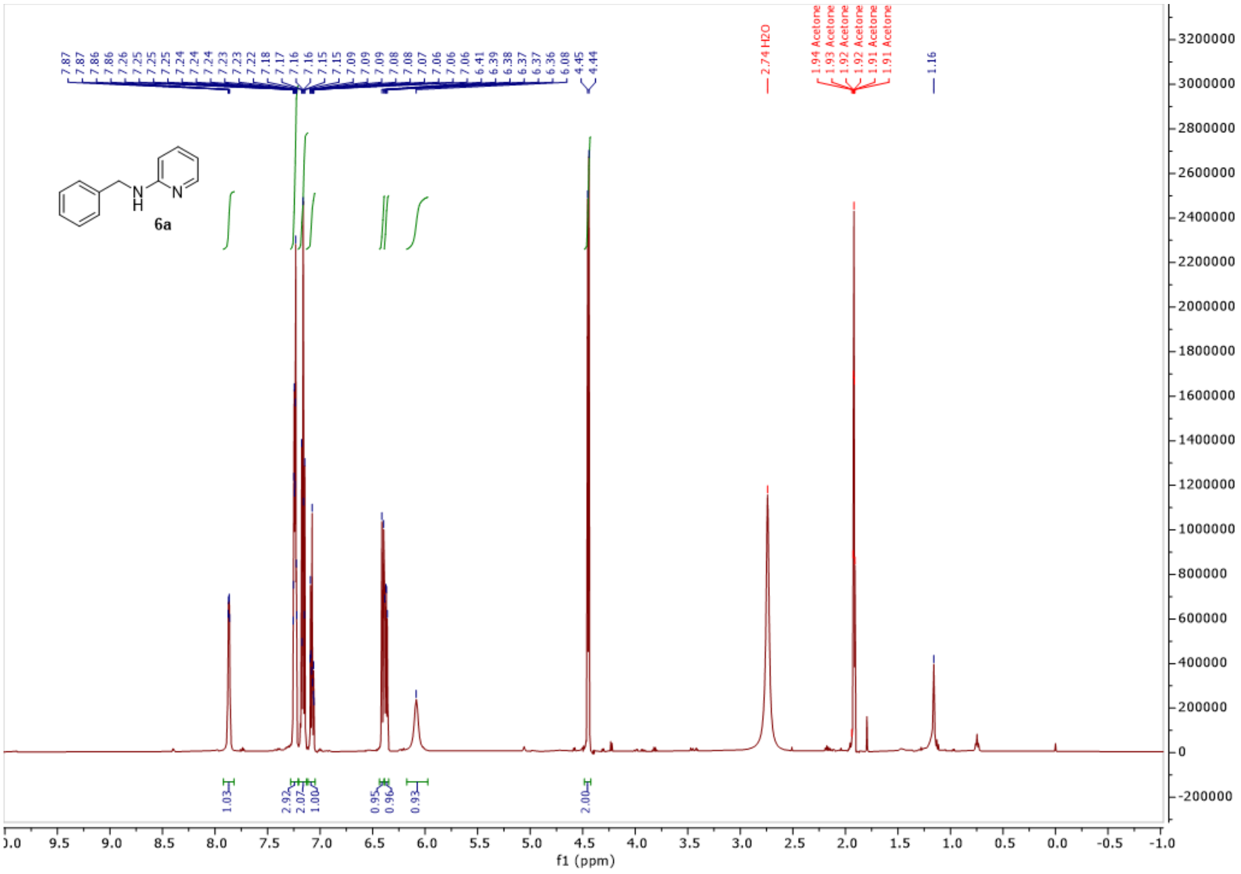

^1H NMR Spectrum of **6q** (500 MHz, Acetone-d_6)

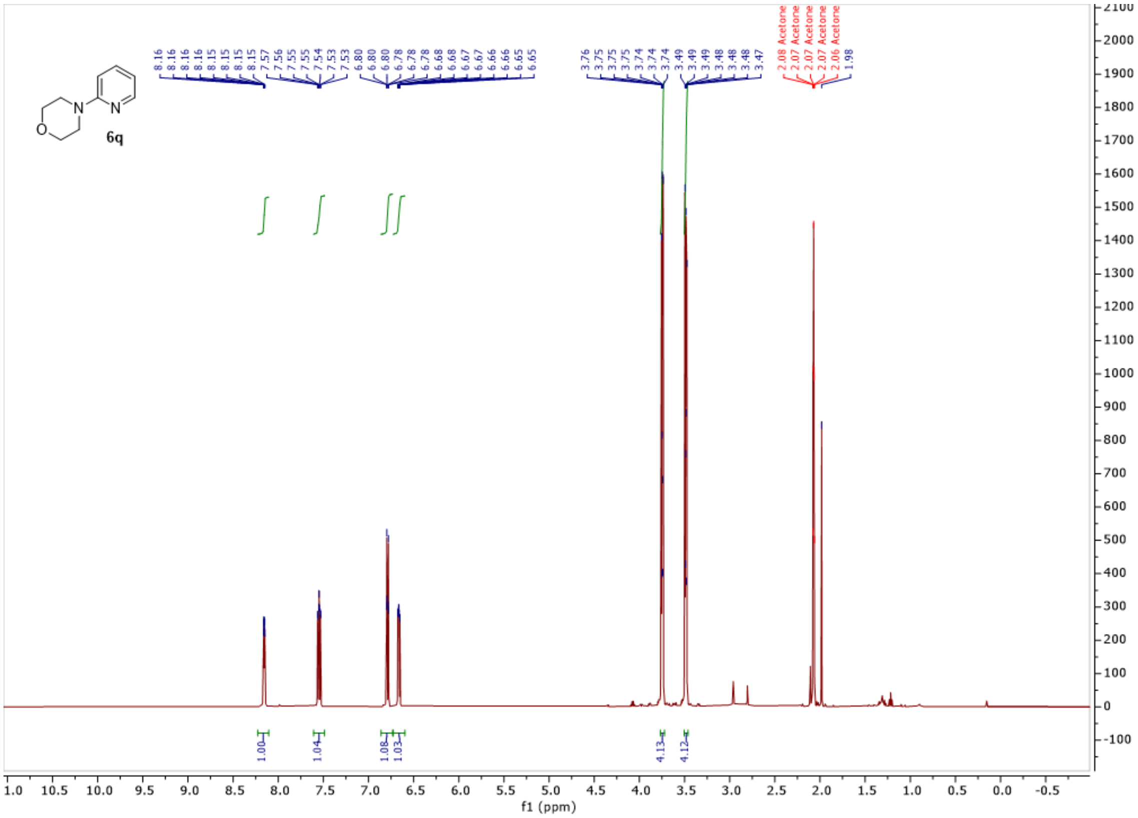

^1H NMR Spectrum of **7a** (500 MHz, DMSO-d_6)

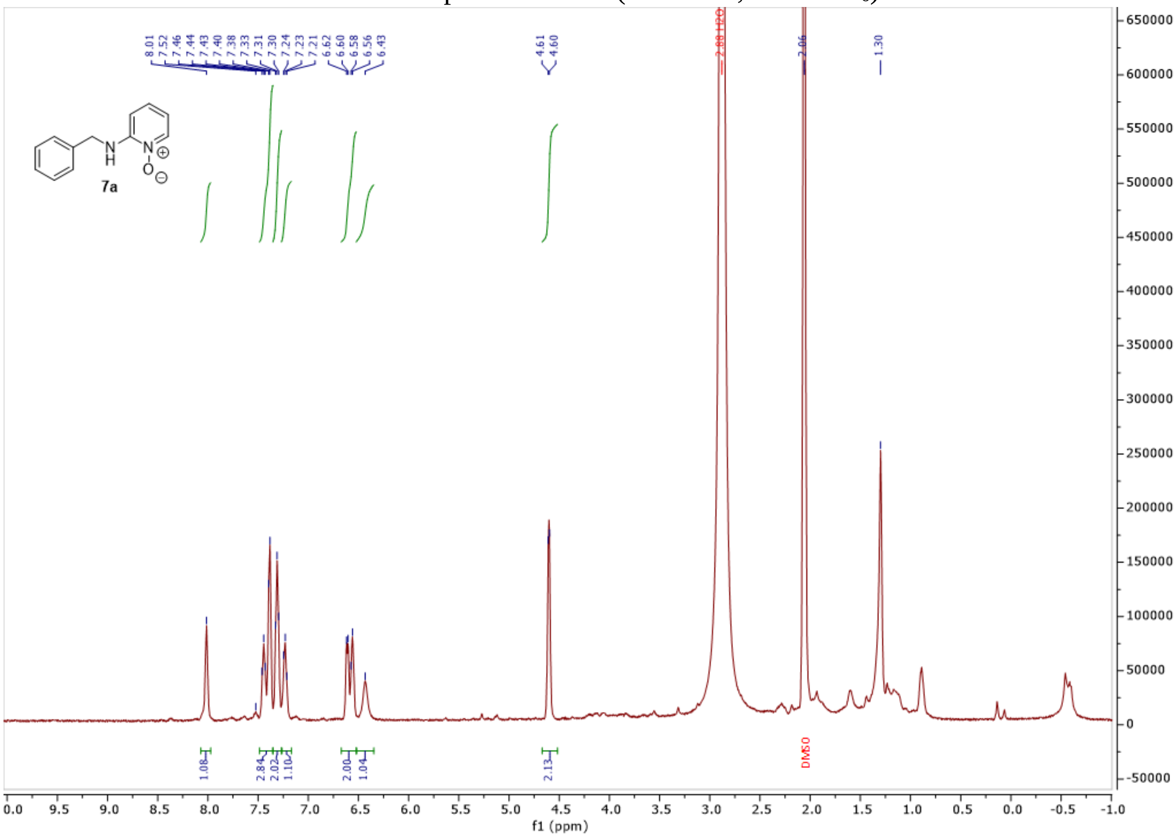

^{1}H NMR Spectrum of **12** (500 MHz, $CDCl_3$)

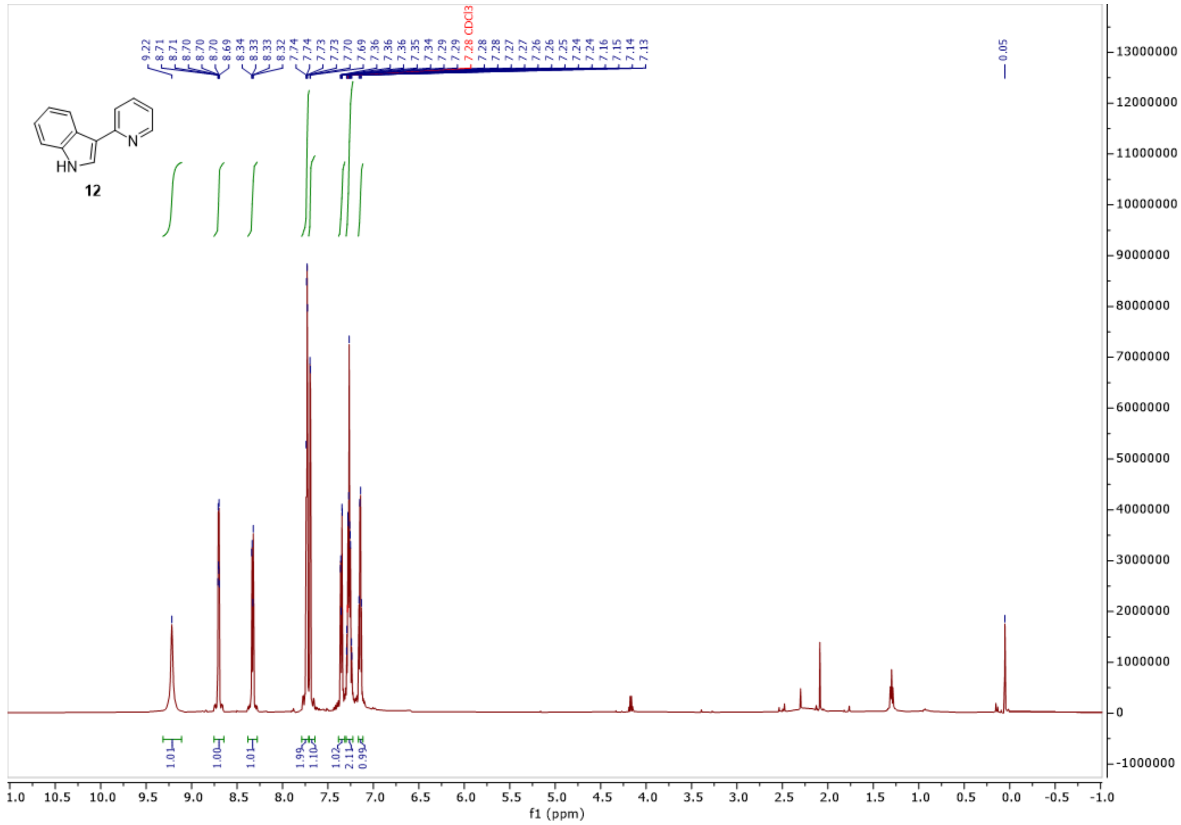

^{1}H NMR Spectrum of **13** (500 MHz, $CDCl_3$)

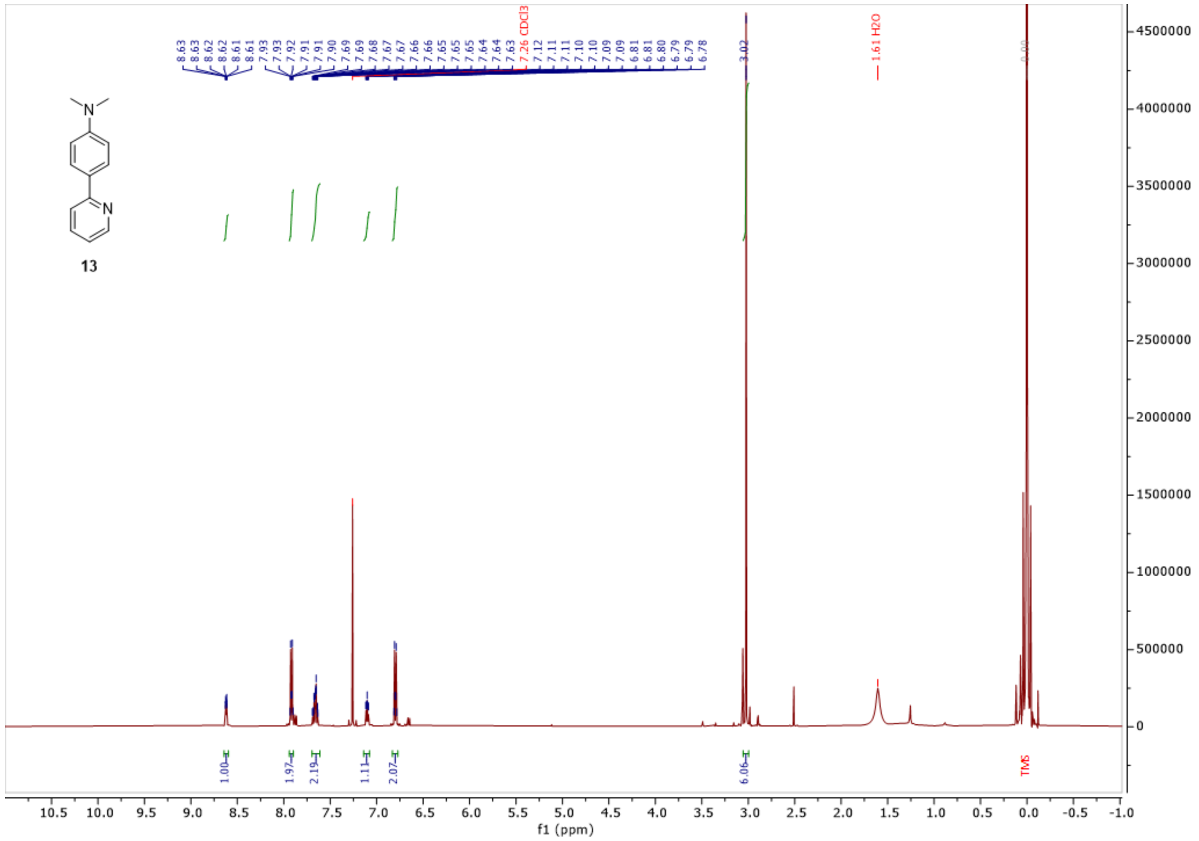

VII. References:

[1] McConnell, D. L.; Blades, A. M.; Rodrigues, D. G.; Keyes, P. V.; Sonberg, J. C.; Anthony, C. E.; Rachad, S.; Simone, O. M.; Sullivan, C. F.; Shapiro, J. D.; Williams, C. C.; Schafer, B. C.; Glanzer, A. G.; Hutchinson, H. H.; Thayaparan, A. T.; Krevlin, Z. A.; Bote, I. C.; Haffaray, Y. A.; Bhandari, S.; Goodman, J. A.; Majireck, M. M. Synthesis of Bench-stable *N*-Quaternized Ketene *N*,*O*-Acetals and Preliminary Evaluation as Reagents in Organic Synthesis. *J. Org. Chem.* **2021**, *86*, 13025-13040.

[2] Frank, J. P.; Strutton, W. R.; Adade, J. K. A.; Majireck, M. M. Preparation of 2-Chloro-1-(1-ethoxyvinyl)pyridinium Triflate. *Org. Synth.* **2024**, *101*, 242-257.

[3] Krevlin, Z. K.; Bote, I. C.; Crespo, M. C. F.; Lam, C.; McMillen, C. D.; Majireck, M. M. Synthesis and crystal structure of a bench-stable pyridinium ketene hemiaminal, 1-(1-ethoxyvinyl)-2-(methyl(phenyl)amino)pyridin-1-ium trifluoromethanesulfonate. *Acta Cryst. E.*, **2023**, *79*, 698-701.

[4] Donthireddy, S. N. R.; Pandey, V. K.; Rit, A. [$(PPh_3)_2NiCl_2$]-Catalyzed C–N Bond Formation Reaction via Borrowing Hydrogen Strategy: Access to Diverse Secondary Amines and Quinolines. *J. Org. Chem.* **2021**, *86*, 6994-7001.

[5] Buchwald, S. L.; Wagaw, S. The Synthesis of Aminopyridines: A Method Employing Palladium-Catalyzed Carbon−Nitrogen Bond Formation. *J. Org. Chem.* **1996**, *61*, 7420-7241.

[6] Liu, W.; Xu, K.; Chen, X.; Zhang, F.; Xu, Z.; Wang, D.; He, Y.; Xia, X.; Zhang, X.; Liang, Y. CuI/2-Aminopyridine 1-Oxide Catalyzed Amination of Aryl Chlorides with Aliphatic Amines. *Org. Lett.* **2020**, *22*, 7486-7490.

[7] Harrison, D. P.; Welch, K. D.; Neilander, A. C.; Sabat, M.; Myers, W. H.; Harman, W. D. Efficient Synthesis of an η2-Pyridine Complex and a Preliminary Investigation of the Bound Heterocycle's Reactivity. *J. Am. Chem. Soc.* **2008**, *130*, 16844–16845.

[8] Gosmini, C. A.; Bassene-Ernst, C.; Durandetti, M. Synthesis of functionalized 2-arylpyridines from 2-halopyridines and various aryl halides via a nickel catalysis. *Tetrahedron* **2009**, *65*, 6141-6146.
